# Stereospecific Synthesis of Cyclohexenone Acids by [3,3]-Sigmatropic Rearrangement Route

**DOI:** 10.1021/acs.joc.3c00757

**Published:** 2023-09-01

**Authors:** Aleksi Eronen, Martin Nieger, Tommi A. Kajander, Timo Repo

**Affiliations:** † Department of Chemistry, University of Helsinki, PO Box 55 (A. I. Virtasen aukio 1), 00014, Helsinki, Finland; ‡ Institute of Biotechnology, University of Helsinki, PO Box 65 (Viikinkaari 1), 00014, Helsinki, Finland

## Abstract

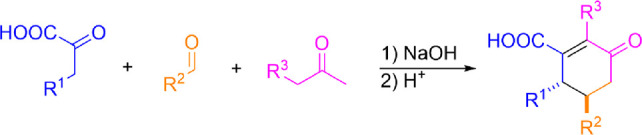

Herein we report a modular synthetic method for the preparation of diaryl-substituted cyclohexenone acids starting from phenyl pyruvate and suitable enones. When the reaction is carried out in alkaline *tert*-butanol or toluene solutions in microwave-assisted conditions mainly anti configuration products are obtained with up to 86% isolated yield. However, when the reaction is carried out in alkaline water, a mixture of products with anti and syn conformations is obtained with up to 98% overall isolated yield. Mechanistically the product with anti conformation forms by a hemiketal–oxy-Cope type [3,3]-sigmatropic rearrangement–intramolecular aldol condensation route and syn product by an intermolecular aldol condensation-electrocyclization (disrotatory type) route.

## Introduction

Chiral cyclohexenones are core structures in many natural products and pharmaceuticals,^1−7^ and therefore important targets for synthetic chemistry.^5−11^ Commonly employed synthetic strategies for chiral cyclohexenones include Robinson annulations,^12,13^ Michael additions,^14−16^ and aldol condensations^15,17^ together with recent examples including homo-Nazarov cyclization^18^ and 1,3 protonic shift.^19^ [3,3]-sigmatropic rearrangements are a convenient way to achieve regio- and stereoselective C–C and C–X bond forming reactions in organic chemistry.^20−24^ Notably, the [3,3]-sigmatropic rearrangement approach is largely an unexplored option for cyclohexenone synthesis.

**Figure 1:**
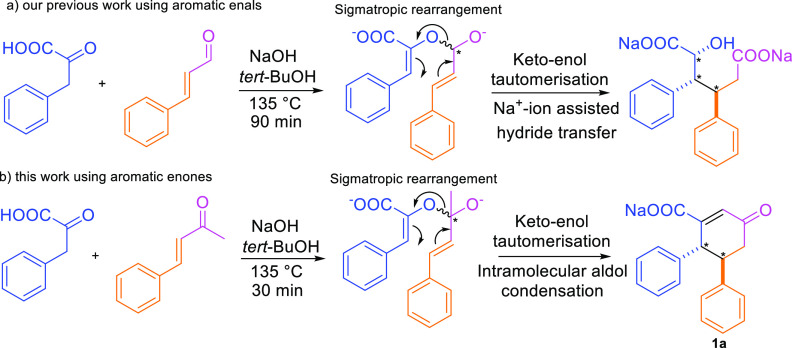
Utilization of [3,3]-sigmatropic rearrangement of phenylpyruvic acid under microwave-assisted conditions with (a) cinnamaldehyde into sodium salt of dicarboxylic acid.^25^ (b) 4-Phenyl-3-buten-2-one into sodium salt of cyclohexanone acid (**1a**) (this work).

**Figure 2:**
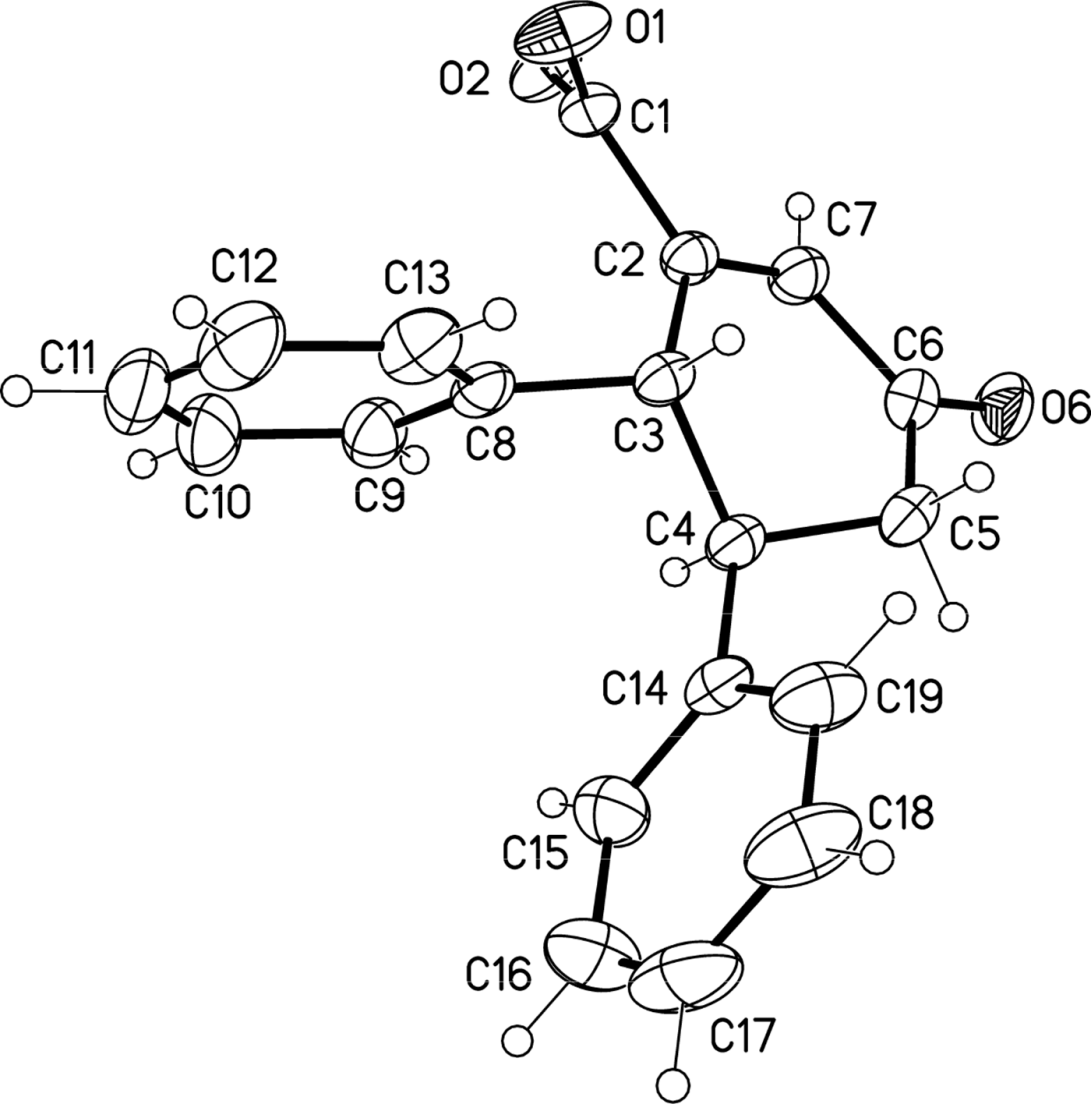
Molecular structure of the anion of the sodium salt of **1a** (displacement parameters are drawn at the 50% probability level). Stereocenters are RR and its enantiomer SS due to centrosymmetry (CDCC 2193907).

In our previous work,^25^ we successfully utilized [3,3]-sigmatropic rearrangement in dicarboxylic acid synthesis from phenylpyruvate and cinnamaldehyde under microwave-assisted conditions from alkaline alcohol solutions (Figure 1a). We report here that the substrate scope can be expanded toward aryl enones, and this subsequently opens a unique synthesis strategy toward substituted cyclohexanones. For example, a reaction between phenylpyruvate and 4-phenyl-3-buten-2-one generates 4,5-diphenyl-cyclohex-2-en-1-one-3-carboxylic acid (**1**) salt (Figure 1b). Regarding the product, there is only one preliminary report from 1956 by Krisensen-Reh, where its identification, let alone the reaction pathway, was not unambigous.^26^ Based on X-ray crystallography herein, the reaction proceeds in a diastereospecific manner; stereocenters in **1a** are either RR or its enantiomer SS due to the centrosymmetry in the crystal structure (Figure 2) (CDCC 2193907). As shown earlier by us, the diastereospecificity in this new synthetic strategy toward chiral cyclohexenones arises from the elemental reaction step, [3,3]-sigmatropic rearrangement.^25^

## Results and Discussion

**Figure 3:**
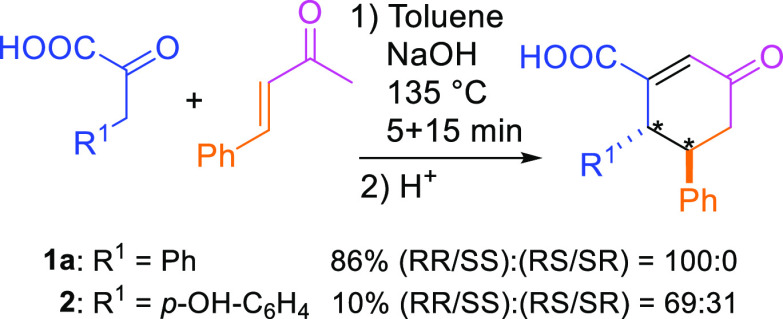
Formation of cyclohexenone acids from aromatic pyruvates and presynthesized aromatic enone (4-phenyl-3-buten-2-one). Given isolated yields are calculated from pyruvic acid.

In our previous work, phenylpyruvic acid was successfully used with aromatic enal (cinnamaldehyde) in the dicarboxylic acid synthesis. The reaction proceeds through a hemiacetal intermediate;^25^ however, it remained uncertain if sterically more demanding aromatic enones could also be utilized in the synthesis. We explored the reaction between phenylpyruvic acid and aryl enone (4-phenyl-3-buten-2-one) under different reaction conditions. When the alkaline toluene solution was heated under microwave-assisted conditions (135 °C, 15 min), a crystalline beige/yellowish powder was obtained after cooling. After acetone washing, a sodium salt of **1a** (4,5-diphenyl-cyclohex-2-en-1-one-3-carboxylic acid) was isolated at 86% yield, and its solid-state structure was determined (Figure 2). Similarly, when 4-hydroxy-phenylpyruvic acid was used instead of phenylpyruvic acid, product 4-(*para*-phenol)-5-phenyl-cyclohex-2-en-1-one-3-carboxylic acid (**2**) is obtained at 10% isolated yield (racemic mixture (RR/SS):(RS/SR) = 69:31). The presence of *para*-phenol R^1^ in **2** indicates that the R^1^ substituent originates from the pyruvate acid and the second phenyl ring originates from the enone (Figure 3). This observation is in line with our previous work, giving R^1^ next to the acid group. Mechanistically the reaction is assumed to proceed through a hemiketal intermediate similar to our previous dicarboxylic acid synthesis.^25^

**Figure 4:**
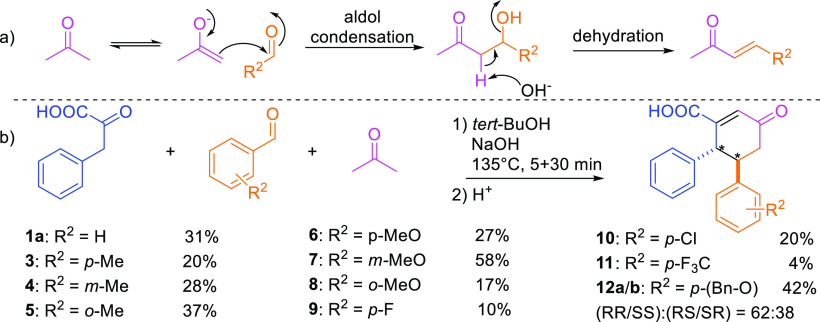
(a) *In situ* formation of aromatic enones from acetone and aromatic aldehydes. (b) Reaction conditions: α-ketoacid (2.0 mmol), aromatic aldehyde (2.0 mmol), acetone (2.0 mmol), NaOH (3 mmol, 10 M at H_2_O solution), *tert*-butanol (3 mL); microwave vial was heated to 135 °C in 5 min and kept at 135 °C for 30 min. Products emerge as an enantiomeric pair of RR and SS. Yields are isolated yields calculated from phenylpyruvic acid.

To further expand the substrate scope, we investigated a series of reactions in which aromatic enones were synthesized *in situ* from different benzaldehydes and acetone via aldol condensation (Figure 4a). When combined with phenylpyruvic acid, this approach grants a straightforward way to synthesize unprecedented cyclohexenone acid derivatives with moderate yields simply by altering the aromatic aldehyde (Figure 4b). The ease of the *in situ* process is offset by lower yields, for example, giving 31% isolated yield of **1a** compared to 86% yield when using the presynthesized aryl enone.

The reaction tolerates halogen functionalities including fluoride, chloride, and trifluoromethyl substitution resulting in, under *in situ* generated enone conditions, products **9**, **10**, and **11** with 10%, 20%, and 4% isolated yields, respectively. Sterically demanding substituents are also compatible within the reaction, which was demonstrated with 4-(benzyloxy)benzaldehyde, giving 4-phenyl-5-(4-benzyloxy)phenyl-cyclohex-2-en-1-one-3-carboxylic acid (**12**) with an isolated 42% ((RR/SS, anti):(RS/SR, syn) = 62:38 stereocenters) yield.

**Figure 5:**
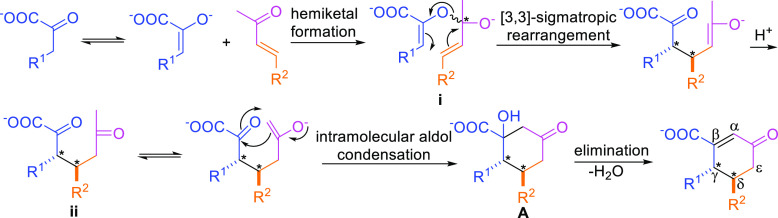
Formation of cyclohexenone acid. Enolate tautomer of pheylpyruvic acid forms hemiketal **i** with an aromatic enone. Intermediate **i** undergoes oxy-Cope type [3,3]-sigmatropic rearrangement, and after H^+^ transfer, the intermediate **ii** is formed. Intermediate **ii** undergoes intramolecular aldol condensation (**A**) and after OH group elimination, the final cyclohexenone acid product is obtained as sodium salt.

Mechanistically, the reaction commences when an enolate tautomer of phenylpyruvic acid attacks the aromatic enone and forms a hemiketal intermediate (**i**, Figure 5). The reaction continues via oxy-Cope type [3,3]-sigmatropic rearrangement followed by H^+^ transfer, generating a diketone intermediate **ii**, which bears pyruvate acid and ketone functionalities. This intermediate undergoes intramolecular aldol condensation (**A**) and, through the elimination of water, yields the desired cyclohexenone acid product as a sodium salt.

Similarly to our previous work,^25^ the oxy-Cope type [3,3]-sigmatropic rearrangement is the source of diastereospecifity. The formation of certain diastereomer pairs is favored when the phenyls of phenylpyruvic acid and the aromatic enone do not overlap during the formation of intermediate **i**. Thus, when enolate tautomer of phenylpyruvic acid (E or Z) attacks the prochiral aromatic enone from either the *re* or *si* face, the hemiketal **i** with R configuration in the chiral center gives **ii** as an SS diastereomer with E enolate or RR diastereomer with Z enolate. Likewise, hemiketal **i** with S configuration gives **ii** as the RR diastereomer with E enolate or the SS diastereomer with Z enolate. Interestingly, when the aryl enone contains a large substituent such as a benzyloxy group in **12a**/**b**, similar to compound **2** the stereochemical preference is disturbed and RS/SR diastereomers are also observed (38% ratio of total yield); see below.

**Figure 6:**
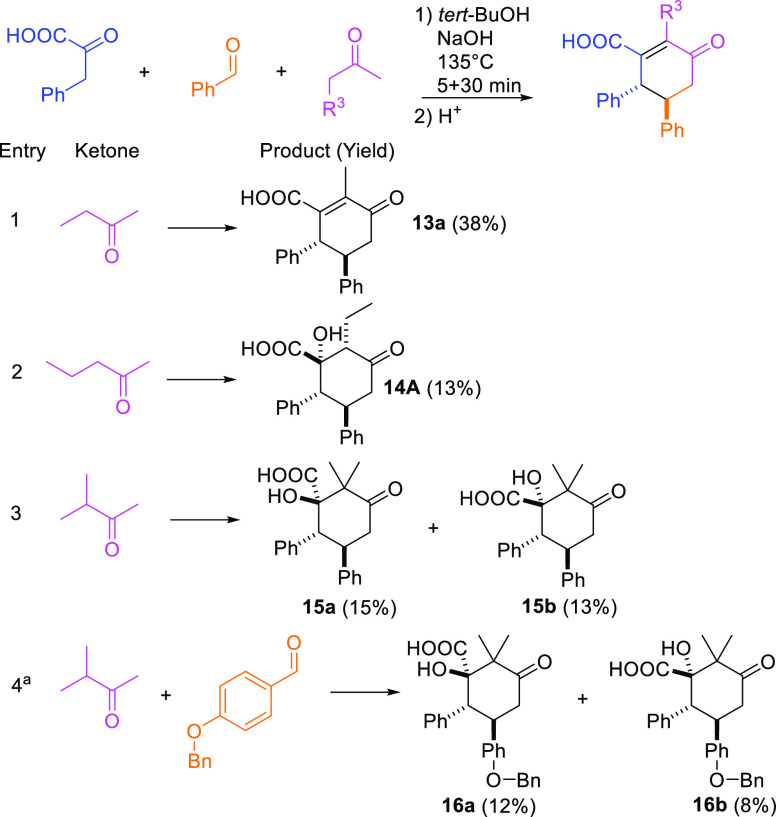
Synthesis of Cyclohexenone Acids with extended ketone (R^3^). Reaction conditions: α-ketoacid (2.0 mmol), benzaldehyde (2.0 mmol), ketone (2.0 mmol), NaOH (3 mmol, 10 M at H_2_O solution), and *tert*-butanol (3 mL); microwave vial was heated to 135 °C in 5 min and kept at 135 °C for 30 min. ^a^Entry 4: 2 mmol of 4-(benzyloxy)benzaldehyde instead of benzaldehyde.

**Figure 7:**
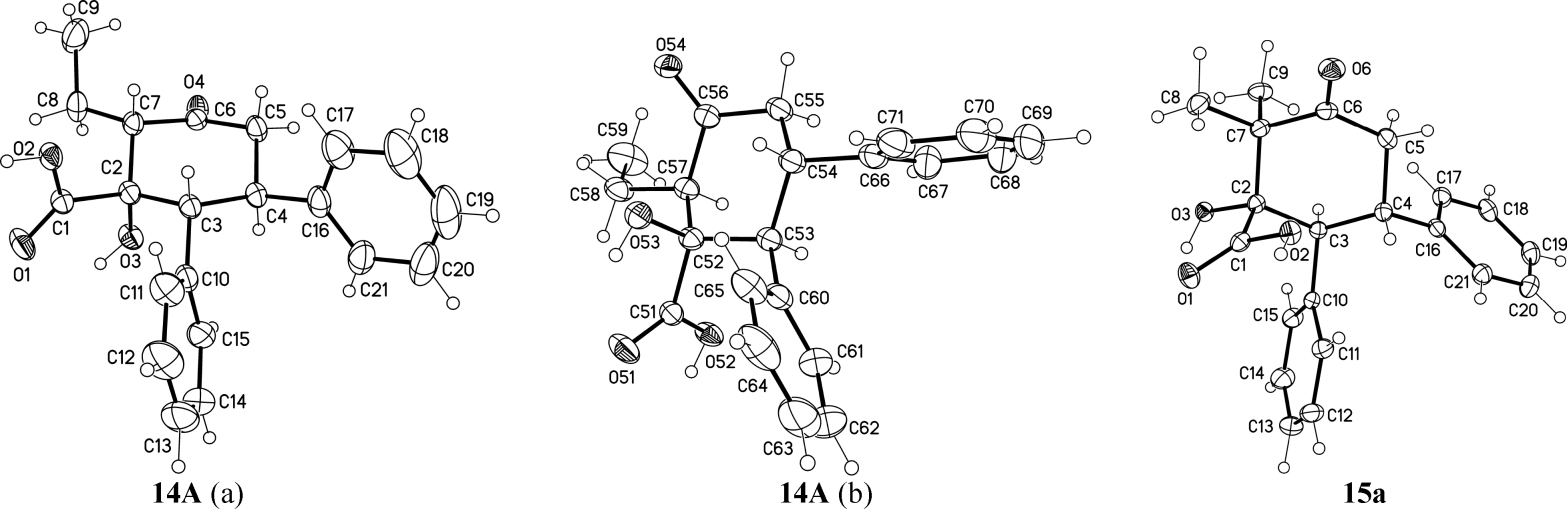
Molecular structures of 1st (a) and 2nd (b) crystallographic independent molecule of **14A** (displacement parameters are drawn at 50% probability level) (CDCC 2193908, stereocenters: 1S, 2S, 5R, 6R and 1R, 2R, 5S, 6S) and molecular structure of **15a** (solvent water omitted for clarity, displacement parameters are drawn at 50% probability level) (CDCC 2193909) with RRR and its enantiomer pair SSS.

When *in situ* synthesis of cyclohexenone acids is performed using 2-butanone instead of acetone, a methyl group at the α-position is introduced, and this opens further possibilities to tailor the cyclohexanone structures (**13a**, Figure 6, R^3^). For example, following this observation and replacing acetone with 2-pentanone, an ethyl group can be introduced at the α-position instead. Interestingly, the product 2-ethyl-1-hydroxy-3-oxo-5,6-diphenyl-cyclohexane-1-carboxylic acid **14A** does not undergo the final dehydration step (Figure 5). The crystal structure of **14A** shows the ethyl group at the α carbon in an equatorial position and the OH group at the β carbon in an axial position (Figure 7, **14A**). Most likely the predicted OH-elimination cannot occur due to the increased steric hindrance introduced by the ethyl group. On the other hand, the remaining OH group in **14A** is further evidence of the ring closure by the intramolecular aldol condensation reaction.

The crystal structure of **14A** also exhibits centrosymmetry. The results suggest that the intramolecular aldol condensation is stereospecific when the R^3^ group is suitably sterically demanding. A stark contrast is obtained when the synthesis is performed with 3-methyl-2-butanone. Two different enantiomeric pairs are obtained (RRR and SSS (**15a**)) and (1S, 5R, and 6R and 1R, 5S, and 6S (**15b**)) at nearly a 1:1 ratio. As the α carbon is missing a proton, OH group elimination cannot occur. Thus, products **15a** and **15b** also contain an OH group, as a result. We expected the stereochemistry of the β carbon to behave similarly to product **14A**, whereby the carboxylic acid group would be at the equatorial position as it is a larger and more demanding functionality. However, the solid state crystal structure of product **15a** clearly has the carboxylic acid group at the axial position (Figure 7, **15a**). Evidently the carboxylic acid group does not provide enough driving force to guide the aldol condensation to be stereospecific in this reaction, when the α-position does not contain additional steric guidance.

Interestingly, when 3-methyl-2-butanone is reacted with the sterically demanding group 4-(benzyloxy)benzaldehyde (Figure 6 entry 4) the products **16a** and **16b** are obtained. Stereocenters of **16a** and **16b** at γ and δ carbons only exist in the anti position with respect to each other, and no RS/SR epimers are observed unlike with compounds **2** and **12**. Instead the compounds follow the same stereocenter pattern as **15a** and **15b**.

**Figure 8:**
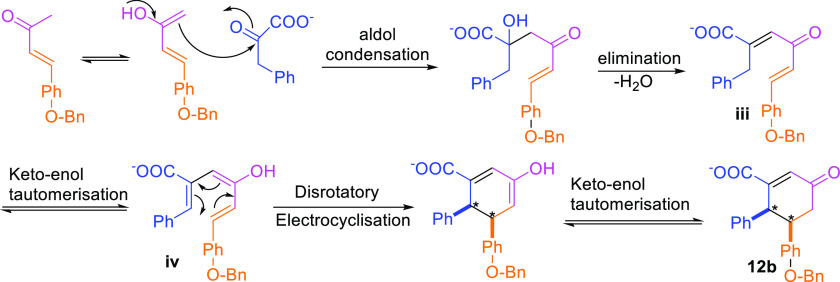
Alternative pathway for the formation of cyclohexenone acid (an example is for **12b**). Enol form of aromatic enone undergoes aldol condensation with phenylpyruvic acid. After elimination, the intermediate **iii** undergoes keto–enol tautomerization into intermediate **iv**, followed up by electrocyclisation. Final product is obtained after keto–enol tautomerisation. As intermediate **iv** undergoes electrocyclization by a disrotatory pathway, the final product is obtained as a syn conformation.

We propose that, in product **12**, the bulky side group -C_6_H_4_-O-Bn from 4-(benzyloxy)benzaldehyde makes the hemiketal formation **i** or [3,3]-sigmatropic rearrangement energetically more demanding. The *in situ* formed enone undergoes keto–enol tautomerization and opts for intermolecular aldol condensation with phenylpyruvic acid (Figure 8). Followed by elimination of water, intermediate **iii** is formed. Next, **iii** undergoes keto–enol tautomerization into intermediate **iv**. The reaction proceeds to an electrocyclization reaction in a disrotatory fashion. A similar reactivity pattern is supported by earlier work from Isoe et al.^27,28^ Disrotatory electrocyclization reaction forces the phenyl groups into the syn configuration. This explains formation of epimers RS/SR. As when **16a**/**b** is synthesized the additional sterics from 3-methyl-2-butanone as the enone constituent, this makes intermolecular aldol condensation unfavorable. This pushes the reaction mechanism back to the [3,3]-sigmatropic rearrangement despite the sterically demanding side group. Similarly, in product **2** we suspect that the electron donating phenolic group encourages pyruvic acid to remain in the ketone form rather than in the enol/enolate form. This makes intermolecular aldol condensation more likely to take place and opens up the system to generate the syn (RS/SR) epimeric pairs as well.

Water is often used as a solvent in the aldol condensation reactions. When the synthesis of **1** (Table 1, entry 1) is performed in water instead of *tert*-BuOH or toluene, the overall yield of the reaction rises to near quantitative (96%). However, water as a reaction medium also promotes formation of **1b** (RS/SR) having a syn-configuration with a partial yield of 36%. Usage of water opens the intermolecular aldol condensation-electrocyclization pathway described in Figure 8 even for examples that previously did not undergo it.

**Table 1:** Synthesis of Cyclohexenones in Water

Entry	Synthesis of	Conditions^a^	Yield (%) (anti:syn)
1	**1**^b^	H_2_O, 135 °C	96 (64:36)
2	**13**	H_2_O, 135 °C	98 (72:28)
3	**14**	H_2_O, 135 °C	98 (75:25)
4	**15**	H_2_O, 135 °C	90 (**15a**:**15b** 67:33), no syn products
5	**12**	H_2_O, 135 °C	83 (62:38)
6	**1**^b^	H_2_O, RT^c^	96 (**1A**, 100:0)
7	**1**^b^	Brine,^d^ 135 °C	94 (95:5)

a Reaction conditions: α-ketoacid (2.0 mmol), benzaldehyde (2.0 mmol), ketone (2.0 mmol), NaOH (3 mmol, 10 M at H_2_O solution), H_2_O (3 mL); microwave vial was heated to 135 °C and heat was maintained for 30 min.

b 4-Phenyl-3-buten-2-one (2 mmol) is used instead of benzaldehyde and ketone.

c Reaction was stirred for 4 h at room temperature at 20 mL vial.

d Brine solution (3 mL, saturated NaCl solution) was used instead of Milli-Q H_2_O.

The usage of water as a solvent also benefits the *in situ* formation of aromatic enones. When synthesis of **13** (Table 1, entry 2) is carried out in water, the overall isolated yield was near quantitative 98% and syn (RS/SR) product (**13b**) is obtained with 28% of overall yield. Interestingly, for the synthesis of **14** (Table 1, entry 3) in water, the reaction went into completion to **14a**:**14b** (75:25) with 98% overall yield. Rather than stopping at stage **14A** (Figure 6, entry 2) like in the above case, the reaction was carried out in *tert*-BuOH solution. For the synthesis of **15** (Table 1, entry 4) in water, high overall yields are obtained (90%; **15a**:**15b** = 67:33). But, as observed previously the enone constituent with a terminal isopropyl group is unable to undergo the intermolecular aldol condensation–electocyclisation pathway, and thus, a syn product does not form. Curiously, when the synthesis of **12** (Table 1, entry 5) is carried out in water, a high yield is obtained (83%) but the ratio between anti (**12a**) and syn (**12b**) stayed the same (62:38) as when the synthesis was done at *tert*-BuOH (Figure 4). Product, which was already undergoing the syn formation pathway, did not get additional benefits from the water for this pathway.

Interestingly, the formation ratio of the syn product has a negative correlation with the stericity of the enone constituent (Table 1, entries 1–4). The least hindered enone constituent yields the highest syn ratio (36%), while the extreme example in the series, the isopropyl-terminated enone component, does not allow for the formation of the syn product (**15**) at all. Thus, it is clear that in water the syn products are coming from the intermolecular aldol condensation-electocyclization pathway described in Figure 8.

Curiously, when the synthesis of **1** is carried out at room temperature in water (Table 1, entry 6), the reaction stops at intermediate **A** (Figure 5) prior to final hydroxyl group elimination. The product **1A** is obtained with near quantitative isolated yield (96%) and only as an anti (RR/SS) conformer. This result suggests that the hemiketal–[3,3]-sigmatropic rearrangement route proceeds through a lower energy pathway than the intermolecular aldol condensation–electrocyclization route. Interestingly, the result also suggests that the intramolecular aldol condensation (Figure 5) is stereospecific at room temperature. Accordingly, the carboxylic acid group directs itself to the equatorial position at β carbon and leaves the hydroxyl group to the axial position.

When the synthesis of **1** is carried out in a hot brine solution (Table 1, entry 7) high yields are still obtained (94%), but the syn product ratio dropped to 5%. Most likely, the increased Na^+^-ion concentration is stabilizing the enolate form of phenylpyruvic acid. This promotes hemiketal formation and the subsequent [3,3]-sigmatropic rearrangement reaction over the intermolecular aldol condensation–electrocyclization pathway even in the water.

## Conclusions

To summarize, we have developed a new strategy for cyclohexenone synthesis by utilizing pyruvates with aromatic enones. Anti conformers of cyclohexenone acids are obtained *via* the hemiketal–oxy-Cope type [3,3]-sigmatropic rearrangement–intramolecular aldol condensation pathway and syn conformers *via* the intermolecular aldol condensation–electrocyclisation pathway. Products form in a diastereospecific manner, with typically two but up to four stereogenic centers. High yields can be obtained in H_2_O, but with a mixture of anti and syn conformers, in organic solvents the conditions favor anti conformer formation although with lower overall yields. As shown, the structure of cyclohexenone acids can be varied in a straightforward manner, by choice of pyruvate (R^1^), aromatic aldehyde (R^2^) and ketone (R^3^). The synthesis tolerates a variety of functional groups, and cyclohexenone acids contain a multitude of functionalities such as ketone, carboxylic acid, and double bonds variants. Thus, cyclohexenone acids reported here could be utilized as building blocks for further synthesis.

## Experimental Section

### General Information

All chemicals were obtained from commercial sources and used as such without further purification. ^1^H, ^13^C{^1^H}, ^19^F, HSQC, and HMBC NMR spectra were recorded with an Avance Neo (500 MHz, 25 °C) NMR spectrometer by Bruker. Recorded spectra were calibrated by solvent signals (acetone-d6: ^1^H 2.050 ppm, ^13^C 29.840 ppm; DMSO-d6: ^1^H 2.500 ppm, ^13^C 39.520 ppm; D_2_O: ^1^H 4.790 ppm). ^19^F NMR chemical shift axis was indirectly referenced via calculating ^19^F 0 ppm from the solvent lock frequency. All spectra were processed with MestReNova software. High-resolution mass spectra were measured by Bruker microTOF-MS in negative ion mode. Sodium formate was used as a calibrant. The samples were diluted to 1 ppm concentration with acetonitrile and filtered with syringe filters prior to measurements. IR spectra were measured with an Alpha ATR-FTIR by Bruker. The single-crystal X-ray diffraction study was carried out on a Bruker D8 Venture diffractometer with a PhotonII CPAD detector at 173(2) K (**1a**, **14A**) or Rigaku XtaLAB Synergy-S with HyPIX-6000 detector at 123(2) K (**15a**) using Cu–Kα radiation (λ = 1.54178 Å). The synthesis of cyclohexenone acid salts were conducted with a microwave reactor Monowave 450 by Anton Paar. Experiments were conducted in G10 vials, which were capped by snap caps with Teflon-coated silicon septums. The temperature during synthesis was monitored by a surface IR sensor, which was calibrated by a Ruby thermometer—an internal temperature probe—frequently.

### General Procedure for Cyclohexenone Acid Synthesis from Presynthesized Enone

Pyruvic acid (2 mmol), 4-phenyl-3-buten-2-one (2 mmol), NaOH (3 mmol, 300 μL, 10 M), and toluene (2 mL) were charged into a microwave vial (10 mL). The vial was capped and heated to 135 °C in 5 min with a microwave reactor. The temperature was maintained for 15 min. Then the vial was cooled to 50 °C using compressed air (see microwave heating profile at Figure S1). Following this, acetone (5 mL) was added to the vial to initiate the crystallization. The next day, the formed powder was collected by filtration, washed with acetone, and dried under air. The obtained product was dissolved with water, and HCl (1 M) was added until the formation of a white powder stopped. The powder was filtered, washed with water, and dried under vacuum.

### General Procedure for Cyclohexenone Acid Synthesis from *in situ* Synthesized Enone

Pyruvic acid (2 mmol), benzaldehyde (2 mmol), ketone (2 mmol), NaOH (3 mmol), and *tert*-BuOH (3 mL) were added into a 10 mL microwave vial. The heating program was set to heat the microwave vial to 135 °C in 5 min and maintain the temperature for 30 min. The stirring speed was set to 600 rpm. Next, the microwave vial was cooled down to 50 °C with compressed air (see microwave heating profile at Figure S2). After the mixture cooled, acetone was added to initiate the crystallization overnight. The following day, the formed product salt was collected by filtration, washed with acetone, and dried under air. Then, the salt of the product was dissolved with water, and 1 M HCl was added until all the product had precipitated from the solution. The formed powder was filtered, washed with water, and dried under vacuum.

### General Procedure for Cyclohexenone Acid Synthesis in Water

Phenylpyruvic acid (2 mmol), enone (2 mmol) (in case of *in situ* synthesized enone corresponding ketone (2 mmol) and aromatic aldehyde (2 mmol) was used instead), NaOH (3 mmol, 300 μL, 10 M), and milli-Q water (3 mL) were charged into a microwave vial (10 mL). The vial was capped and heated to 135 °C as fast as possible with a microwave reactor. The temperature was maintained for 30 min. Then the vial was cooled to 50 °C using compressed air (see microwave heating profile at Figure S3). Following this, HCl (1M, ca. 4 mL) was added into the microwave vial until precipitation formation stopped. The next day, the formed powder was collected by filtration, washed with water and *n*-hexane, and dried under vacuum.

#### 4,5-Diphenyl-cyclohex-2-en-1-one-3-carboxylic Acid (**1a** and **1b**) (Enantiomeric Pair of RR/SS (anti), RS/SR (syn))

The synthesis of a mixture of anti and syn products was carried out according to the general procedure for cyclohexenone acid synthesis in water using phenylpyruvic acid (2 mmol, 328.3 mg) and 4-phenyl-3-buten-2-one (2 mmol, 292.4 mg). After reaction, the products were obtained as a mixture of **1a** and **1b** as a beige powder 562 mg (96%). According to ^1^H NMR measurement, the ratio of **1a** (RR/SS, anti):**1b** (RS/SR, syn) was 64:36.

##### Isolation of **1b**

After the reaction was performed in water, the water dissolved salt mixture of **1a** and **1b** was transferred into a 20 mL vial. Acetone, ca. 10 mL, was added into the vial. In the following 5 days crystals formed in the vial; this salt (180 mg) was filtered and washed with acetone (ca. 5 mL), and this first fraction was recognized as the salt of **1a**. Within the next week, another fraction of salt (60 mg) formed in the vial. This fraction was filtered and washed with acetone (ca. 5 mL). This fraction was identified as the **1b** salt. The salt was dissolved into water and protonated with HCl, filtered, washed with water, and dried in vacuum.

Synthesis of **1a** by the general procedure from presynthesized enone: The product was synthesized from phenylpyruvic acid (2 mmol, 328.3 mg) and from presynthesized enone 4-phenyl-3-buten-2-one (2 mmol, 292.4 mg). After reaction, the product was isolated as a brownish beige salt (541 mg, 86%). The product was then dissolved with water, and HCl (1 M) was added until formation of the white powder stopped. The powder was filtered, washed with water, and dried under vacuum. The isolated protonated yield was 425 mg (73%).

Synthesis of **1a** by the general procedure for *in situ* synthesized enone phenylpyruvic acid (2 mmol, 328.3 mg), acetone (2 mmol, 150 μL), and benzaldehyde (2 mmol, 205 μL). Product was isolated as 182 mg (31%) of a white powder.

**1a**: ^1^H NMR (500 MHz, acetone-d6): δ 7.29–7.16 (m, 10H), 6.80 (d, *J* = 1.8 Hz, 1H), 4.41 (dd, *J* = 6.8, 1.6 Hz, 1H), 3.53 (ddd, *J* = 9.3, 6.8, 4.6 Hz, 1H), 2.91 (dd, *J* = 16.4, 9.3 Hz, 1H), 2.66 (dd, *J* = 16.4, 4.6 Hz, 1H). ^13^C{^1^H} NMR (125 MHz, acetone-d6): δ 198.8, 167.7, 151.1, 143.2, 141.8, 133.3, 129.28, 129.25, 129.1 128.5, 127.7, 127.6, 49.3, 49.0, 42.5. IR (atr) cm^–1^: 2700–3200 (broad), 1362 (m) (R-COOH), 1721 (s) (C=C-ROOH), 1643 (s) (C=C-**CO**-R), 1452 (m) (R-CO-**CH**_**2**_-R), 1238 (s), 1216 (s) (R-**CO**-R), 762 (s), 695 (s) (5 adjacent H (Ph)). HRMS (ESI-TOF) *m*/*z*: [**1a**-H]^−^ calculated for C_19_H_15_O_3_ 291.1016; found 291.1010. See the X-ray diffraction section for solved crystal structure of **1a** (CCDC: 2193907).

**1b**: ^1^H NMR (500 MHz, acetone-d6): δ 7.21–7.17 (m, 3H), 7.17–7.14 (m, 1H), 7.14–7.09 (m, 2H), 6.98 (s, 1H), 6.90 (m, 2H), 6.72 (m, 2H), 4.40 (d, *J* = 4.9 Hz, 1H), 3.95 (dt, *J* = 15.1, 4.4 Hz, 1H), 2.95 (dd, *J* = 17.0, 15.0 Hz, 1H), 2.42 (dd, *J* = 16.9, 3.7 Hz, 1H). ^13^C{^1^H} NMR (125 MHz, acetone-d6): δ 200.2, 167.1, 150.9, 141.9, 135.8, 134.0, 130.3, 128.82, 128.81, 128.7, 127.9, 127.7, 48.5, 45.0, 38.1. IR (atr) cm^–1^: 2800–3200 (broad), 1279 (s) (R-COOH), 1692 (s) (C=C-ROOH), 1672 (s) (C=C-**CO**-R), 1452 (m) (R-CO-**CH**_**2**_-R), 1253 (s) (R-**CO**-R), 772 (s), 696 (s) (5 adjacent H (Ph)). HRMS (ESI-TOF) *m*/*z*: [**1b**-H]^−^ calculated for C_19_H_15_O_3_ 291.1016; found 291.1016.

#### 1-Hydroxy-5-oxo-2,3-diphenylcyclohexane-1-carboxylic acid (**1A**) (Enantiomeric Pair of RRR and SSS)

The product was synthesized from 4-phenyl-3-buten-2-one (2 mmol, 292.4 mg), phenylpyruvic acid (2 mmol, 328.3 mg), water (3 mL), and NaOH (3 mmol). Reactants and solvents were placed in a 20 mL vial. The vial was stirred (600 rpm) at room temperature for 4 h. After reaction, 1 M HCl was added to the vial until the precipitation formation stopped. Product was filtered, washed with water, and dried under vacuum. Product was isolated as 596 mg (96%) of white powder (500 MHz, DMSO-d6): 7.24–7.20 (m, 2H), 7.20–7.16 (m, 2H), 7.11–7.07 (m, 2H), 7.05–7.01 (m, 2H), 7.00–6.95 (m, 2H), 5.35 (s (broad, 1H, OH proton), 3.83 (d, *J* = 12.2 Hz, 1H), 3.74 (td, *J* = 12.3, 4.4 Hz, 1H), 3.30 (d, *J* = 13.6 Hz, 1H), 2.95 (dd, *J* = 14.1, 12.5 Hz, 1H), 2.44 (dd, *J* = 13.7, 2.3 Hz, 1H), 2.37 (ddd, *J* = 14.3, 4.4, 2.3 Hz, 1H). ^13^C{^1^H} NMR (125 MHz, DMSO-d6): δ 206.8, 174.3, 143.1, 138.3, 129.8, 128.0, 127.8, 127.16, 126.17, 126.0, 78.56, 52.6, 51.1, 49.1, 44.1. IR (atr) cm^–1^: 3515 (m, sharp), 1075 (s) (Axial −OH), 2800–3200 (broad), 1721 (s), 932 (m) (R-COOH), 1680 (s), 1216 (s) (R-**CO**-R), 1454 (m) (R-CH_2_–R), 768 (s), 702 (s) (5 adjacent H (Ph)). HRMS (ESI-TOF) *m*/*z*: [**1A**-H]^−^ calculated for C_19_H_17_O_4_ 309.1121; found 309.1123.

#### 4-(para-Phenol)-5-phenyl-cyclohex-2-en-1-one-3-carboxylic Acid (**2**) (Mixture of Epimers RR/SS and RS/SR)

The product was synthesized according to the general procedure from presynthesized enone using 4-hydroxy phenylpyruvic acid (2 mmol, 360.3 mg) instead of phenylpyruvic acid. Purification: After the reaction, the remaining solvent in the microwave tube was evaporated. Next the crude powder was washed with hexane. The crude powder was then dissolved in water and filtered. The filtrate was protonated by adding HCl until the formation of a cloudy precipitate stops. The precipitate was difficult to filter; thus, it was dried under vacuum. The protonated powder was moved into a clean vial and dissolved with ethanol (ca. 3 mL). In the following weeks, each day a small amount of water (ca. 300 μL) was added each day to the vial. After the first week, mainly a brown-red powder was formed from the slow crystallization process. The brown-red powder was filtered out, and the crystallization process continues until colorless crystals are formed (see Figure S4 as reference picture). This crystallization must be done very slowly in order to obtain good high purity crystals. The crystals are filtered, washed with water, and dried under vacuum. Colorless crystals in an epimeric mixture were isolated with a 60 mg (10%) yield. Epimers RR/SS and RS/SR form in a 69% and 31% ratio respectively. ^1^H NMR (500 MHz, acetone-d6): δ 8.23 (broad, 1H, phenolic OH.), 7.29–7.16 (m, 5H), 7.03–6.99 (m, 1.5H), 6.94–6.90 (m, 1H), 6.75–6.71 (m, 2H), 6.64–6.50 (m, 1.7H), 4.32 (m, 1H), 3.87 (dt, *J* = 15.0, 4.2 Hz, 0.36H), 3.48 (ddd, *J* = 8.9, 6.5, 4.6 Hz, 0.81H), 2.94 (dd, *J* = 17.0, 14.9 Hz, 1.2H), 2.87 (dd, *J* = 16.4, 8.9 Hz, 1.4H), 2.67 (dd, *J* = 16.5, 4.7 Hz, 1H). 2.39 (dd, *J* = 16.9, 3.7 Hz, 0.38H). ^13^C{^1^H} NMR (125 MHz, acetone-d6): δ 200.4, 199.0, 167.8, 167.2, 157.2, 151.6, 151.1, 143.5, 142.2, 133.5, 132.8, 132.2, 131.4, 130.1, 129.3, 128.9, 128.8, 128.5, 127.6, 127.5, 126.0, 116.1, 115.6, 49.1, 48.5, 47.7, 45.1, 42.3, 38.2. IR (atr) cm^–1^: 2700–3200 (broad) (R-COOH), 3200–3500 (broad) (intermolecular hydrogen bonds), 1663 (s) (C=C-**CO**-R), 1610 (m) (R_2_-C=**CH**-R), 1446 (m) (R-**CH**_**2**_-R), 1361 (w), 1212 (s) (**OH**-Ph), 831 (s) (2 adjacent H (Ph)), 760 (s), 697 (s) (5 adjacent H (Ph)). HRMS (ESI-TOF) *m*/*z*: [**2**-H]^−^ calculated for C_19_H_15_O_4_ 307.0965; found 307.0962.

#### 4-Phenyl-5-(4-methylphenyl)-cyclohex-2-en-1-one-3-carboxylic acid (**3**) (Enantiomeric Pair of RR and SS)

The product was synthesized according to the general procedure for *in situ* synthesized enone using *p*-tolualdehyde (2 mmol, 235 μL) instead of benzaldehyde. Product was isolated as 121 mg (20%) of a white powder. ^1^H NMR (500 MHz, acetone-d6): δ 7.28–7.24 (m, 2H), 7.22–7.17 (m, 3H), 7.13–7.06 (m, 4H), 6.79 (d, *J* = 1.8 Hz, 1H), 4.39 (dd, *J* = 6.6, 1.6 Hz, 1H), 3.49 (ddd, *J* = 9.0, 6.6, 4.7 Hz, 1H), 2.87 (dd, *J* = 16.4, 9.1 Hz, 1H), 2.64 (dd, *J* = 16.4, 4.6 Hz, 1H), 2.26 (s, 3H). ^13^C{^1^H} NMR (125 MHz, acetone-d6): δ 198.9, 167.7, 151.0, 141.8, 140.2, 137.0, 133.4, 129.9, 129.3, 129.1, 128.3, 127.7, 49.3, 48.6, 42.5, 21.0. IR (atr) cm^–1^: 2800–3200 (broad) (R-COOH), 1680 (s) (C=C-**CO**-R), 1419 (m) (R-CO-**CH**_**2**_-R), 1255 (s) (R-**CO**-R), 1146 (m) (R-**CO**OH), 1058 (m) (CH_3_–Ph), 816 (s) (2 adjacent H (R-Ph-*p*-Me)), 703 (s) (5 adjacent H (Ph)). HRMS (ESI-TOF) *m*/*z*: [**3**-H]^−^ calculated for C_20_H_17_O_3_ 305.1172; found 305.1170.

#### 4-Phenyl-5-(3-methylphenyl)-cyclohex-2-en-1-one-3-carboxylic acid (**4**) (Enantiomeric Pair of RR and SS)

The product was synthesized according to the general procedure for *in situ* synthesized enone using *m*-tolualdehyde (2 mmol, 235 μL) instead of benzaldehyde. Product was isolated as 172 mg (28%) of a white powder. ^1^H NMR (500 MHz, acetone-d6): δ 7.29–7.23 (m, 2H), 7.22–7.17 (m, 3H), 7.16–7.12 (m, 1H), 7.07 (s, 1H), 7.07–6.98 (m, 2H), 6.80 (d, *J* = 1.8 Hz, 1H), 4.40 (dd, *J* = 6.6, 1.7 Hz, 1H), 3.48 (ddd, *J* = 9.0, 6.6, 4.7 Hz, 1H), 2.88 (dd, *J* = 16.4, 9.0 Hz, 1H), 2.65 (dd, *J* = 16.5, 4.6 Hz, 1H), 2.26 (s, 3H). ^13^C{^1^H} NMR (125 MHz, acetone-d6): δ 198.9, 167.7, 150.9, 143.1, 141.8, 138.7, 133.4, 129.24, 129.23, 129.16, 129.0, 128.3, 127.7, 125.4, 49.2, 48.9, 42.4, 21.4. IR (atr) cm^–1^: 2800–3200 (broad) (R-COOH), 1711 (s) (C=C-ROOH), 1651 (s) (C=C-**CO**-R), 1454 (m), 1058 (m) (Ph–CH_3_), 1415 (m) (R-CO-**CH**_**2**_-R), 1228 (s) (R-**CO**-R), 880 (m) (isolated H (Ph)), 780 (m) (3 adjacent H (Ph)), 699 (s) (5 adjacent H (Ph)). HRMS (ESI-TOF) *m*/*z*: [**4**-H]^−^ calculated for C_20_H_17_O_3_ 305.1172; found 305.1173.

#### 4-Phenyl-5-(2-methylphenyl)-cyclohex-2-en-1-one-3-carboxylic acid (**5**) (Enantiomeric Pair of RR and SS)

The product was synthesized according to the general procedure for *in situ* synthesized enone by using *o*-tolualdehyde (2 mmol, 230 μL) instead of benzaldehyde. Product was isolated as 224 mg (37%) of a white powder. ^1^H NMR (500 MHz, acetone-d6): δ 7.53 (d, *J* = 7.8 Hz, 1H), 7.25–7.13 (m, 4H), 7.10–7.05 (m, 3H), 7.01 (d, *J* = 7.5 Hz, 1H), 6.80 (dd, *J* = 2.2, 0.6 Hz, 1H), 4.32 (dd, *J* = 8.2, 2.2 Hz, 1H), 3.76 (ddd, *J* = 11.2, 8.2, 4.5 Hz, 1H), 2.90 (dd, *J* = 16.3, 11.2 Hz, 1H), 2.57 (dd, *J* = 16.2, 4.4 Hz, 1H), 1.92 (s, 3H). ^13^C{^1^H} NMR (125 MHz, acetone-d6): δ 198.9, 167.9, 152.1, 142.2, 141.4, 136.9, 133.0, 131.2, 129.1, 129.0, 127.6, 127.5, 127.4, 127.1, 49.5, 44.2, 43.3, 19.5. IR (atr) cm^–1^: 2800–3200 (broad) (R-COOH), 1704 (m) (C=C-ROOH), 1669 (s) (C=C-**CO**-R), 1454 (w), 1052 (m) (Ph–CH_3_), 1412 (w) (R-CO-**CH**_**2**_-R), 1254 (s) (R-**CO**-R), 1152 (m) (R-**CO**OH), 762 (s) (4 adjacent H (Ph)), 701 (s), 726 (s) (5 adjacent H (Ph)). HRMS (ESI-TOF) *m*/*z*: [**5**-H]^−^ calculated for C_20_H_17_O_3_ 305.1172; found 305.1174.

#### 4-Phenyl-5-(4-methoxyphenyl)-cyclohex-2-en-1-one-3-carboxylic acid (**6**) (Enantiomeric Pair of RR and SS)

The product was synthesized according to the general procedure for *in situ* synthesized enone using 4-methoxybenzaldehyde (2 mmol, 245 μL) instead of benzaldehyde. Product was isolated as 175 mg (27%) of a white powder. ^1^H NMR (500 MHz, acetone-d6): δ 7.28–7.23 (m, 2H), 7.21–7.12 (m, 5H), 6.84–6.80 (m, 2H), 6.78 (d, *J* = 1.8 Hz, 1H), 4.36 (dd, *J* = 6.7, 1.6 Hz, 1H), 3.74 (s, 3H), 3.46 (ddd, *J* = 9.2, 6.7, 4.6 Hz, 1H), 2.86 (dd, *J* = 16.4, 9.2 Hz, 1H), 2.63 (dd, *J* = 16.4, 4.5 Hz, 1H). ^13^C{^1^H} NMR (125 MHz, acetone-d6): δ 199.1, 167.8, 159.4, 151.2, 141.9, 135.0, 133.3, 129.4, 129.2, 129.0, 127.6, 114.6, 55.4, 49.5, 48.3, 42.7. IR (atr) cm^–1^: 2800–3200 (broad), 1182 (s), 1146 (s) (R-COOH), 1679 (s) (C=C-**CO**-R), 1610 (m) (R_2_-C=**CH**-R), 1238 (s) (R-**CO**-R), 1030 (m) (R-O–CH_3_), 824 (s) (2 adjacent H (Ph)), 773 (s), 703 (s) (5 adjacent H (Ph)), 755 (m) (Ph-O–CH_3_). HRMS (ESI-TOF) *m*/*z*: [**6**-H]^−^ calculated for C_20_H_17_O_4_ 321.1121; found 321.1120.

#### 4-Phenyl-5-(3-methoxyphenyl)-cyclohex-2-en-1-one-3-carboxylic acid (**7**) (Enantiomeric Pair of RR and SS)

The product was synthesized according to the general procedure for *in situ* synthesized enone using 3-methoxybenzaldehyde (2 mmol, 245 μL) instead of benzaldehyde. Product was isolated as 374 mg (58% yield) of a white powder. ^1^H NMR (500 MHz, acetone-d6): δ 7.28–7.23 (m, 2H), 7.22–7.15 (m, 4H), 6.81–6.78 (m, 3H), 6.77–6.74 (m, 1H), 4.41 (dd, *J* = 6.8, 1.5 Hz, 1H), 3.73 (s, 3H), 3.50 (ddd, *J* = 9.4, 6.8, 4.6 Hz, 1H) 2.91 (dd, *J* = 16.4, 9.4 Hz, 1H), 2.65 (dd, *J* = 16.4, 4.5 Hz, 1H). ^13^C{^1^H} NMR (125 MHz, acetone-d6): δ 198.9, 167.8, 160.7, 151.2, 144.7, 141.8, 133.2, 130.3, 129.2, 129.1, 127.7, 120.6, 114.4, 112.9, 55.4, 49.2, 49.1, 42.6. IR (atr) cm^–1^: 2800–3200 (broad) (R-COOH), 1720 (s) (C=C-ROOH), 1644 (s) (C=C-**CO**-R), 1454 (m) (R-O–CH_3_), 1439 (m) (R-CO-**CH**_**2**_-R), 1234 (s) (R-**CO**-R), 1051 (m), 754 (s) (Ph-O–CH_3_), 903 (m) (isolated H (Ph)), 771 (s), 706 (s) (3 adjacent H (Ph)), 695 (s) (5 adjacent H (Ph)). HRMS (ESI-TOF): *m*/*z*: [**7**-H]^−^ calculated for C_20_H_17_O_4_ 321.1121; found 321.1118.

#### 4-Phenyl-5-(2-methoxyphenyl)-cyclohex-2-en-1-one-3-carboxylic acid (**8**) (Enantiomeric Pair of RR and SS)

The product was synthesized according to the general procedure for *in situ* synthesized enone using 2-methoxybenzaldehyde (2 mmol, 240 μL) instead of benzaldehyde. Product was isolated as 111 mg (17%) of a white powder. ^1^H NMR (500 MHz, acetone-d6): δ 7.33–7.26 (m, 4H), 7.25–7.18 (m, 2H), 7.01 (m, 2H), 6.88 (m, 2H), 4.52 (d, *J* = 4.8 Hz, 1H), 3.89 (s, 3H), 3.83 (q, *J* = 5.0 Hz, 1H), 2.78 (dd, *J* = 17.0, 6.4 Hz, 1H), 2.65 (dd, *J* = 17.0, 5.3 Hz, 1H). ^13^C{^1^H} NMR (125 MHz, acetone-d6): δ 199.6, 167.7, 158.1, 150.2, 142.1, 133.6, 131.5, 129.3, 129.0, 128.8, 128.5, 127.7, 121.3, 111.9, 55.8, 46.9, 43.1, 39.7. IR (atr) cm^–1^: 2800–3200 (broad) (R-COOH), 1716 (s) (C=C-ROOH), 1643 (s) (C=C-**CO**-R), 1463 (m), 1029 (m) (Ph-O–CH_3_), 1438 (w) (R-CO-**CH**_**2**_-R), 1221 (s) (R-**CO**-R), 1165 (m), 1115 (m) (R-COOH), 753 (s) (4 adjacent H (Ph)), 697 (s) (5 adjacent H (Ph)). HRMS (ESI-TOF): *m*/*z*: [**8**-H]^−^ calculated for C_20_H_17_O_4_ 321.1121; found 321.1119.

#### 4-Phenyl-5-(4-fluorophenyl)-cyclohex-2-en-1-one-3-carboxylic acid (**9**) (Enantiomeric Pair of RR and SS)

The product was synthesized according to the general procedure for *in situ* synthesized enone using 4-fluorobenzaldehyde (2 mmol, 215 μL) instead of benzaldehyde. Product was isolated as 61 mg (10%) of a white powder. ^1^H NMR (500 MHz, acetone-d6): δ 7.28–7.22 (m, 4H), 7.21–7.17 (m, 1H), 7.16–7.12 (m, 2H), 7.04–6.99 (m, 2H), 6.78 (d, *J* = 2.0 Hz, 1H), 4.36 (dd, *J* = 7.4, 1.9 Hz, 1H), 3.53 (ddd, *J* = 10.1, 7.4, 4.4 Hz, 1H), 2.93 (dd, *J* = 16.3, 10.1 Hz, 1H), 2.65 (dd, *J* = 16.3, 4.4 Hz, 1H). ^13^C{^1^H} NMR (125 MHz, acetone-d6): δ 198.7, 167.7, 162.5 (d, J_C–F_ = 243.6 Hz), 151.4, 141.7, 139.1 (d, J_C–F_ = 3.2 Hz), 133,1, 130.3 (d, J_C–F_ = 7.8 Hz), 129.2, 129.1, 127.7, 115.9 (d, J_C–F_ = 21.1 Hz), 49.6, 48.5, 42.9. ^19^F NMR (470 MHz, acetone-d6): δ −117.63. IR (atr) cm^–1^: 2800–3200 (broad) (R-COOH), 1732 (m) (C=C-ROOH), 1671 (s) (C=C-**CO**-R), 1454 (m) (R-CO-**CH**_**2**_-R), 1268 (m), 533 (s) (Ph-F), 1217(s) (R-**CO**-R), 1154 (s), 1079 (m) (R-COOH), 823 (s), 780 (s) (2 adjacent H (R-Ph-*p*-F)), 757 (s) (C–F), 701 (s) (5 adjacent H (Ph)). HRMS (ESI-TOF): *m*/*z*: [**9**-H]^−^ calculated for C_19_H_14_O_3_F 309.0921; found 309.0919.

#### 4-Phenyl-5-(4-chlorophenyl)-cyclohex-2-en-1-one-3-carboxylic acid (**10**) (Enantiomeric Pair of RR and SS)

The product was synthesized according to the general procedure for *in situ* synthesized enone using 4-clorobenzaldehyde (2 mmol, 281.1 mg) instead of benzaldehyde. Product was isolated as 131 mg (20%) of a white powder. ^1^H NMR (500 MHz, acetone-d6): δ 7.30–7.14 (m, 9H), 6.77 (d, *J* = 2.0 Hz, 1H), 4.36 (dd, *J* = 7.4, 2.0 Hz, 1H), 3.55 (ddd, *J* = 10.1, 7.5, 4.4 Hz, 1H), 2.93 (dd, *J* = 16.3, 10.1 Hz, 1H) 2.65 (dd, *J* = 16.3, 4.4 Hz, 1H). ^13^C{^1^H} NMR (125 MHz, acetone-d6): δ 198.5, 167.7, 151.4, 142.0, 141.6, 133.1, 132.9, 130.3, 129.3, 129.1, 127.8, 49.4, 48.6, 42.8. IR (atr) cm^–1^: 2800–3200 (broad), 1149 (m) (R-COOH), 1732 (s) (C=C-ROOH), 1676 (s) (C=C-**CO**-R), 1427 (m) (R-CO-**CH**_**2**_-R), 1255(s) (R-**CO**-R), 1090 (m), 533 (s) (Ph–Cl), 821 (s), 780 (s) (2 adjacent H (R-Ph-*p*-Cl)), 703 (s) (5 adjacent H (Ph)). HRMS (ESI-TOF): *m*/*z*: [**10**-H]^−^ calculated for C_19_H_14_O_3_Cl 325.0626; found 325.0625.

#### 4-Phenyl-5-(4-(trifluoromethyl)phenyl)-cyclohex-2-en-1-one-3-carboxylic acid (**11**) (Enantiomeric Pair of RR and SS)

The product was synthesized according to the general procedure for *in situ* synthesized enone using 4-(trifluoromethyl)benzaldehyde (2 mmol, 275 μL) instead of benzaldehyde. Product was isolated as 28 mg (4%) of a white powder. ^1^H NMR (500 MHz, acetone-d6): δ 7.61 (d, *J* = 8.1 Hz, 2H), 7.47 (d, *J* = 8.1 Hz, 2H), 7.27–7.16 (m, 5H), 6.79 (d, *J* = 2.0 Hz, 1H), 4.44 (dd, *J* = 7.5, 1.9 Hz, 1H), 3.67 (ddd, *J* = 10.3, 7.5, 4.4 Hz, 1H), 2.99 (dd, 16.4, 10.1 Hz, 1H), 2.69 (dd, *J* = 16.3, 4.4 Hz, 1H). ^13^C{^1^H} NMR (125 MHz, acetone-d6): δ 198.3, 167.6, 151.3, 147.7 (d, J_C–F_ = 1.4 Hz), 141.4, 133.2, 129.4, 129.3, 129.1, 127.9, 126.4, 126.1 (q, J_C–F_ = 3.8 Hz), 124.3, 49.1, 49.0, 42.6. ^19^F NMR (470 MHz, acetone-d6): δ −62.93. IR (atr) cm^–1^: 2800–3200 (broad), 1111 (s) (R-COOH), 1723 (s) (C=C-ROOH), 1645 (s) (C=C-**CO**-R), 1421 (m) (R-CO-**CH**_**2**_-R), 1330 (s), 1160 (s), 512 (s) (R-CF_3_), 1240 (s) (R-**CO**-R), 836 (s) (2 adjacent H (R-Ph-*p*-CF_3_)), 703 (s) (5 adjacent H (Ph)) HRMS (ESI-TOF): *m*/*z*: [**11**-H]^−^ calculated for C_20_H_14_O_3_F_3_ 359.0890; found 359.0893.

#### 4-Phenyl-5-(4-(benzyloxy)phenyl)-cyclohex-2-en-1-one-3-carboxylic acid (**12a** and **12b**) (Enantiomeric Pair of RR/SS, **12a**, and RS/SR, **12b**)

Synthesis and separation of: **12a** and **12b**. The product was synthesized according to the general procedure for *in situ* synthesized enone using 4-(benzyloxy)benzaldehyde (2 mmol, 424.5 mg) instead of benzaldehyde. The products are obtained as a mixture of **12a** and **12b** (337 mg, 42%) as a white powder. According to the ^1^H NMR measurement, 62% of this mixture is **12a** (RR/SS) and the remaining 38% is **12b** (RS/SR).

##### Separation of **12a** and **12b**

Prior protonation of the product. The salt crystals formed of **12a** and **12b** are collected and washed with acetone and dried overnight under air. Then the crystals are heated with water to 90 °C for 15 min and cooled back down to room temperate. During the cooling crystals are forming and these are primarily the salts of **12b** crystals. These **12b** crystals are purified by dissolving them again with hot water (90 °C) and recrystallization to form pure **12b** salt crystals. The water solutions from the filtrations contain mainly **12a** crystals. The corresponding crystals are collected, dissolved into H_2_O (hot one for **12b**), and protonated with HCl, followed by filtration, washing with water, and drying under vacuum. Utilization of this separation method provided us pure **12a** and **12b** as white powders.

Synthesis of **12a** and **12b** was also done in water. The general procedure for cyclohexenone acid synthesis was utilized in water using phenylpyruvic acid (2 mmol, 328.3 mg) and *in situ* synthesized enone from acetone (2 mmol, 150 μL) and 4-(benzyloxy)benzaldehyde (2 mmol, 424.5 mg). Product was obtained as a mixture of **12a** and **12b** as 661 mg (83%) of a white powder. According to ^1^H NMR measurement, the ratio of **12a** (RR/SS, anti):**12b** (RS/SR, syn) was 62:38.

**12a**: ^1^H NMR: (500 MHz, acetone-d6): δ 7.47 (m, 2H), 7.39 (m, 2H), 7.32 (m, 1H), 7.26 (m, 2H), 7.22–7.11 (m, 5H), 6.91 (m, 2H), 6.79 (d, *J* = 1.9 Hz, 1H), 5.07 (s, 2H), 4.37 (dd, *J* = 6.7, 2.0 Hz, 1H), 3.47 (ddd, *J* = 9.3, 6.7, 4.5 Hz, 1H), 2.87 (dd, *J* = 16.4, 9.2 Hz, 1H), 2.64 (dd, *J* = 16.4, 4.5 Hz, 1H). ^13^C{^1^H} NMR (125 MHz, acetone-d6): δ 199.0, 167.8, 158.6, 151.1, 141.9, 138.4, 135.4, 133.3, 129.5, 129.3, 129.1, 128.6, 128.5, 127.7, 115.6, 70.4, 49.5, 48.3, 42.7. Note: One carbon signal is missing, probably due to overlapping at the aromatic region. IR (atr) cm^–1^: 2800–3200 (broad) (R-COOH), 1723 (s) (C=C-ROOH), 1460 (m) (R-CH_2_–R), 1246 (s) (R-**CO**-R), 1025 (m) (Ph-O-R), 830 (m) (2 adjacent H (R-Ph-R)), 690 (s) (5 adjacent H (Ph)). HRMS (ESI-TOF): *m*/*z*: [**12a**-H]^−^ calculated for C_26_H_21_O_4_ 397.1434; found 397.1430.

**12b**: ^1^H NMR (500 MHz, acetone-d6): δ 7.49–7.43 (m, 2H), 7.42–7.36 (m, 2H), 7.35–7.29 (m, 1H), 7.19–7.09 (m, 3H), 6.96 (s, 1H), 6.87–6.78 (m, 4H), 6.82 (d, *J* = 6.8 Hz, 2H), 5.08 (s, 2H), 4.36 (d, *J* = 4.9 Hz, 1H), 3.88 (dt, *J* = 15.2, 4.1 Hz, 1H), 2.88 (dd, *J* = 16.9, 15.1 Hz, 1H), 2.38 (dd, *J* = 16.8, 3.6 Hz, 1H). ^13^C{^1^H} NMR (125 MHz, acetone-d6): δ 200.3, 167.1, 158.7, 151.0, 138.5, 135.9, 134.2, 134.0, 130.4, 129.8, 129.3, 128.7, 128.6, 128.4, 127.9, 115.2, 70.4, 48.6, 44.4, 38.5. IR (atr) cm^–1^: 2800–3200 (broad) (R-COOH), 1692 (s), 1613 (m) (C=C-ROOH), 1676 (s), 1254 (s) (R-**CO**-R), 1493 (w), 1453 (m), 1030 (m) (R-O-**CH**_2_-R), 1435 (m) (R-**CH**_2_-R), 828 (m) (2 adjacent H (R-Ph-R)), 700 (s) (5 adjacent H (Ph)). HRMS (ESI-TOF): *m*/*z*: [**12b**-H]^−^ calculated for C_26_H_21_O_4_ 397.1434; found 397.1435.

#### 4,5-Diphenyl-2-methyl-cyclohex-2-en-1-one-3-carboxylic acid (**13a** and **13b**) (Enantiomeric pair of RR/SS (anti) and RS/SR (syn))

The synthesis of the mixture of anti and syn products was carried out according to the general procedure for cyclohexenone acid synthesis in water using phenylpyruvic acid (2 mmol, 328.3 mg) and *in situ* synthesized enone from 2-butanone (2 mmol, 180 μL) and benzaldehyde (2 mmol, 205 μL). Product was obtained as a mixture of **13a** and **13b** as 600 mg (98%) of a white powder. According to ^1^H NMR measurement, the ratio of **13a** (RR/SS, anti):**13b** (RS/SR, syn) was 72:28.

##### Enrichment of **13b**

The white powder mixture (600 mg) of **13a** and **13b** (72:28 ratio) was dissolved in ethanol (ca. 2 mL). Water (ca. 6 mL) was added into the solution. After a week crystals form in the solution, and the crystals were filtered and washed with water. This set of crystals (180 mg) was identified as **13a**. The next set of crystals (300 mg) precipitated from the solution after another week. The second set of crystals were identified as a mixture of **13a** and **13b** (1:1 ratio). This enriched **13b** powder mixture was dissolved into chloroform (ca. 2 mL), and hexane (ca. 6 mL) was added as an anti solvent. The following day, precipitation formed from the chloroform/hexane solution, which was identified as pure **13a**, and the precipitate was put aside. The remaining solution was evaporated in vacuum, and the formed crude solid was collected by filtration and washed with hexane. The crude solid had a slightly higher **13b** ratio than earlier. The slightly more enriched **13b** is subjected to the same chloroform (solvent)/hexane (anti solvent) recrystallization treatment repeatedly until 85% enrichment was achieved.

Synthesis of **13a** was carried out by the general procedure for *in situ* synthesized enone using 2-butanone (2 mmol, 180 μL) instead of acetone. Product was isolated as 233 mg (38%) of a white powder.

**13a**: ^1^H NMR (500 MHz, acetone-d6): δ 7.22–7.09 (m, 10H), 4.32 (dq, *J* = 9.3, 2.3 Hz, 1H), 3.48 (ddd, *J* = 12.4, 9.3, 4.0 Hz, 1H), 3.00 (dd, *J* = 15.9, 12.5 Hz, 1H), 2.64 (dd, *J* = 15.9, 4.0 Hz, 1H), 1.98 (d, *J* = 2.3 Hz, 3H). ^13^C{^1^H} NMR (125 MHz, acetone-d6): δ 198.5, 168.9, 148.2, 142.9, 141.4, 135.4, 129.9, 129.2, 128.9, 128.5, 127.7, 127.6, 51.7, 49.2, 44.3, 12.9. IR (atr) cm^–1^: 2800–3200 (broad), 1347 (m), 1122 (m), 926 (m) (R-COOH), 1722 (s) (C=C-ROOH), 1638 (s) (C=C-**CO**-R), 1448 (m) (R-CH_3_), 1219 (s) (R-**CO**-R), 1034(w), 755 (s), 699 (s) (5 adjacent H (Ph)) HRMS (ESI-TOF): *m*/*z*: [**13a**-H]^−^ calculated for C_20_H_17_O_3_ 305.1172; found 305.1170.

**13b**: ^1^H NMR (500 MHz, acetone-d6): δ 7.21–7.17 (m, 3H), 7.15–7.12 (m, 1H), 7.11–7.07 (m, 2H), 6.90–6.86 (m, 2H), 6.74–6.69 (m, 2H), 4.39 (d, *J* = 5.1 Hz, 1H), 3.93 (ddd, *J* = 15.1, 5.0, 3.6 Hz, 1H), 2.96 (dd, *J* = 16.7, 15.1 Hz, 1H), 2.44 (dd, *J* = 16.7, 3.6 Hz, 1H), 2.12 (d, *J* = 1.3 Hz, 3H). ^13^C{^1^H} NMR (125 MHz, acetone-d6): δ 199.8, 169.1, 146.9, 142.0, 137.9, 135.8, 130.7, 128.80, 128.79, 128.5, 127.9, 127.6, 50.6, 44.6, 37.8, 13.2. IR (atr) cm^–1^: 2800–3200 (broad) (R-COOH), 1695 (s) (C=C-ROOH), 1675 (s) (C=C-**CO**-R), 1452 (w) (R-CH_3_), 1245 (s) (R-**CO**-R), 763 (s), 696 (s) (5 adjacent H (Ph)) HRMS (ESI-TOF) *m*/*z*: [**13b**-H]^−^ calculated for C_20_H_17_O_3_ 305.1172; found 305.1173.

#### 4,5-Diphenyl-2-ethyl-cyclohex-2-en-1-one-3-carboxylic acid (**14a** and **14b**) (Enantiomeric Pair of RR/SS (anti) and RS/SR (syn))

The synthesis of the mixture of anti and syn products was carried out according to the general procedure for cyclohexenone acid synthesis in water using phenylpyruvic acid (2 mmol, 328.3 mg) and *in situ* synthesized enone from 2-pentanone (2 mmol, 215 μL) and benzaldehyde (2 mmol, 205 μL). Product was obtained as a mixture of **14a** and **14b** as 628 mg (98%) of a white powder. According to ^1^H NMR measurement, the ratio of **14a** (RR/SS, anti):**14b** (RS/SR, syn) was 75:25.

##### Separation of **14a** and **14b**

The mixture of **14a** and **14b** was dissolved into acetone (ca. 4 mL), and water was added into it (ca. 4 mL). After 2 days the formed crystals were collected, and 180 mg of **14a** were obtained as a white powder.

##### Enrichment of **14b**

The enrichment process is the same as that for **13b**, except acetone was used instead of EtOH at the initial crystallization. We managed to enrich the product to a 55% ratio.

**14a**: ^1^H NMR (500 MHz, acetone-d6): δ 7.23–7.19 (m, 4H), 7.19–7.09 (m, 6H), 4.32 (d, *J* = 9.2 Hz, 1H), 3.48 (ddd, *J* = 12.2, 9.3, 4.0 Hz,1H), 2.98 (dd, *J* = 15.9, 12.3 Hz), 2.64 (dd, *J* = 15.9, 4.0 Hz, 1H), 2.56 (m, 1H), 2.43 (dq, *J* = 12.5, 7.5 Hz, 1H), 1.08 (t, *J* = 7.4 Hz, 3H). ^13^C{^1^H} NMR (125 MHz, acetone-d6): δ 198.1, 168.9, 148.1, 142.9, 141.2, 140.9, 129.9, 129.1, 128.9, 128.5, 127.7, 127.5, 51.6, 49.1, 44.6, 21.1, 14.6. IR (atr) cm^–1^: 2800–3200 (broad), 1174 (s), 918 (w) (R-COOH), 1725 (C=C-ROOH), 1641 (s) (C=C-**CO**-R), 1454 (m) (R-CO-**CH**_**2**_-R), 1216 (s) (R-**CO**-R), 762 (s), 697 (s) (5 adjacent H (Ph)). HRMS (ESI-TOF) *m*/*z*: [**14a**-H]^−^ calculated for C_21_H_19_O_3_ 319.1329; found 319.1331.

**14b**: ^1^H NMR (500 MHz, acetone-d6): δ 7.22–7.07 (m, 6H), 6.91–6.85 (m, 2H), 6.74–6.69 (m, 2H), 4.36 (d, *J* = 5.0 Hz, 1H), 3.92 (ddd, *J* = 15.1, 5.0, 3.6 Hz, 1H), 2.96 (m, 1H), 2.69 (m, 1H), 2.56 (m, 1H), 2.43 (m, 1H), 1.14 (t, *J* = 7.4 Hz, 3H). ^13^C{^1^H} NMR (125 MHz, acetone-d6): δ 199.4, 169.2, 146.9, 142.9, 141.9, 135.6, 130.7, 128.8, 128.8, 128.5, 127.9, 127.6, 50.5, 44.8, 38.1, 21.2, 14.5. Note: Aromatic area has heavy overlap with **14a**; thus, proton integrals are not accurate. Carbon signal 146.9 is not observed in regular ^13^C{^1^H} NMR, but it is observed in the HMBC spectrum. IR (atr) cm^–1^: 2800–3200 (broad), 1275 (m) (R-COOH), 1674 (s) (C=C-**CO**-R), 1453 (m) (R-CH_3_), 1417 (w) (R-CO-**CH**_**2**_-R), 1241 (s) (R-**CO**-R), 764 (s), 697 (s) (5 adjacent H (Ph)). HRMS (ESI-TOF) *m*/*z*: [**14b**-H]^−^ calculated for C_21_H_19_O_3_ 319.1329; found 319.1329.

#### 2-Ethyl-1-hydroxy-3-oxo-5,6-diphenylcyclohexane-1-carboxylic acid (**14A**) (Enantiomeric Pair 1S, 2S, 5R, 6R and 1R, 2R, 5S, 6S)

The product was synthesized according to the general procedure for *in situ* synthesized enone using 2-pentanone (2 mmol, 215 μL) instead of acetone. Product was isolated as 88 mg (13%) of a white powder. ^1^H NMR (500 MHz, acetone-d6): δ 7.32–7.16 (m, 4H), 7.12 (t, *J* = 7.7 Hz, 2H), 7.06–6.94 (m, 4H), 4.01 (d, *J* = 12.2 Hz, 1H), 3.80 (td, *J* = 12.6, 4.4 Hz, 1H), 3.16 (dd, *J* = 9.3, 1.5 Hz, 1H), 3.03 (t, *J* = 13.2 Hz, 1H), 2.53 (dd, *J* = 13.5, 4.4 Hz, 1H), 2.04 (m, 1H), 1.04 (m, 1H), 0.94 (t, *J* = 7.3 Hz, 3H). ^13^C{^1^H} NMR (125 MHz, acetone-d6): δ 206.6, 174.6, 143.9, 138.9, 129.0, 128.7, 128.4, 127.5, 127.0, 83.8, 58.8, 55.8, 50.8, 46.3, 18.5, 13.5. IR (atr) cm^–1^: 3467 (m, sharp) (Axial −OH), 2800–3200 (broad), 1724 (s), 1123 (R-COOH) 1682 (s), 1224 (s) (R-**CO**-R) 1454 (m) (R-CH_2_–R) 1373(m), 926 (m), (R-CH_3_), 1092 (s) (R–OH), 696 (s) (5 adjacent H (Ph)). HRMS (ESI-TOF): *m*/*z*: [**14A**-H]^−^ calculated for C_21_H_21_O_4_ 337.1434; found 337.1438. See the X-ray diffraction section for the solved crystal structure of **14A** (CCDC: 2193908). Notes: proton 2.04 overlapped by acetone-d6 signal. One aromatic carbon missing at the carbon spectrum. 128.74 is wider than usual; thus, it might be a double peak. Compound has sharp axial −OH signal in IR spectrum. Similar signals can be observed in **15b**.

#### 1-Hydroxy-2,2-dimethyl-3-oxo-5,6-diphenylcyclohexane-1-carboxylic acid (**15a**) (Enantiomeric Pair RRR and SSS) and (**15b**) Enantiomeric Pair (1S, 5R, 6R and 1R, 5S, 6S)

The product was synthesized according to the general procedure for *in situ* synthesized enone using 3-methyl-2-butanone (2 mmol, 215 μL) instead of acetone. Isolation: When acetone (5 mL) is added into the microwave tube, the product **15a** salt is formed within a few hours in the tube. The salt is filtered and washed with acetone. The obtained solid is dissolved with water and protonated with HCl. **15a** was isolated as 103 mg (15%) of a white powder. The remaining filtrated reaction solution contains the corresponding **15b** salt. The **15b** salt precipitated out from the filtrated reaction and washing solution overnight. The salt was filtered and washed with acetone. The salt is dissolved with water and protonated with HCl and dried under vacuum. **15b** was isolated as 88 mg (13%) of a white powder.

##### Synthesis of **15a** and **15b** was also done in water

By utilizing the general procedure for cyclohexenone acid synthesis in water using phenylpyruvic acid (2 mmol, 328.3 mg) and *in situ* synthesized enone from 3-methyl-2-butanone (2 mmol, 215 μL) and benzaldehyde (2 mmol, 205 μL). Product was obtained as a mixture of **15a** and **15b** as 610 mg (90%) of a white powder. According to ^1^H NMR measurement, the ratio of **15a** (RRR/SSS):**15b** (1S, 5R, 6*R*/1R, 5S, 6S) was 67:33.

**15a**: ^1^H NMR (500 MHz, acetone-d6): δ 7.33–7.26 (m, 2H), 7.24–7.18 (m, 2H), 7.16–7.06 (m, 4H), 7.05–6.96 (m, 2H), 4.06 (td, *J* = 12.6, 5.2 Hz, 1H), 3.90 (d, *J* = 12.5 Hz, 1H), 3.02 (m, 1H), 2.53 (dd, *J* = 15.4, 5.4 Hz), 1.52 (s, 3H), 1.07 (s, 3H). ^13^C{^1^H} NMR (125 MHz, acetone-d6): δ 209.6, 175.1, 144.3, 138.8, 130.8, 129.2, 128.7, 128.4, 127.6, 127.1, 83.8, 53.1, 52.9, 46.1, 42.9, 23.1, 19.1. IR (atr) cm^–1^: 3633 (m, sharp), 1189 (s) (R–OH), 3250–3450 (m, broad) (intermolecular hydrogen bonds), 2800–3200 (broad), 1721 (s), 1102 (s) (R-COOH), 1703 (s), 1280 (s) (R-**CO**-R), 1454 (m), 1385 (m) (R-CH_3_), 751 (s), 695 (s) (5 adjacent H (Ph)). HRMS (ESI-TOF): *m*/*z*: [**15a**-H]^−^ calculated for C_21_H_21_O_4_ 337.1434; found 337.1430. See the X-ray diffraction section for the solved crystal structure of **15a** (CCDC: 2193909).

**15b**: ^1^H NMR (500 MHz, acetone-d6): δ 7.48–7.25 (m, 4H), 7.13 (t, *J* = 7.8 Hz, 2H), 7.07–6.94 (m, 4H), 4.19 (d, *J* = 12.3 Hz, 1H), 3.83 (td, *J* = 12.6, 5.3 Hz, 1H), 3.11 (dd, *J* = 14.9, 12.8 Hz, 1H), 2.46 (dd, *J* = 14.9, 5.3 Hz, 1H), 1.67 (s, 3H), 1.09 (s, 3H). ^13^C{^1^H} NMR (125 MHz, acetone-d6): δ 210.1, 173.7, 144.0, 139.4, 131.1, 129.1, 128.8, 128.2, 127.4, 127.0, 84.3, 53.1, 50.3, 46.1, 45.6, 24.3, 19.1. IR (atr) cm^–1^: 3539 (m, sharp) (Axial −OH), 2800–3200 (broad), 1725 (s) (R-COOH), 1677 (s), 1189 (s) (R-**CO**-R), 1456 (w), 1387 (w), 965 (m) (R-CH_3_), 1121 (m) (*tert*–OH), 750 (m), 698 (s) (5 adjacent H (Ph)). HRMS (ESI-TOF): *m*/*z*: [**15b**-H]^−^ calculated for C_21_H_21_O_4_ 337.1434; found 337.1431. Note: IR spectrum has a similar sharp axial −OH signal as **14**; this further suggests the axial nature of the alcohol group here.

#### 5-(4-(Benzyloxy(phenyl)-1-hydroxy-2,2-dimethyl-3-oxo-6-phenylcyclohexane-1-carboxylic acid (**16a**) (Enantiomeric Pair RRR and SSS) and (**16b**) Enantiomeric Pair (1S, 5R, 6R and 1R, 5S, 6S)

The product was synthesized according to the general procedure for *in situ* synthesized enone using 3-methyl-2-butanone (2 mmol, 215 μL) instead of acetone and 4-(benzyloxy)benzaldehyde (2 mmol, 424.5 mg) instead of benzaldehyde. Isolation: When acetone (5 mL) is added into the microwave tube to initiate the crystallization, the product **16a** salt is crystallized within a few hours. The salt is filtered and washed with acetone. The obtained solid is dissolved with water and protonated with HCl (1 M). **16a** was isolated as 104 mg (12%) of a white powder. In order to precipitate **16b** salt, acetone (ca. 20 mL) was added into the solution where the **16a** salt precipitated out. After waiting overnight, **16b** salt precipitated out from the remaining solution. It is then filtered and washed with acetone. Then the crystals are dissolved with water and protonated with HCl (1 M), washed with water, and dried under vacuum. **16b** is obtained as a beige powder (72 mg, 8%).

**16a**: ^1^H NMR (500 MHz, acetone-d6): δ 7.45–7.38 (m, 2H), 7.38–7.32 (m, 2H), 7.32–7.27 (m, 1H), 7.27–7.16 (m, 4H), 7.12–7.05 (m, 2H), 7.05–7.00 (m, 1H), 6.77 (d, *J* = 8.7 Hz, 2H), 4.95 (s, 2H), 4.04 (td, *J* = 12.3, 5.4 Hz, 1H), 3.80 (d, *J* = 12.8 Hz, 1H), 2.92 (dd, *J* = 15.1, 12.4 Hz, 1H), 2.50 (dd, *J* = 15.2, 5.5 Hz, 1H), 1.49 (s, 3H), 1.06 (s, 3H). ^13^C{^1^H} NMR (125 MHz, acetone-d6): δ 176.0, 158.1, 139.4, 138.5, 136.9, 130.9, 129.7, 129.2, 128.6, 128.5, 128.3, 127.3, 115.4, 83.5, 70.3, 53.4, 52.7, 46.1, 42.2, 22.8, 19.2. IR (atr) cm^–1^: 2800–3200 (broad), 1738 (s) (R-COOH), 1710 (s), 1249 (s) (R-**CO**-R), 1463 (m) (R-CH_2_–R), 1384 (m) (R-CH_3_), 1306 (m), 1075 (s), 1024 (w), (Ph-O-R), 1174 (m) ((CH_3_)_2_-C-R_2_), 900 (m), 735 (s), 688 (s) (5 adjacent H (Ph)), 830 (m) (2 adjacent H (R-Ph-R)). HRMS (ESI-TOF) *m*/*z*: [**16a**-H]^−^ calculated for C_28_H_27_O_5_ 443.1853; found 443.1849. Note: Ketone carbon ketone signal is missing. It is not obtained even with a wider spectral window or increased relaxation time.

**16b**: ^1^H NMR (500 MHz, acetone-d6): δ 7.43–7.26 (m, 7H), 7.21 (m, 2H), 7.08–6.95 (m, 3H), 6.77 (m, 2H), 4.96 (s, 2H), 4.14 (d, *J* = 12.2 Hz, 1H), 3.78 (td, *J* = 12.5, 5.2 Hz), 3.07 (dd, *J* = 14.9, 12.8 Hz, 1H), 2.43 (dd, *J* = 14.9, 5.2 Hz, 1H), 1.66 (s, 3H), 1.07 (s, 3H). ^13^C{^1^H} NMR (125 MHz, acetone-d6): δ 210.2, 173.8, 158.2, 139.6, 138.5, 136.4, 129.8, 129.2, 128.6, 128.5, 128.2, 127.4, 115.3, 84.2, 70.3, 53.0, 50.6, 46.3, 44.8, 24.4, 19.1. IR (atr) cm^–1^: 2800–3200 (broad), 1712 (s) (R-COOH), 1678 (s), 1250 (s) (R-**CO**-R), 1467 (m) (R-CH_2_–R), 1426 (m) (R-**CH**_**2**_-CO-R), 1379 (m) (R-CH_3_), 1176 (s) ((CH_3_)_2_-C-R_2_), 1081 (m) (Ph-O-R), 1028 (m), 748 (s) (Ph–CH_2_–O-R), 880 (m), 694 (s) (5 adjacent H (Ph)), 827 (m) (2 adjacent H (R-Ph-R)). HRMS (ESI-TOF) *m*/*z*: [**16b**-H]^−^ calculated for C_28_H_27_O_5_ 443.1853; found 443.1853. Note: one carbon signal is missing, probably due to overlapping at the aromatic region.

## Data Availability

The data underlying this study are available in the published article and its Supporting Information.

## Supporting Information Available

Experimental details and NMR, IR, HRMS and X-ray data for products **1a** (CCDC 2193907), **14A** (CCDC 2193908), and **15a** (CCDC 2193909) (PDF)
FAIR data, including the primary NMR FID files, for compounds **1**–**16** (ZIP)

## References

[ref1] Ledoux A.; St-Gelais A.; Cieckiewicz E.; Jansen O.; Bordignon A.; Illien B.; Di Giovanni N.; Marvilliers A.; Hoareau F.; Pendeville H.; Quetin-Leclercq J.; Frédérich M. Antimalarial Activities of Alkyl Cyclohexenone Derivatives Isolated from the Leaves of *Poupartia borbonica*. J. Nat. Prod. 2017, 80, 1750–1757. 10.1021/acs.jnatprod.6b01019. 28557449

[ref2] Ledoux A.; Bériot D.; Mamede L.; Desdemoustier P.; Detroz F.; Jansen O.; Frédérich M.; Maquoi E. Cytotoxicity of Poupartone B, an Alkyl Cyclohexenone Derivative from *Poupartia borbonica*, against Human Cancer Cell Lines. Planta Med. 2021, 87, 1008–1017. 10.1055/a-1532-2384. 34687029

[ref3] Shakil N. A.; Singh M. K.; Kumar J.; Sathiyendiran M.; Kumar G.; Singh M. K.; Pandey R. P.; Pandey A.; Parmar V. S. Microwave synthesis and antifungal evaluations of some chalcones and their derived diaryl-cyclohexenones. J. Environ. Sci. Health B 2010, 45, 524–530. 10.1080/03601234.2010.493482. 20574873

[ref4] Ghavre M.; Froese J.; Murphy B.; Simionescu R.; Hudlicky T. A Formal Approach to Xylosmin and Flacourtosides E and F: Chemoenzymatic Total Synthesis of the Hydroxylated Cyclohexenone Carboxylic Acid Moiety of Xylosmin. Org. Lett. 2017, 19, 1156–1159. 10.1021/acs.orglett.7b00194. 28186763

[ref5] Miyashita M.; Sasaki M.; Hattori I.; Sakai M.; Tanino K. Total Synthesis of Norzoanthamine. Science 2004, 305, 495–499. 10.1126/science.1098851. 15205476

[ref6] Kuroda Y.; Nicacio K. J.; da Silva I. A. Jr.; Leger P. R.; Chang S.; Gubiani J. R.; Deflon V. M.; Nagashima N.; Rode A.; Blackford K.; Ferreira A. G.; Sette L. D.; Williams D. E.; Andersen R. J.; Jancar S.; Berlinck R. G. S; Sarpong R. Isolation, synthesis and bioactivity studies of phomactin terpenoids. Nat. Chem. 2018, 10, 938–945. 10.1038/s41557-018-0084-x. 30061613

[ref7] Yang X.; Wang J.; Li P. Recent progress on asymmetric organocatalytic construction of chiral cyclohexenone skeletons. Org. Biomol. Chem. 2014, 12, 2499–2513. 10.1039/C3OB42293C. 24599029

[ref8] Mohr P. J.; Halcomb R. L. Total Synthesis of (+)-Phomactin A Using a B-Alkyl Suzuki Macrocyclization. J. Am. Chem. Soc. 2003, 125, 1712–1713. 10.1021/ja0296531. 12580592

[ref9] Bradshaw B.; Bonjoch J. The Wieland-Miescher Ketone: A Journey from Organocatalysis to Natural Product Synthesis. Synlett. 2012, 23, 337–356. 10.1055/s-0031-1290107.

[ref10] Liu Z.-Q. How to Start a Total Synthesis from the Wieland-Miescher Ketone?. Current Organic Synthesis 2019, 16, 328–341. 10.2174/1570179416666190328233710. 31984897

[ref11] Poplata S.; Bach T. Enantioselective Intermolecular [2 + 2] Photocycloaddition Reaction of Cyclic Enones and Its Application in a Synthesis of (−)-Grandisol. J. Am. Chem. Soc. 2018, 140, 3228–3231. 10.1021/jacs.8b01011. 29458250 PMC5849358

[ref12] Huang Y.-m; Zheng C.-w; Zhao G. Asymmetric Robinson-Type Annulation Reaction between β-Ketoamides and α,β-Unsaturated Ketones. J. Org. Chem. 2015, 80, 3798–3805. 10.1021/jo502904n. 25803128

[ref13] Liu Z.-Q. An Overview on the Robinson Annulation. Curr. Org. Chem. 2018, 22, 1347–1372. 10.2174/1385272822666180511122631.

[ref14] Wen Z.-K; Wu X.-X; Bao W.-K; Xiao J.-J; Chao J.-B. Palladium-Catalyzed Regioselective Coupling Cyclohexenone into Indoles: Atom-Economic Synthesis of β-Indolyl Cyclohexenones and Derivatization Applications. Org. Lett. 2020, 22, 4898–4902. 10.1021/acs.orglett.0c01763. 32515596

[ref15] Tang L.; Luo Y.; Xue J.-W; He Y.-H; Guan Z. Highly enantioselective Michael-aldol-dehydration reaction for the synthesis of chiral 3,5-diaryl-cyclohexenones catalysed by primary amine. Tetrahedron 2017, 73, 1114–1119. 10.1016/j.tet.2017.01.004.

[ref16] Lee J.; Wang S.; Callahan M.; Nagorny P. Copper(II)-Catalyzed Tandem Decarboxylative Michael/Aldol Reactions Leading to the Formation of Functionalized Cyclohexenones. Org. Lett. 2018, 20, 2067–2070. 10.1021/acs.orglett.8b00607. 29560721 PMC5889748

[ref17] Chen L.; Luo S.; Li J.; Li X.; Cheng J.-P. Organocatalytic kinetic resolution *via* intramolecular aldol reactions: Enantioselective synthesis of both enantiomers of chiral cyclohexenones. Org. Biomol. Chem. 2010, 8, 2627–2632. 10.1039/b927343c. 20358094

[ref18] Kimaru N.; Komatsuki K.; Saito K.; Yamada T. Decarboxylation-triggered homo-Nazarov cyclization of cyclic enol carbonates catalyzed by rhenium complex. Chem. Commun. 2021, 57, 6133–6136. 10.1039/D1CC01144H. 34042121

[ref19] Golec J. C.; Carter E. M.; Ward J. W.; Whittingham W. G.; Simón L.; Paton R. S.; Dixon D. J. BIMB-Catalyzed 1,3-Prototropic Shift for the Highly Enantioselective Synthesis of Conjugated Cyclohexenones. Angew. Chem., Int. Ed. 2020, 59, 17417–17422. 10.1002/anie.202006202. PMC7540019 32558981

[ref20] Kobzev M. S.; Titov A. A.; Varlamov A. V. Synthesis of heterocyclic systems involving [3,3]-sigmatropic rearrangements. Russ. Chem. Bull., Int. Ed. 2021, 70, 1213–1259. 10.1007/s11172-021-3208-1.

[ref21] Huang X.; Klimczyk S.; Maulide N. Charge-Accelerated Sulfonium [3,3]-Sigmatropic Rearrangements. Synthesis 2012, 2012, 175–183. 10.1055/s-0031-1289632.

[ref22] Ilardi E. A.; Stivala C. E.; Zakarian A. [3,3]-Sigmatropic rearrangements: recent applications in the total synthesis of natural products. Chem. Soc. Rev. 2009, 38, 3133–3148. 10.1039/b901177n. 19847347 PMC4103198

[ref23] Jones A. C.; May J. A.; Sarpong R.; Stoltz B. M. Toward a Symphony of Reactivity: Cascades Involving Catalysis and Sigmatropic Rearrangements. Angew. Chem., Int. Ed. 2014, 53, 2556–2591. 10.1002/anie.201302572. PMC4030764 24677683

[ref24] de la Pradilla R. F.; Tortosa M.; Viso A. Sulfur Participation in [3,3]-Sigmatropic Rearrangements. Top. Curr. Chem. 2006, 275, 103–129. 10.1007/128_059. 23605511

[ref25] Eronen A. E. K; Mannisto J. K.; Moslova K.; Nieger M.; Heliövaara E.; Repo T. Synthesis of Diaryl Hydrocyl Dicarboxylic Acids from Amino Acids. J. Org. Chem. 2020, 85, 5799–5806. 10.1021/acs.joc.9b03320. 32126166 PMC7497638

[ref26] Kristensen-Reh M. Reaction of phenylpyruvic acid with benzylidene- and anisylidene-acetone and corresponding saturated ketones. Bull. Soc. Chim. Fr. 1956, 882–887.

[ref27] Katsumura S.; Isoe S. An Efficient Synthesis of Jolkinolide E Involving the Butenolide Ring Formation by Intramolecular Wittig Reaction. Chem. Lett. 1982, 11, 1689–1692. 10.1246/cl.1982.1689.

[ref28] Kimura A.; Katsumura S.; Isoe S. Total Synthesis of 6-Oxo-Grindelic Acid Methyl Ester. Chem. Lett. 1983, 12, 15–16. 10.1246/cl.1983.15.

