# Supplementary material for: Stereospecific Synthesis of Cyclohexenone Acids by [3,3]-Sigmatropic Rearrangement Route
Source: J Org Chem. 2023 Sep 1;88(18):12914–23. doi: 10.1021/acs.joc.3c00757 (PMC10507681; doi:10.1021/acs.joc.3c00757)
Supplement: Supplementary file 1 — jo3c00757_si_001.pdf [file jo3c00757_si_001.pdf]

Stereospecific Synthesis of Cyclohexenone Acids by [3,3]-Sigmatropic Rearrangement Route

Aleksi Eronen†, Martin Nieger†, Tommi A. Kajander‡ and Timo Repo†*

†Address: PO Box 55, A.I. Virtasen aukio 1, 00014, Helsinki, Finland

Department of Chemistry, University of Helsinki

‡Address: PO Box 65, Viikinkaari 1, 00014, Helsinki, Finland

Institute of Biotechnology, University of Helsinki

*email: timo.repo@helsinki.fi

Supplementary information

<u>Table of Contents</u>

1. Experimental section S2
 1.1 General procedures for cyclohexenone acid synthesis S2
  1.1.1 Synthesis from pyruvic acids from pre-synthesized aromatic enone S2
  1.1.2 Synthesis from pyruvic acids from *in situ* synthesis aromatic enone S2
  1.1.3 Synthesis of cyclohexenone acids in water S3
 1.2 Purification of **2** S3
2. ESI-TOF-HRMS parameters S4
3. Spectroscopic data of cyclohexenone acids S5
 3.1 Spectroscopic data of **1a** S5
 3.2 Spectroscopic data of **1b** S12
 3.3 Spectroscopic data of **1A** S19
 3.4 Spectroscopic data of **2** S26
 3.5 Spectroscopic data of **3** S38
 3.6 Spectroscopic data of **4** S45
 3.7 Spectroscopic data of **5** S52
 3.8 Spectroscopic data of **6** S61
 3.9 Spectroscopic data of **7** S68
 3.10 Spectroscopic data of **8** S75
 3.11 Spectroscopic data of **9** S84
 3.12 Spectroscopic data of **10** S92
 3.13 Spectroscopic data of **11** S99
 3.14 Spectroscopic data of **12a** S108
 3.15 Spectroscopic data of **12b** S115
 3.16 Spectroscopic data of **13a** S122
 3.17 Spectroscopic data of **13b** S129
 3.18 Spectroscopic data of **14a** S136
 3.19 Spectroscopic data of **14b** S144
 3.20 Spectroscopic data of **14A** S151
 3.21 Spectroscopic data of **15a** S158
 3.22 Spectroscopic data of **15b** S165
 3.23 Spectroscopic data of **16a** S172
 3.24 Spectroscopic data of **16b** S179
4. The single-crystal X-ray diffraction studies S186
 4.1 Single crystal growing and data S186

1 Experimental section

1.1 General procedures for cyclohexenone acid synthesis

1.1.1 Synthesis from pyruvic acids and pre-synthesized aromatic enone

Pyruvic acid (2 mmol), 4-phenyl-3-buten-2-one (2 mmol), NaOH (3 mmol, 300 µL, 10M) and toluene (2 ml) were charged into a microwave vial (10 ml). The vial was capped and heated to 135 °C within 5 min with a microwave reactor. The temperature was maintained for 15 min. Then the vial was cooled to 50 °C using compressed air (see microwave heating profile at Fig. S1). Following this acetone (5 ml) was added to the vial to initiate the crystallization. The next day the formed powder was collected by filtration, washed with acetone, and dried under air. The obtained product was dissolved with water and HCl (1M) was added until the formation of a white powder stopped. The powder was filtered, washed with water, and dried under vacuum.

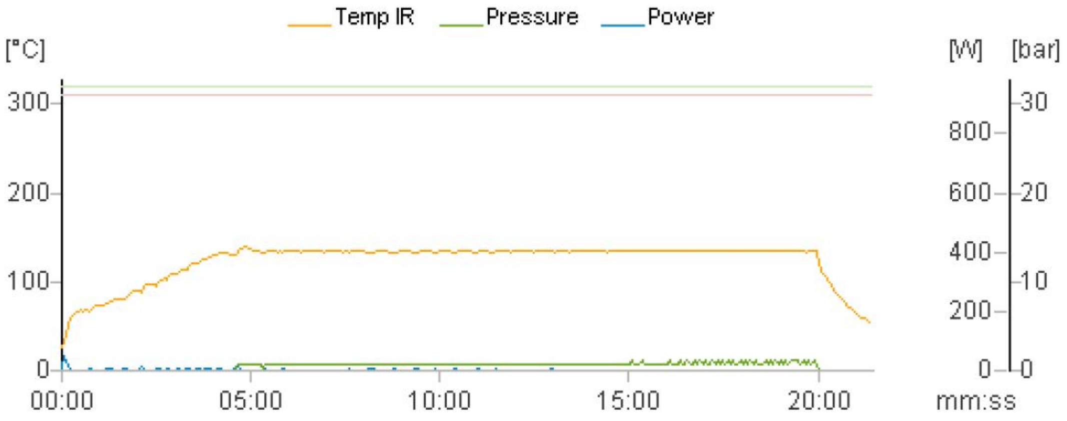

*Figure S1: Microwave heating profile of synthesis of **1a** (5+15 min) using pre-made enone at toluene (2 ml).*

1.1.2 Synthesis from pyruvic acids and *in situ* synthesized aromatic enone

Pyruvic acid (2 mmol), benzaldehyde (2 mmol), ketone (2 mmol), NaOH (3 mmol) and *tert*-BuOH (3ml) were added into a 10 mL microwave vial. The heating program was set to heat the microwave vial to 135 °C in 5 min and maintain the temperature for 30 minutes. The stirring speed was set to 600 rpm. Next, the microwave vial was cooled down to 50 °C with compressed air (see microwave heating profile at Fig. S2). After cooling, acetone was added to initiate the crystallization overnight. The following day the formed product salt was collected by filtration and washed with acetone and dried under air. Then, the salt of the product was dissolved with water and 1M HCl was added until all the product had precipitated from the solution. The formed powder was filtered, washed with water, and dried under vacuum.

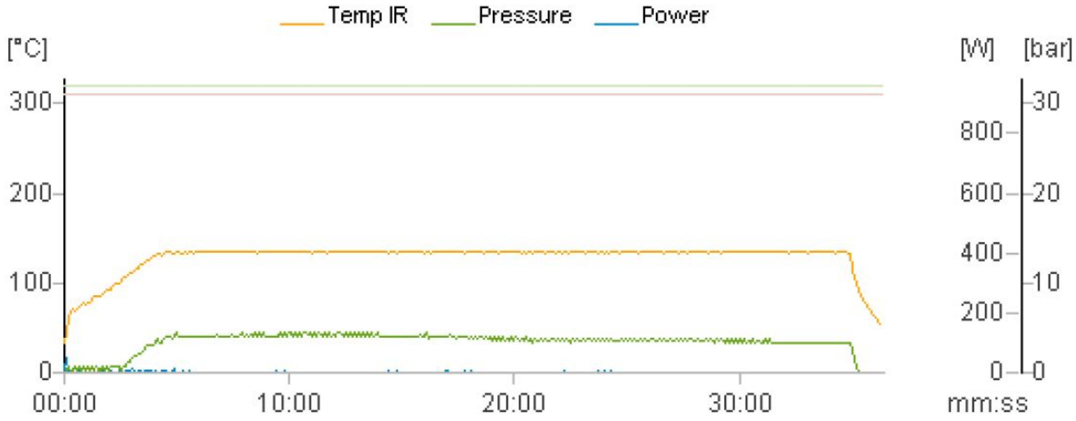

*Figure S2: Microwave heating profile of synthesis of **1a** (5+30 min) using in situ made enone at tert-BuOH (3 ml).*

1.1.3 Synthesis of cyclohexenone acids in water

Phenylpyruvic acid (2 mmol), enone (2 mmol) (in case of *in situ* synthesized enone corresponding ketone (2 mmol) and aromatic aldehyde (2 mmol) was used instead), NaOH (3 mmol, 300 µL, 10M) and milli-Q water (3 ml) were charged into a microwave vial (10 ml). The vial was capped and heated to 135 °C as fast as possible with a microwave reactor. The temperature was maintained for 30 min. Then the vial was cooled to 50 °C using compressed air (see microwave heating profile at Fig. S3). Following this HCl (1M, ca 4 ml) was added into the microwave vial until precipitation formation stopped. The next day the formed powder was collected by filtration, washed with water and n-hexane, and dried under vacuum.

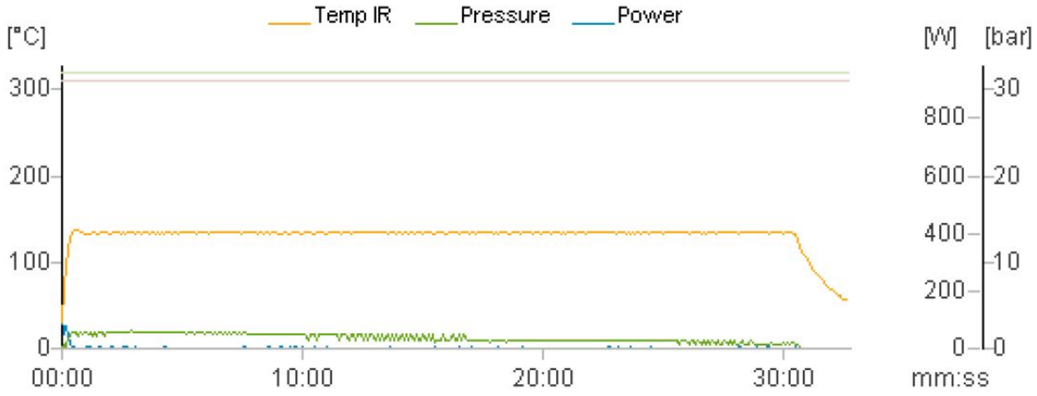

*Figure S3: Microwave heating profile of synthesis of **1a** (30 min) using in situ made enone at H_2O (3 ml).*

1.2 Purification of 2

Purification: After reaction, the remaining solvent in the microwave tube was evaporated. Next the crude powder was washed with hexane. The crude powder was then dissolved with water and filtered. The filtrate was protonated by adding HCl until the formation of a cloudy precipitate stops. The precipitate was difficult to filter, thus it was dried under vacuum. The protonated powder was moved into a clean vial and dissolved with ethanol (ca. 3 ml). In the following weeks each day a small amount of water (ca. 300 µL) was added each day to the vial. First week mainly a brown-red powder was formed from the slow crystallization process. The brown-red powder was filtered out and the crystallization process continues until colourless crystals are formed (See Fig. S3 as reference picture). This crystallization must be done very slowly in order to get good high purity crystals. The crystals are filtered and washed with water and dried under vacuum. Colourless crystals in an epimeric mixture were isolated with 60 mg (10%) yield. Epimers RR/SS and RS/SR forms in 69% and 31% ratio respectively.

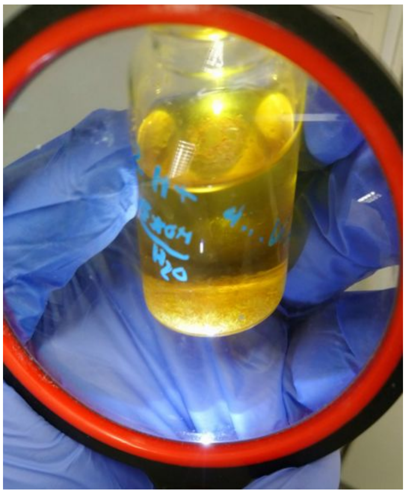

*Figure S4: Product **2** crystallization from EtOH (solvent) / H2O (anti-solvent) solution after 3 weeks of crystallization.*

2 ESI-TOF-HRMS parameters

Table S1: Example parameters at negative-ion mode:

End Plate Offset	-500 V	Capillary Exit	-100 V	Skimmer 1	-35.0 V	negative Ion Polarity
Capillary	+4500 V	Hexapole 1	-23.0 V	Skimmer 2	-23 V	Mass Range 50-1200 m/z
Nebulizer	0.4 bar	Hexapole 2	-20.2 V	Hexapole RF	80.0 Vpp	Rollin Average 3 x 1.0 Hz
Dry Gas	4.0 L min^{-1}	Transfer time	3.0 µs	Pre Puls Storage	1.0 µs	
Dry Temp	180 °C	Lens 1 Storage	-21.5 V	Lens 1 Extraction	-21.3 V	
		Lens 2	2 V	Lens 3	30.0 V	
		Lens 4	-1.0 V	Lens 5	33.5 V	
		Detector	0 V			

The instrument was calibrated with sodium formate. The samples were measured as 1 ppm concentrations from acetonitrile solutions.

3 Spectroscopic data of cyclohexenone acids

3.1 Spectroscopic data of 1a

1D NMR of **1a**

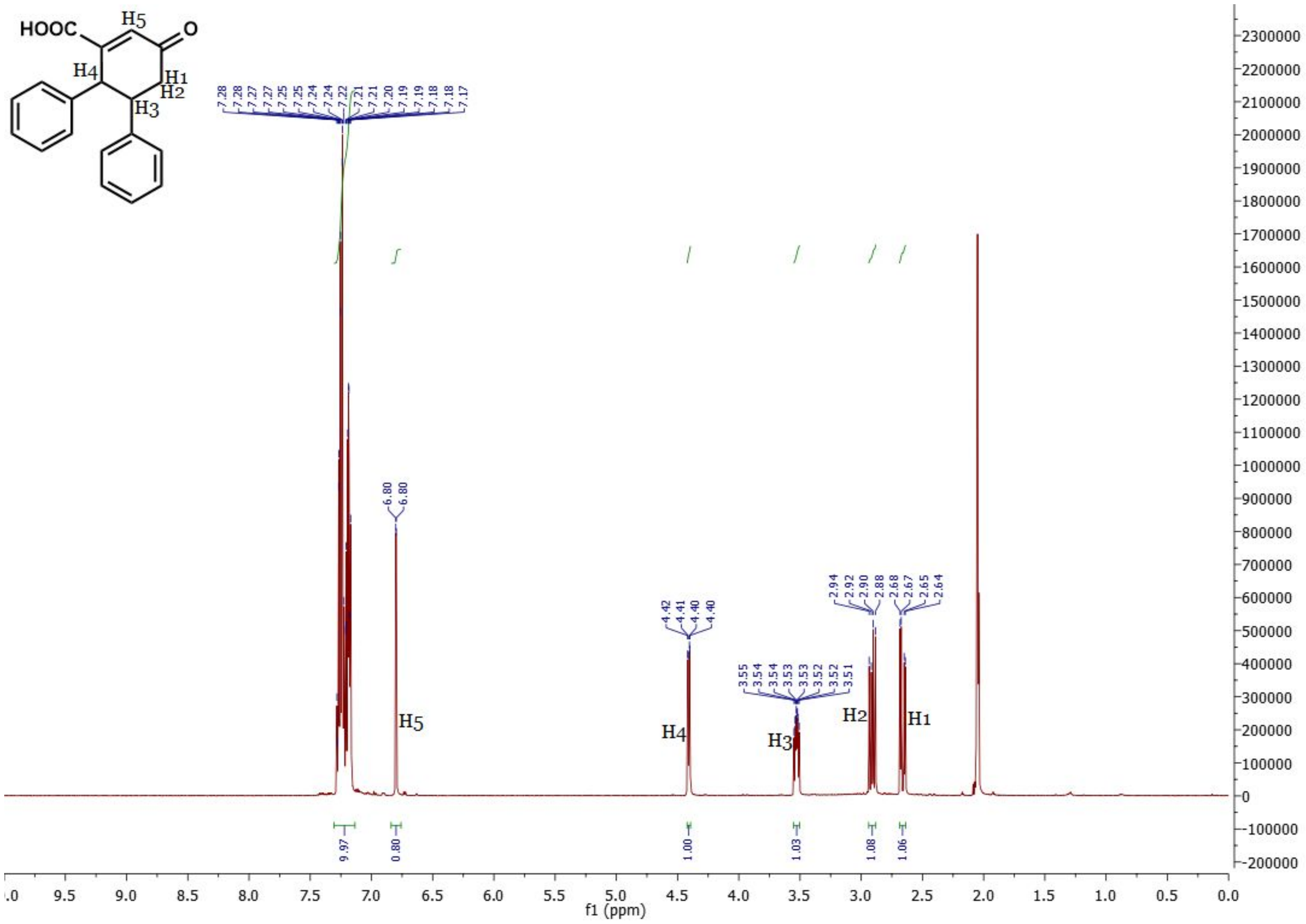

*Figure S5: ^{1}H NMR spectrum of **1a** (500 MHz, acetone-d6): δ 7.29 – 7.16 (m, 10H), 6.80 (d, J = 1.8 Hz, 1H), 4.41 (dd, J = 6.8, 1.6 Hz, 1H), 3.53 (ddd, J = 9.3, 6.8, 4.6 Hz, 1H), 2.91 (dd, J = 16.4, 9.3 Hz, 1H), 2.66 (dd, J = 16.4, 4.6 Hz, 1H).*

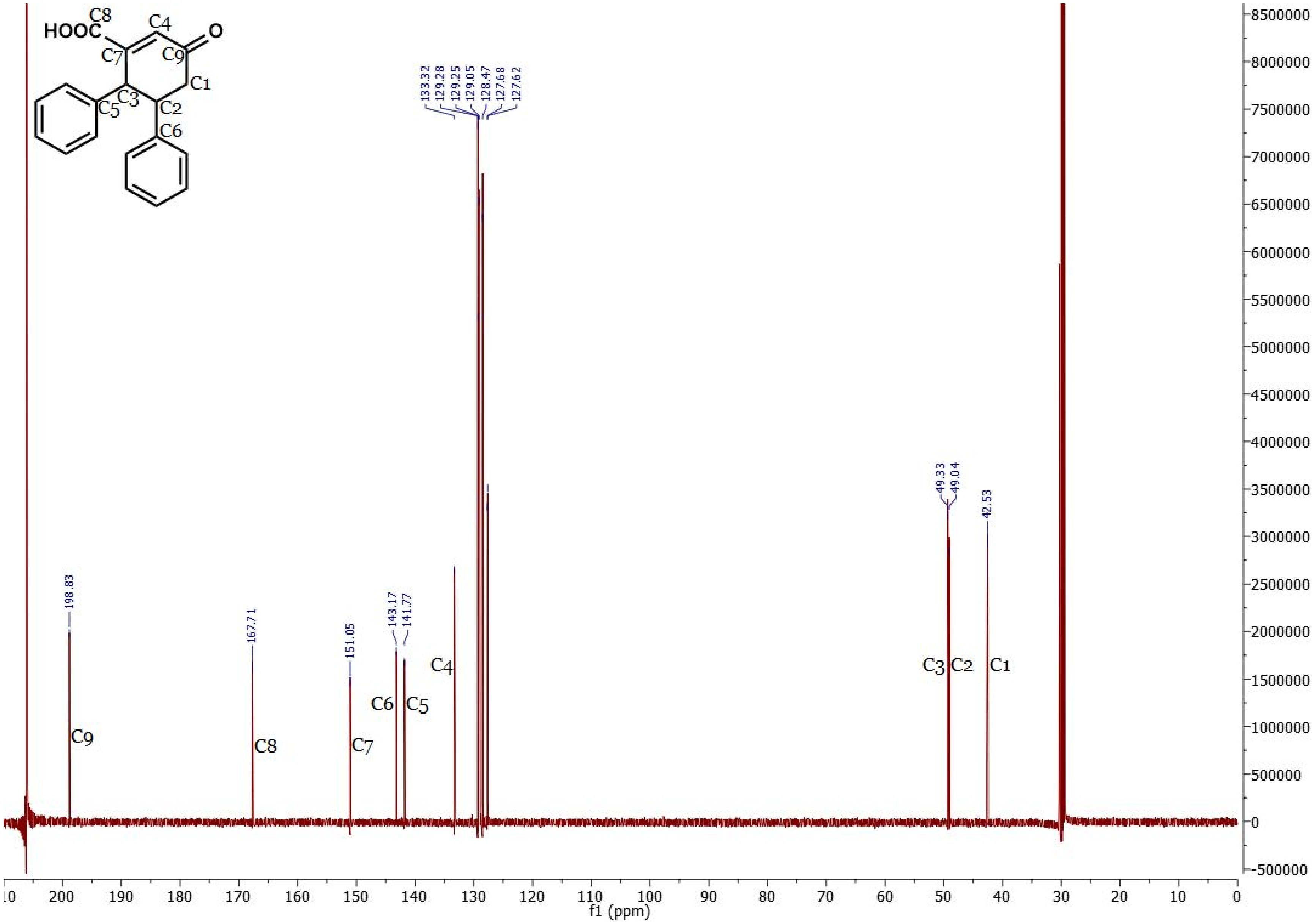

*Figure S6: $^{13}C\{^{1}H\}$ NMR spectra of **1a** (125 MHz, acetone-d6): δ 198.83, 167.71, 151.05, 143.17, 141.77, 133.32, 129.28, 129.25, 129.05 128.47, 127.68, 127.62, 49.33, 49.04, 42.53.*

2D NMR of **1a**

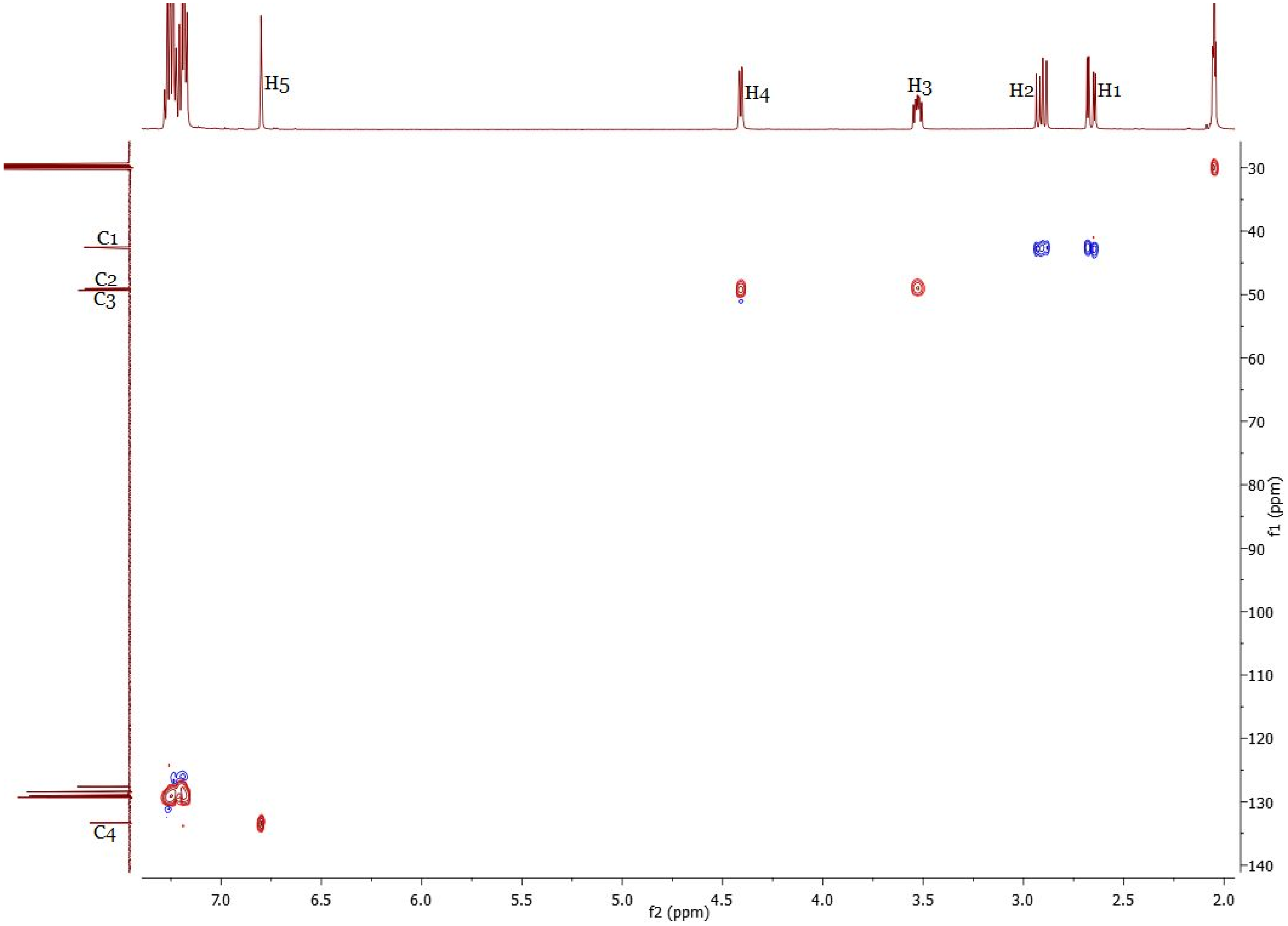

*Figure S7: HSQC spectrum of **1a**.*

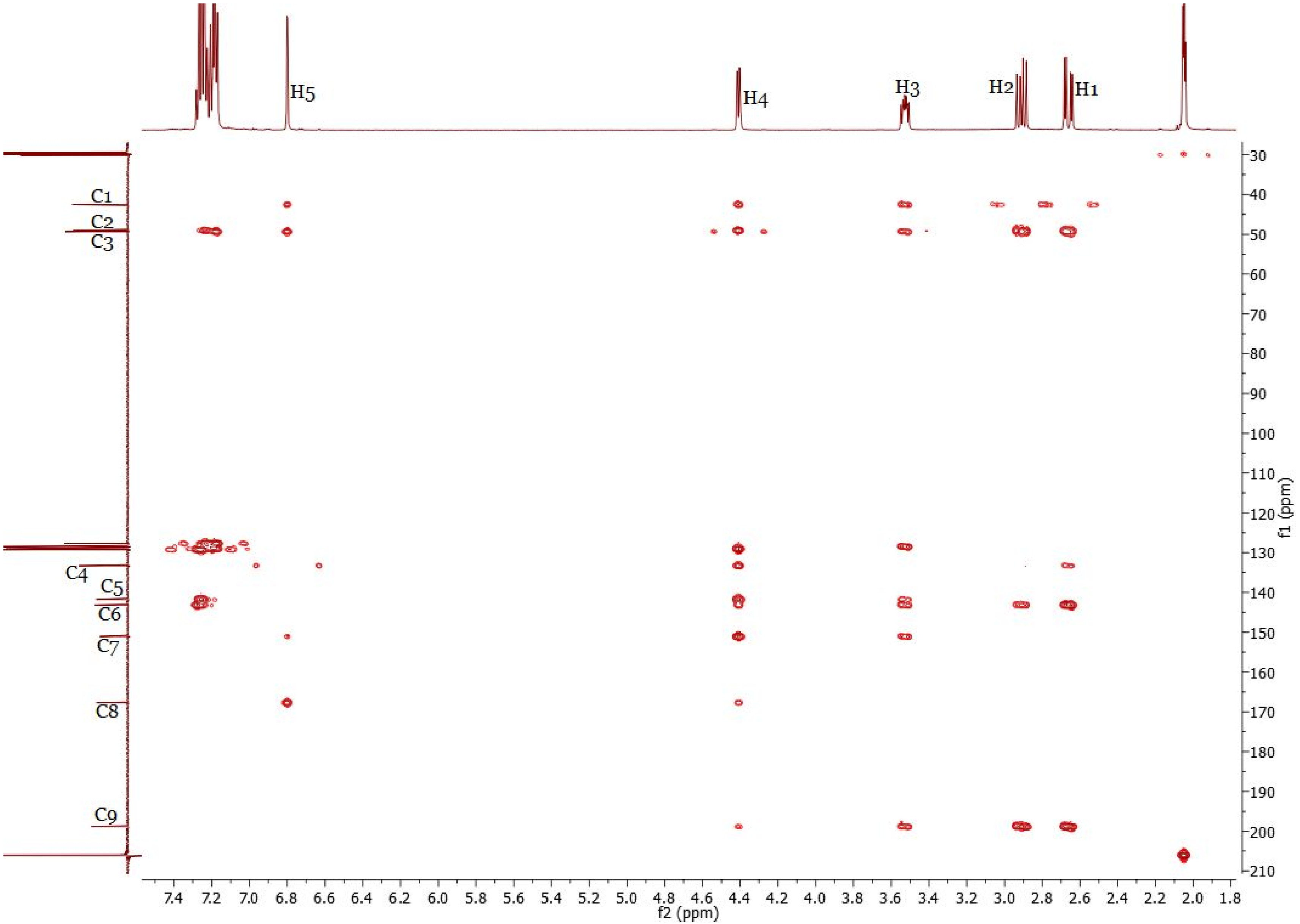

*Figure S8: HMBC spectrum of **1a**.*

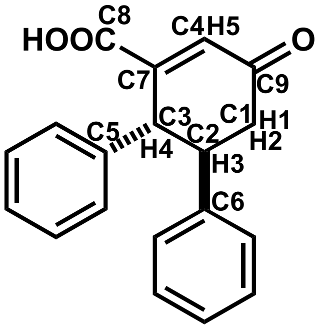

*Figure S9: 2D NMR observations of **1a**.*

2D NMR observations of **1a**:

Protons H1 and H2 are attached to carbon C1 forming CH_2 group. The group has connectivity to carbons C2, C3, C4 (weak), C6 and C9.

Proton H3 is attached to carbon C2 forming CH group. The group has connectivity to carbons C1, C3, C5, C6, C7 and C9. The group has connectivity to inside of aromatic ring, suggesting nearby location.

Proton H4 is attached to carbon C3 forming CH group. The group has connectivity to carbons C1, C2, C4, C5, C6, C7, C8 and C9. The group has connectivity to inside of aromatic ring, suggesting nearby location.

Proton H5 is attached to carbon C4 forming CH group. The group has connectivity to carbons C1, C3, C5 (very weak), C7 and C8.

IR spectroscopy of **1a**

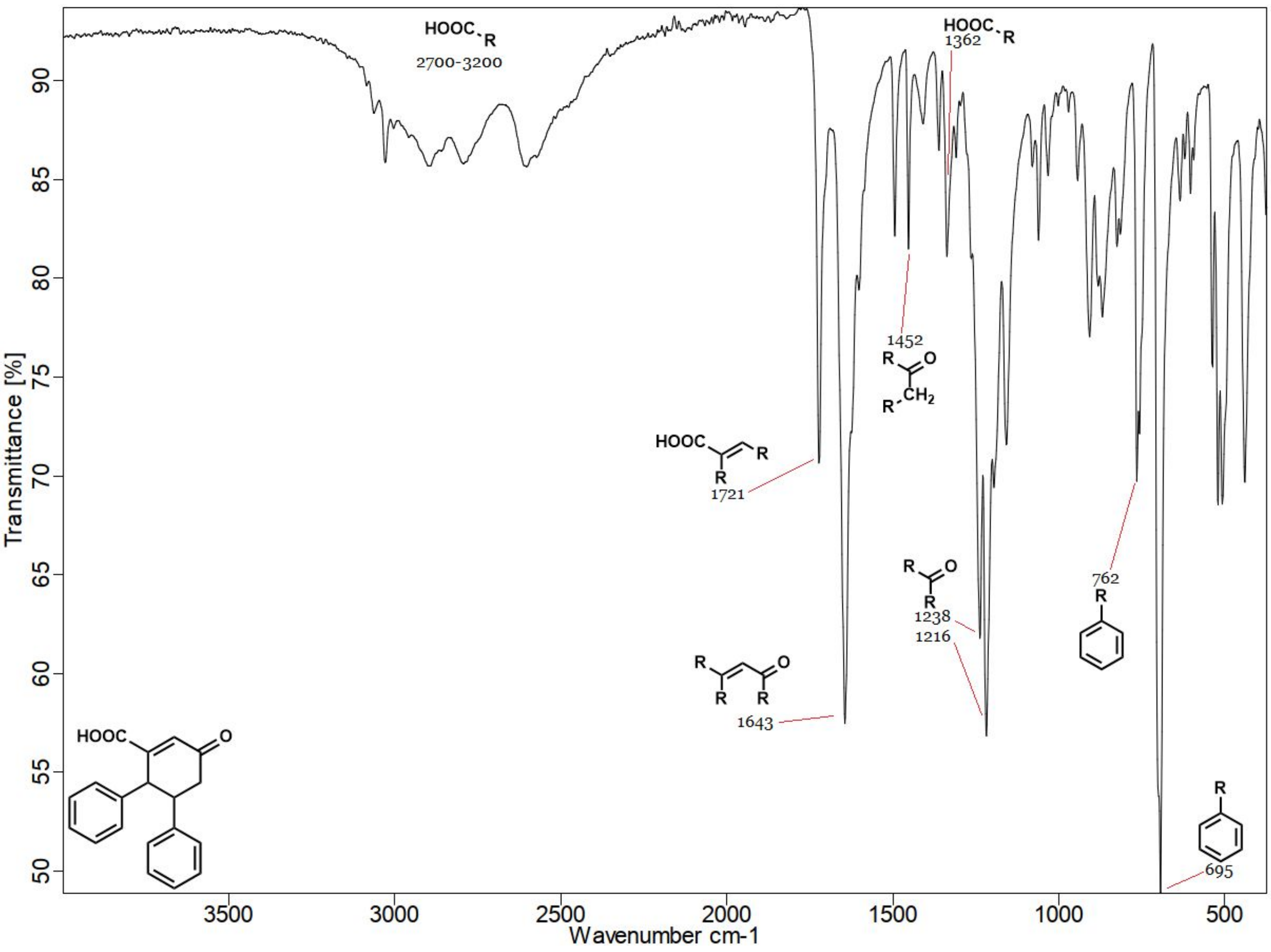

*Figure S10: IR spectrum of **1a** (2700-3200 (broad), 1362 (m) (R-COOH), 1721 (s) (C=C-ROOH), 1643 (s) (C=C-**CO**-R), 1452 (m) (R-CO-**CH₂**-R), 1238 (s), 1216 (s) (R-**CO**-R), 762 (s), 695 (s) (5 adjacent H (Ph)) cm^{-1}).*

HRMS of **1a**

HRMS (ESI-TOF) m/z: [**1a**-H]$^-$ calculated for $C_{19}H_{15}O_3$ 291.1016; Found 291.1010; Error 1.962 ppm.

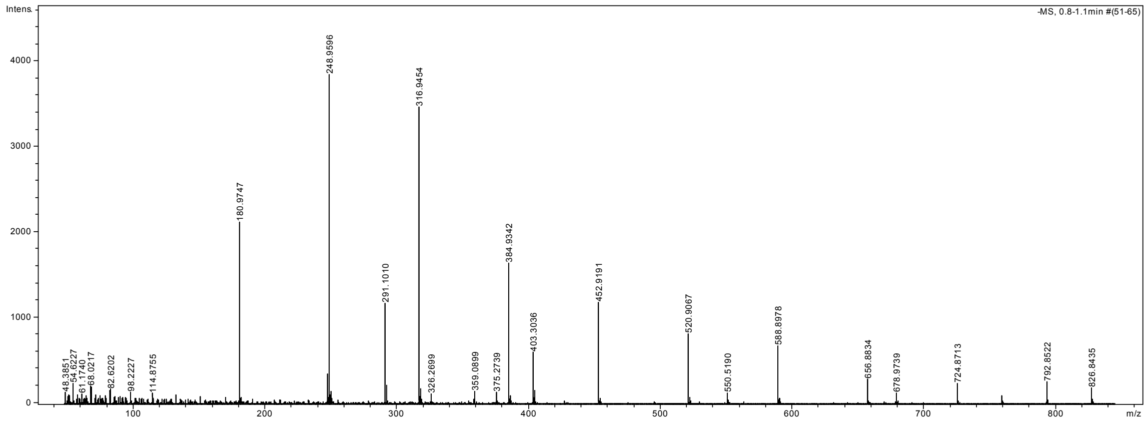

*Figure S11: ESI-TOF-MS of [**1a**-H]$^-$ (peak: 291.1010 m/z, negative-ion mode).*

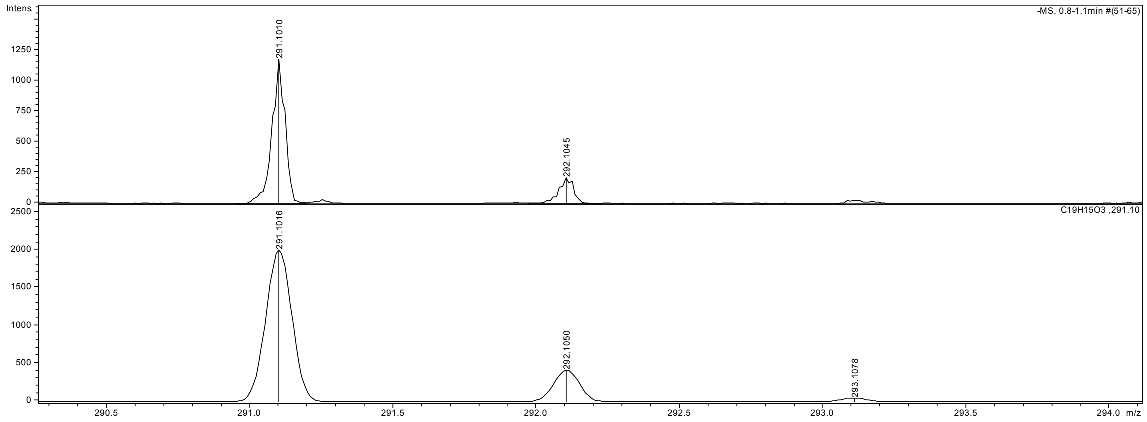

*Figure S12: Measured compound peak of [**1a**-H]$^-$ (291.1010 m/z) at top, simulated peak ($C_{19}H_{15}O_3$) below.*

3.2 Spectroscopic data of 1b

1D NMR of **1b**

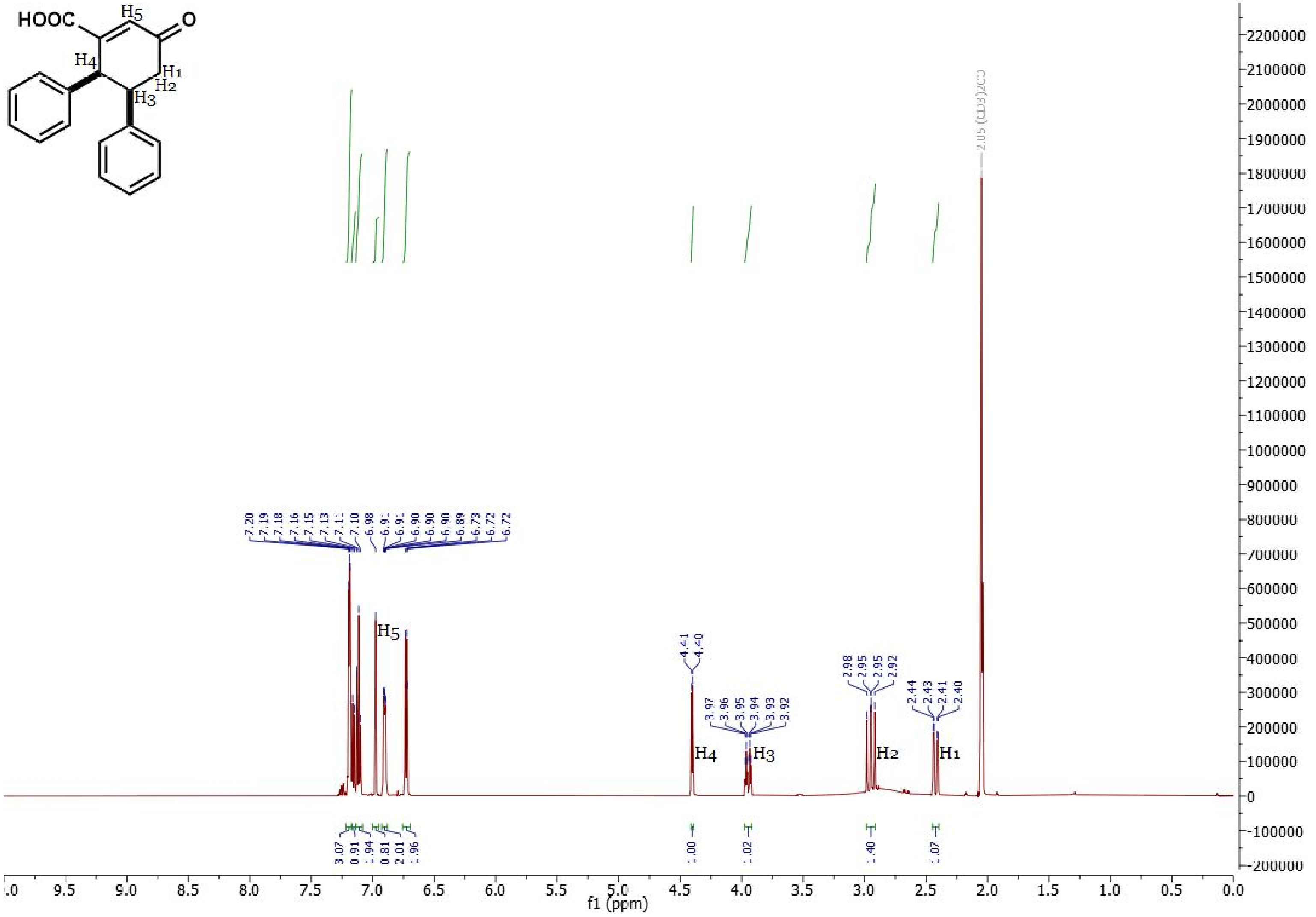

*Figure S13: ^{1}H NMR spectrum of **1b** (500 MHz, acetone-d6): δ 7.21-7.17 (m, 3H), 7.17-7.14 (m, 1H), 7.14-7.09 (m, 2H), 6.98 (s, 1H), 6.90 (m, 2H), 6.72 (m, 2H), 4.40 (d, J = 4.9 Hz, 1H), 3.95 (dt, J = 15.1, 4.4 Hz, 1H), 2.95 (dd, J = 17.0, 15.0 Hz, 1H), 2.42 (dd, J = 16.9, 3.7 Hz, 1H).*

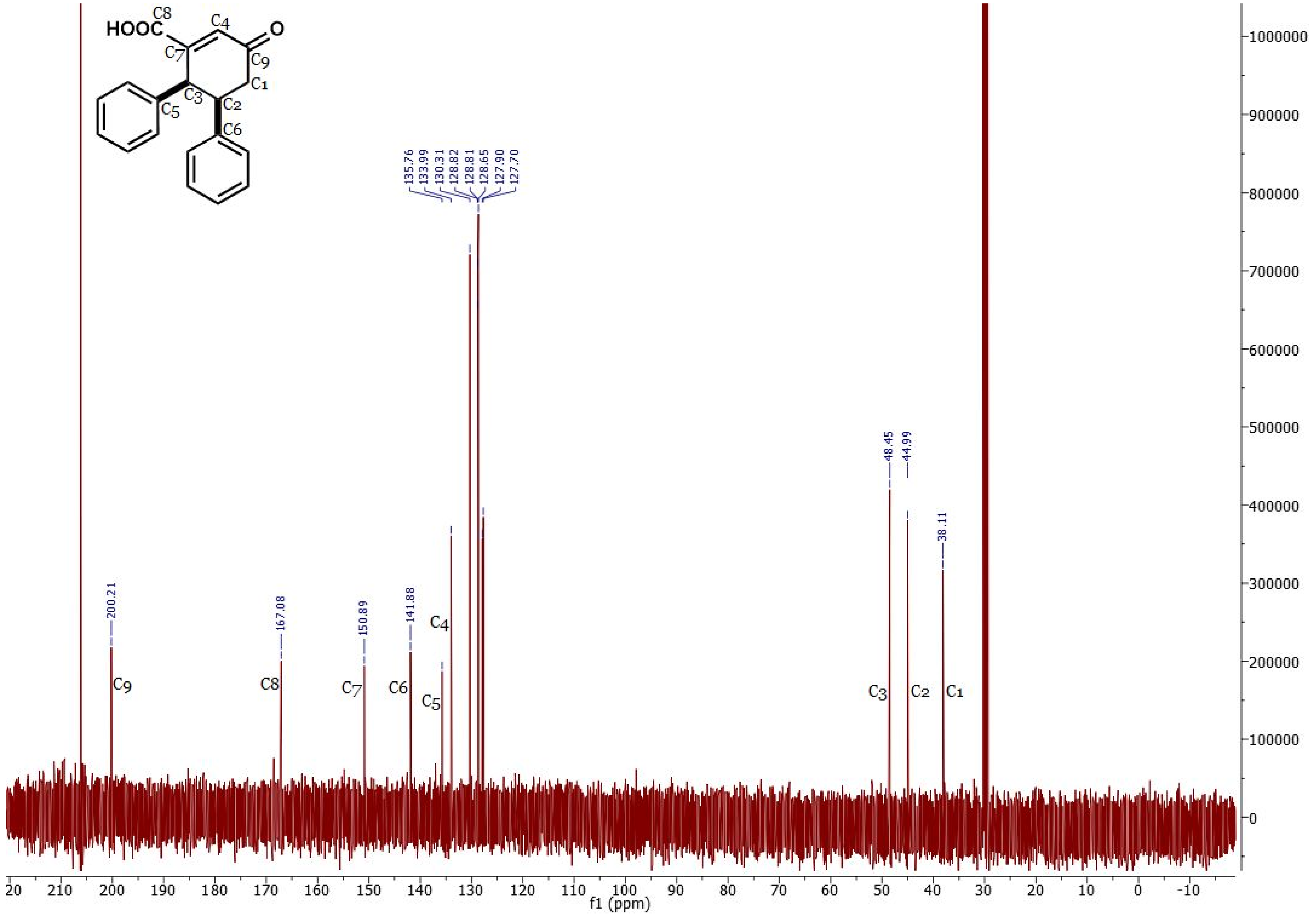

*Figure S14: $^{13}C\{^1H\}$ NMR spectra of **1b** (125 MHz, acetone-d6): δ 200.21, 167.08, 150.89, 141.88, 135.76, 133.99, 130.31, 128.82, 128.81, 128.65, 127.90, 127.70, 48.45, 44.99, 38.11.*

2D NMR of **1b**

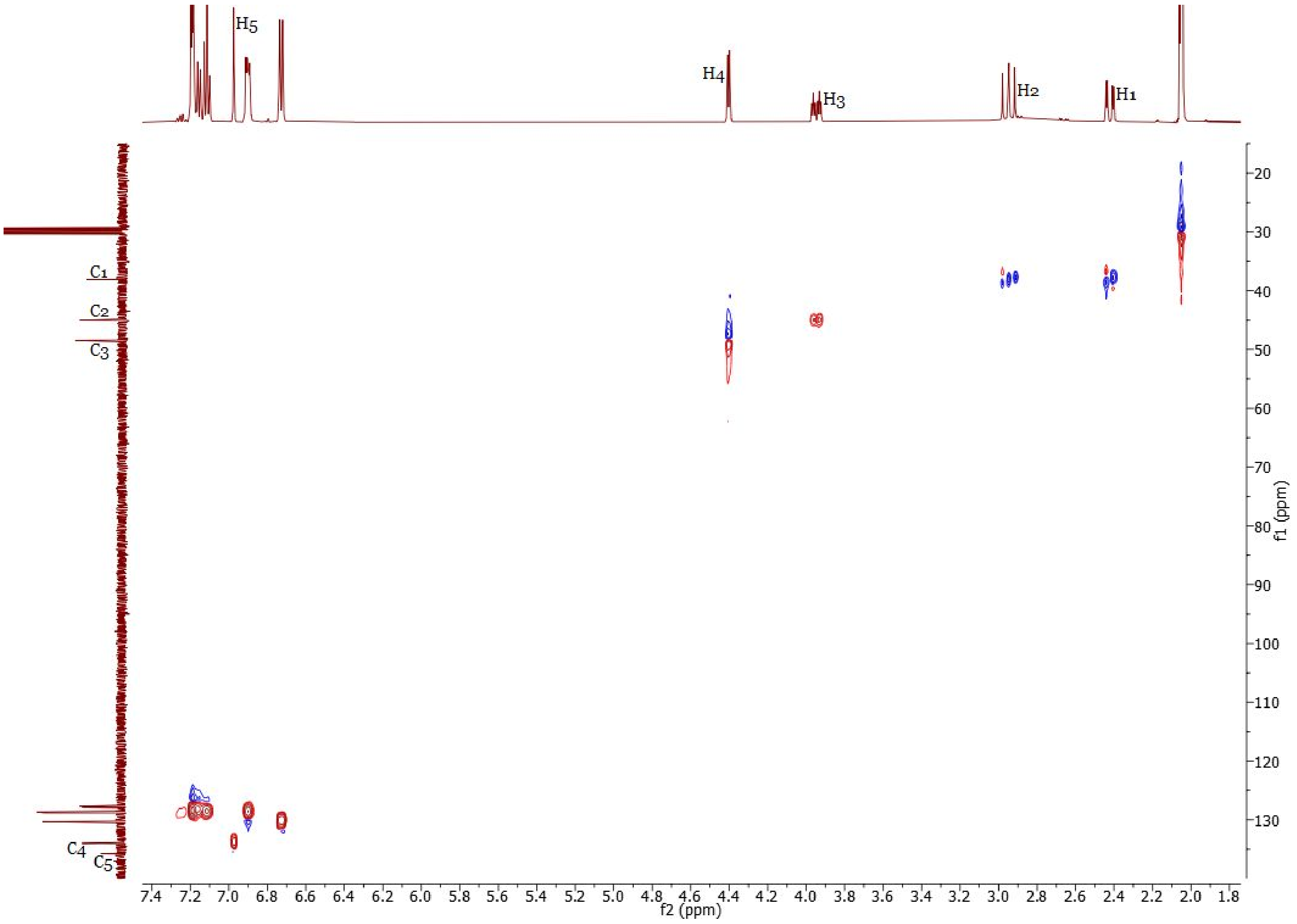

*Figure S15: HSQC spectrum of **1b**.*

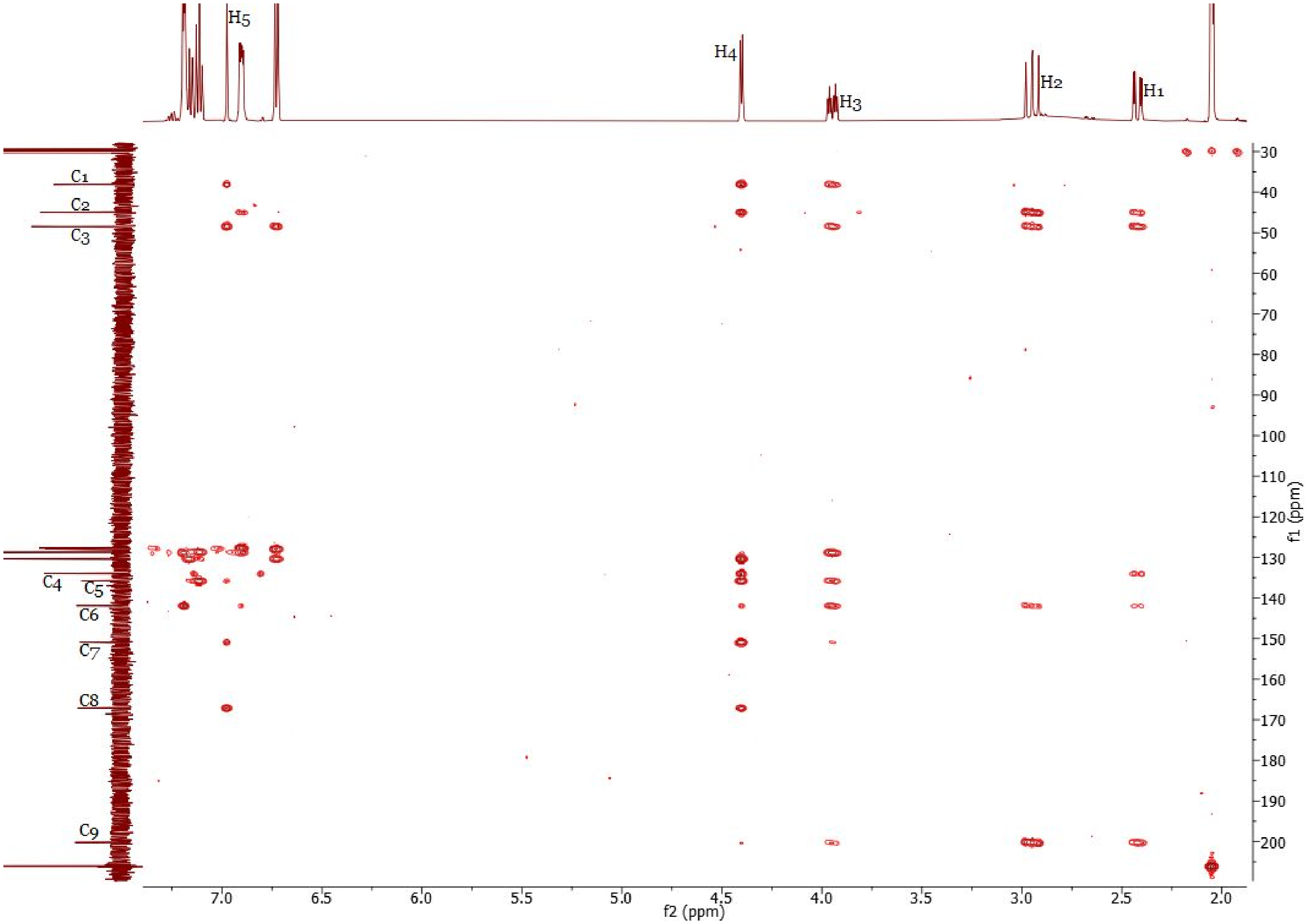

*Figure S16: HMBC spectrum of **1b**.*

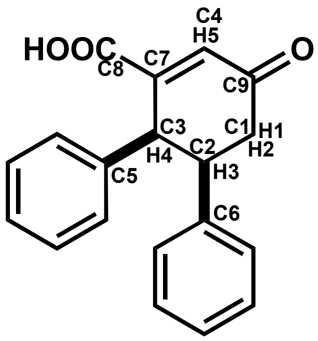

*Figure S17: 2D NMR observations of **1b**.*

2D NMR observations of **1b**:

Protons H1 and H2 are attached to carbon C1 forming CH_2 group. The group has connectivity to carbons C2, C3, C4, C6 and C9.

Proton H3 is attached to carbon C2 forming CH group. The group has connectivity to carbons C1, C3, C5, C6, C7 (weak) and C9 (weak). The group has connectivity to inside one of the phenyl groups, suggesting nearby location.

Proton H4 is attached to carbon C3 forming CH group. The group has connectivity to carbons C1, C2, C4, C5, C6, C7, C8 and C9 (very weak). The group has connectivity to inside one of the phenyl groups, suggesting nearby location.

Proton H5 is attached to carbon C4 forming CH group. The group has connectivity to carbons C1, C3, C5 (weak), C7 and C8.

IR spectroscopy of **1b**

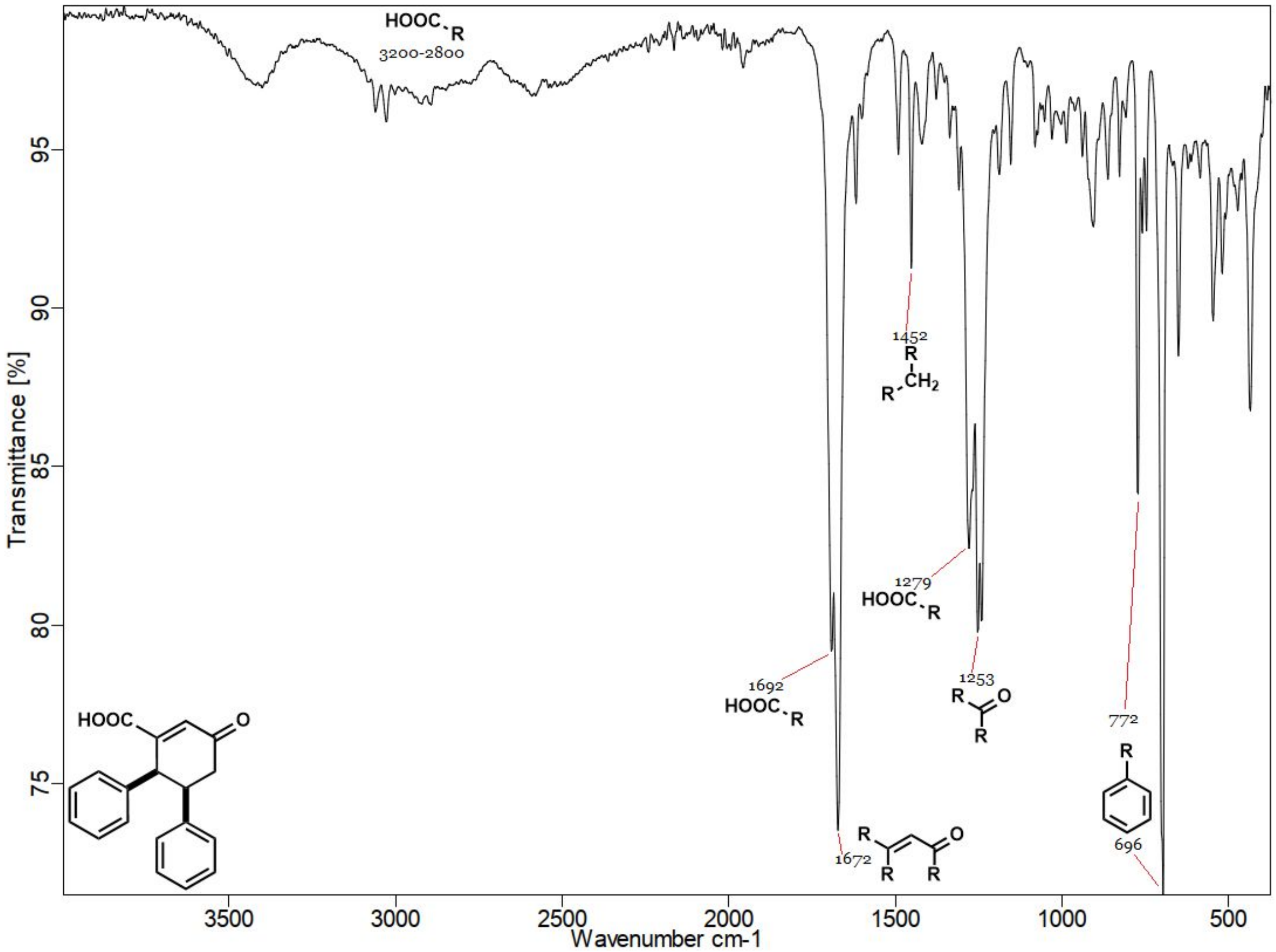

*Figure S18: IR spectrum of **1b** (2800-3200 (broad), 1279 (s) (R-COOH), 1692 (s) (C=C-ROOH), 1672 (s) (C=C-**CO**-R), 1452 (m) (R-CO-**CH_2**-R), 1253 (s) (R-**CO**-R), 772 (s), 696 (s) (5 adjacent H (Ph)) cm^{-1}).*

HRMS of **1b**

HRMS (ESI-TOF) m/z: [**1b**-H]$^-$ calculated for $C_{19}H_{15}O_3$ 291.1016; Found 291.1016; Error 0.123 ppm.

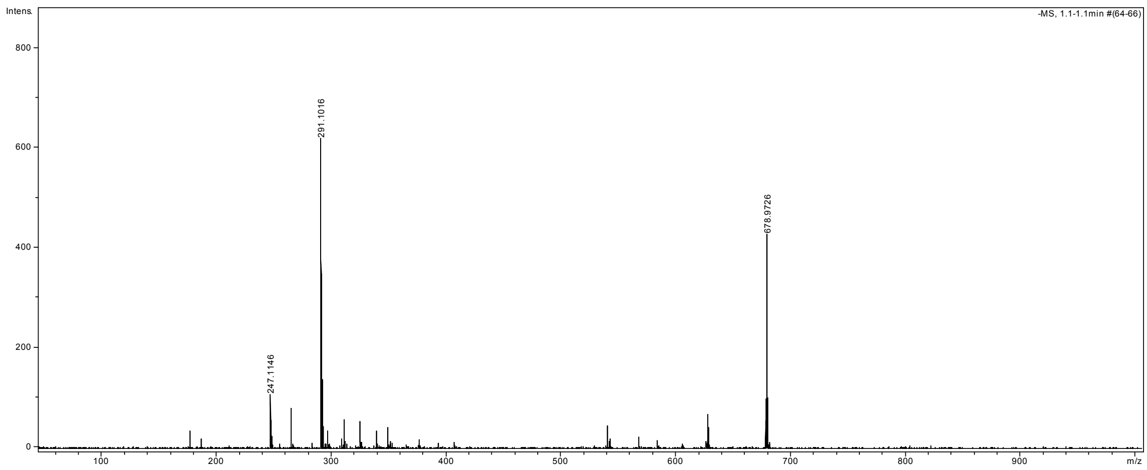

*Figure S19: ESI-TOF-MS of [**1b**-H]$^-$ (peak: 291.1016 m/z, negative-ion mode).*

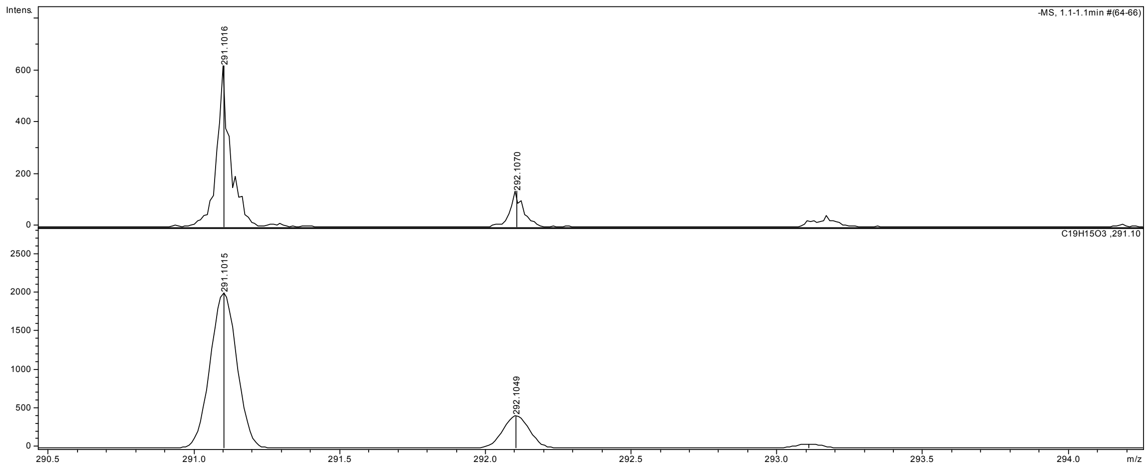

*Figure S20: Measured compound peak of [**1b**-H]$^-$ (291.1016 m/z) at top, simulated peak ($C_{19}H_{15}O_3$) below.*

3.3 Spectroscopic data of 1A

1D NMR of **1A**

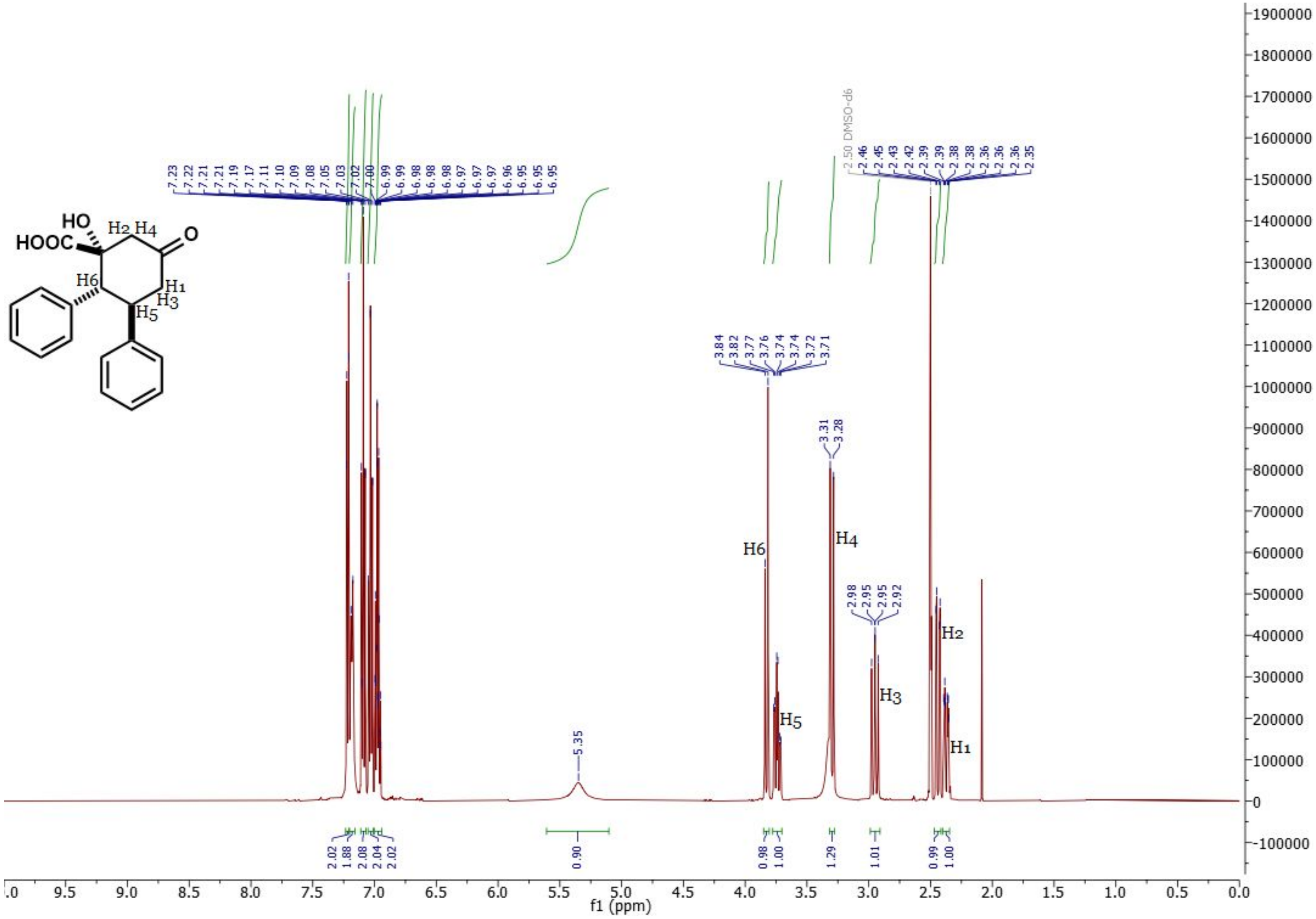

*Figure S21: ^{1}H NMR spectrum of **1A** (500 MHz, DMSO-d6): 7.24-7.20 (m, 2H), 7.20-7.16 (m, 2H), 7.11-7.07 (m, 2H), 7.05-7.01 (m, 2H), 7.00-6.95 (m, 2H), 5.35 (s (broad, 1H, OH proton), 3.83 (d, J = 12.2 Hz, 1H), 3.74 (td, J = 12.3, 4.4 Hz, 1H), 3.30 (d, J = 13.6 Hz, 1H), 2.95 (dd, J = 14.1, 12.5 Hz, 1H), 2.44 (dd, J = 13.7, 2.3 Hz, 1H), 2.37 (ddd, J = 14.3, 4.4, 2.3 Hz, 1H).*

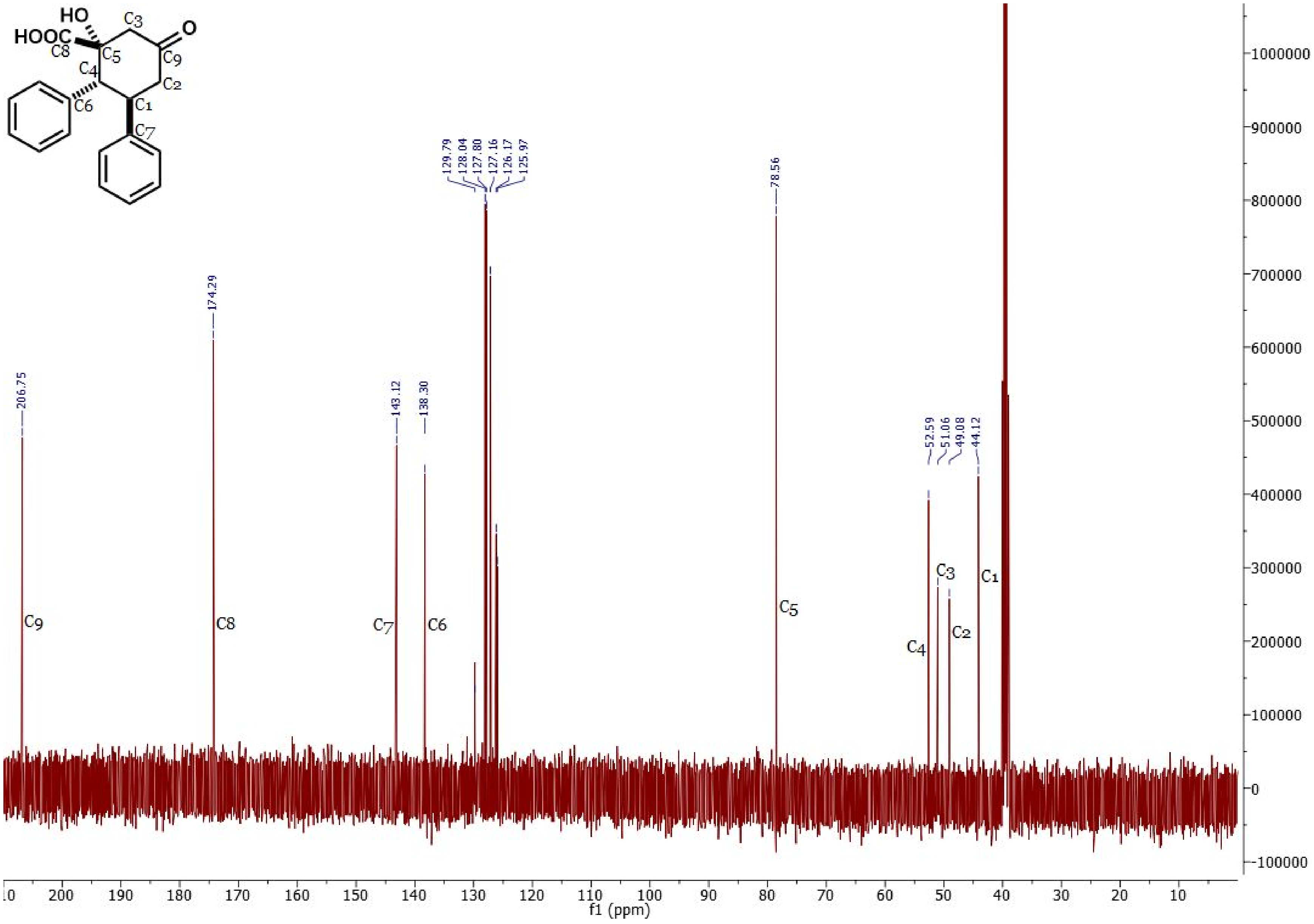

*Figure S22: $^{13}C\{^{1}H\}$ NMR spectra of **1A** (125 MHz, DMSO-d6): δ 206.75, 174.29, 143.12, 138.30, 129.79, 128.04, 127.80, 127.16, 126.17, 125.97, 78.56, 52.59, 51.06, 49.08, 44.12.*

2D NMR of **1A**

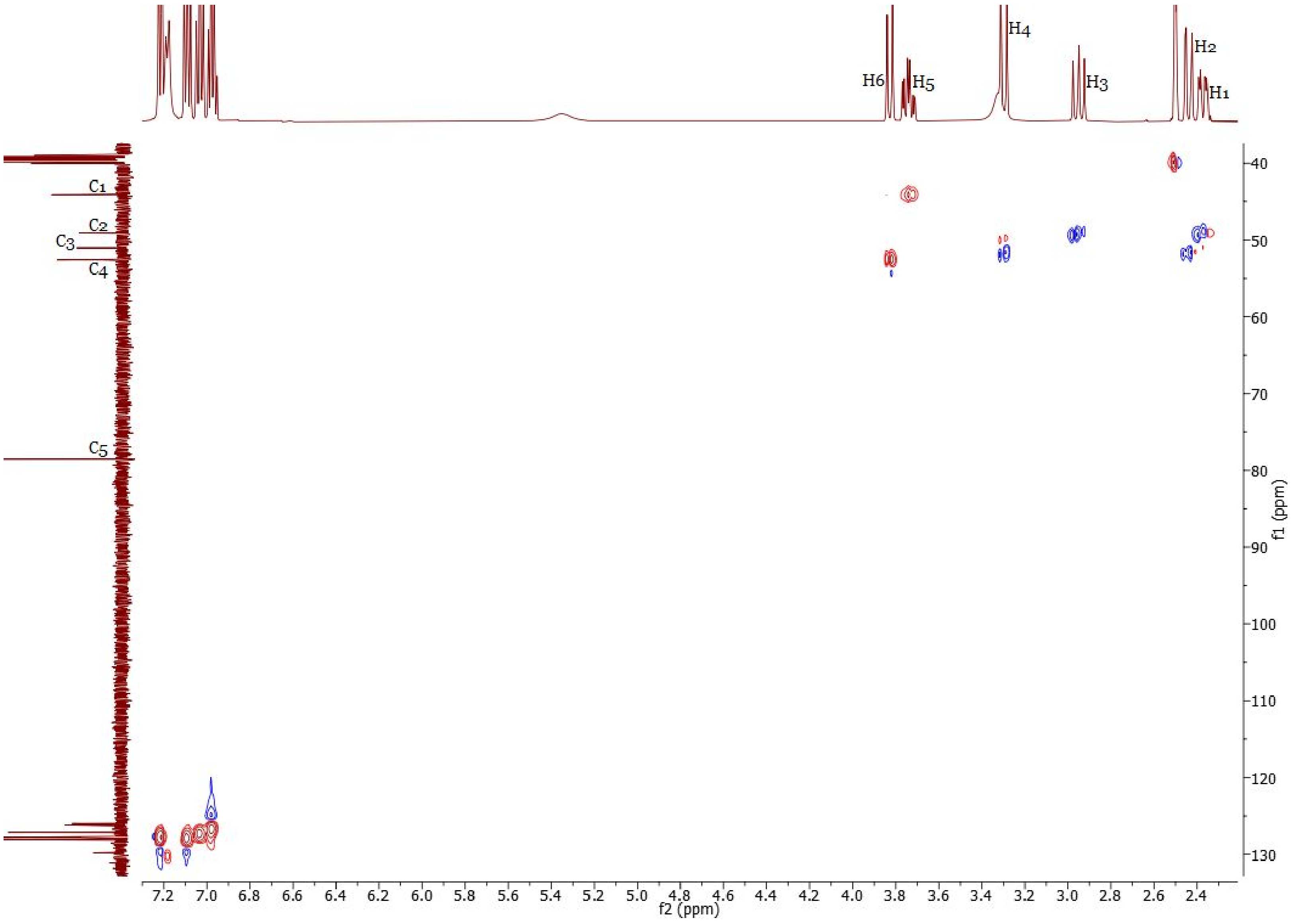

*Figure S23: HSQC spectrum of **1A**.*

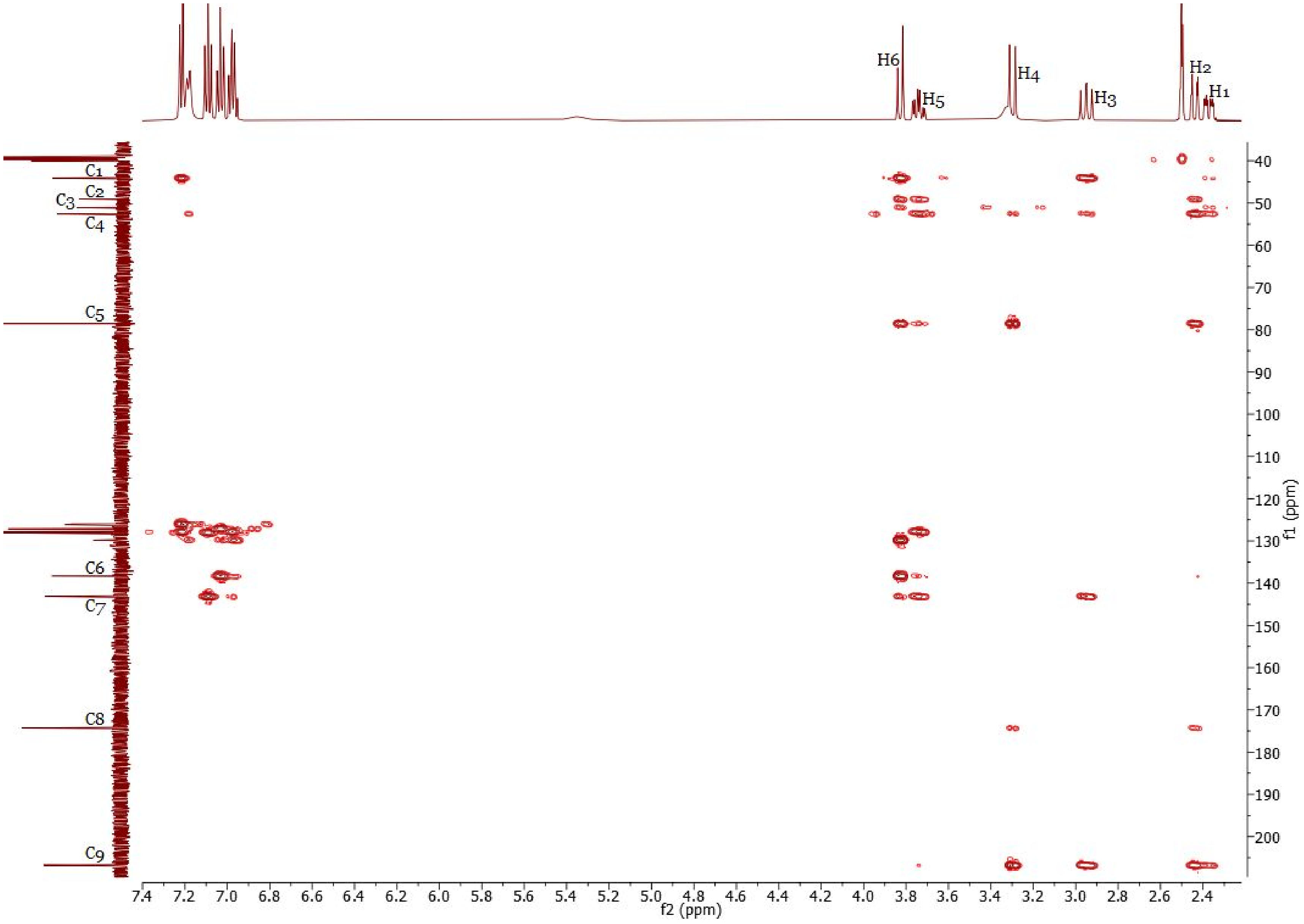

*Figure S24: HMBC spectrum of **1A**.*

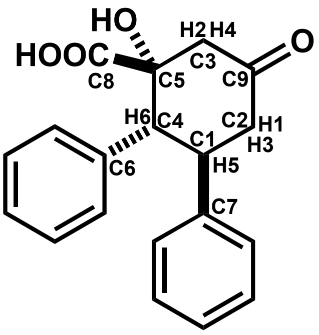

*Figure S25: 2D NMR observations of **1A**.*

2D NMR observations of **1A**:

Protons H1 and H3 are attached to carbon C2 forming CH_2 group. The group has connectivity to carbons C1, C3 (weak), C4, C7 and C9.

Protons H2 and H4 are attached to carbon C3 forming CH_2 group. The group has connectivity to carbons C2, C4, C5, C6 (very weak), C8 and C9.

Proton H5 is attached to carbon C1 forming CH group. The group has connectivity to carbons C2, C4, C5, C6 (weak), C7 and C9 (very weak). The group has connectivity to inside one of the phenyl groups, suggesting nearby location.

Proton H6 is attached to carbon C4 forming CH group. The group has connectivity to carbons C1, C2, C3, C5, C6, C7 (weak), C8 (very weak). The group has connectivity to inside one of the phenyl groups, suggesting nearby location.

IR spectroscopy of **1A**

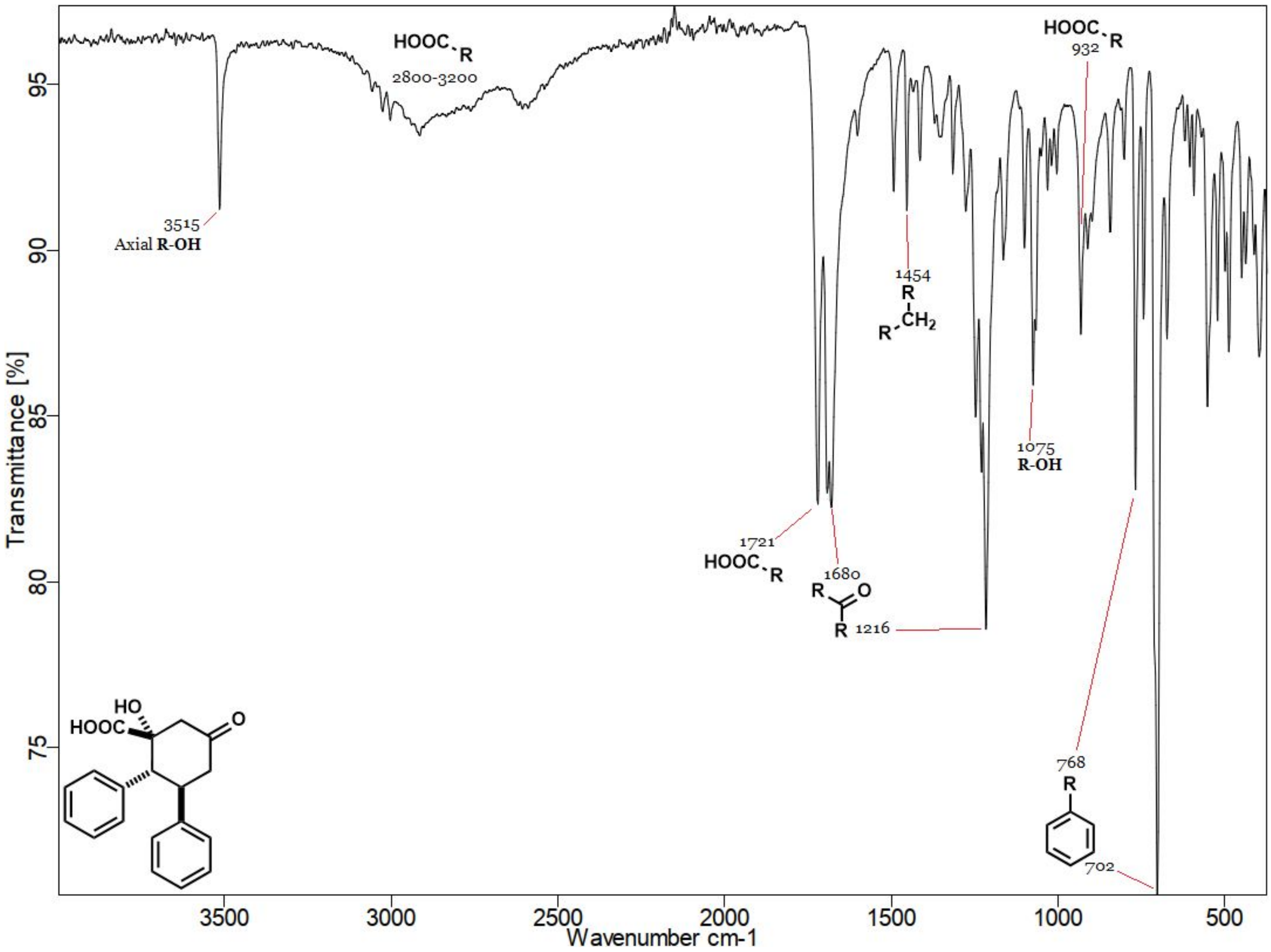

*Figure S26: IR spectrum of **1A** (3515 (m, sharp), 1075 (s) (Axial -OH), 2800-3200 (broad), 1721 (s), 932 (m) (R-COOH), 1680 (s), 1216 (s) (R-**CO**-R), 1454 (m) (R-CH_2-R), 768 (s), 702 (s) (5 adjacent H (Ph)) cm^{-1}).*

HRMS of **1A**

HRMS (ESI-TOF) m/z: [**1A**-H]$^-$ calculated for $C_{19}H_{17}O_4$ 309.1121; Found 309.1123; Error 0.482 ppm.

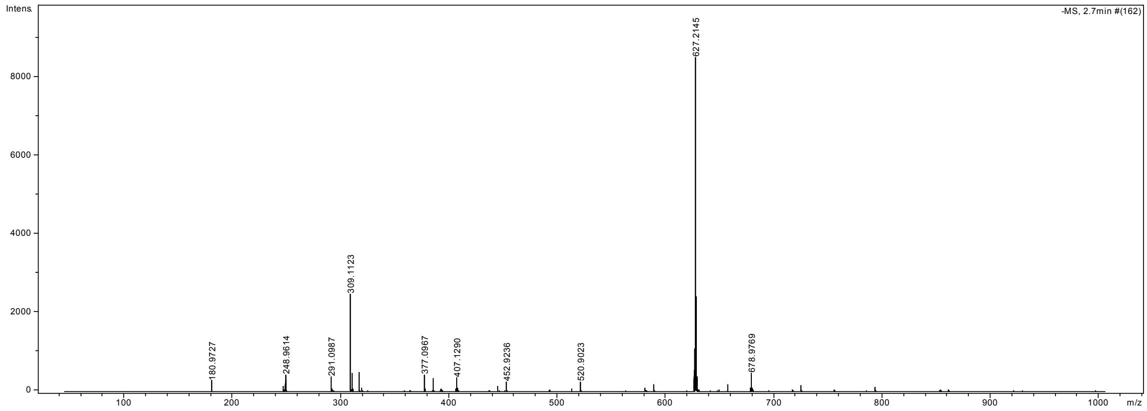

*Figure S27: ESI-TOF-MS of [**1A**-H]$^-$ (peak: 309.1123 m/z, negative-ion mode).*

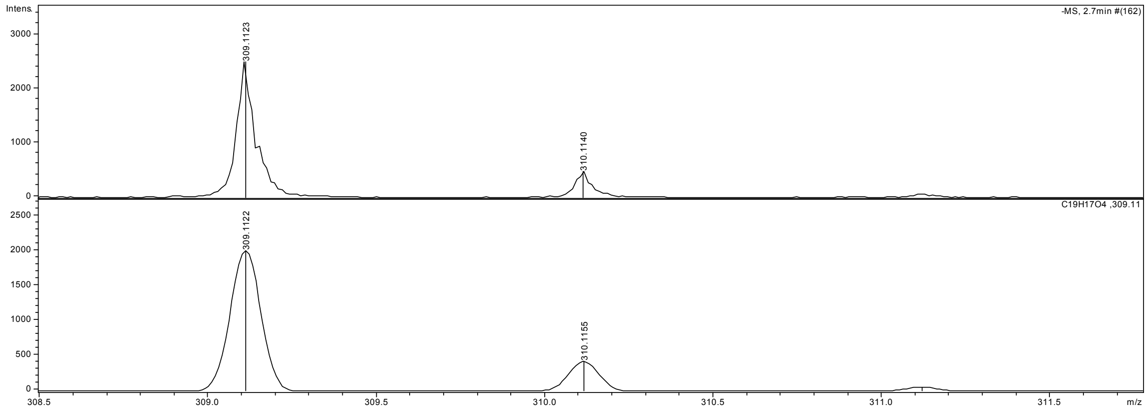

*Figure S28: Measured compound peak of [**1A**-H]$^-$ (309.1123 m/z) at top, simulated peak ($C_{19}H_{17}O_4$) below.*

3.4 Spectroscopic data of 2

1D NMR of **2**

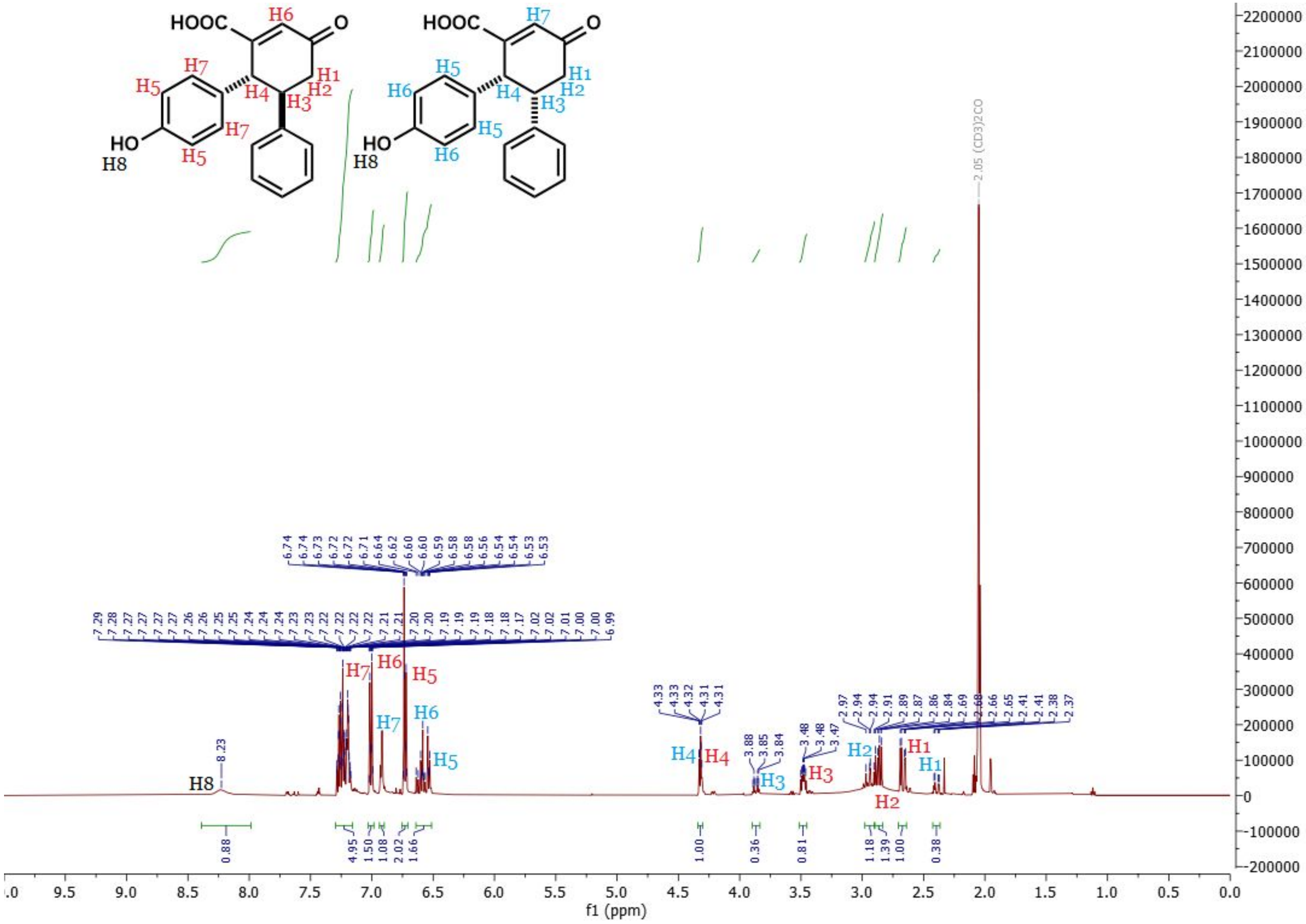

*Figure S29: ^{1}H NMR spectrum of **2** from full area. (500 MHz, acetone-d6): δ 8.23 (broad, 1H, phenolic OH.), 7.29-7.16 (m, 5H), 7.03-6.99 (m, 1.5H), 6.94-6.90 (m, 1H), 6.75-6.71 (m, 2H), 6.64-6.50 (m, 1.7H), 4.32 (m, 1H), 3.87 (dt, J = 15.0, 4.2 Hz, 0.36H), 3.48 (ddd, J = 8.9, 6.5, 4.6 Hz, 0.81H), 2.94 (dd, J = 17.0, 14.9 Hz, 1.2H), 2.87 (dd, J = 16.4, 8.9 Hz, 1.4H), 2.67 (dd, J = 16.5, 4.7 Hz, 1H). 2.39 (dd, J = 16.9, 3.7 Hz, 0.38H).*

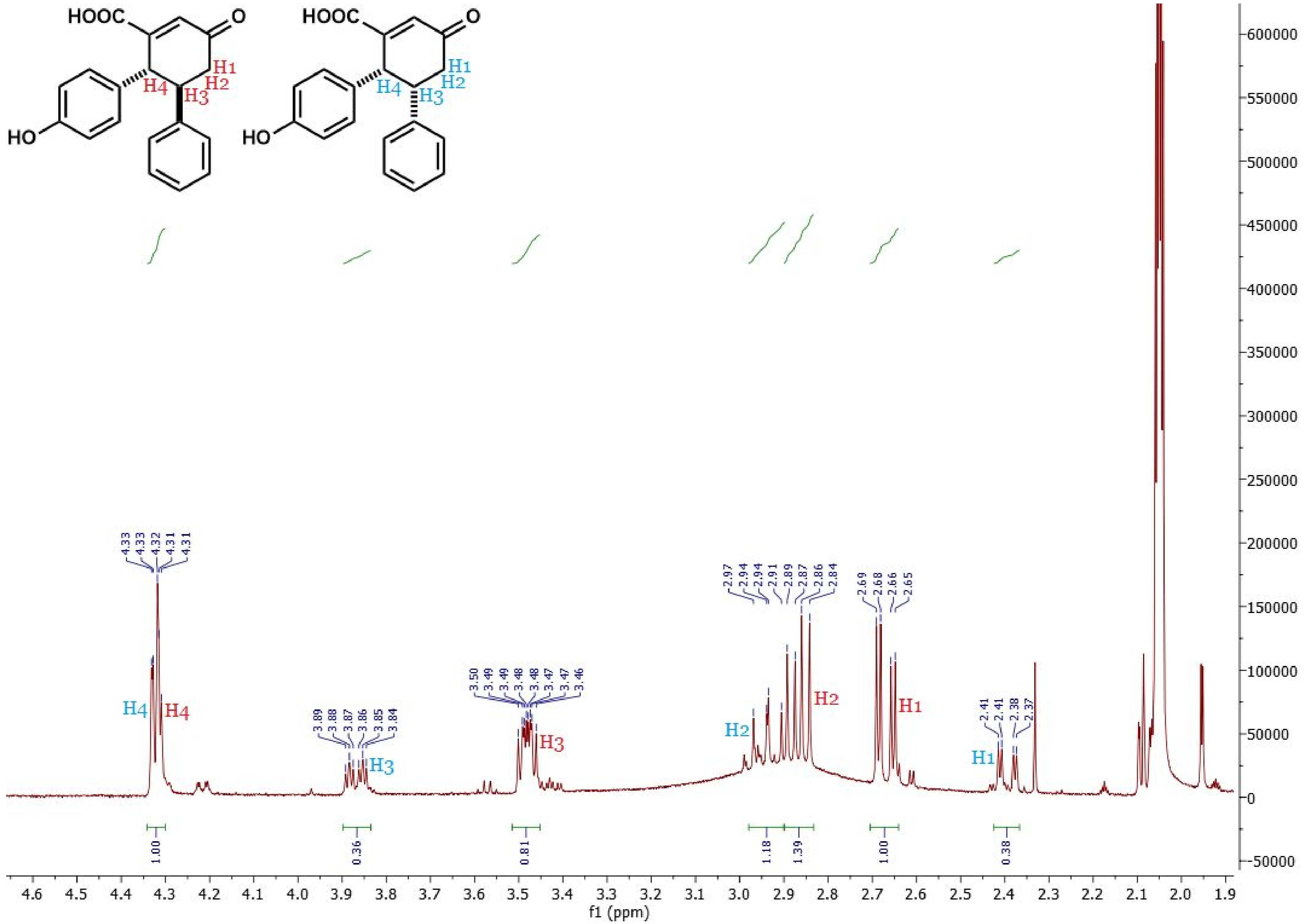

*Figure S30: ¹H NMR spectrum of **2** from aliphatic area. (500 MHz, acetone-d6): δ 4.32 (m, 1H), 3.87 (dt, J = 15.0, 4.2 Hz, 0.36H), 3.48 (ddd, J = 8.9, 6.5, 4.6 Hz, 0.81H), 2.94 (dd, J = 17.0, 14.9 Hz, 1.2H), 2.87 (dd, J = 16.4, 8.9 Hz, 1.4H), 2.67 (dd, J = 16.5, 4.7 Hz, 1H). 2.39 (dd, J = 16.9, 3.7 Hz, 0.38H).*

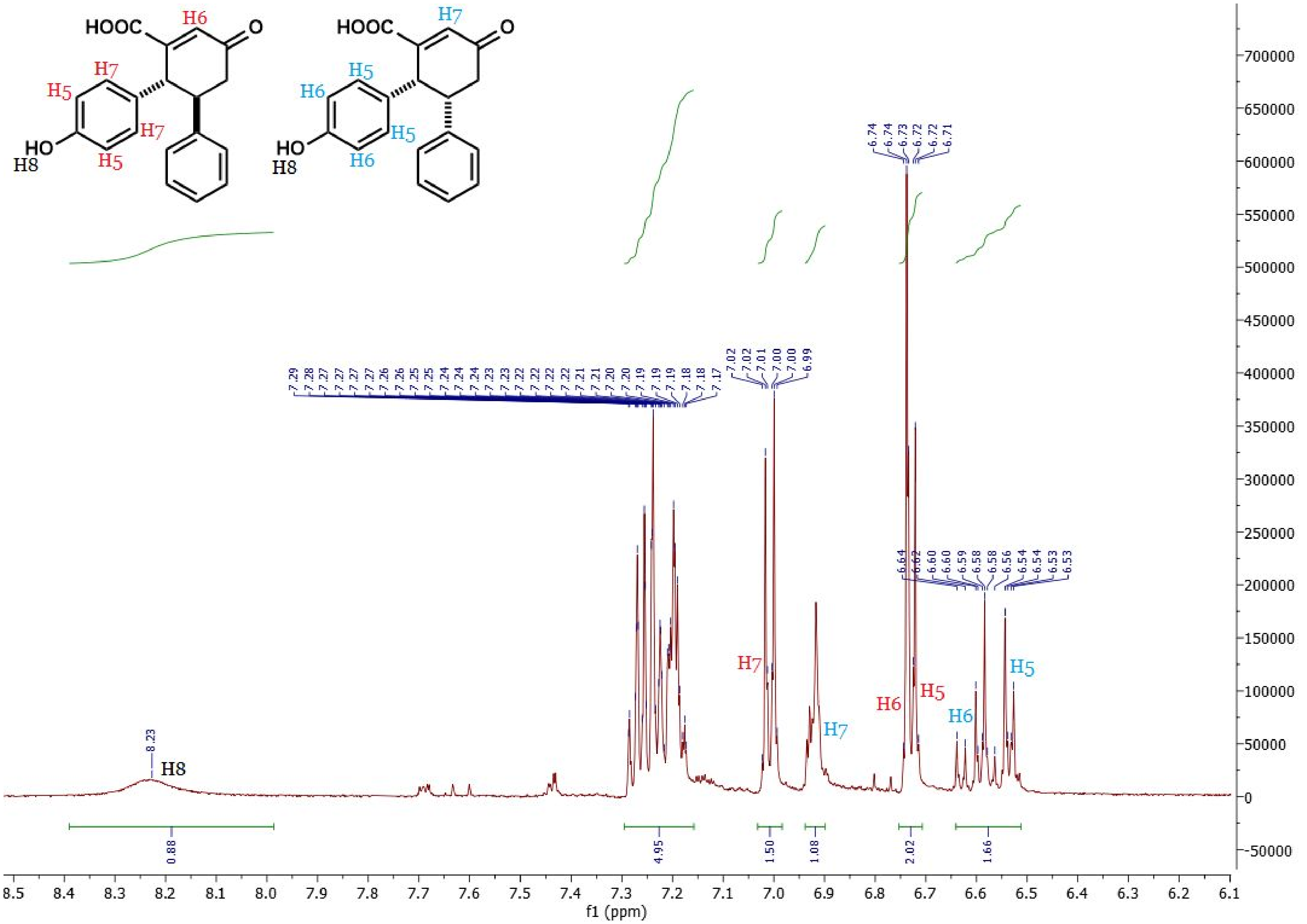

*Figure S31: ^{1}H NMR spectrum of **2** from aromatic area. (500 MHz, acetone-d6): δ8.23 (broad, 1H, phenolic OH.), 7.29-7.16 (m, 5H), 7.03-6.99 (m, 1.5H), 6.94-6.90 (m, 1H), 6.75-6.71 (m, 2H), 6.64-6.50 (m, 1.7H).*

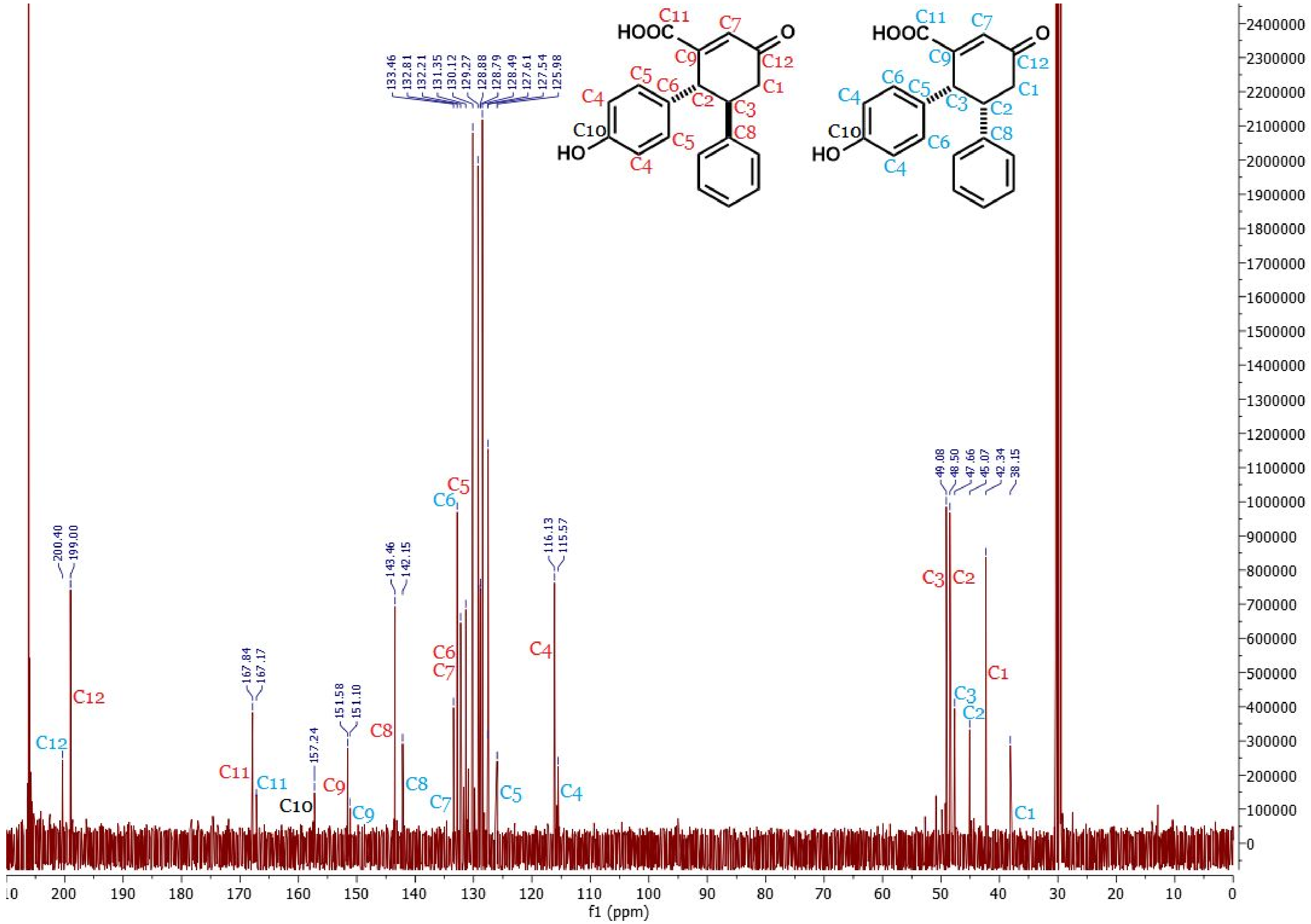

*Figure S32: $^{13}C\{^1H\}$ NMR spectra of **2** from full area. (125 MHz, acetone-d6): δ 200.40, 199.00, 167.84, 167.17, 157.24, 151.58, 151.10, 143.46, 142.15, 133.46, 132.81, 132.21, 131.35, 130.12, 129.27, 128.88, 128.79, 128.49, 127.61, 127.54, 125.98, 116.13, 115.57, 49.08, 48.50, 47.66, 45.07, 42.34, 38.15.*

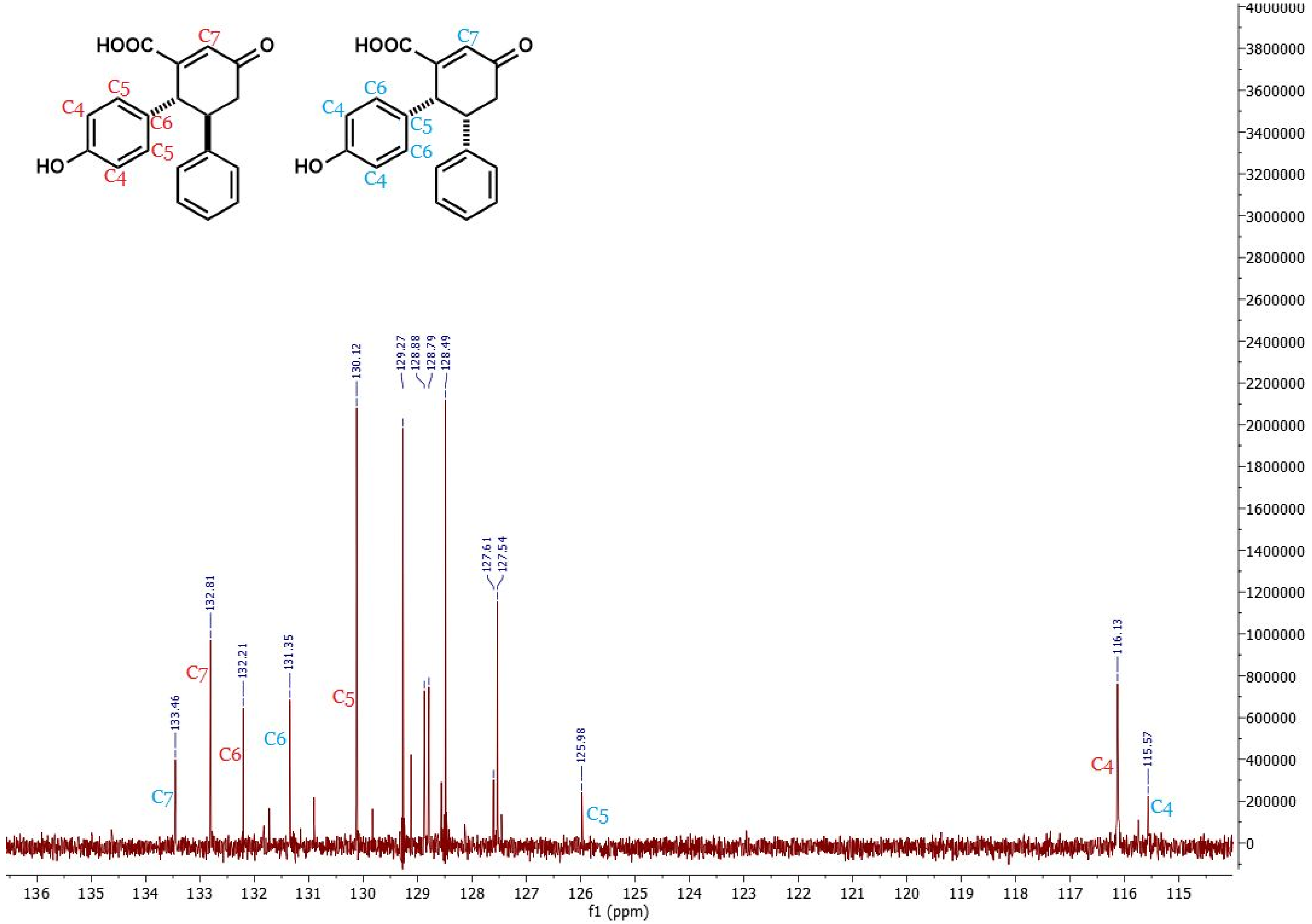

*Figure S33: $^{13}C\{^{1}H\}$ NMR spectra of **2** from aromatic area. (125 MHz, acetone-d6): δ 133.46, 132.81, 132.21, 131.35, 130.12, 129.27, 128.88, 128.79, 128.49, 127.61, 127.54, 125.98, 116.13, 115.57.*

2D NMR of **2**

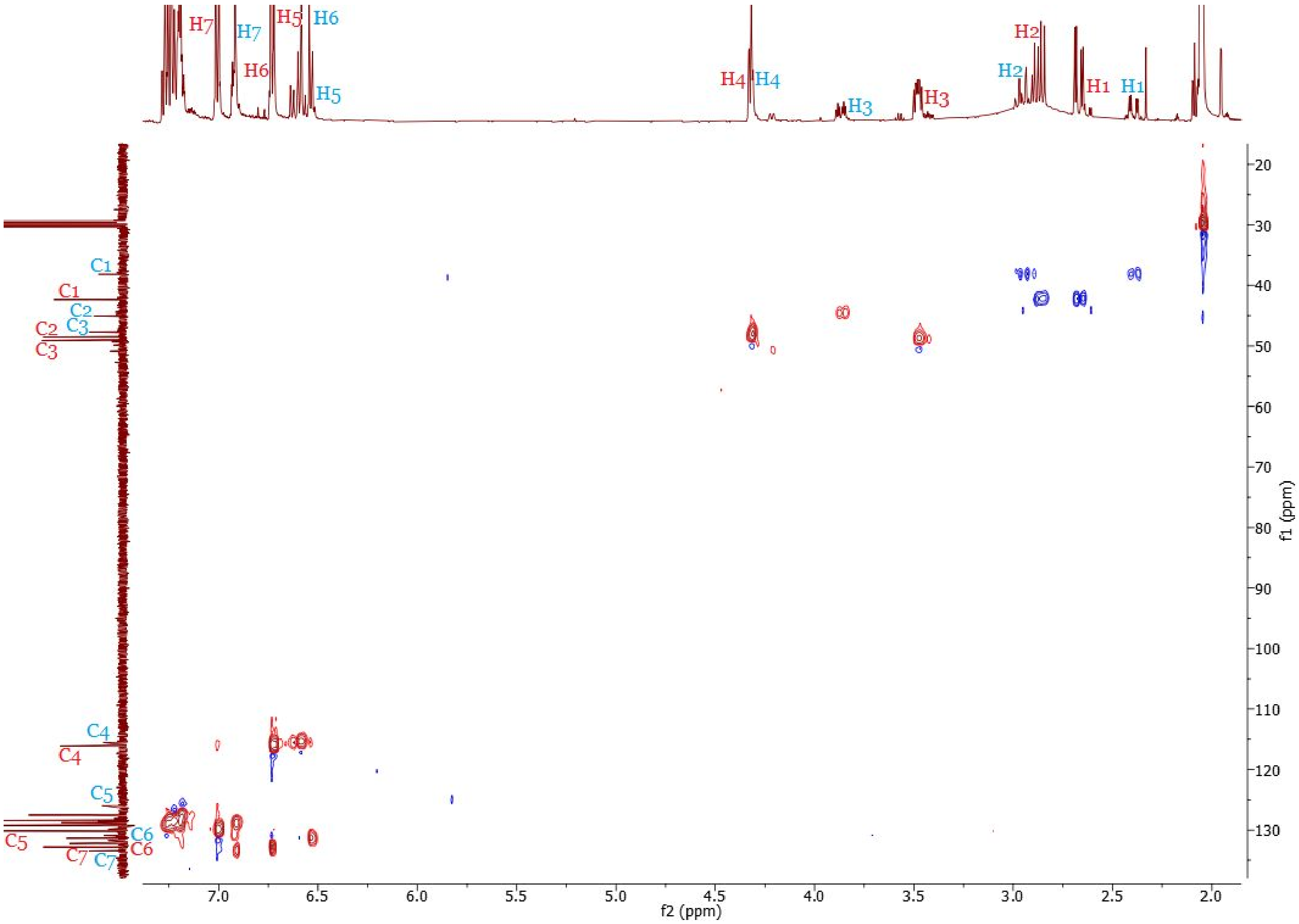

*Figure S34: HSQC spectrum of **2**.*

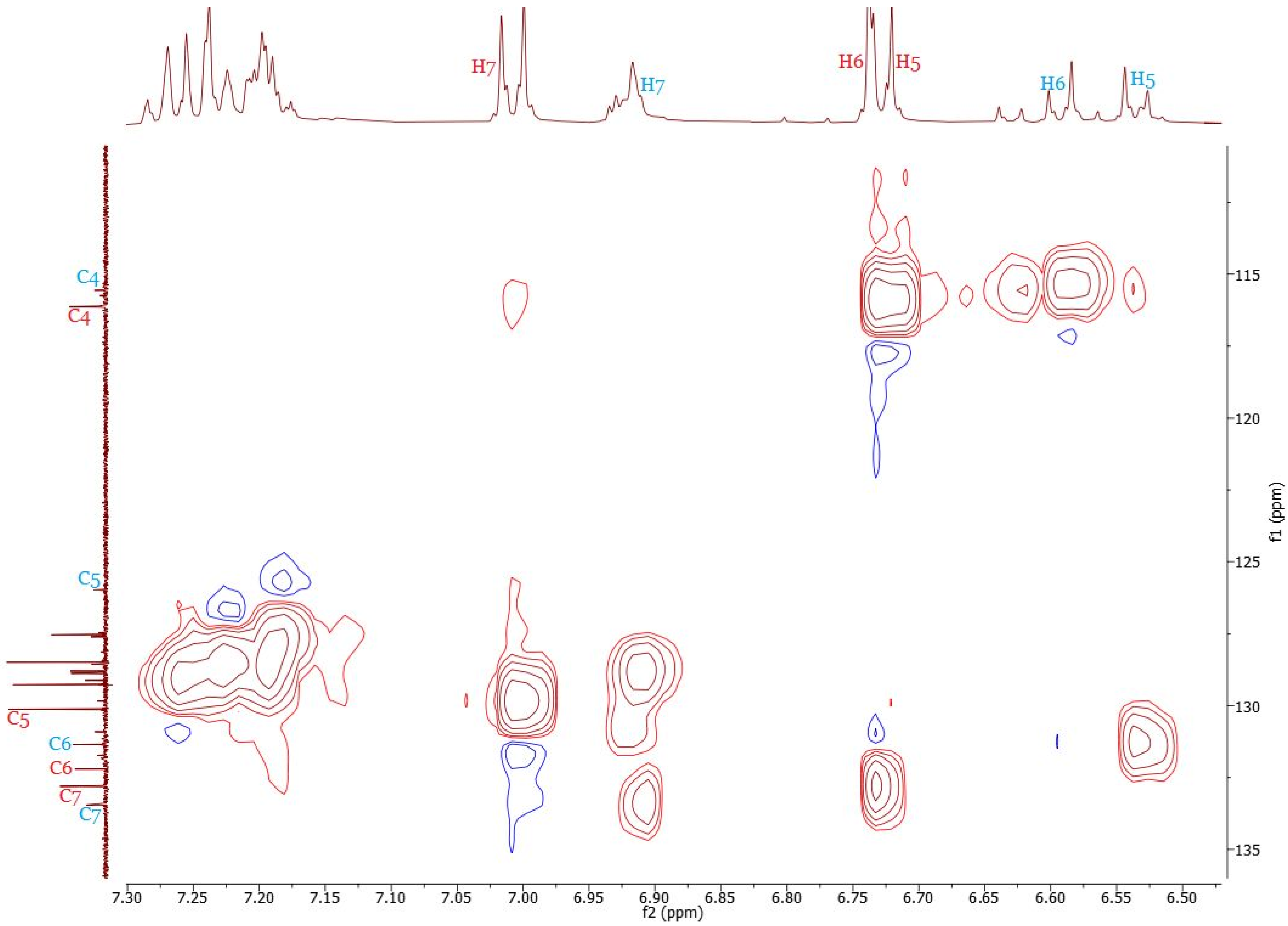

*Figure S35: HSQC spectrum of **2** from aromatic region.*

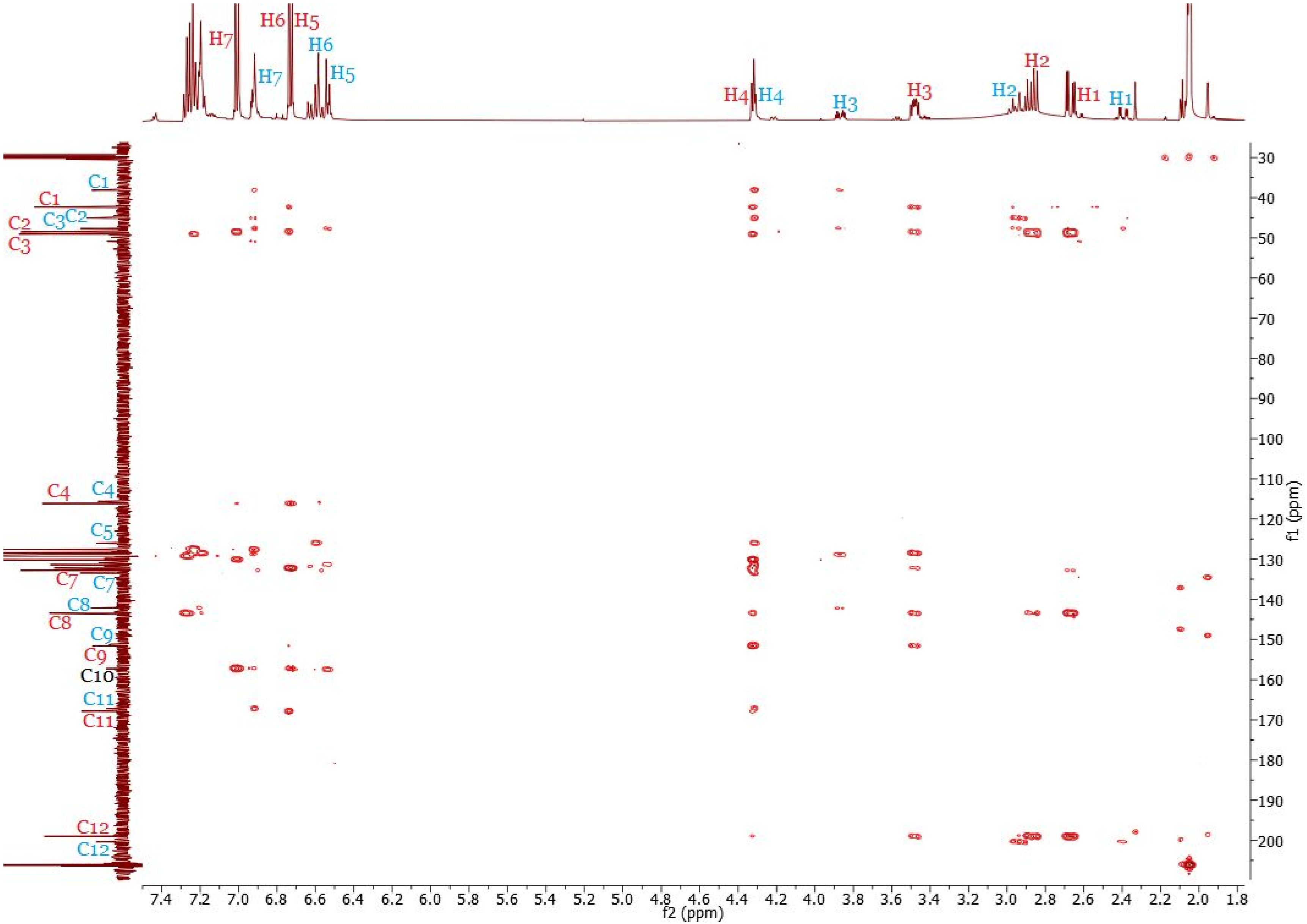

*Figure S36: HMBC spectrum of **2**.*

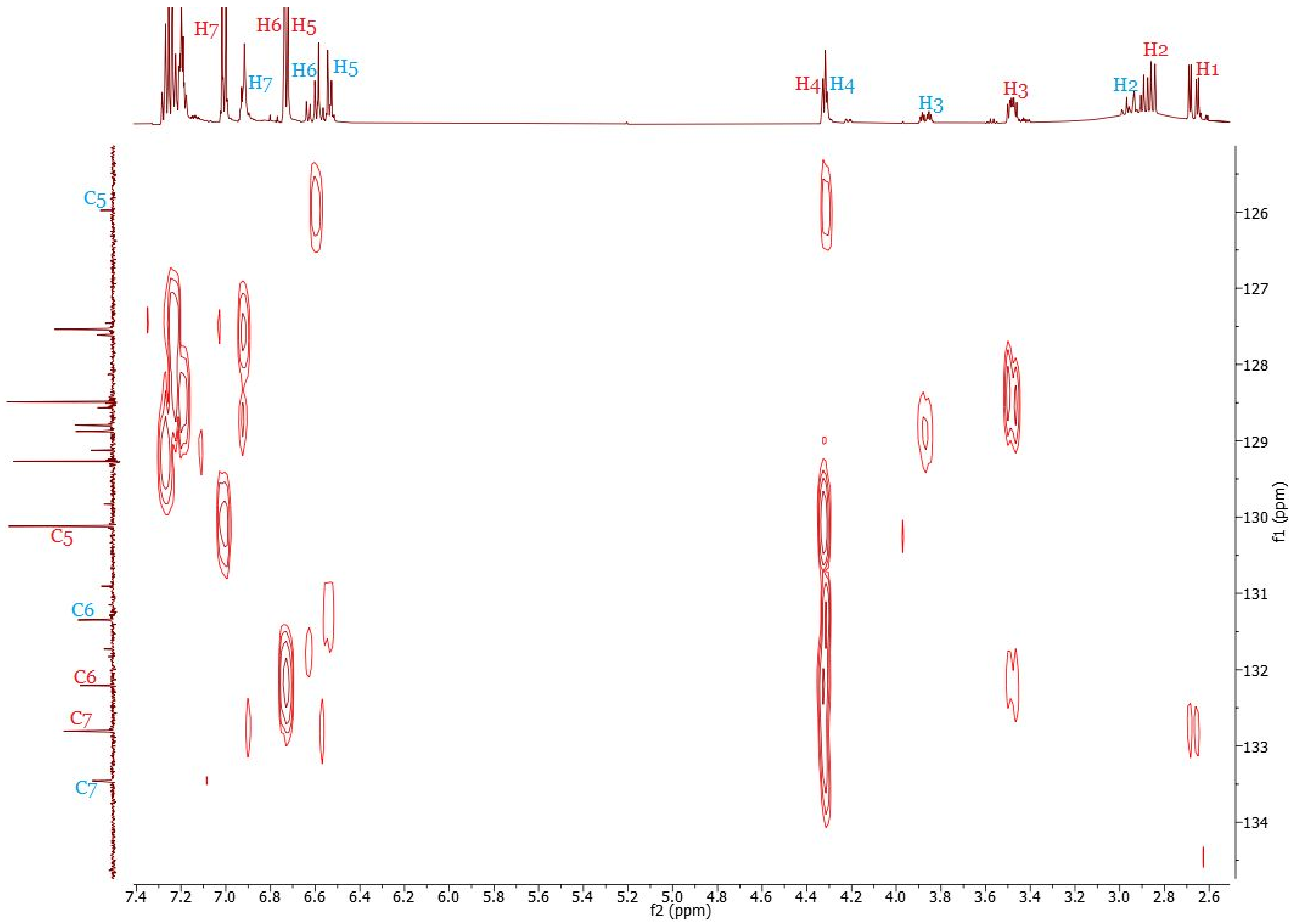

*Figure S37: HMBC spectrum of **2** from aromatic region.*

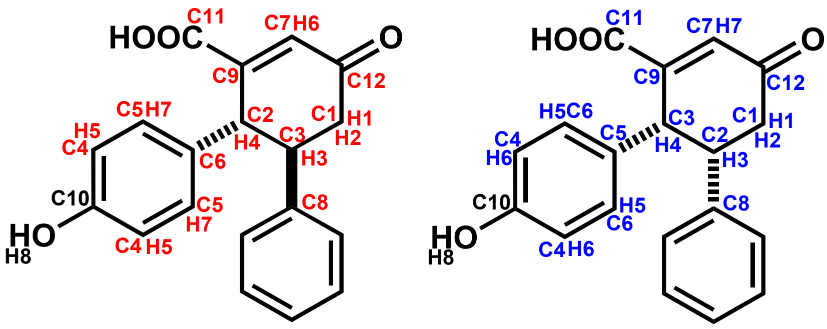

*Figure S38: 2D NMR observations of **2**.*

2D NMR observations:

RR/SS stereocenters:

Protons H1 and H2 are attached to carbon C1 forming CH_2 group. The group has connectivity to carbons C2, C3, C7 (weak) C8 and C12.

Proton H3 is attached to carbon C3 forming CH group. The group has connectivity to carbons C1, C2, C6 (weak), C8, C9 and C12. The group has connectivity inside phenyl group, suggesting nearby location.

Proton H4 is attached to carbon C2 forming CH group. The group has connectivity to carbons C1, C3, C5, C6, C8, C9, C11 and C12 (weak).

Protons H5 are attached to carbons C4 forming two CH groups. The groups have connectivity to carbons C4 (itself), C6 and C10.

Proton H6 is attached to carbon C7 forming CH group. The group has connectivity to carbons C1 (weak), C2, C9 (very weak) and C11.

Protons H7 are attached to carbons C5 forming two CH groups. The groups have connectivity to carbons C2, C5 (itself) and C10.

RS/SR stereocetners:

Protons H1 and H2 are attached to carbon C1 forming CH_2 group. The group has connectivity to carbons C3, C2 and C12.

Proton H3 is attached to carbon C2 forming CH group. The group has connectivity to carbons C1, C3 and C8. The group has connectivity inside a phenyl group, suggesting nearby location.

Proton H4 is attached to carbon C3 forming CH group. The group has connectivity to carbons C1, C2, C5, C6 and C11.

Protons H5 are attached to carbons C6 forming two CH groups. The groups have connectivity to carbons C3, C6 (itself) and C10.

Protons H6 are attached to carbons C4 forming two CH groups. The groups have connectivity to carbons C4 (itself), C5 and C10.

Proton H7 is attached to carbon C7 forming CH group. The group has connectivity to carbons C3 and C11.

IR spectroscopy of **2**

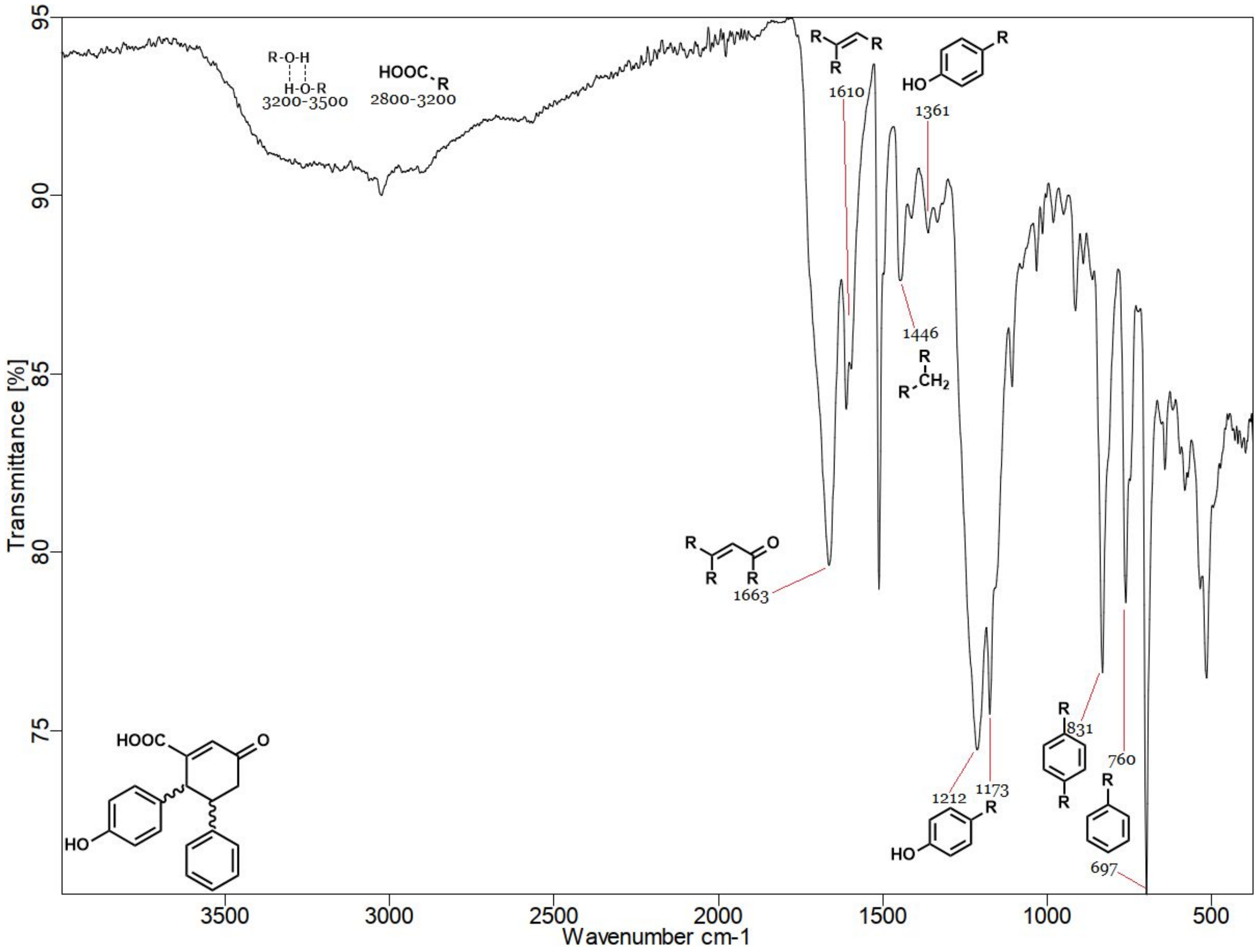

Figure S39: IR spectrum of ***2*** *(2700-3200 (broad) (R-COOH), 3200-3500 (broad) (intermolecular hydrogen bonds), 1663 (s) (C=C-**CO**-R), 1610 (m) (R_2-C=**CH**-R), 1446 (m) (R-**CH_2**-R), 1361 (w), 1212 (s) (**OH**-Ph), 831 (s) (2 adjacent H (Ph)), 760 (s), 697 (s) (5 adjacent H (Ph)) cm^{-1}).*

HRMS of **2**

HRMS (ESI-TOF) m/z: [**2**-H]$^-$ calculated for $C_{19}H_{15}O_4$ 307.0965; Found 307.0962; Error 0.945 ppm.

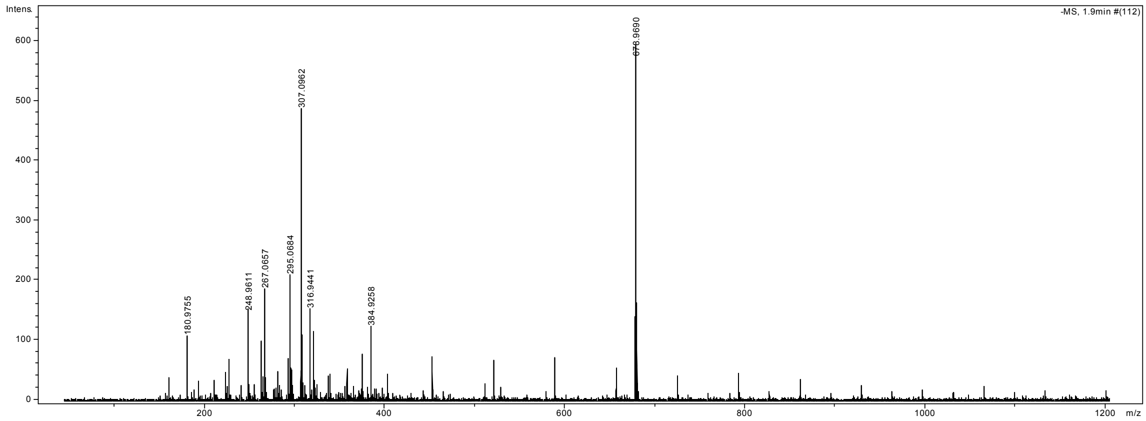

*Figure S40: ESI-TOF-MS of [**2**-H]$^-$ (peak: 307.0962 m/z, negative-ion mode).*

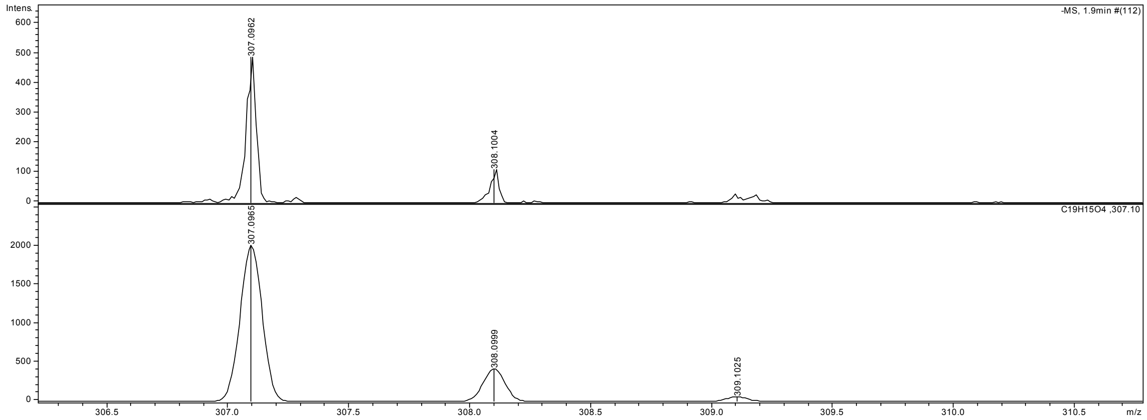

*Figure S41: Measured compound peak of [**2**-H]$^-$ (307.0962 m/z) at top, simulated peak ($C_{19}H_{15}O_4$) below.*

3.5 Spectroscopic data of 3

1D NMR of **3**

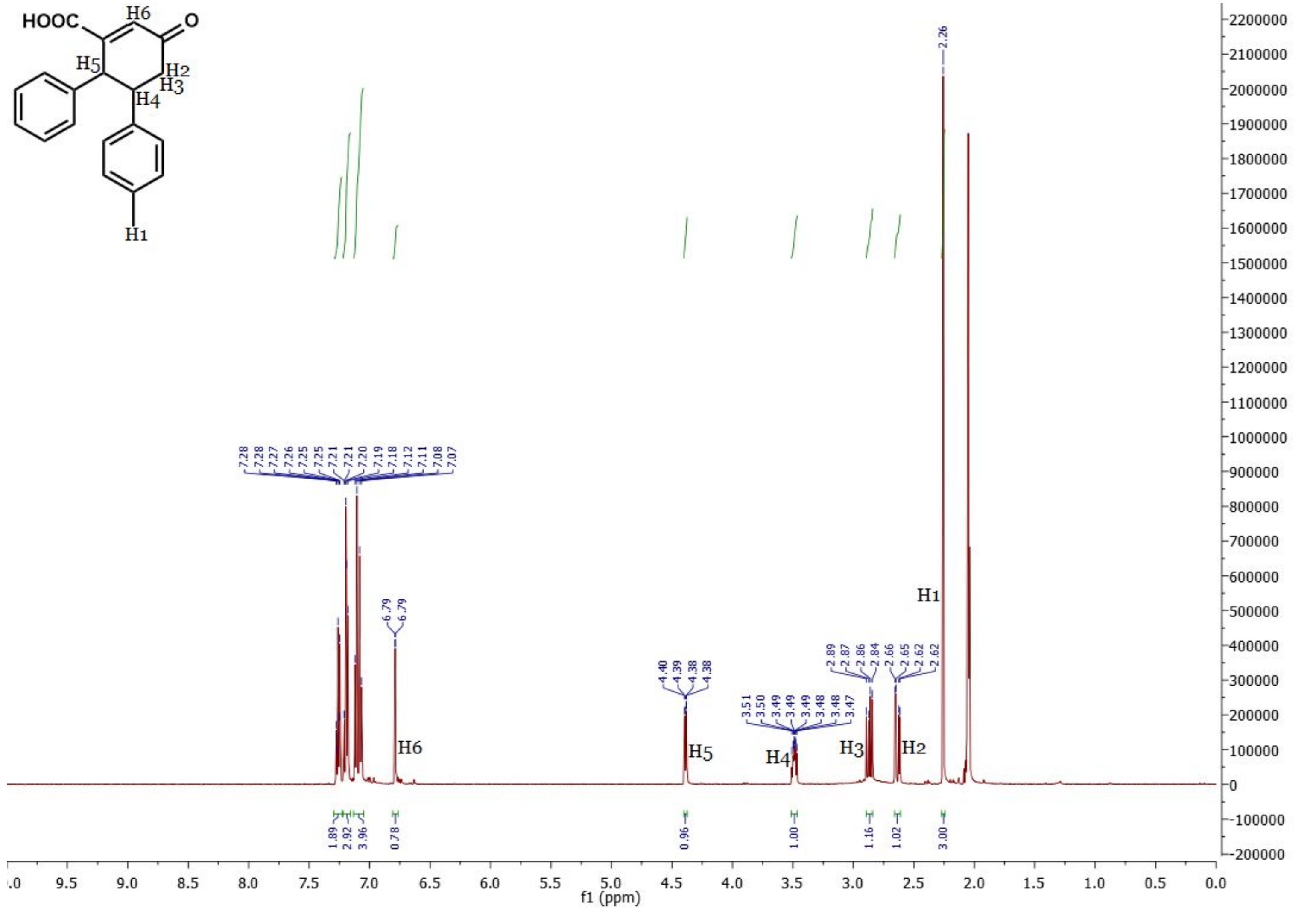

*Figure S42: ^{1}H NMR spectrum of **3** (500 MHz, acetone-d6): δ 7.28 - 7.24 (m, 2H), 7.22 – 7.17 (m, 3H), 7.13 – 7.06 (m, 4H), 6.79 (d, J = 1.8 Hz, 1H), 4.39 (dd, J = 6.6, 1.6 Hz, 1H), 3.49 (ddd, J = 9.0, 6.6, 4.7 Hz, 1H), 2.87 (dd, J = 16.4, 9.1 Hz, 1H), 2.64 (dd, J = 16.4, 4.6 Hz, 1H), 2.26 (s, 3H).*

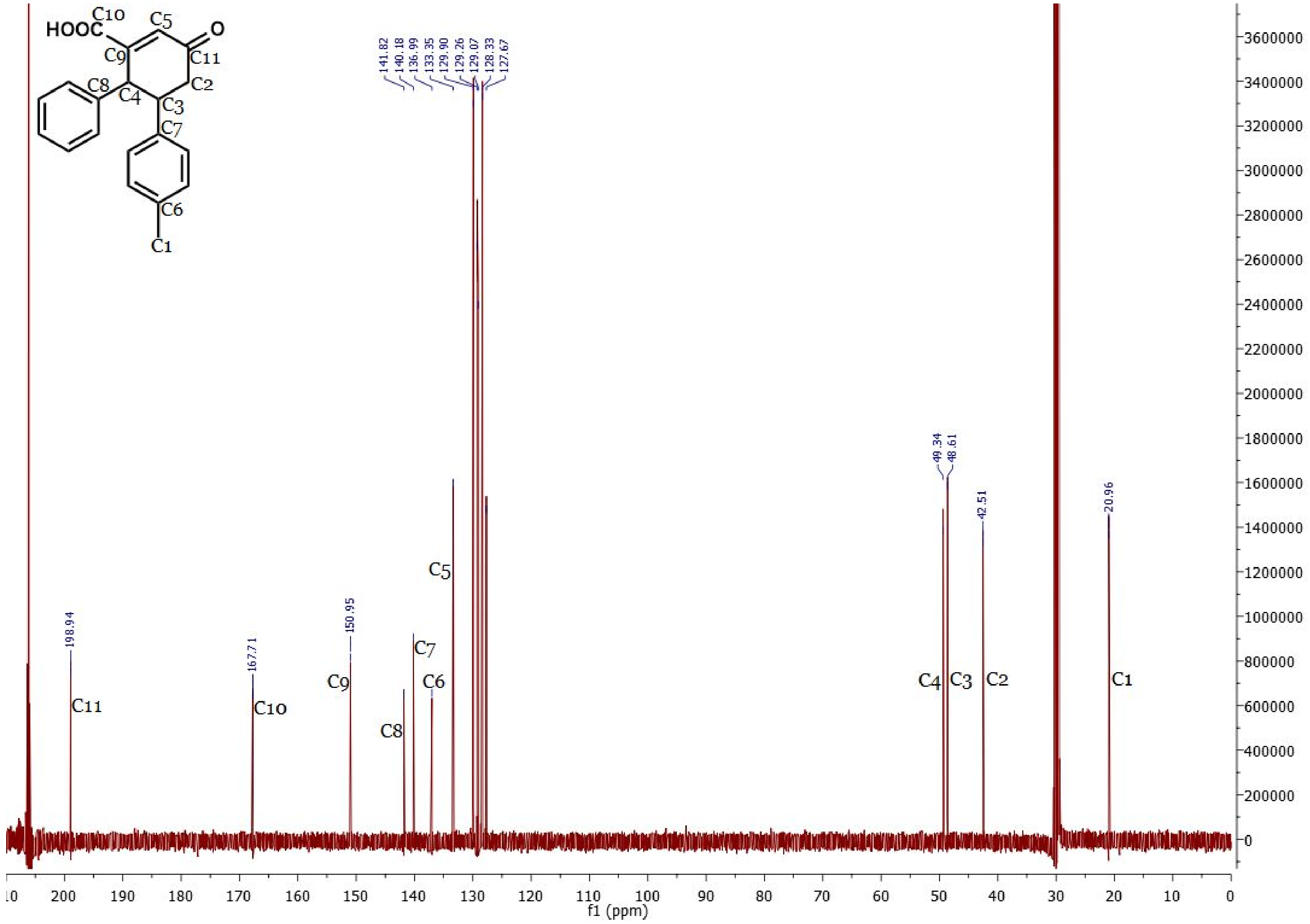

*Figure S43: $^{13}C\{^{1}H\}$ NMR spectrum of **3** (125 MHz, acetone-d6): δ 198.94, 167.71, 150.95, 141.82, 140.18, 136.99, 133.35, 129.90, 129.26, 129.07, 128.33, 127.67, 49.34, 48.61, 42.51, 20.96.*

2D NMR of **3**

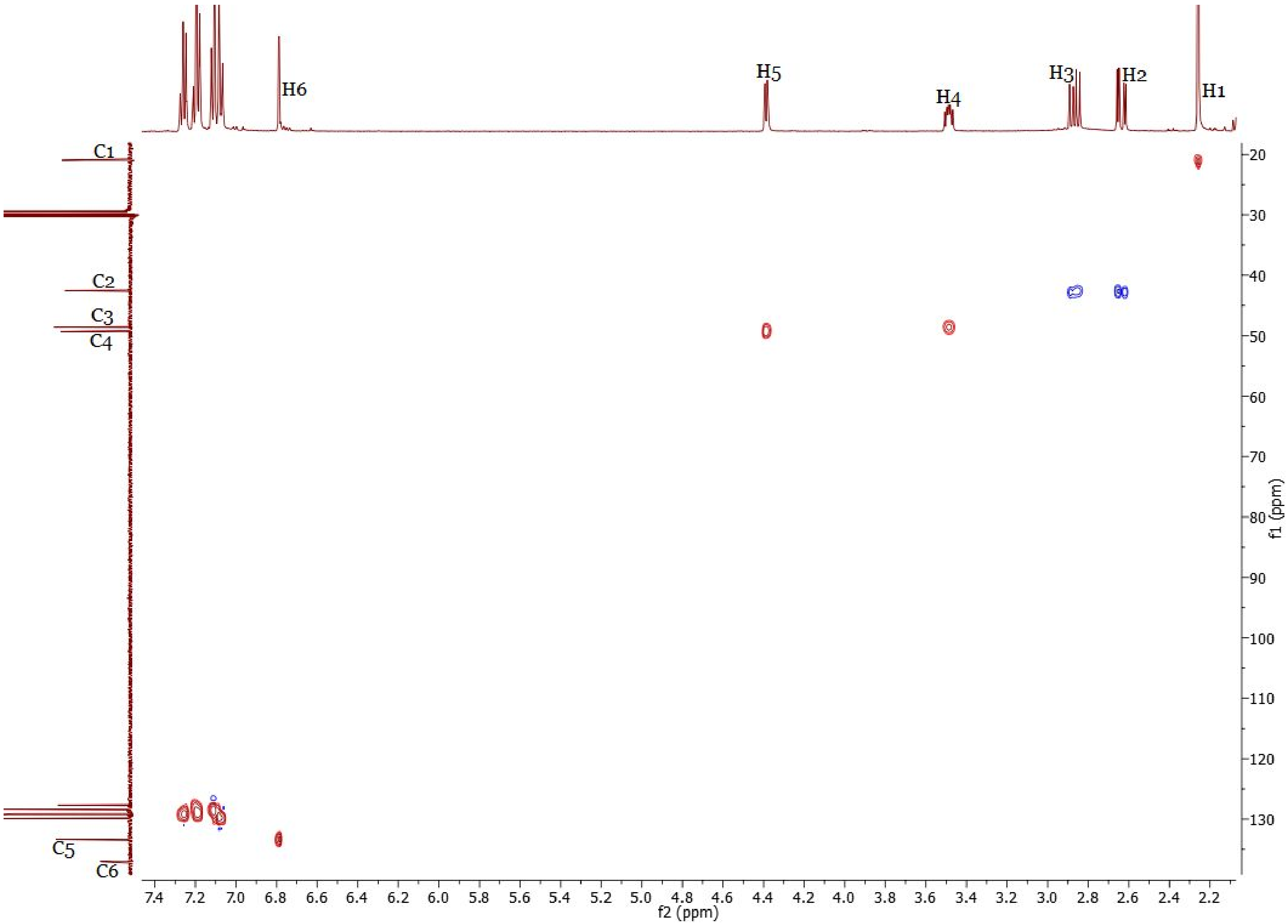

*Figure S44: HSQC spectrum of **3**.*

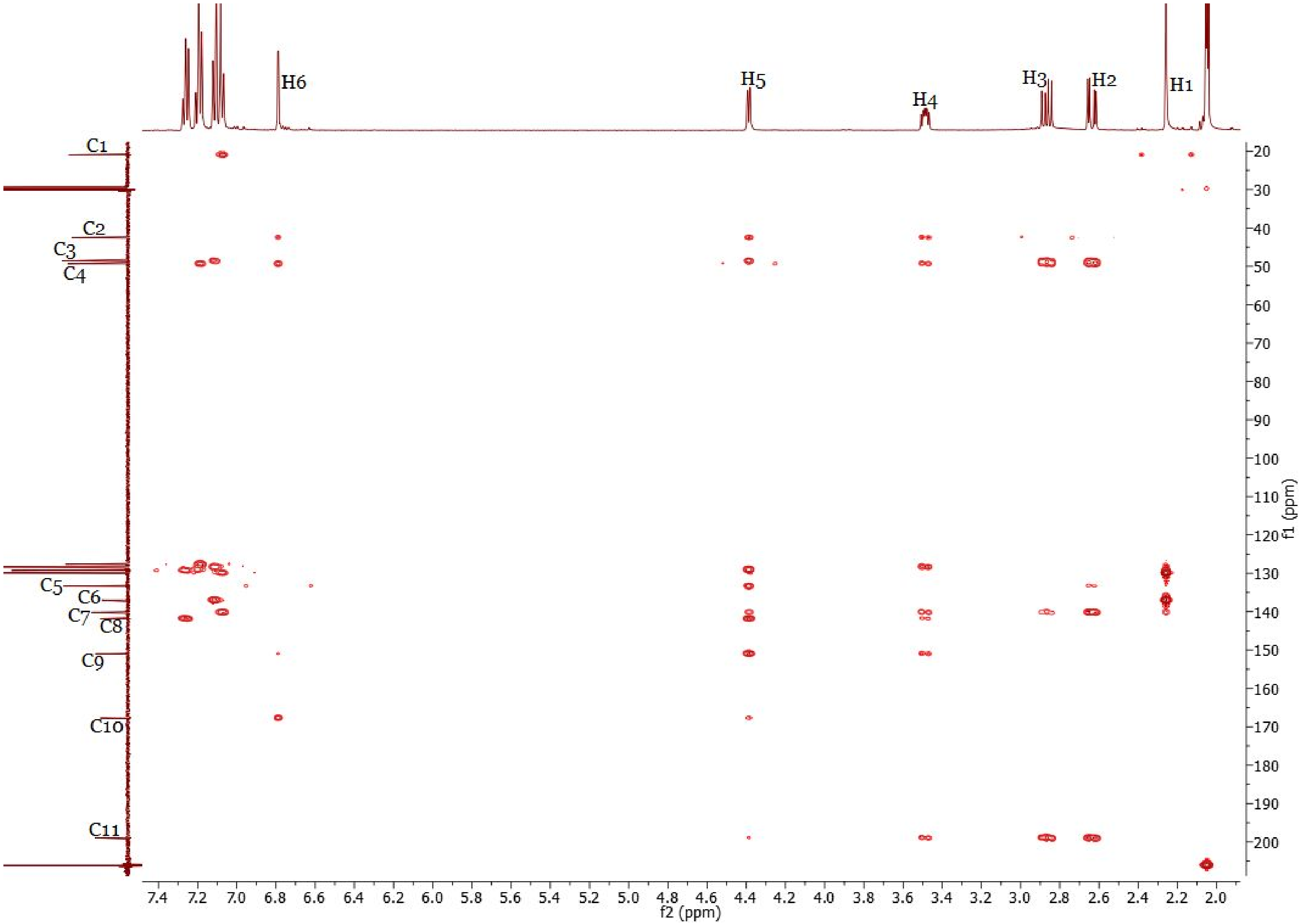

*Figure S45: HMBC spectrum of **3**.*

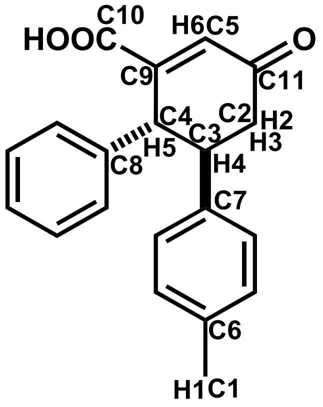

*Figure S46: 2D NMR observations of **3**.*

2D NMR observations of **3**:

Protons H1 are attached to carbon C1 forming CH_3 group. The group has connectivity to carbons C6 and C7 (weak). The group also has connectivity inside the phenyl ring.

Protons H2 and H3 are attached to carbon C2 forming CH_2 group. The group has connectivity to carbons C3, C4, C7 and C11. (Proton H2 has weak connectivity to carbon C5)

Proton H4 is attached to carbon C3 forming CH group. The group has connectivity to carbons C2, C4, C7, C8 (weak), C9 and C11. The group has connectivity inside the methylated phenyl group.

Proton H5 is attached to carbon C4 forming CH group. The group has connectivity to carbons C2, C3, C5, C7, C8, C9, C10 (weak) and C11 (weak).

Proton H6 is attached to carbon C5 forming CH group. The group has connectivity to carbons C2, C4, C9 (weak) and C10.

IR spectroscopy of **3**

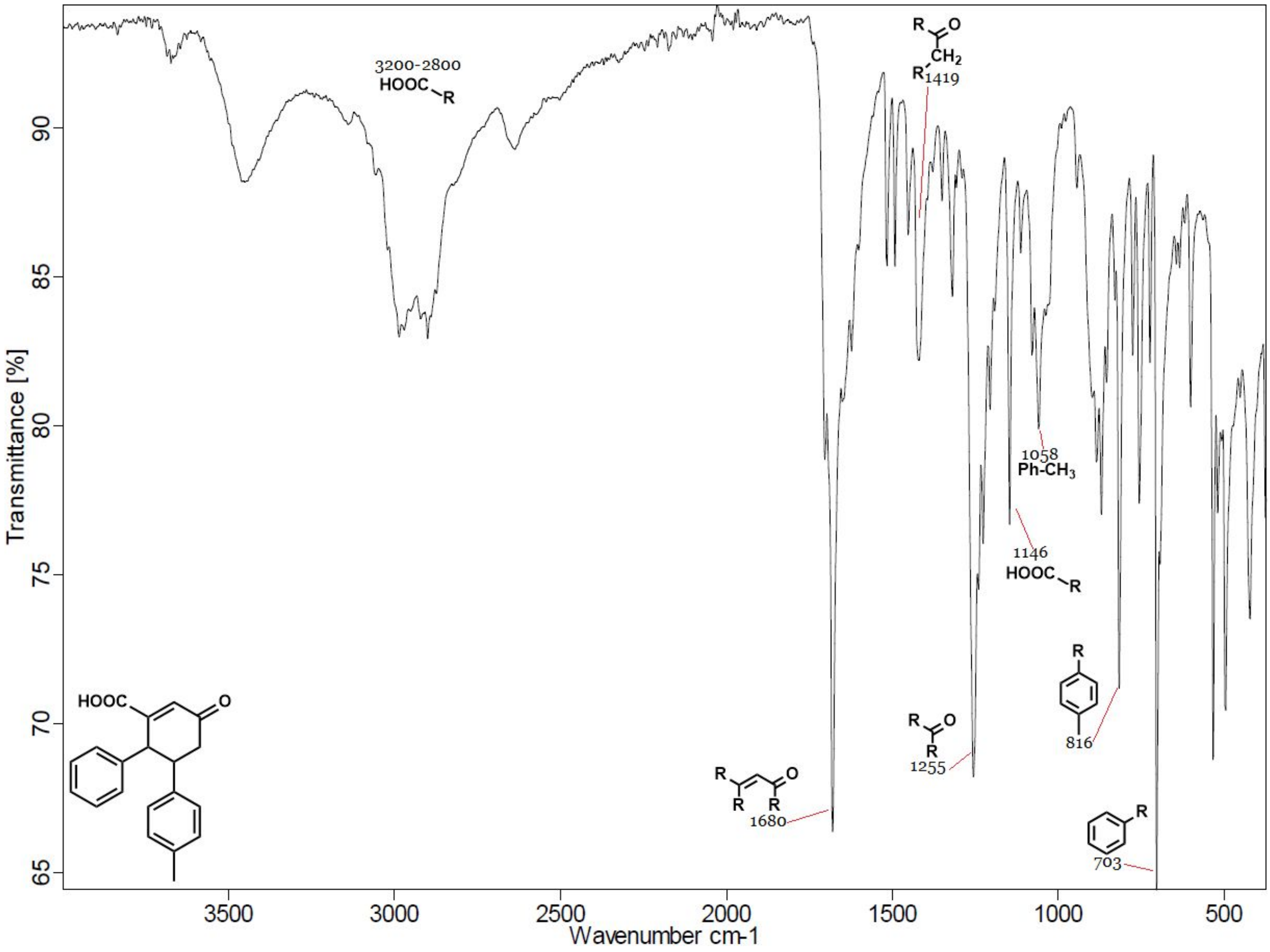

*Figure S47: IR spectrum of **3** (2800-3200 (broad) (R-COOH), 1680 (s) (C=C-**CO**-R), 1419 (m) (R-CO-**CH_2**-R), 1255 (s) (R-**CO**-R), 1146 (m) (R-**CO**OH), 1058 (m) (CH_3-Ph), 816 (s) (2 adjacent H (R-Ph-p-Me)), 703 (s) (5 adjacent H (Ph)) cm^{-1})*

HRMS of **3**

HRMS (ESI-TOF) m/z: [**3**-H]⁻ calculated for $C_{20}H_{17}O_3$ 305.1172; Found 305.1170; Error 0.680 ppm.

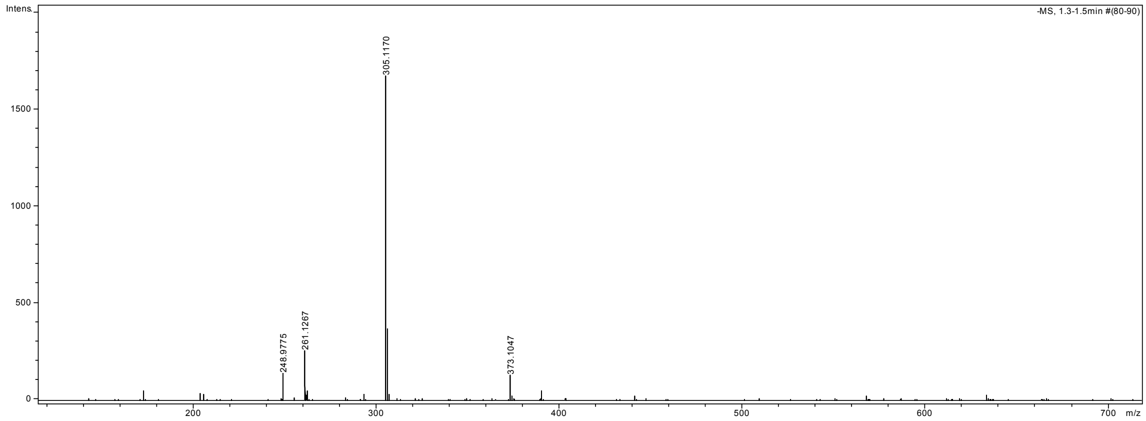

*Figure S48: ESI-TOF-MS of [**3**-H]⁻ (peak: 305.1170 m/z, negative-ion mode).*

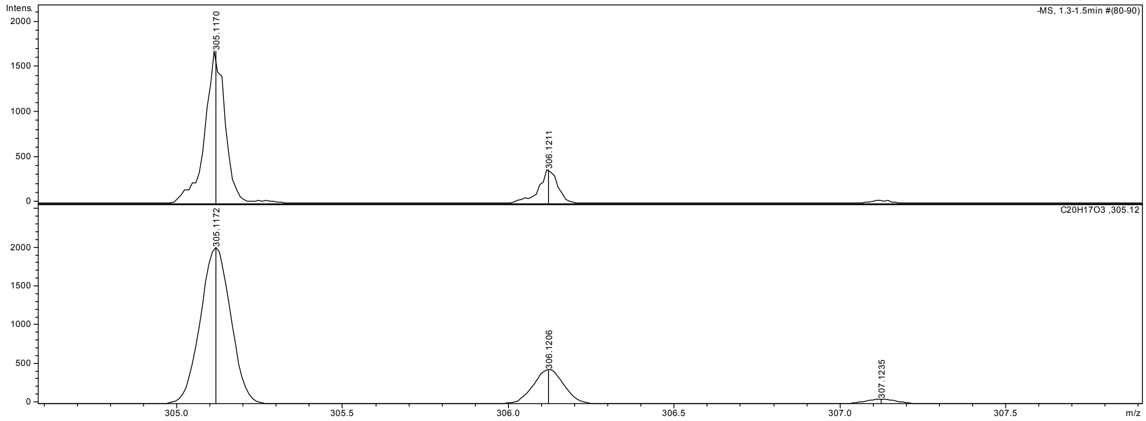

*Figure S49: Measured compound peak of [**3**-H]⁻ (305.1170 m/z) at top, simulated peak ($C_{20}H_{17}O_3$) below.*

3.6 Spectroscopic data of 4

1D NMR of **4**

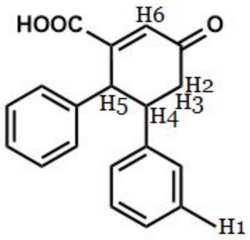

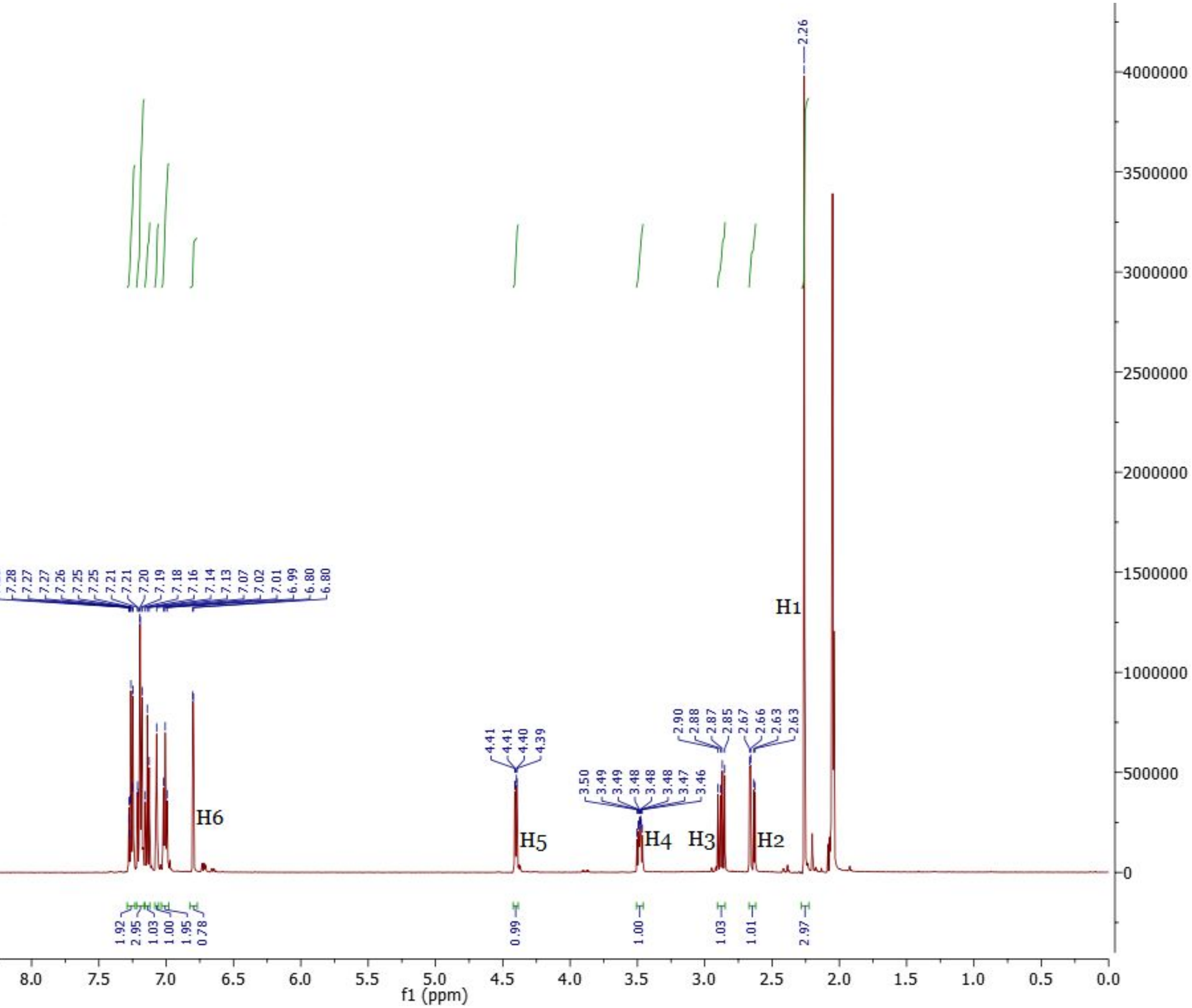

*Figure S50: ^{1}H NMR spectrum of **4** (500 MHz, acetone-d6): δ 7.29-7.23 (m, 2H), 7.22-7.17 (m, 3H), 7.16-7.12 (m, 1H), 7.07 (s, 1H), 7.07-6.98 (m, 2H), 6.80 (d, J = 1.8 Hz, 1H), 4.40 (dd, J = 6.6, 1.7 Hz, 1H), 3.48 (ddd, J = 9.0, 6.6, 4.7 Hz, 1H), 2.88 (dd, J = 16.4, 9.0 Hz, 1H), 2.65 (dd, J = 16.5, 4.6 Hz, 1H), 2.26 (s, 3H).*

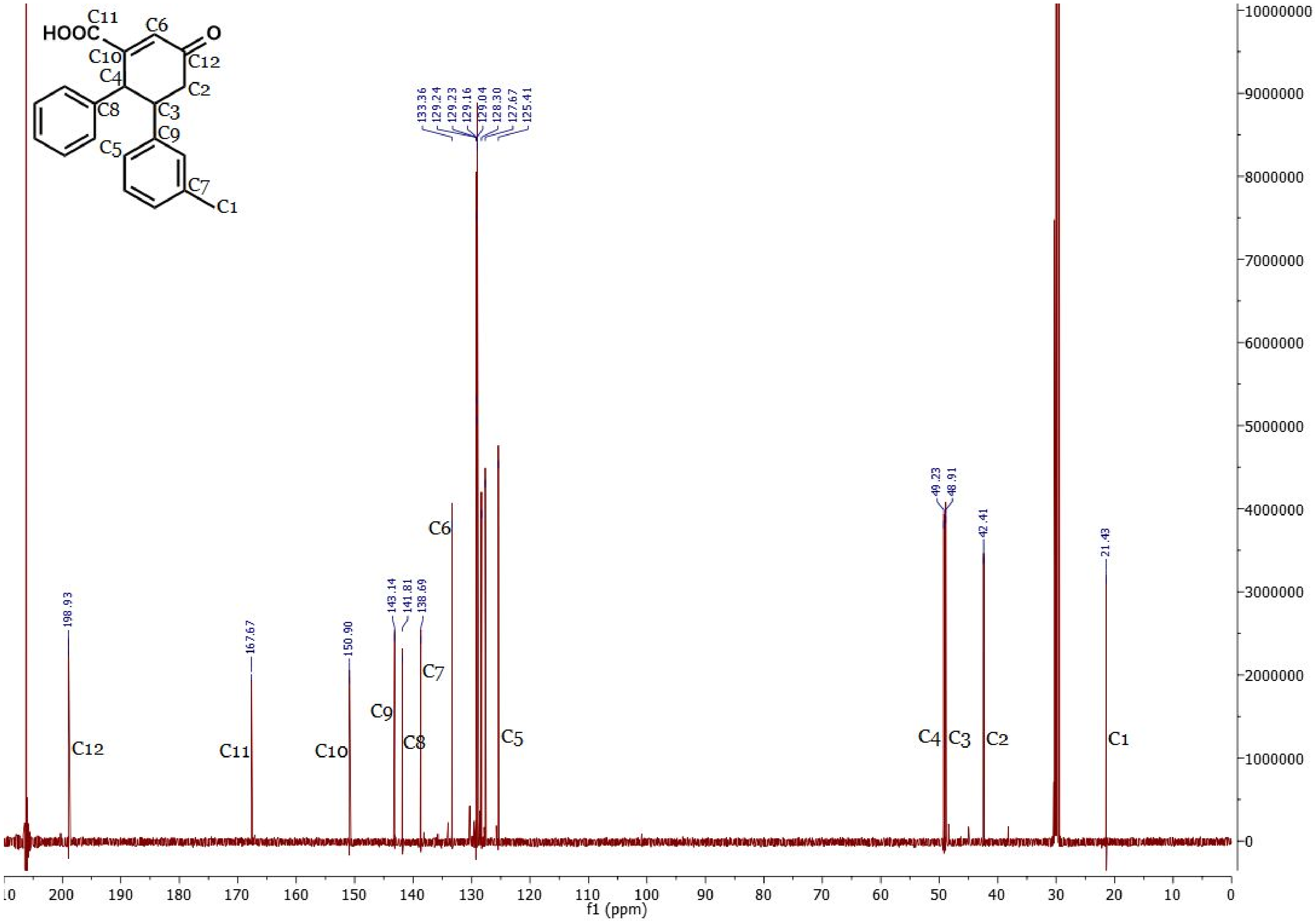

*Figure S51: $^{13}C\{^{1}H\}$ NMR spectrum of **4** (125 MHz, acetone-d6): δ 198.93, 167.67, 150.90, 143.14, 141.81, 138.69, 133.36, 129.24, 129.23, 129.16, 129.04, 128.30, 127.67, 125.41, 49.23, 48.91, 42.41, 21.43.*

2D NMR of **4**

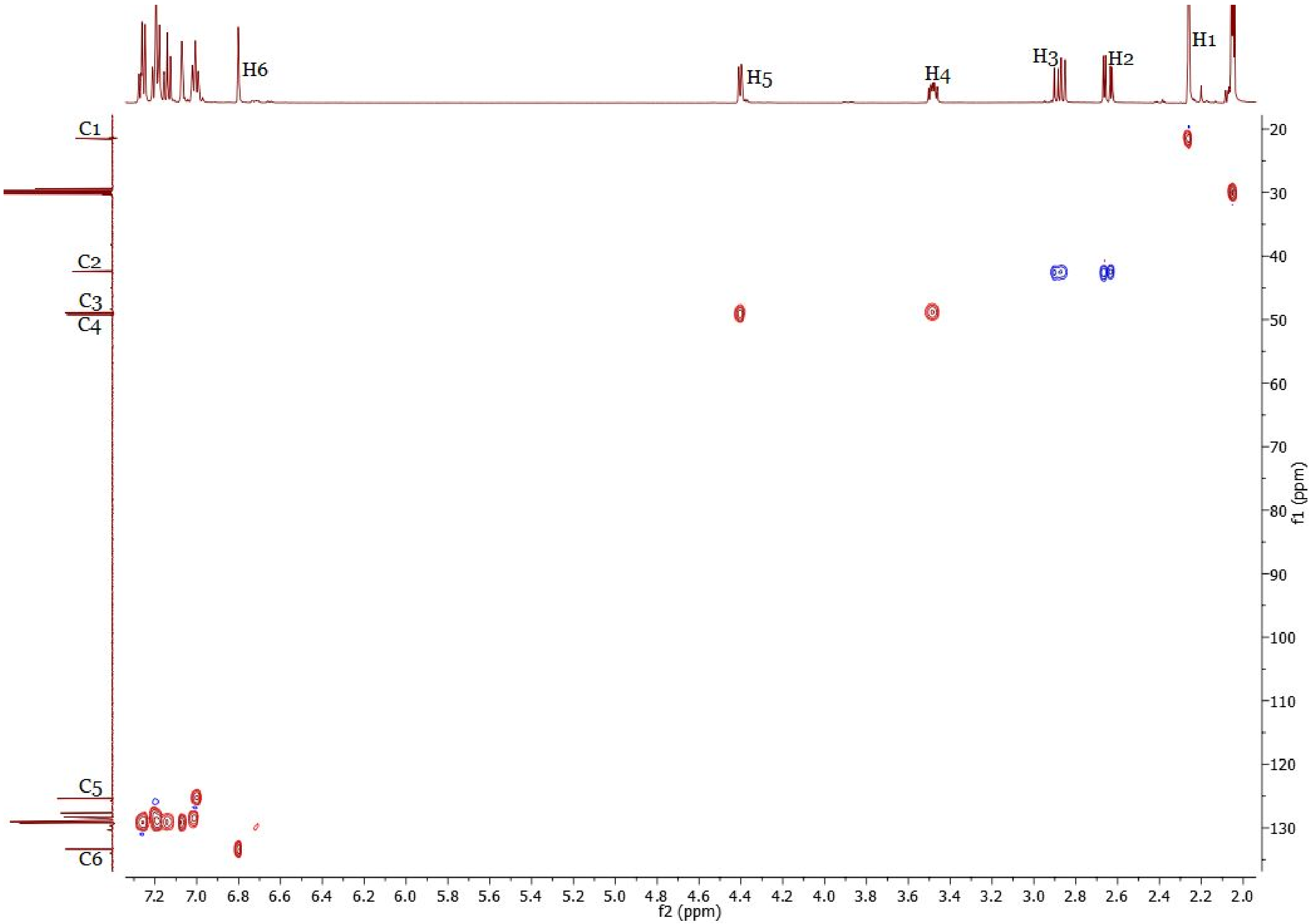

*Figure S52: HSQC spectrum of **4**.*

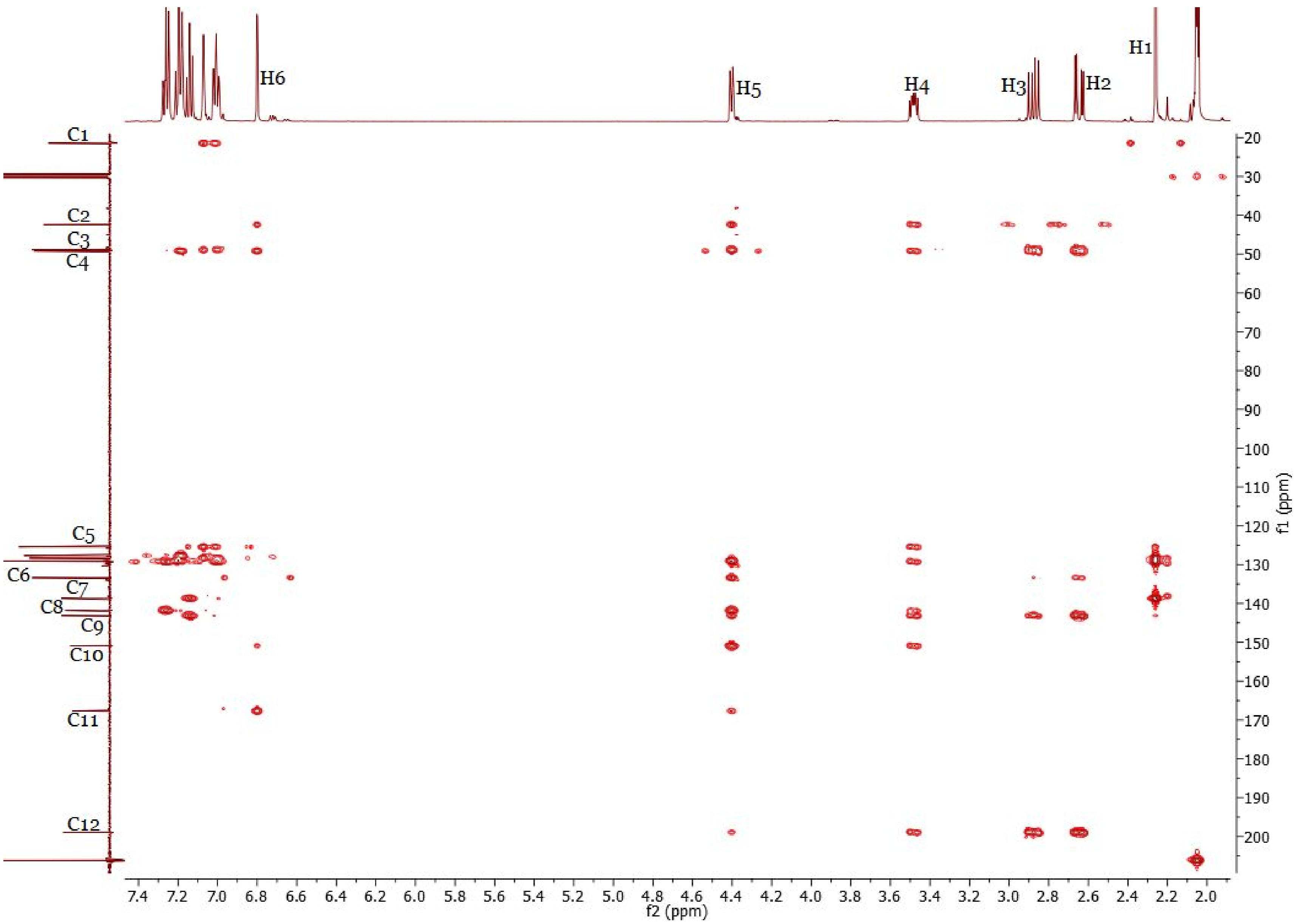

*Figure S53: HMBC spectrum of **4**.*

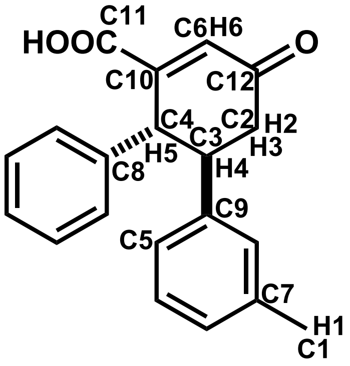

Figure S54: 2D NMR observations of ***4****.*

2D NMR observations of **4**:

Protons H1 are attached to carbon C1 forming CH_3 group. The group has connectivity to carbon C7 and elsewhere in the substituted phenyl group.

Protons H2 and H3 are attached to carbon C2 forming CH_2 group. The group has connectivity to carbons C3, C4, C6 (weak), C9 and C12.

Proton H4 is attached to carbon C3 forming CH group. The group has connectivity to carbons C2, C4, C5, C8, C9, C10 and C12.

Proton H5 is attached to carbon C4 forming CH group. The group has connectivity to carbons C2, C3, C6, C8, C9, C10, C11 and C12 (weak). The group has connectivity inside the phenyl group, suggesting nearby location.

Proton H6 is attached to carbon C6 forming CH group. The group has connectivity to carbons C2, C4, C10 and C11.

IR spectroscopy of **4**

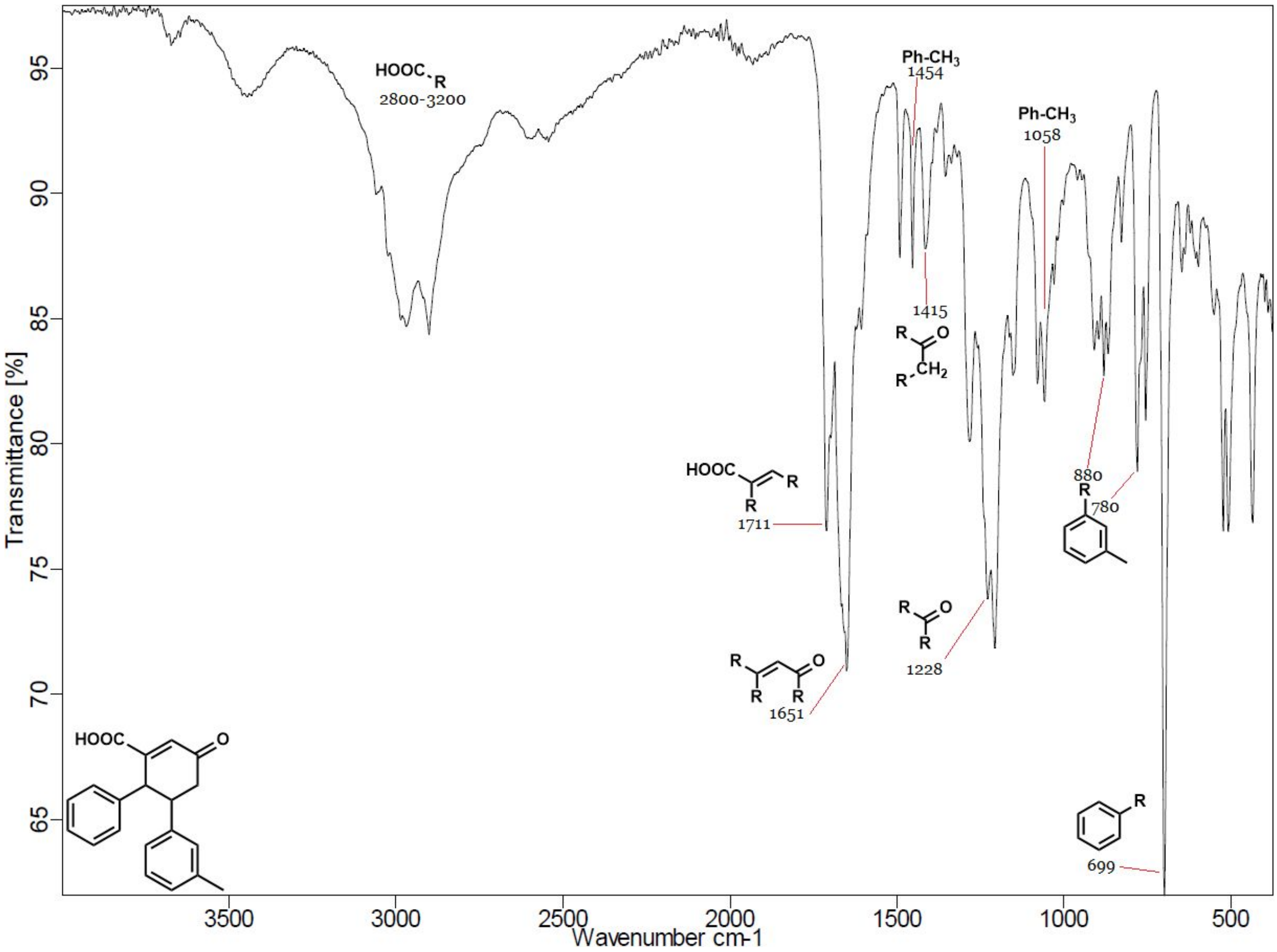

*Figure S55: IR spectrum of **4** (2800-3200 (broad) (R-COOH), 1711 (s) (C=C-ROOH), 1651 (s) (C=C-**CO**-R), 1454 (m), 1058 (m) (Ph-CH_3), 1415 (m) (R-CO-**CH_2**-R), 1228 (s) (R-**CO**-R), 880 (m) (isolated H (Ph)), 780 (m) (3 adjacent H (Ph)), 699 (s) (5 adjacent H (Ph)) cm^{-1})*

HRMS of **4**

HRMS (ESI-TOF) m/z: [**4**-H]$^-$ calculated for $C_{20}H_{17}O_3$ 305.1172; Found 305.1173; Error 0.292 ppm.

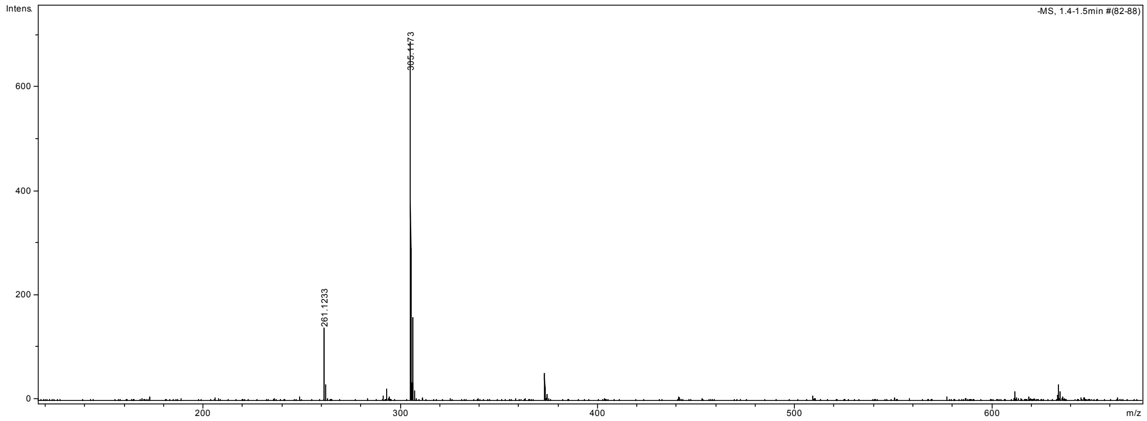

*Figure S56: ESI-TOF-MS of [**4**-H]$^-$ (peak: 305.1173 m/z, negative-ion mode).*

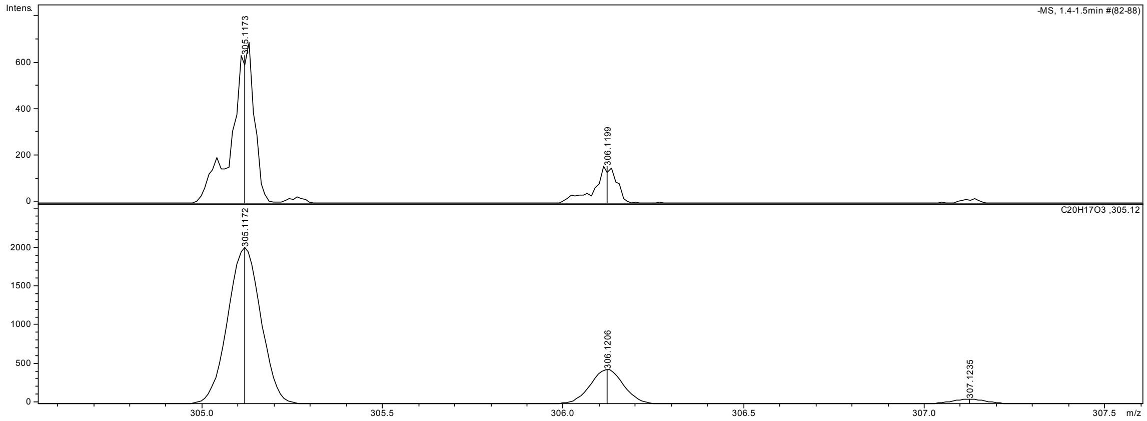

*Figure S57: Measured compound peak of [**4**-H]$^-$ (305.1173 m/z) at top, simulated peak ($C_{20}H_{17}O_3$) below.*

3.7 Spectroscopic data of 5

1D NMR of **5**

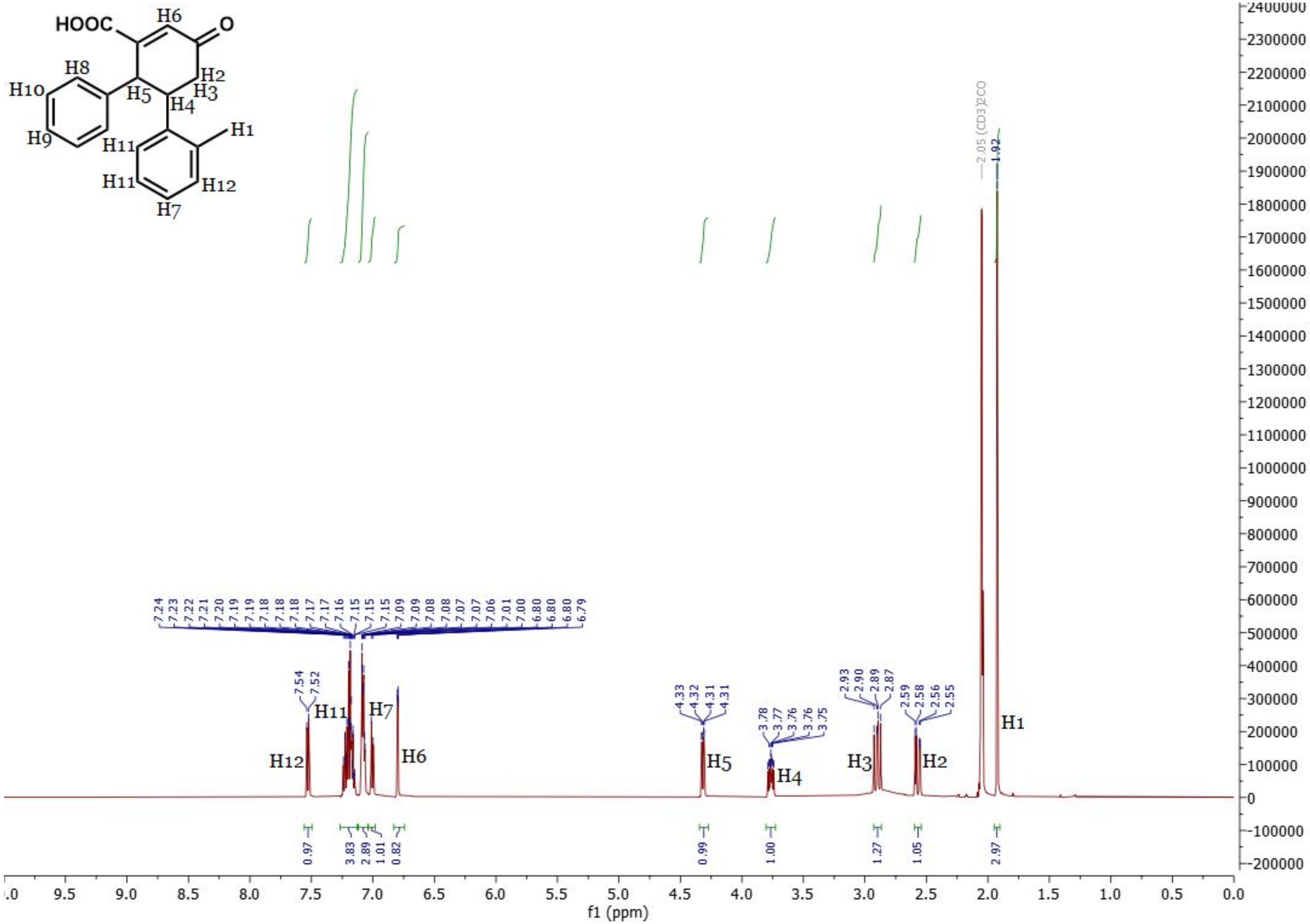

*Figure S58: ^{1}H NMR spectrum of **5** from full region (500 MHz, acetone-d6): δ 7.53 (d, J = 7.8 Hz, 1H), 7.25-7.13 (m, 4H), 7.10-7.05 (m, 3H), 7.01 (d, J = 7.5 Hz, 1H), 6.80 (dd, J = 2.2, 0.6 Hz, 1H), 4.32 (dd, J = 8.2, 2.2 Hz, 1H), 3.76 (ddd, J = 11.2, 8.2, 4.5 Hz, 1H), 2.90 (dd, J = 16.3, 11.2 Hz, 1H), 2.57 (dd, J = 16.2, 4.4 Hz, 1H), 1.92 (s, 3H).*

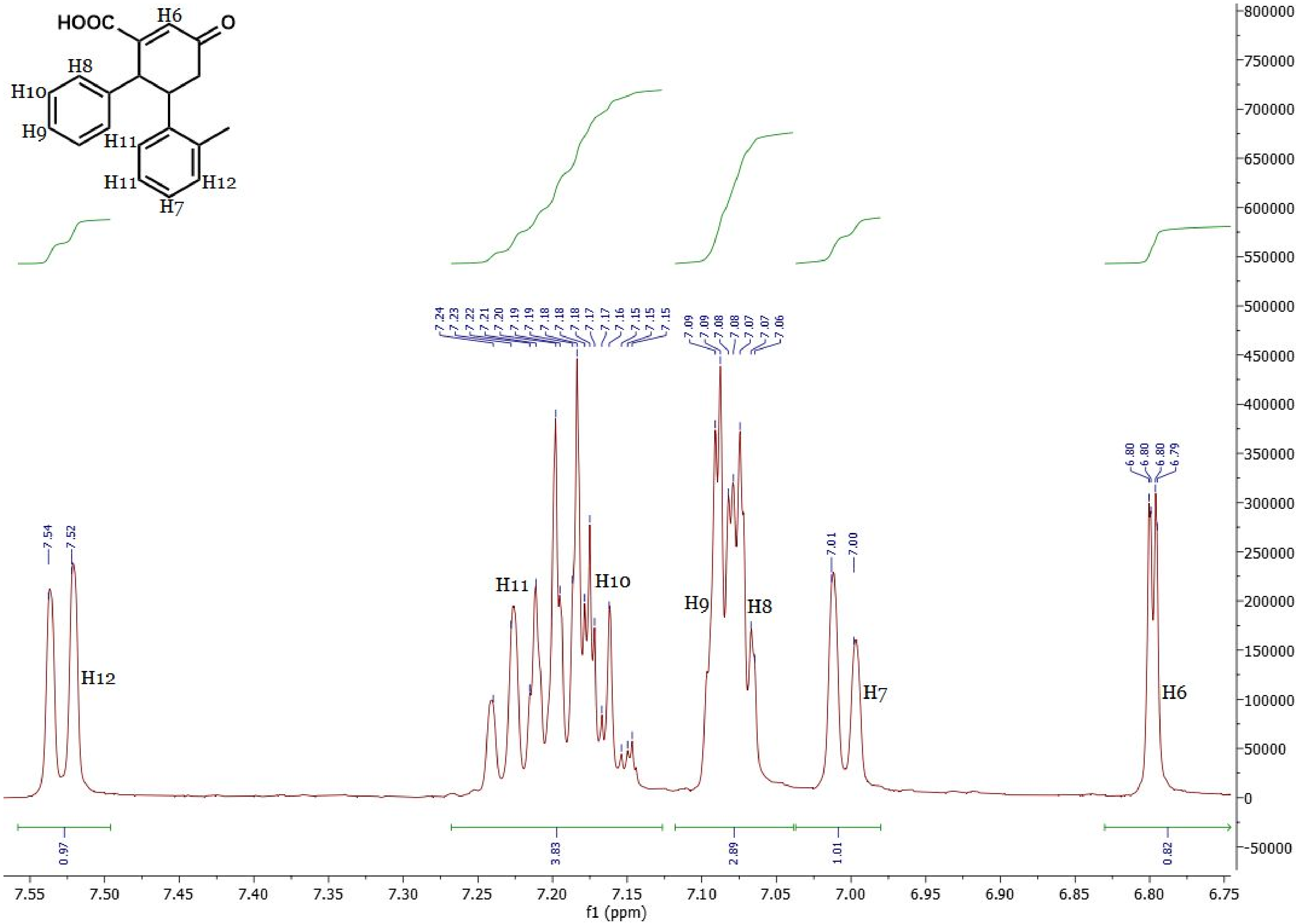

*Figure S59: ^{1}H NMR spectrum of **5** from aromatic region (500 MHz, acetone-d6): δ 7.53 (d, J = 7.8 Hz, 1H), 7.25-7.13 (m, 4H), 7.10-7.05 (m, 3H), 7.01 (d, J = 7.5 Hz, 1H), 6.80 (dd, J = 2.2, 0.6 Hz, 1H).*

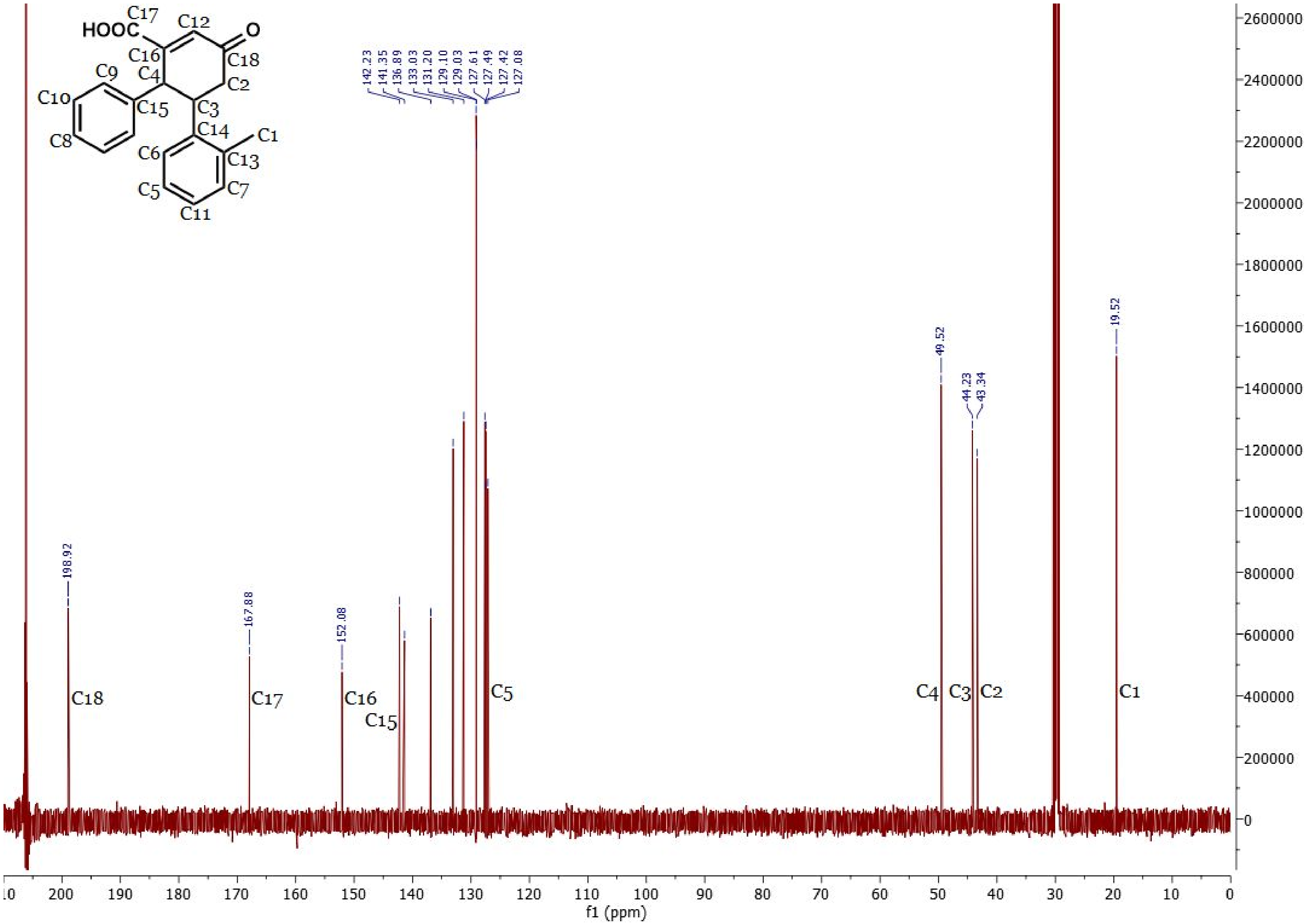

*Figure S60: $^{13}C\{^{1}H\}$ NMR spectrum of **5** from full region (125 MHz, acetone-d6): δ 198.92, 167.88, 152.08, 142.23, 141.35, 136.89, 133.03, 131.20, 129.10, 129.03, 127.61, 127.49, 127.42, 127.08, 49.52, 44.23, 43.34, 19.52.*

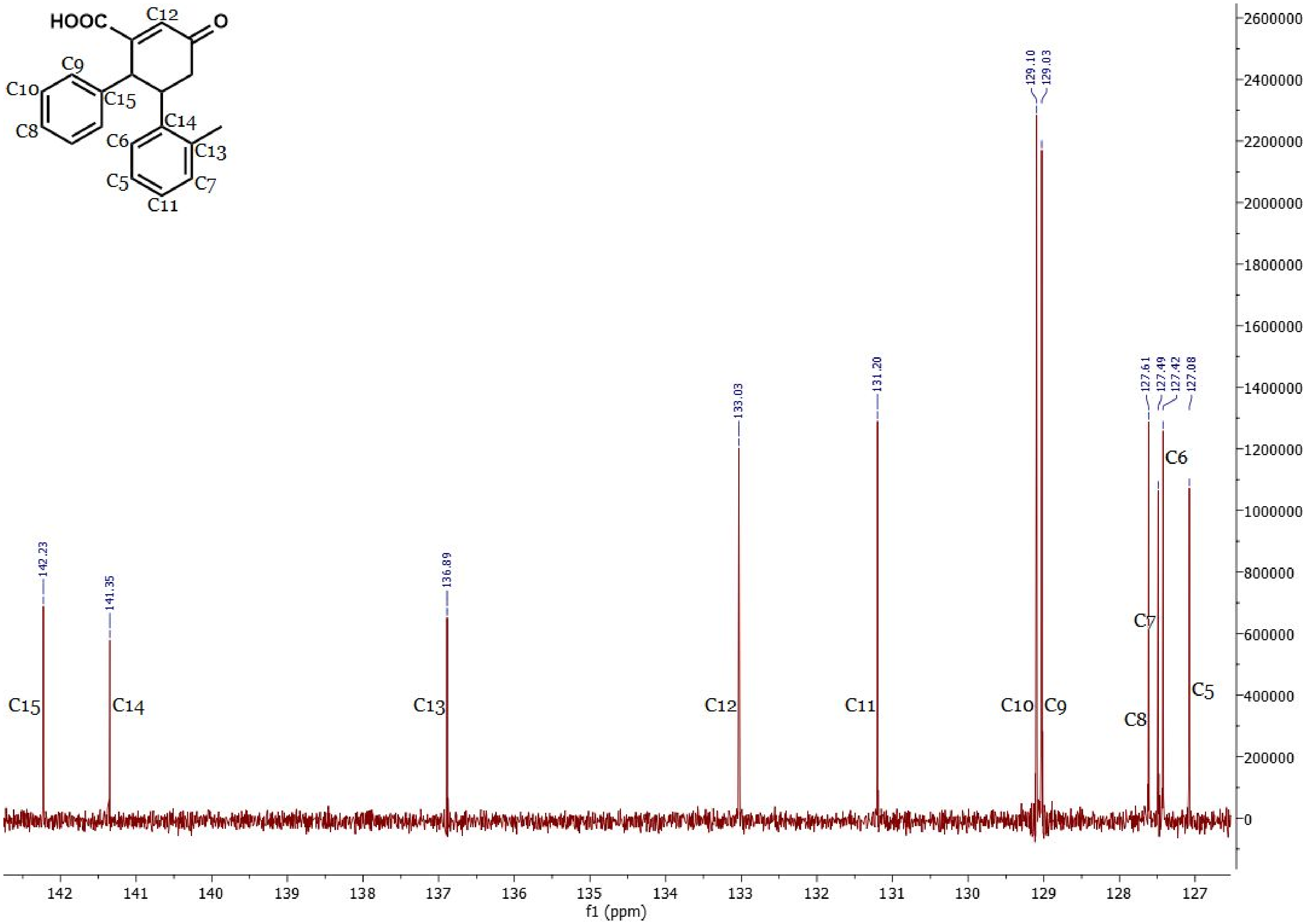

*Figure S61: $^{13}C\{^{1}H\}$ NMR spectrum of **5** from aromatic region (125 MHz, acetone-d6): δ 142.23, 141.35, 136.89, 133.03, 131.20, 129.10, 129.03, 127.61, 127.49, 127.42, 127.08.*

2D NMR of **5**

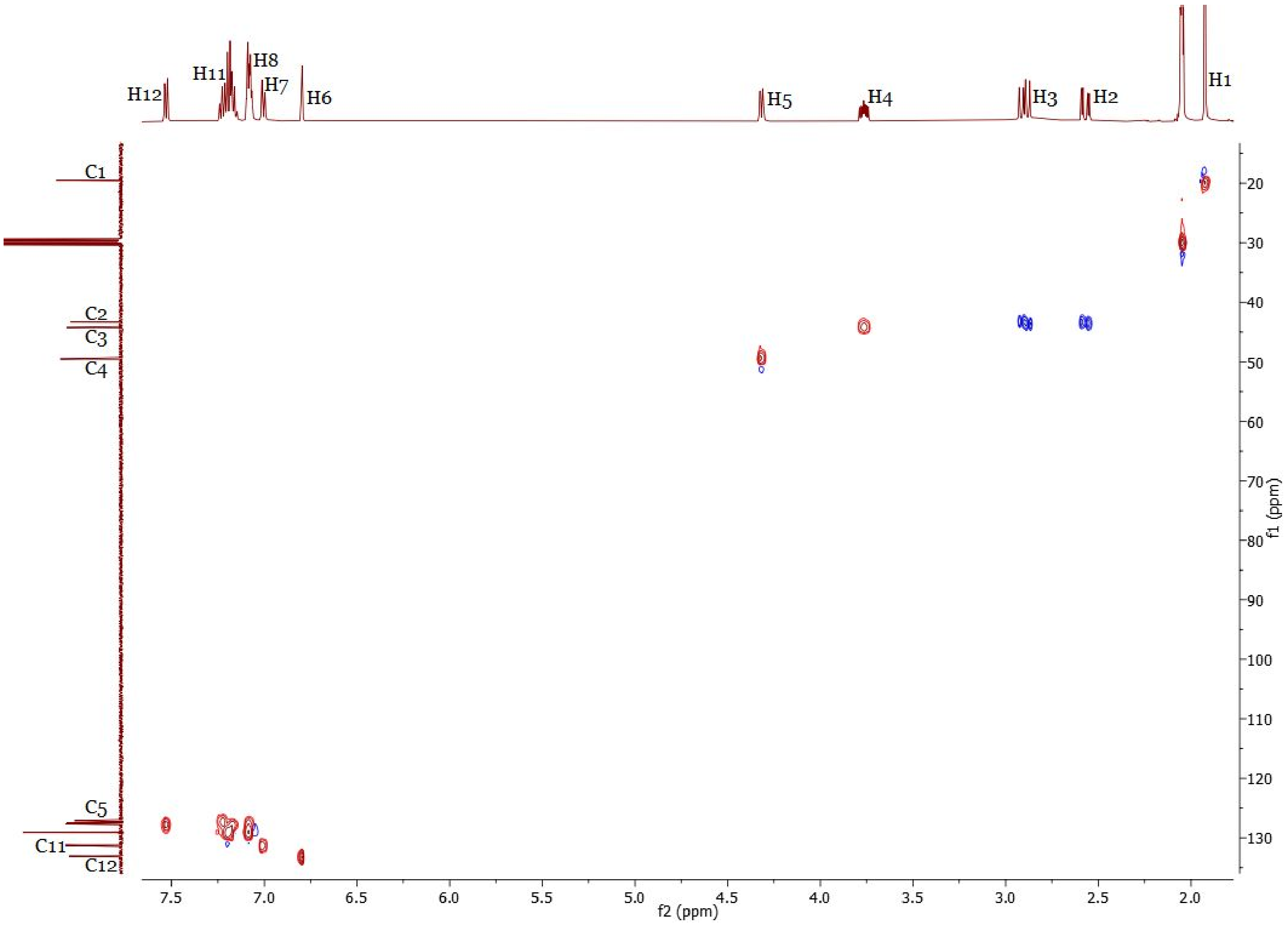

*Figure S62: HSQC spectrum of **5**.*

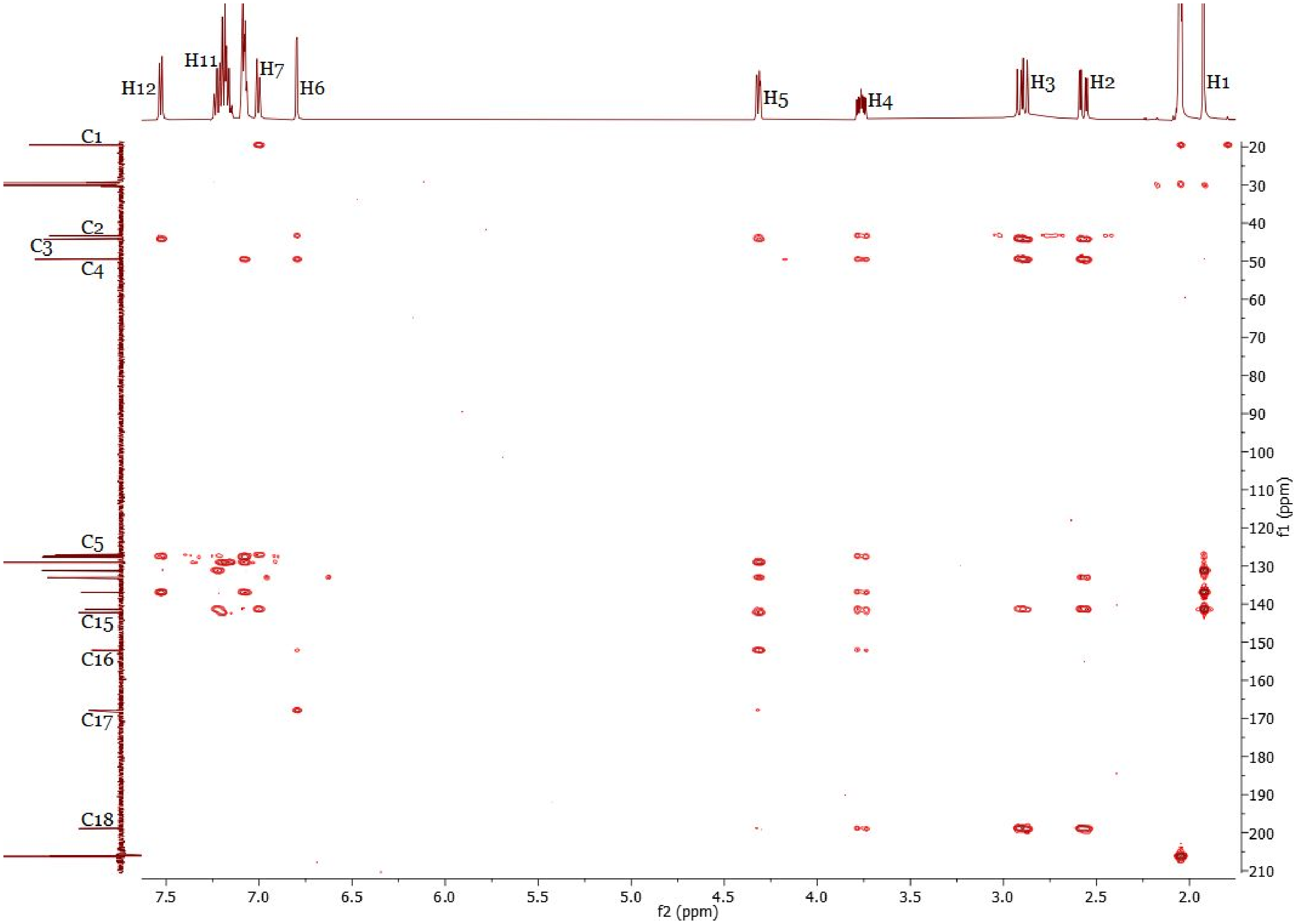

*Figure S63: HMBC spectrum of **5**.*

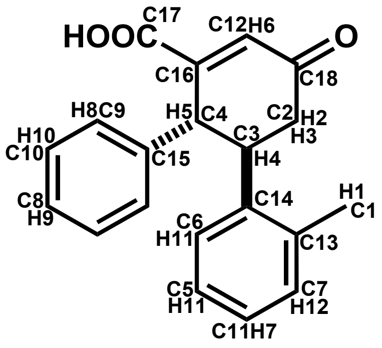

*Figure S64: 2D NMR observations of **5**.*

2D NMR observations for **5**:

Protons H1 are attached to carbon C1 forming CH_3 group. The group has connectivity to carbons C11, C13 and C14.

Protons H2 and H3 are attached to carbon C2 forming CH_2 group. The group has connectivity to carbons C3, C4, C12 and C18.

Proton H4 is attached to carbon C3 forming CH group. The group has connectivity to carbons C2, C4, C6, C13, C14, C16 (weak) and C18.

Proton H5 is attached to carbon C4 forming CH group. The group has connectivity to carbons C3, C9, C12, C14 and C15.

Proton H6 is attached to carbon C12 forming CH group. The group has connectivity to carbons C2, C4, C16 and C17.

Proton H7 is attached to carbon C11 forming CH group. The group has connectivity to carbons C1, C5 and C14.

Protons H8 are attached to carbons C9 forming two CH groups. The groups have connectivity to carbons C4, C9 (itself) and C10.

Proton H9 is attached to carbon C8 forming CH group. The group has connectivity to carbons C9 and C10.

Protons H10 are attached to carbons C10 forming two CH groups. The groups have connectivity to carbons C9 and C15.

Proton H11 is attached to carbon C5 forming CH group. The group has connectivity to carbons C7, C11 and C14.

Proton H11 is attached to carbon C6 forming CH group. The group has connectivity to carbon C3, C7, C11 and C14.

Proton H12 is attached to carbon C7 forming CH group. The group has connectivity to carbons C3, C6 and C13.

IR spectroscopy of **5**

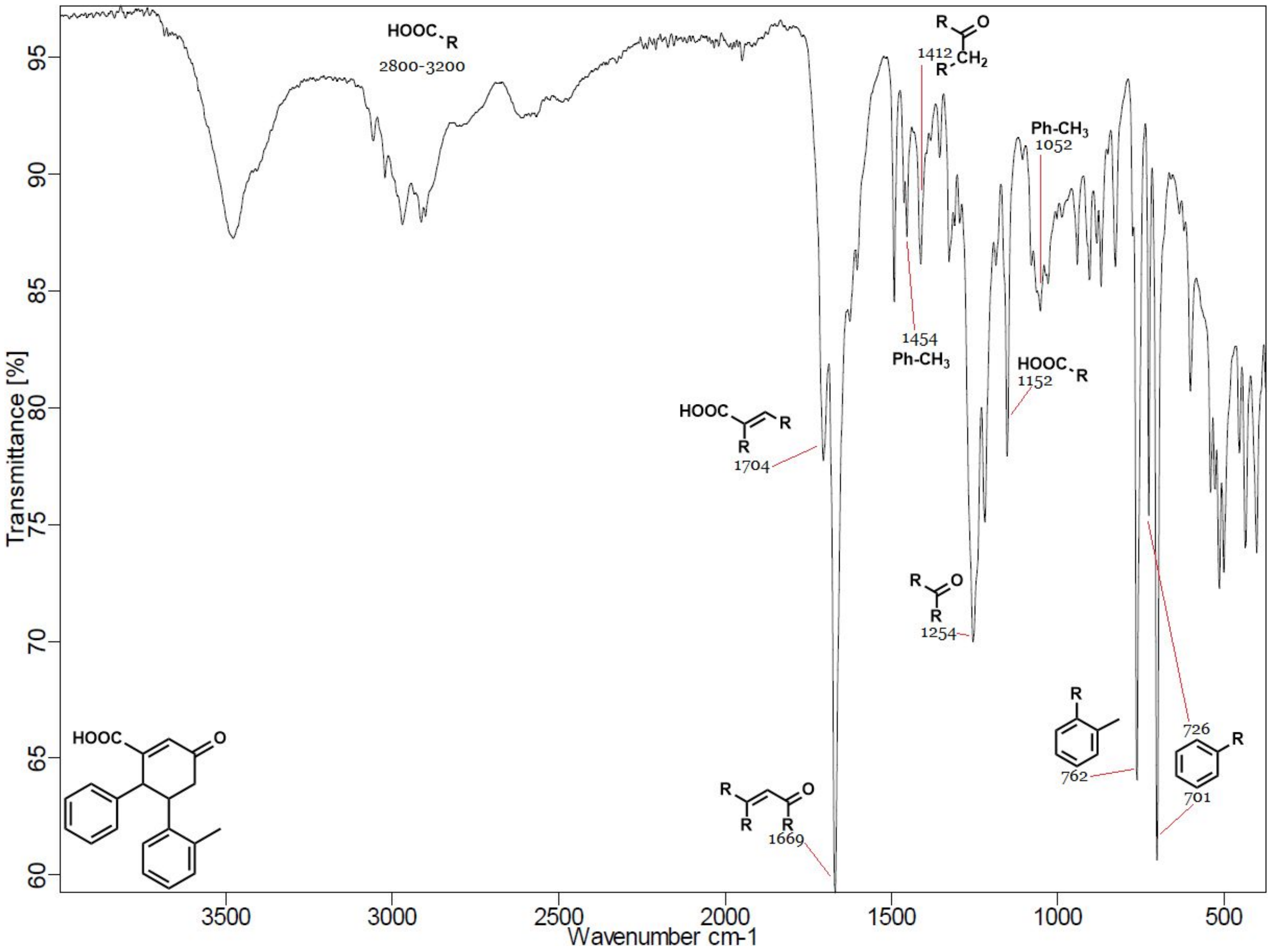

*Figure S65: IR spectrum of **5** (2800-3200 (broad) (R-COOH), 1704 (m) (C=C-ROOH), 1669 (s) (C=C-**CO**-R), 1454 (w), 1052 (m) (Ph-CH_3), 1412 (w) (R-CO-**CH_2**-R), 1254 (s) (R-**CO**-R), 1152 (m) (R-**CO**OH), 762 (s) (4 adjacent H (Ph)), 701 (s), 726 (s) (5 adjacent H (Ph)) cm^{-1}).*

HRMS of **5**

HRMS (ESI-TOF) m/z: [**5**-H]$^-$ calculated for $C_{20}H_{17}O_3$ 305.1172; Found 305.1174; Error 0.597 ppm.

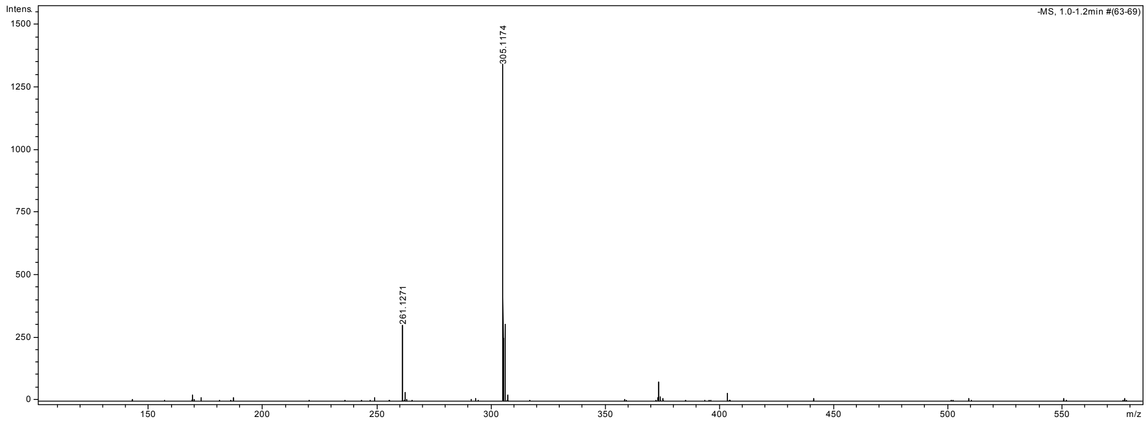

*Figure S66: ESI-TOF-MS of [**5**-H]$^-$ (peak: 305.1174 m/z, negative-ion mode).*

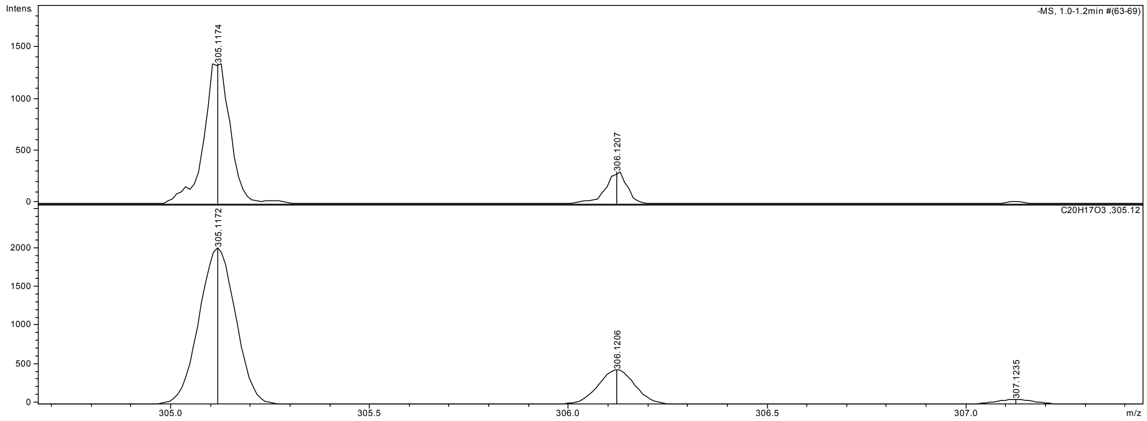

*Figure S67: Measured compound peak of [**5**-H]$^-$ (305.1174 m/z) at top, simulated peak ($C_{20}H_{17}O_3$) below.*

3.8 Spectroscopic data of 6

1D NMR of **6**

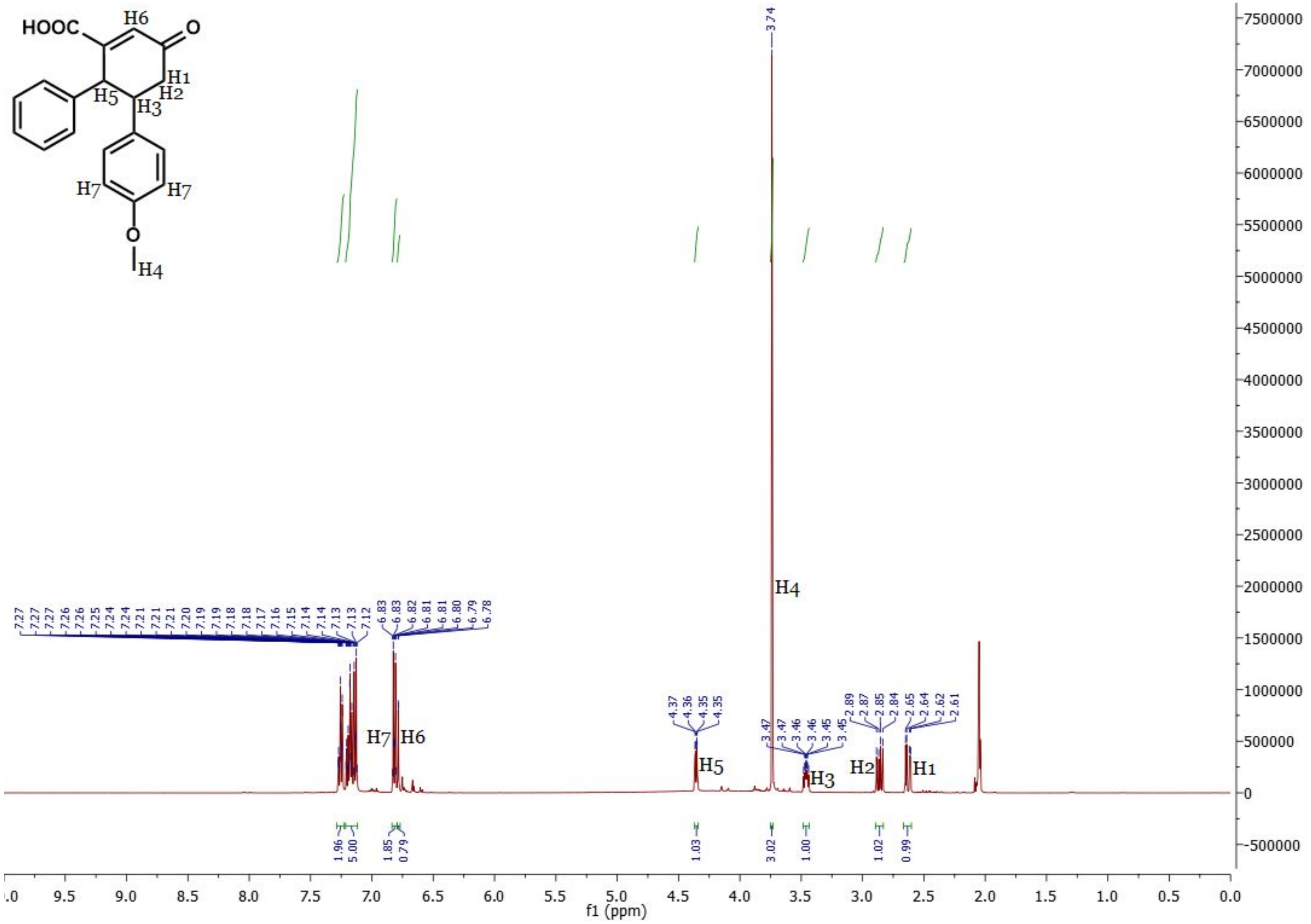

*Figure S68: ^{1}H NMR spectrum of **6** (500 MHz, acetone-d6): δ 7.28-7.23 (m, 2H), 7.21-7.12 (m, 5H), 6.84-6.80 (m, 2H), 6.78 (d, J = 1.8 Hz, 1H), 4.36 (dd, J = 6.7, 1.6 Hz, 1H), 3.74 (s, 3H), 3.46 (ddd, J = 9.2, 6.7, 4.6 Hz, 1H), 2.86 (dd, J = 16.4, 9.2 Hz, 1H), 2.63 (dd, J = 16.4, 4.5 Hz, 1H).*

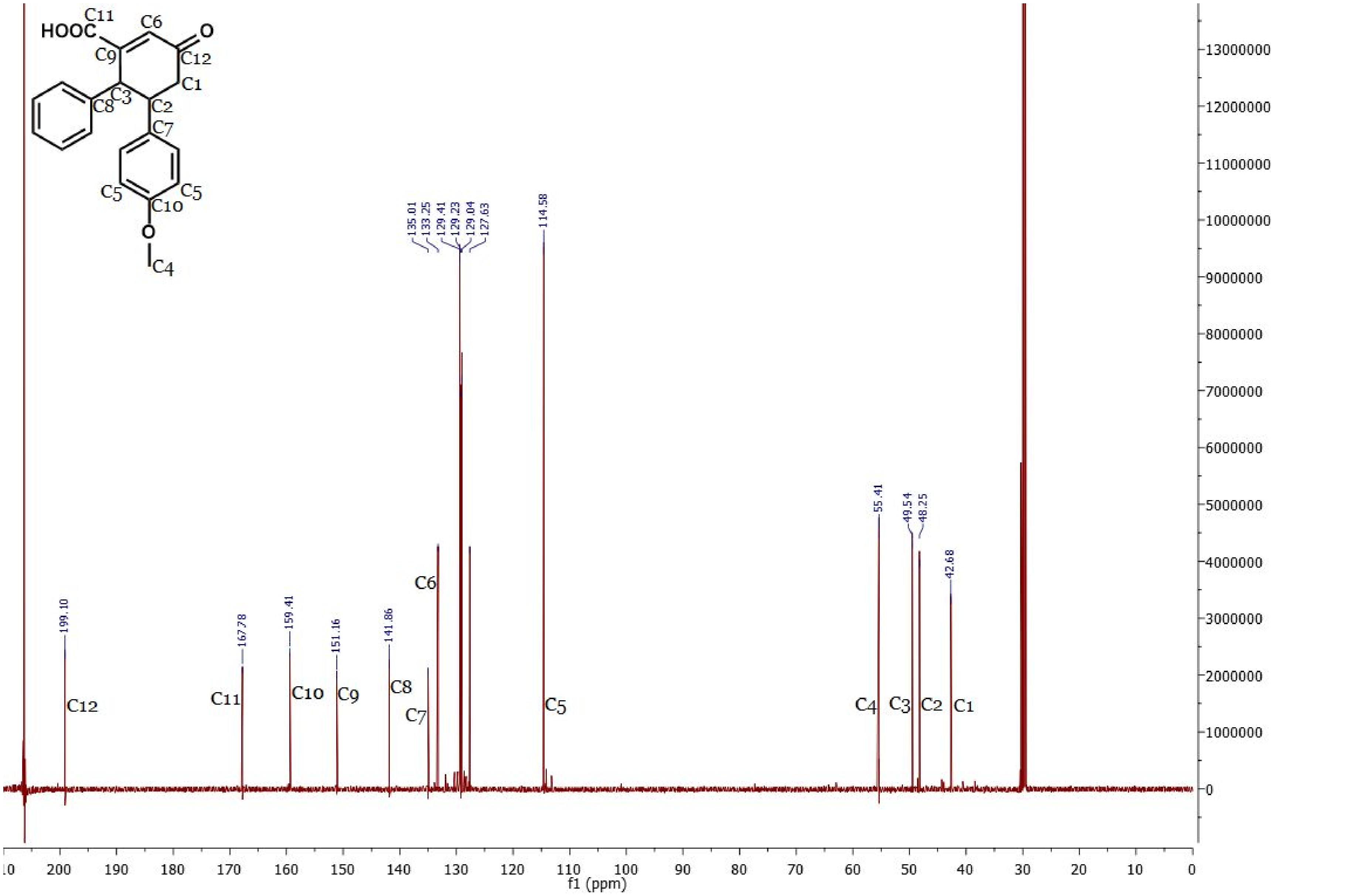

*Figure S69: $^{13}C\{^{1}H\}$ NMR spectrum of **6** (125 MHz, acetone-d6): δ 199.10, 167.78, 159.41, 151.16, 141.86, 135.01, 133.25, 129.41, 129.23, 129.04, 127.63, 114.58, 55.41, 49.54, 48.25, 42.68.*

2D NMR of **6**

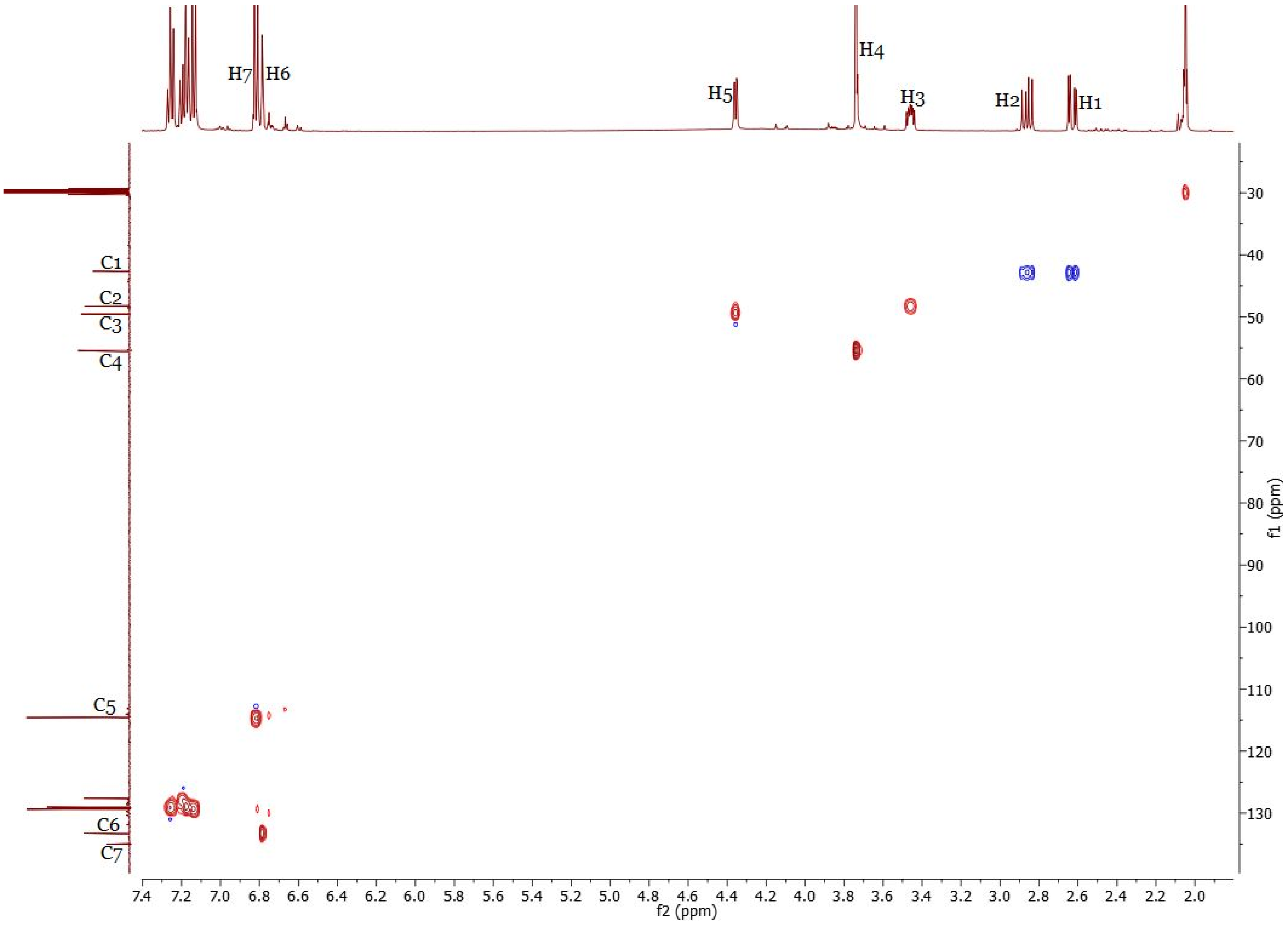

*Figure S70: HSQC spectrum of **6**.*

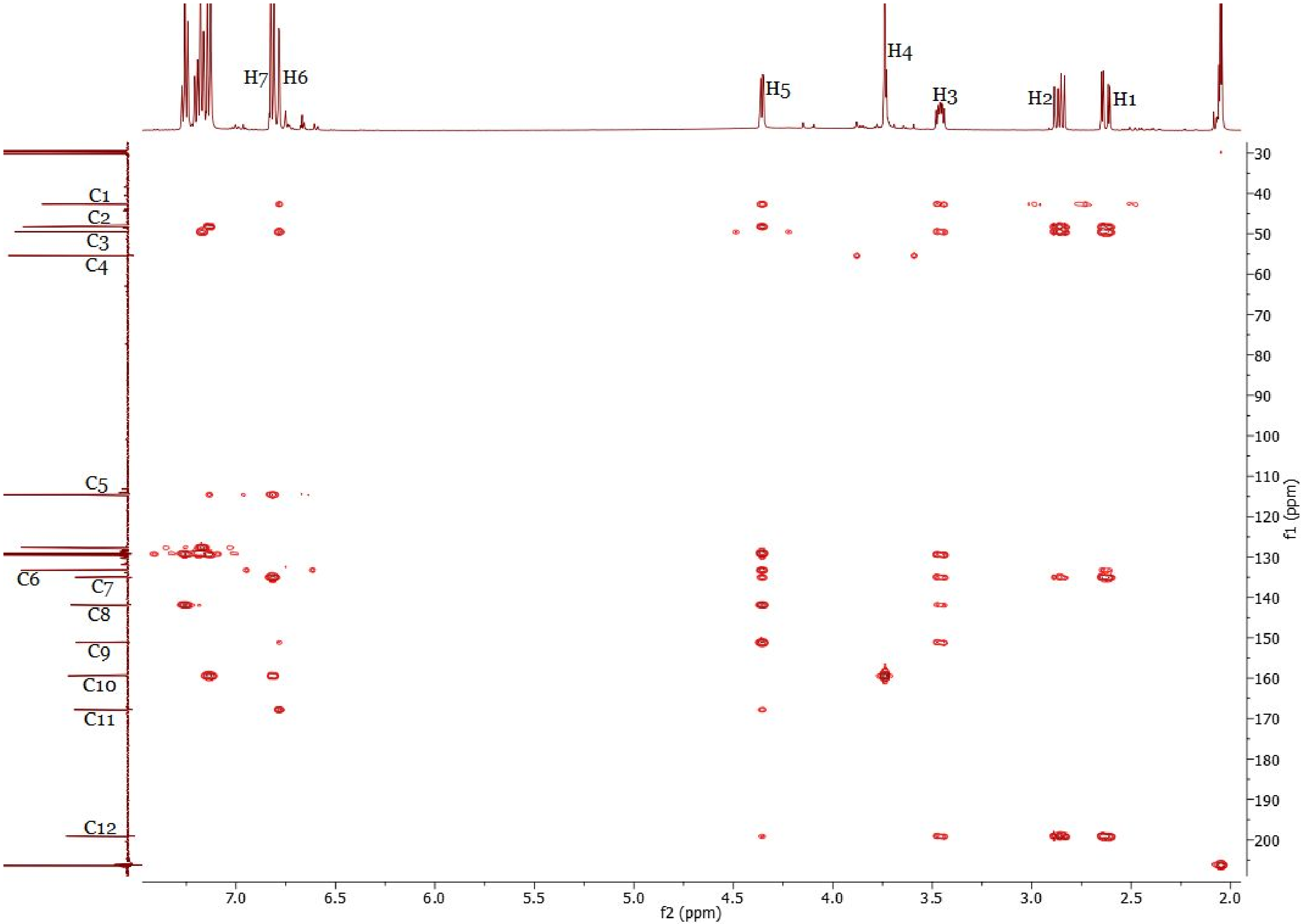

*Figure S71: HMBC spectrum of **6**.*

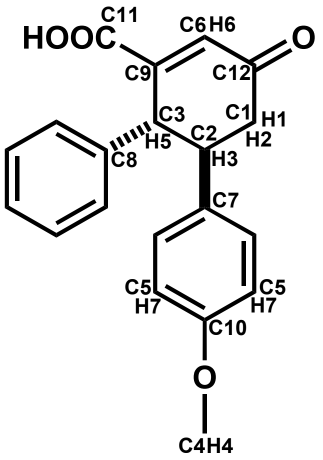

*Figure S72: 2D NMR observations of **6**.*

2D NMR observations of **6**:

Protons H1 and H2 are attached to carbon C1 forming CH_2 group. The group has connectivity to carbons C2, C3, C6 (weak), C7 and C12.

Proton H3 is attached to carbon C2 forming CH group. The group has connectivity to carbons C1, C3, C7, C8, C9 and C12. The group sees inside substituted phenyl ring, suggesting nearby location.

Protons H4 are attached to carbon C4 forming CH_3 group. The group has connectivity to carbon C10.

Proton H5 is attached to carbon C3 forming CH group. The group has connectivity to carbons C1, C2, C6, C7, C8, C9, C11 (weak) and C12 (weak). The group sees inside of the phenyl group suggesting nearby location.

Proton H6 is attached to carbon C6 forming CH group. The group has connectivity to carbons C1, C3, C9 (weak) and C11.

Protons H7 are attached to carbons C5 forming two CH groups. The groups has connectivity to carbons C5, C7 and C10.

IR spectroscopy of **6**

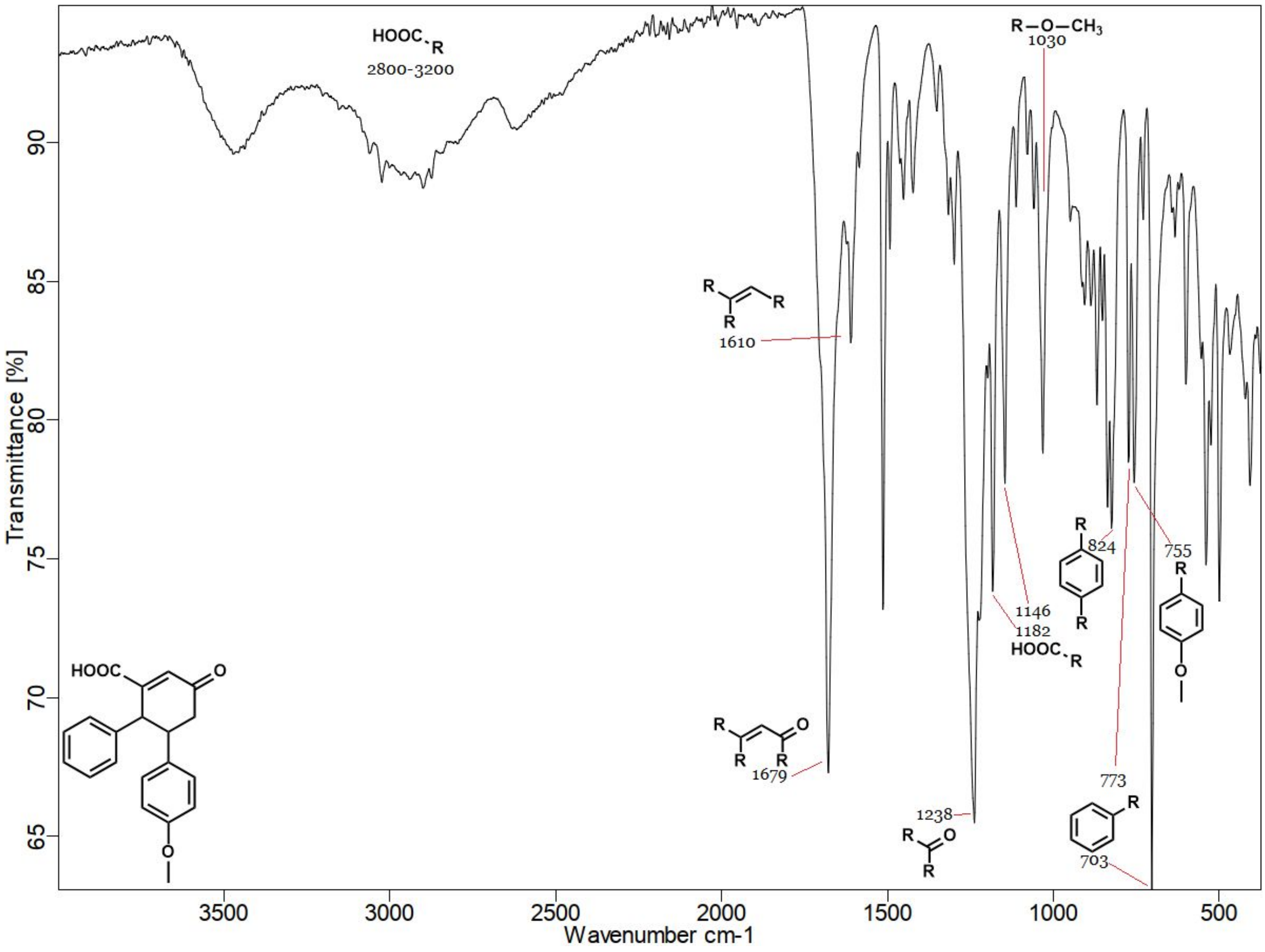

*Figure S73: IR spectrum of **6** (2800-3200 (broad), 1182 (s), 1146 (s) (R-COOH), 1679 (s) (C=C-**CO**-R), 1610 (m) (R_2-C=**CH**-R), 1238 (s) (R-**CO**-R), 1030 (m) (R-O-CH_3), 824 (s) (2 adjacent H (Ph)), 773 (s), 703 (s) (5 adjacent H (Ph)), 755 (m) (Ph-O-CH_3)) cm^{-1}).*

HRMS of **6**

HRMS (ESI-TOF) m/z: [**6**-H]$^-$ calculated for $C_{20}H_{17}O_4$ 321.1121; Found 321.1120; Error 0.485 ppm.

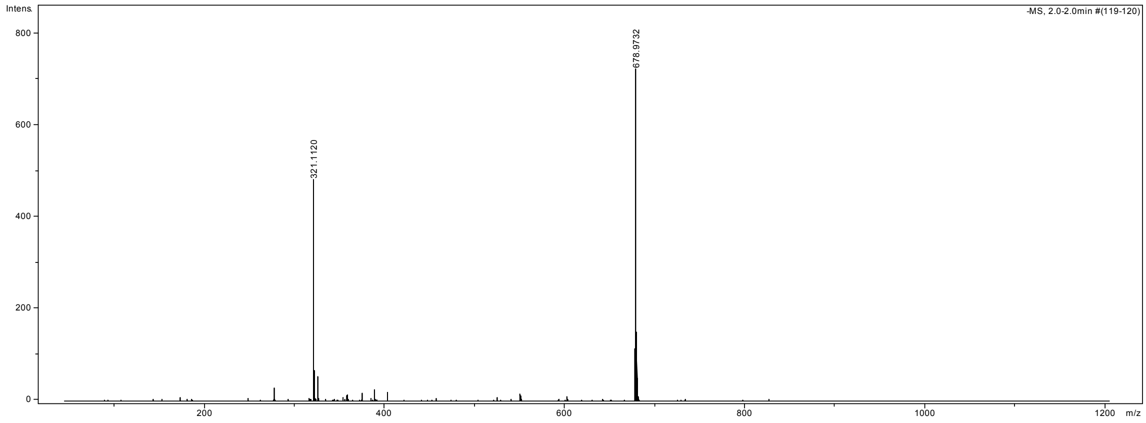

*Figure S74: ESI-TOF-MS of [**6**-H]$^-$ (peak: 321.1120 m/z, negative-ion mode).*

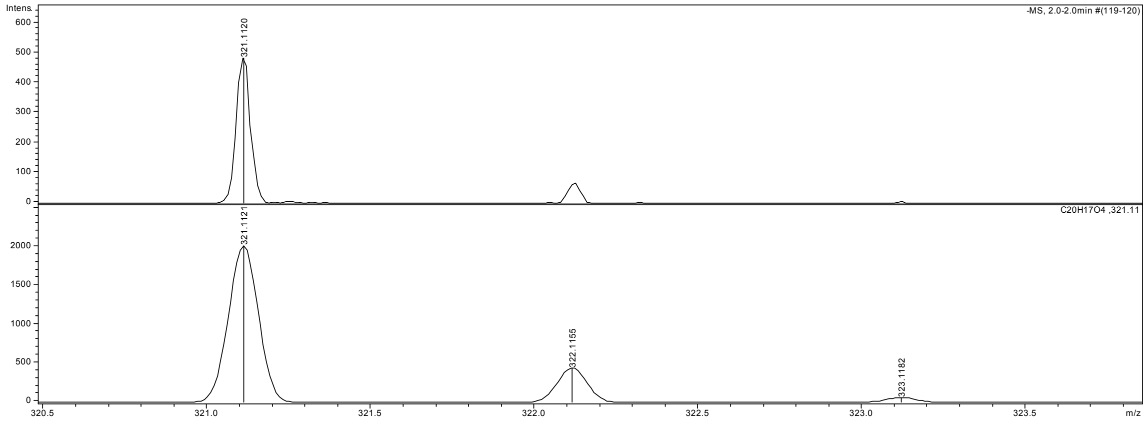

*Figure S75: Measured compound peak of [**6**-H]$^-$ (321.1120 m/z) at top, simulated peak ($C_{20}H_{17}O_4$) below.*

3.9 Spectroscopic data of 7

1D NMR of **7**

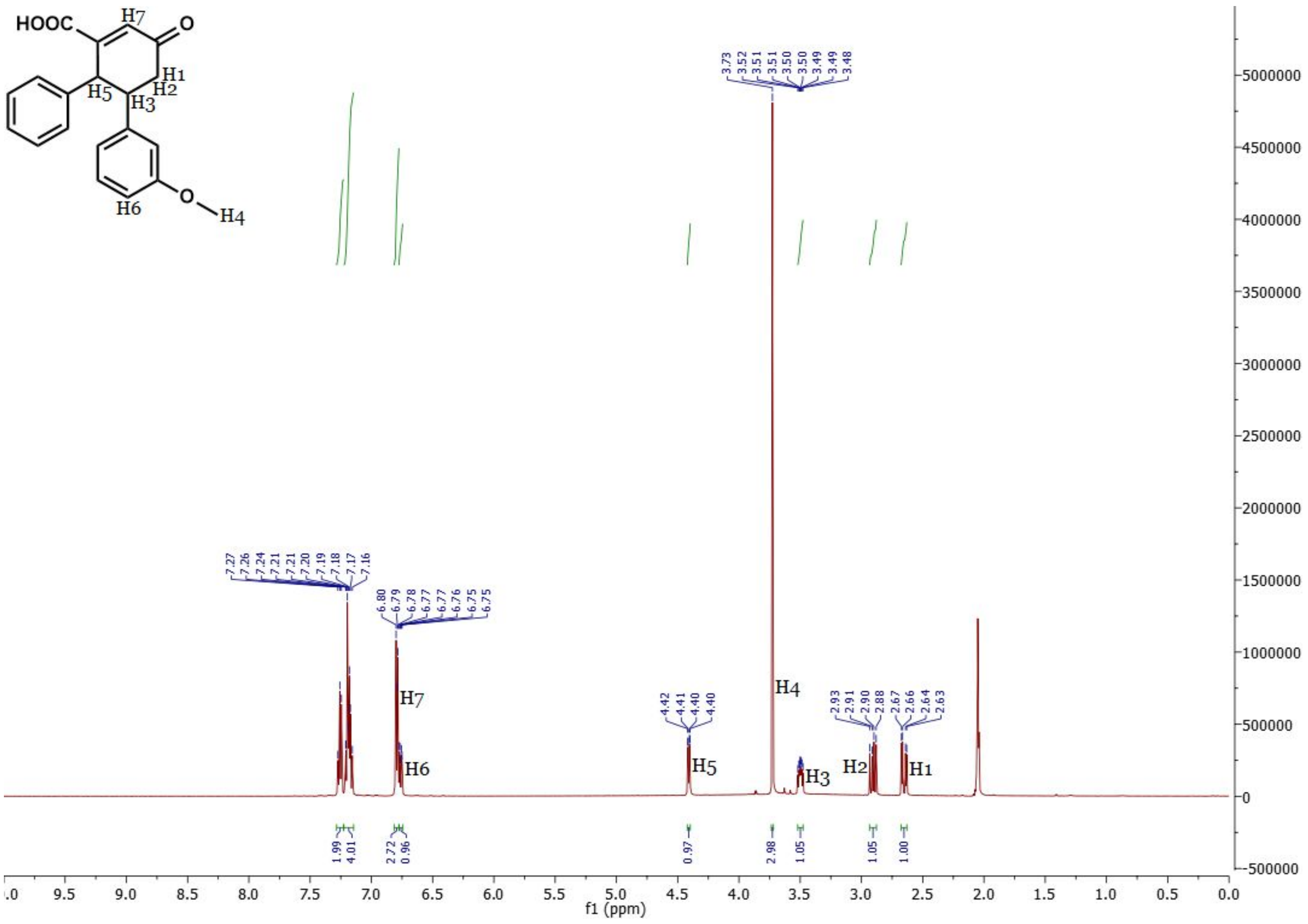

*Figure S76: ^{1}H NMR spectrum of **7** (500 MHz, acetone-d6): δ 7.28-7.23 (m, 2H), 7.22-7.15 (m, 4H), 6.81-6.78 (m, 3H), 6.77-6.74 (m, 1H), 4.41 (dd, J = 6.8, 1.5 Hz, 1H), 3.73 (s, 3H), 3.50 (ddd, J = 9.4, 6.8, 4.6 Hz, 1H) 2.91 (dd, J = 16.4, 9.4 Hz, 1H), 2.65 (dd, J = 16.4, 4.5 Hz, 1H).*

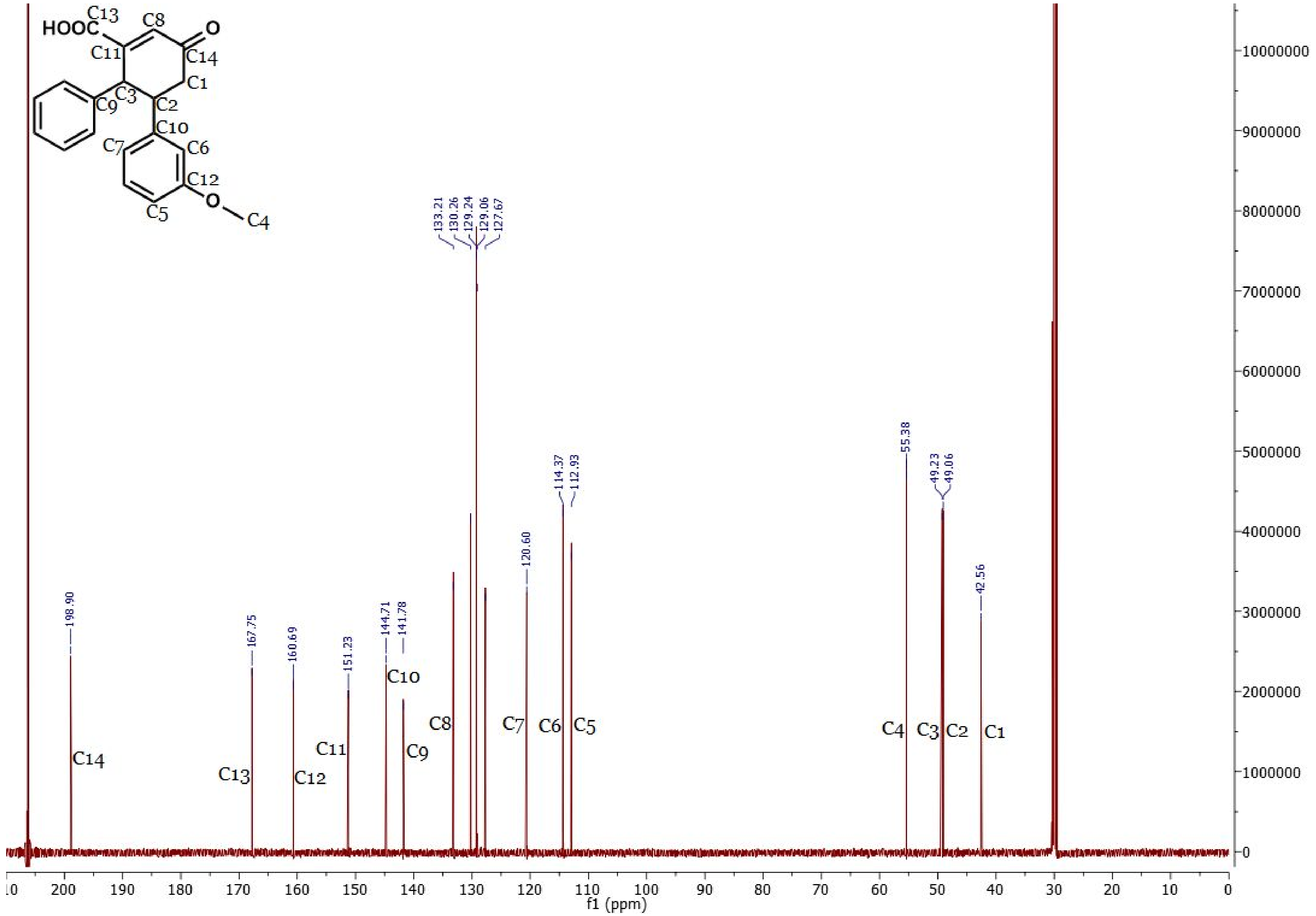

*Figure S77: $^{13}C\{^{1}H\}$ NMR spectrum of **7** (125 MHz, acetone-d6): δ 198.90, 167.75, 160.69, 151.23, 144.71, 141.78, 133.21, 130.26, 129.24, 129.06, 127.67, 120.60, 114.37, 112.93, 55.38, 49.23, 49.06, 42.56.*

2D NMR of **7**

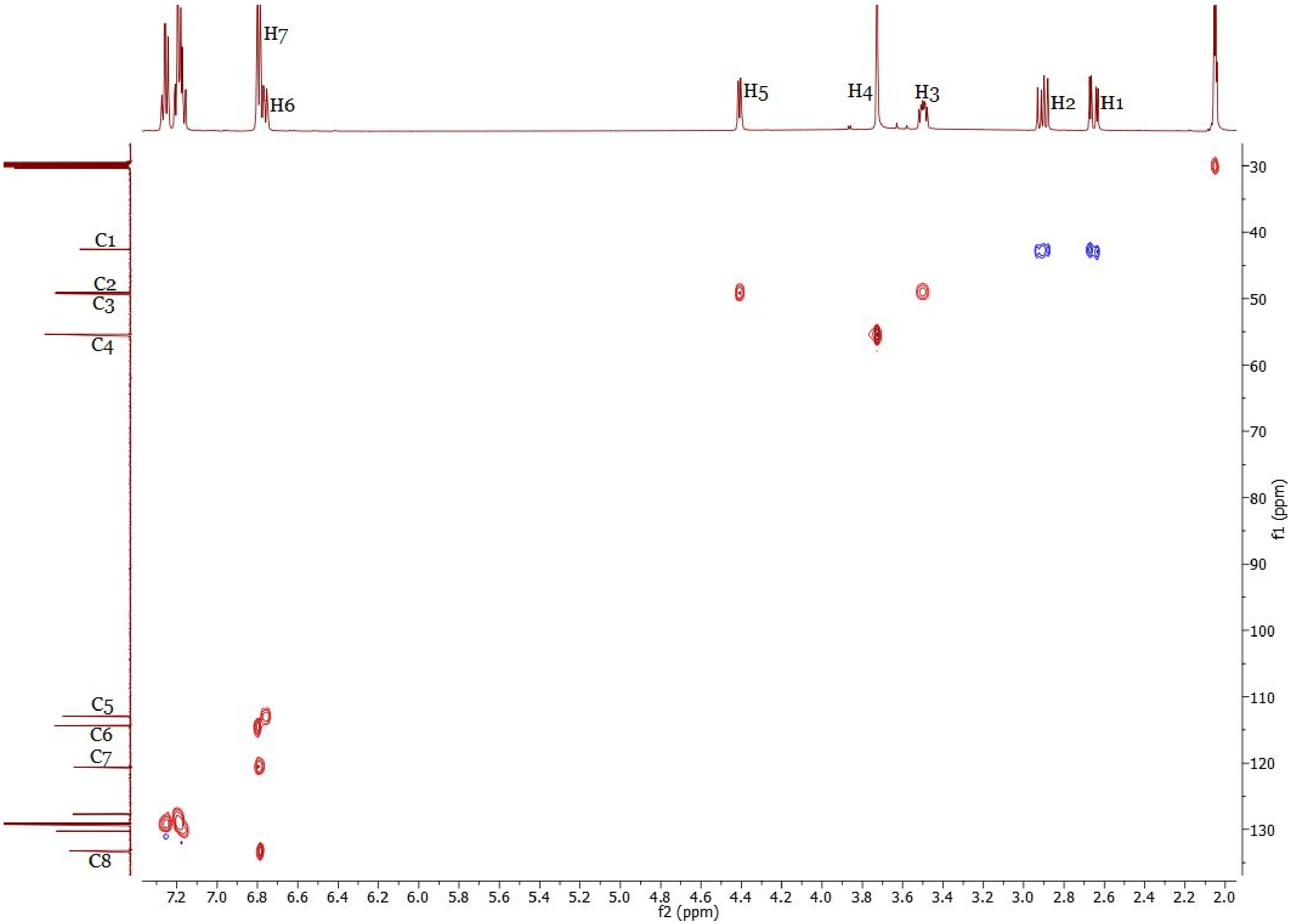

*Figure S78: HSQC spectrum of **7**.*

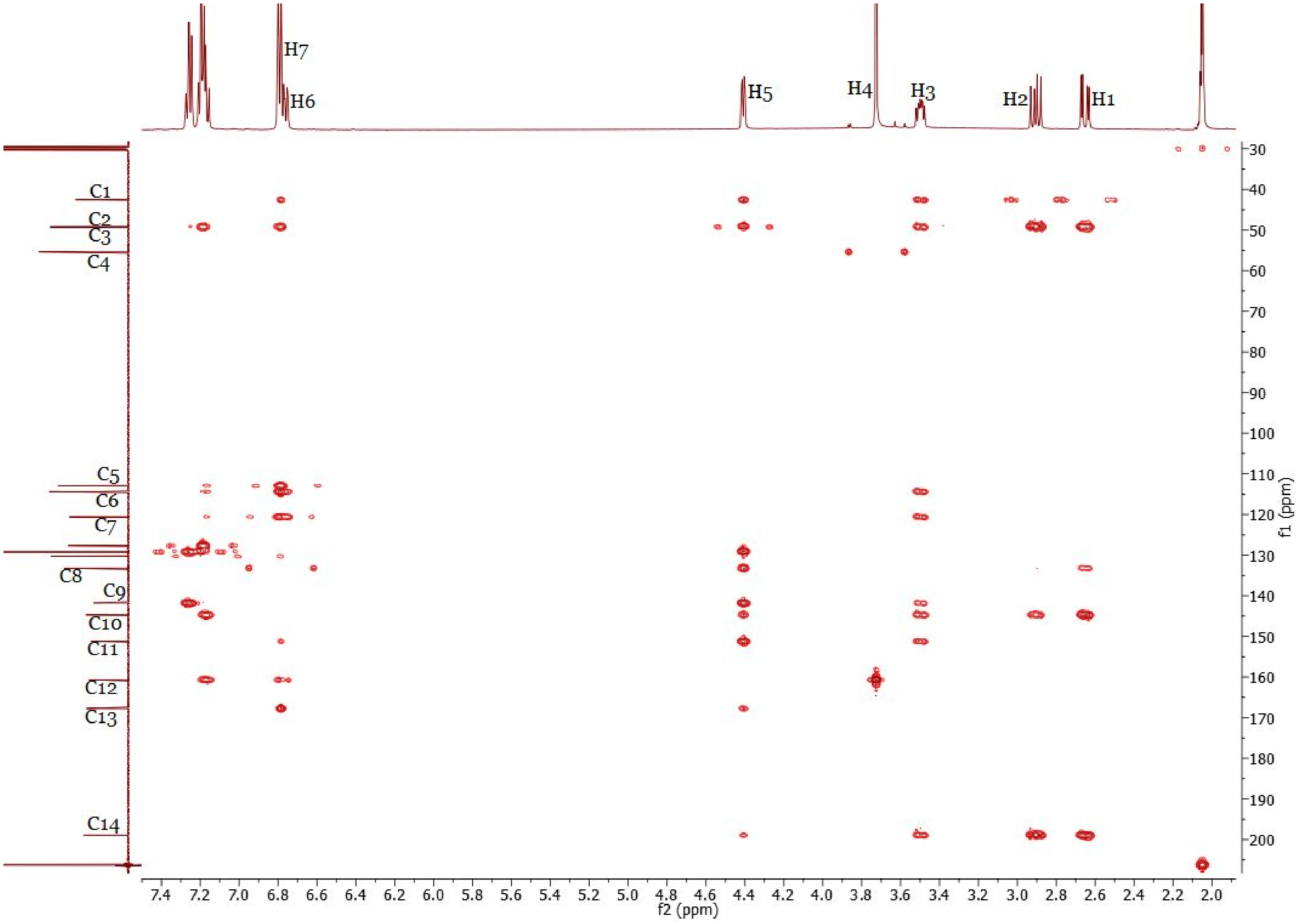

*Figure S79: HMBC spectrum of **7**.*

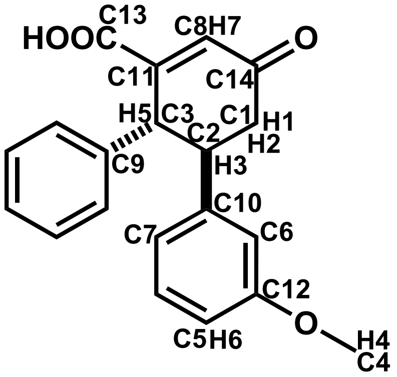

*Figure S80: 2D NMR observation of **7**.*

2D NMR observations of **7**:

Protons H1 and H2 are attached to carbon C1 forming CH_2 group. The group has connectivity to carbons C2, C3, C8, C10 and C14.

Proton H3 is attached to carbon C2 forming CH group. The group has connectivity to carbons C1, C3, C6, C7, C9, C10, C11 and C14.

Protons H4 are attached to carbon C4 forming CH_3 group. The group has connectivity to carbon C12.

Proton H5 is attached to carbon C3 forming CH group. The group has connectivity to carbons C1, C2, C8, C9, C10, C11, C13 and C14 (weak). The group has connectivity to non-substituted phenyl group, suggesting nearby location.

Proton H6 is attached to carbon C5 forming CH group. The group has connectivity only inside the substituted phenyl group.

Proton H7 is attached to carbon C8 forming CH group. The group has connectivity to C1 (weak), C3, C11 and C13.

IR spectroscopy of **7**

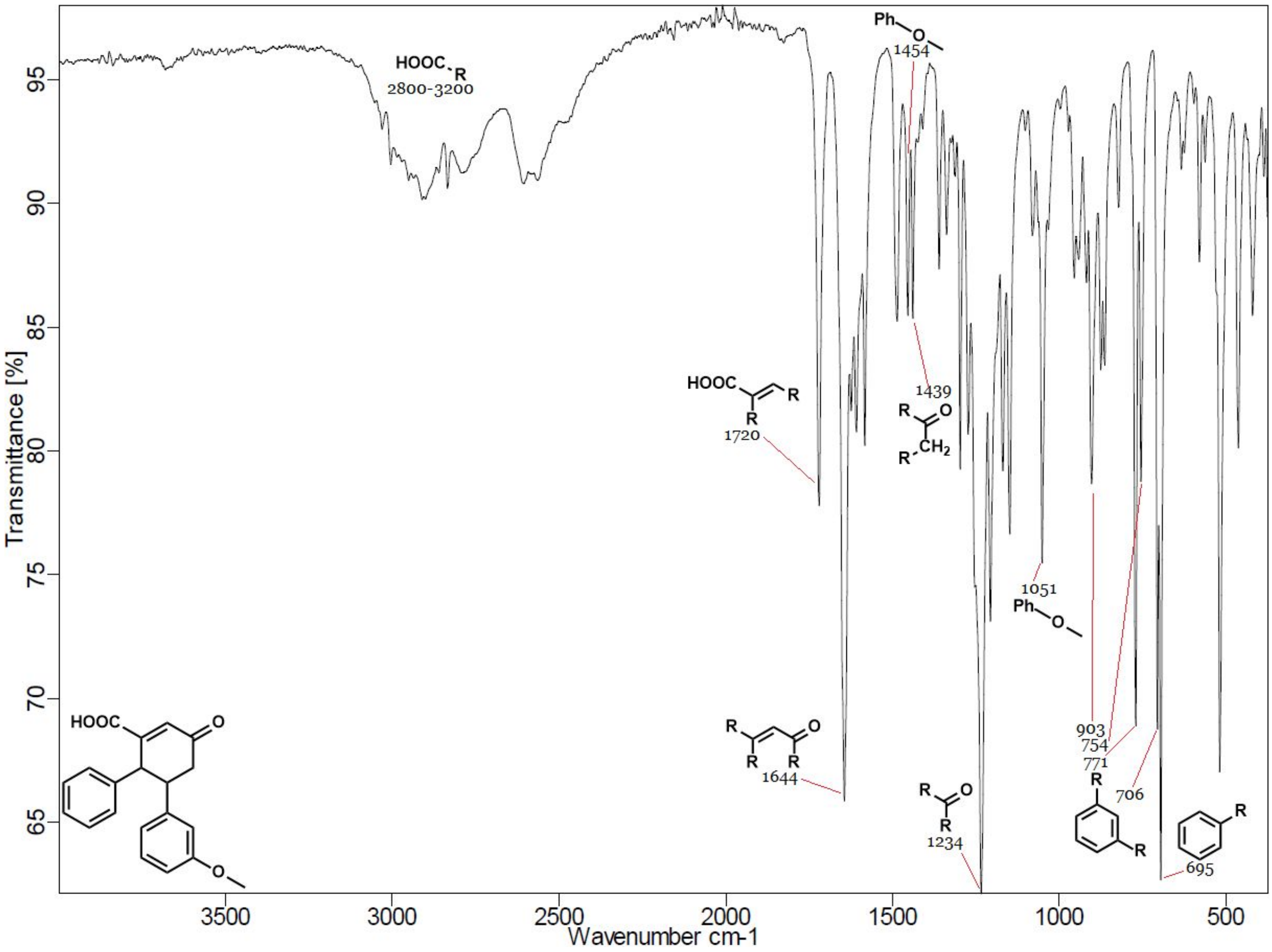

*Figure S81: IR spectrum of **7** (2800-3200 (broad) (R-COOH), 1720 (s) (C=C-ROOH), 1644 (s) (C=C-**CO**-R), 1454 (m) (R-O-CH_3), 1439 (m) (R-CO-$\mathbf{CH_2}$-R), 1234 (s) (R-**CO**-R), 1051 (m), 754 (s) (Ph-O-CH_3), 903 (m) (isolated H (Ph)), 771 (s), 706 (s) (3 adjacent H (Ph)), 695 (s) (5 adjacent H (Ph)) cm^{-1}).*

HRMS of **7**

HRMS (ESI-TOF) m/z: [**7**-H]- calculated for $C_{20}H_{17}O_4$ 321.1121; Found 321.1118; Error 0.949 ppm.

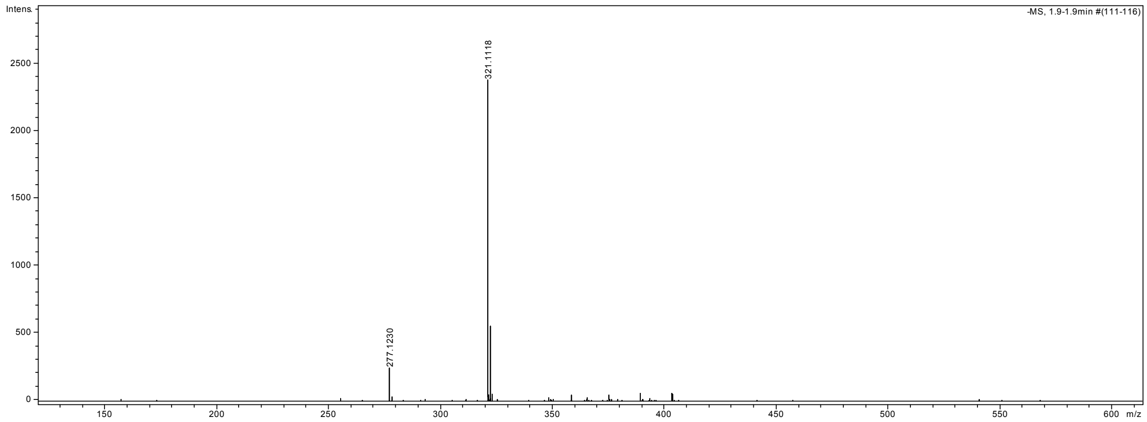

*Figure S82: ESI-TOF-MS of [**7**-H]- (peak: 321.1118 m/z, negative-ion mode).*

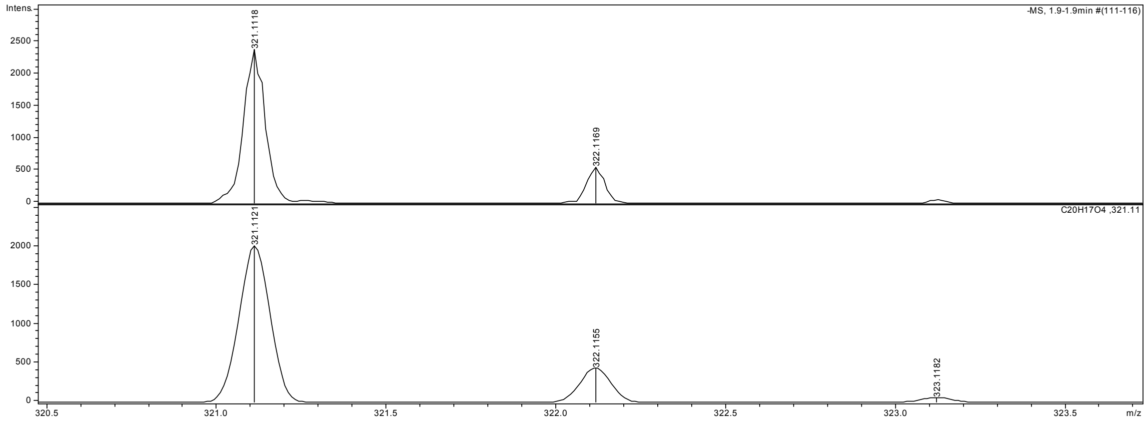

*Figure S83: Measured compound peak of [**7**-H]- (321.1118 m/z) at top, simulated peak ($C_{20}H_{17}O_4$) below.*

3.10 Spectroscopic data of 8

1D NMR of **8**

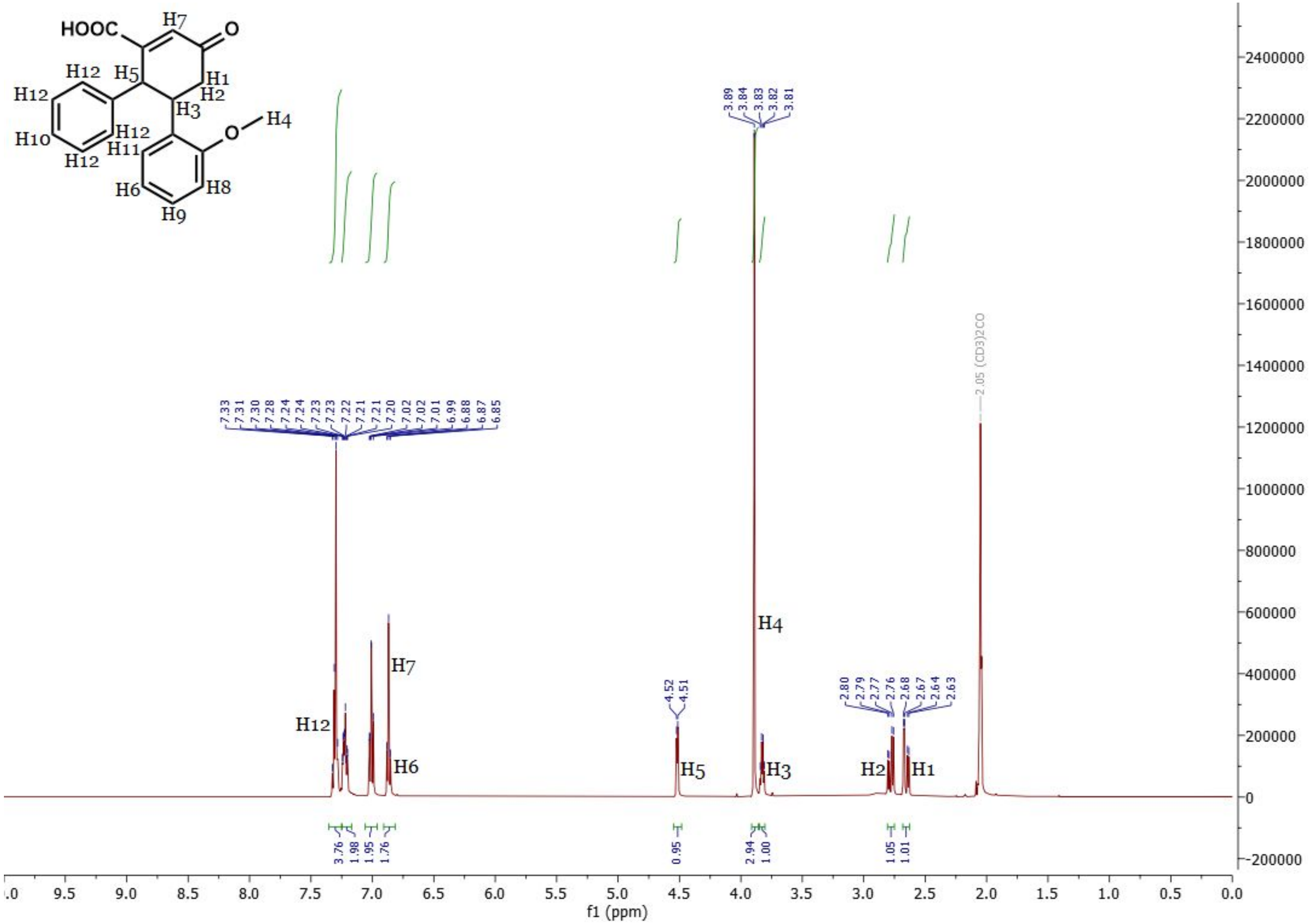

*Figure S84: ^{1}H NMR spectrum of **8** from full region (500 MHz, acetone-d6): δ 7.33-7.26 (m, 4H), 7.25-7.18 (m, 2H), 7.01 (m, 2H), 6.88 (m, 2H), 4.52 (d, J = 4.8 Hz, 1H), 3.89 (s, 3H), 3.83 (q, J = 5.0 Hz, 1H), 2.78 (dd, J = 17.0, 6.4 Hz, 1H), 2.65 (dd, J = 17.0, 5.3 Hz, 1H).*

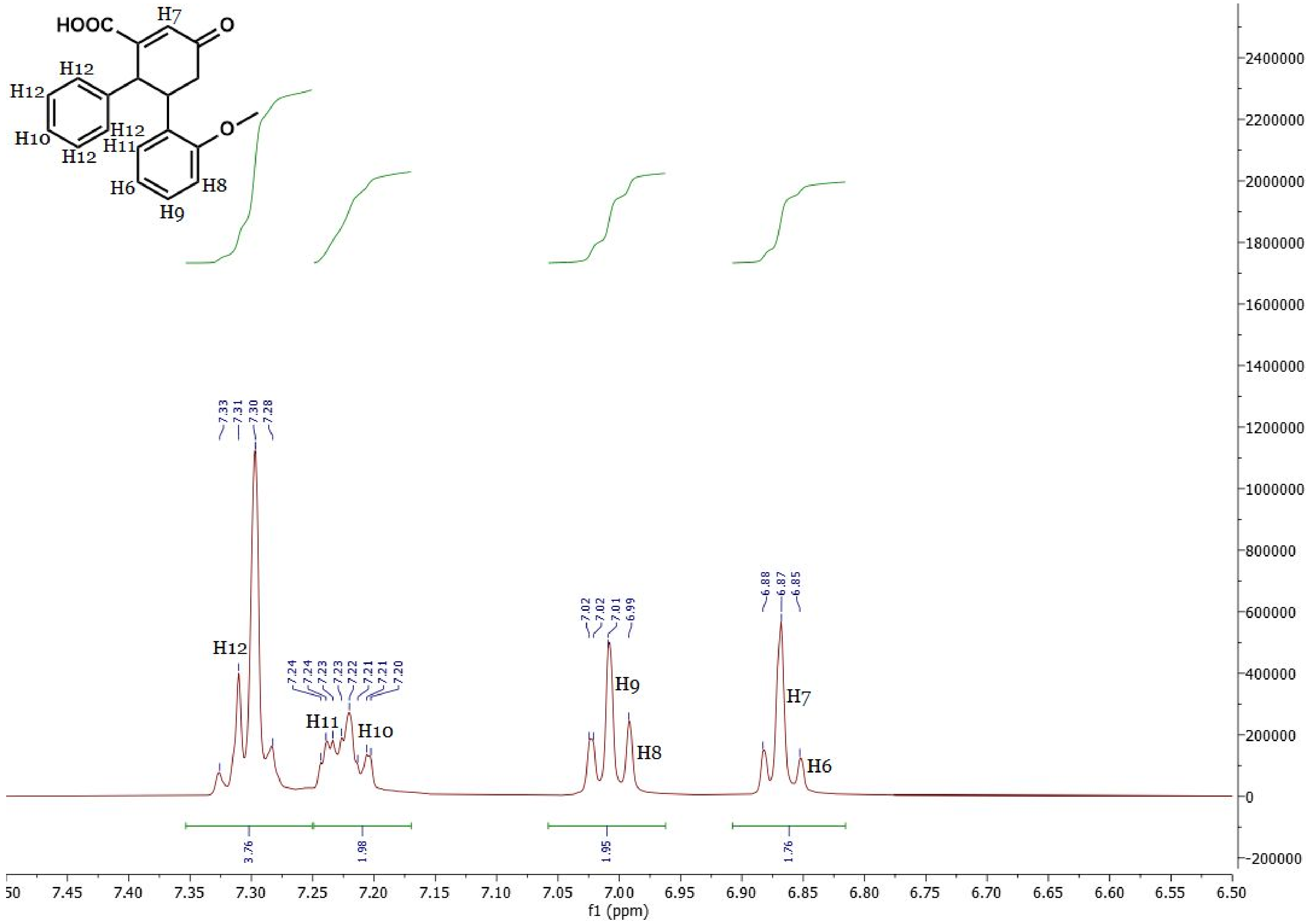

*Figure S85: ¹H NMR spectrum of **8** from aromatic region (500 MHz, acetone-d6): δ 7.33-7.26 (m, 4H), 7.25-7.18 (m, 2H), 7.01 (m, 2H), 6.88 (m, 2H).*

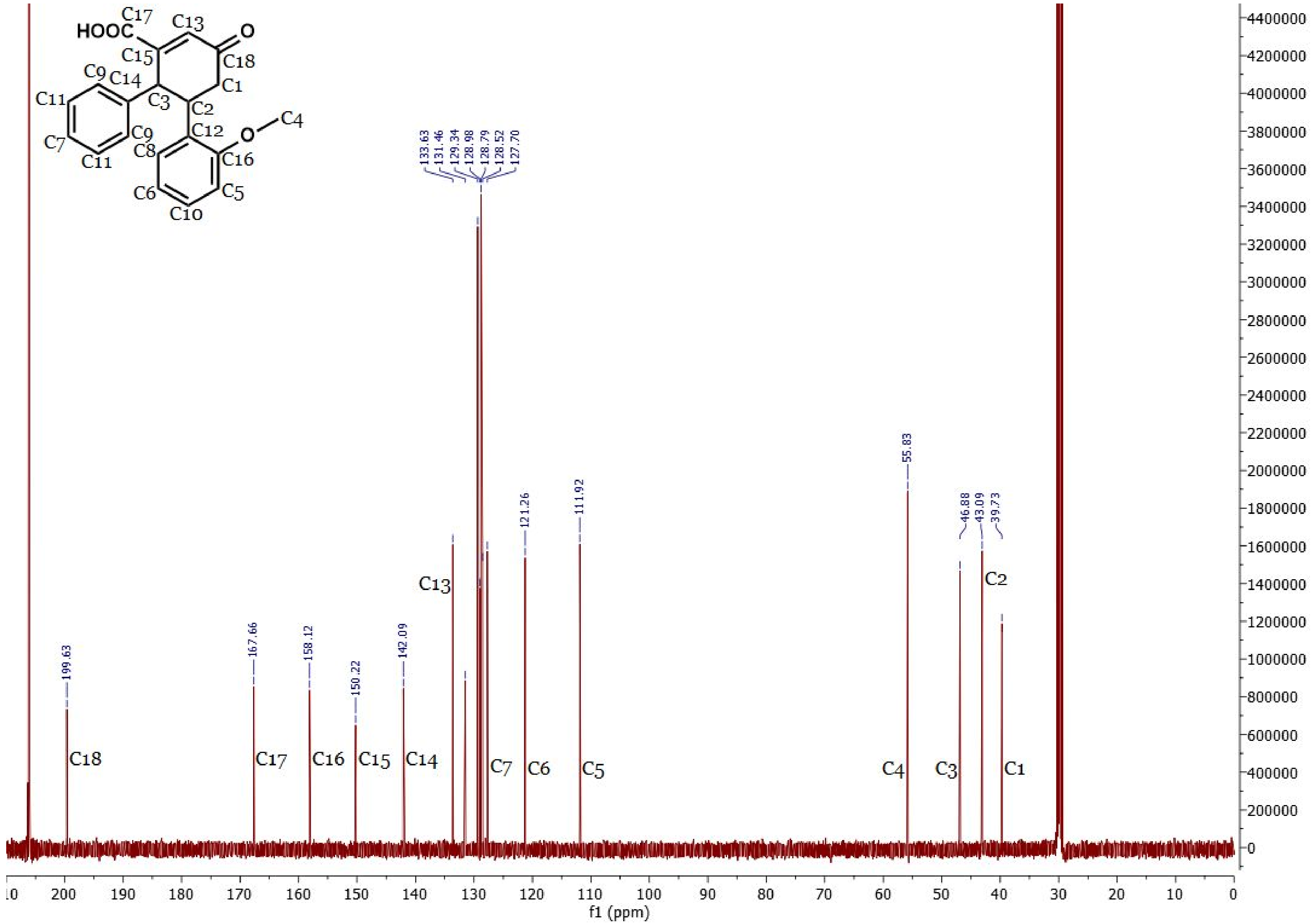

*Figure S86: $^{13}C\{^{1}H\}$ NMR spectrum of **8** from full region (125 MHz, acetone-d6): δ 199.63, 167.66, 158.12, 150.22, 142.09, 133.63, 131.46, 129.34, 128.98, 128.79, 128.52, 127.70, 121.26, 111.92, 55.83, 46.88, 43.09, 39.73.*

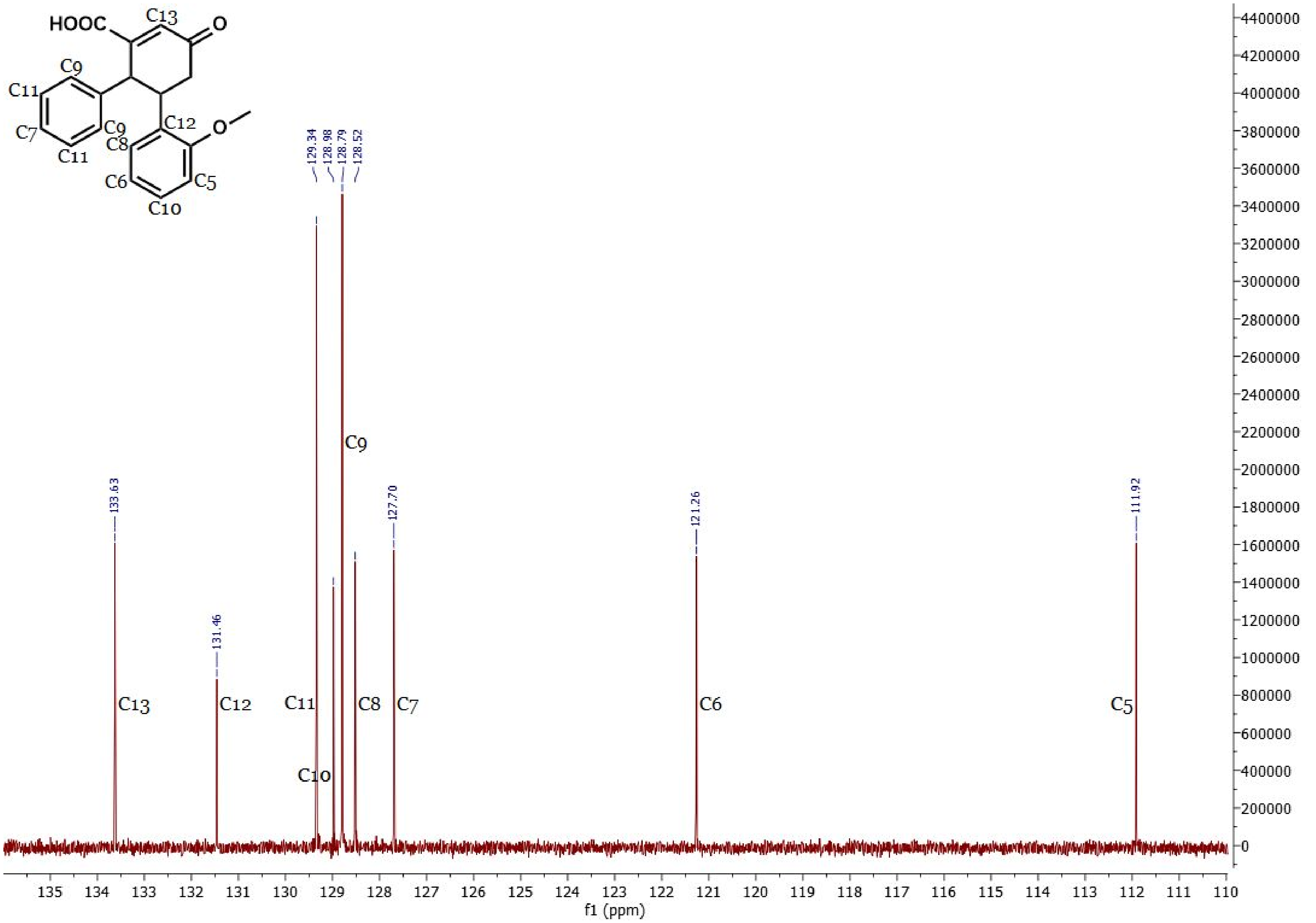

*Figure S87: $^{13}C\{^1H\}$ NMR spectrum of **8** from aromatic region (125 MHz, acetone-d6): δ 133.63, 131.46, 129.34, 128.98, 128.79, 128.52, 127.70, 121.26, 111.92.*

2D NMR of **8**

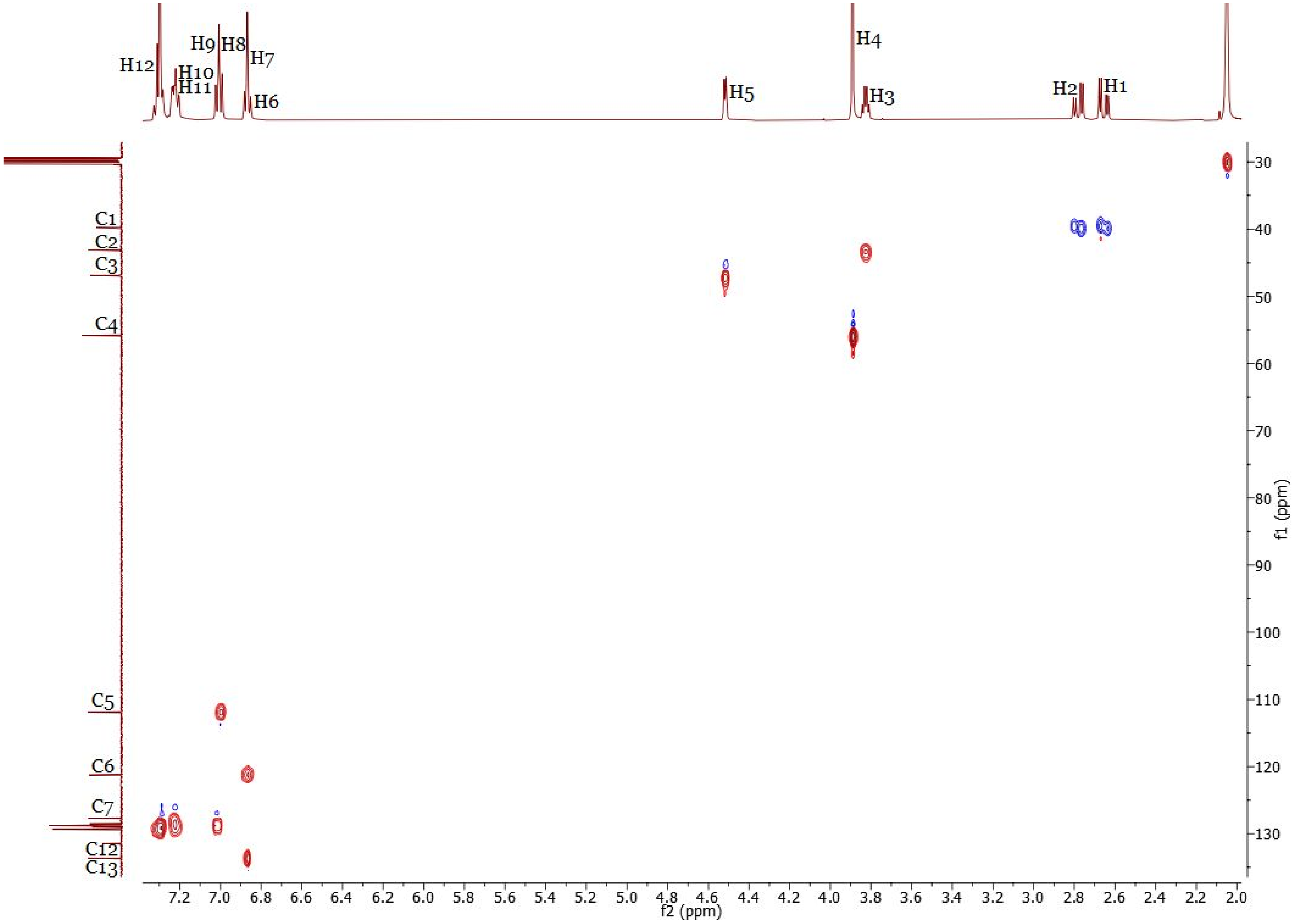

*Figure S88: HSQC spectrum of **8**.*

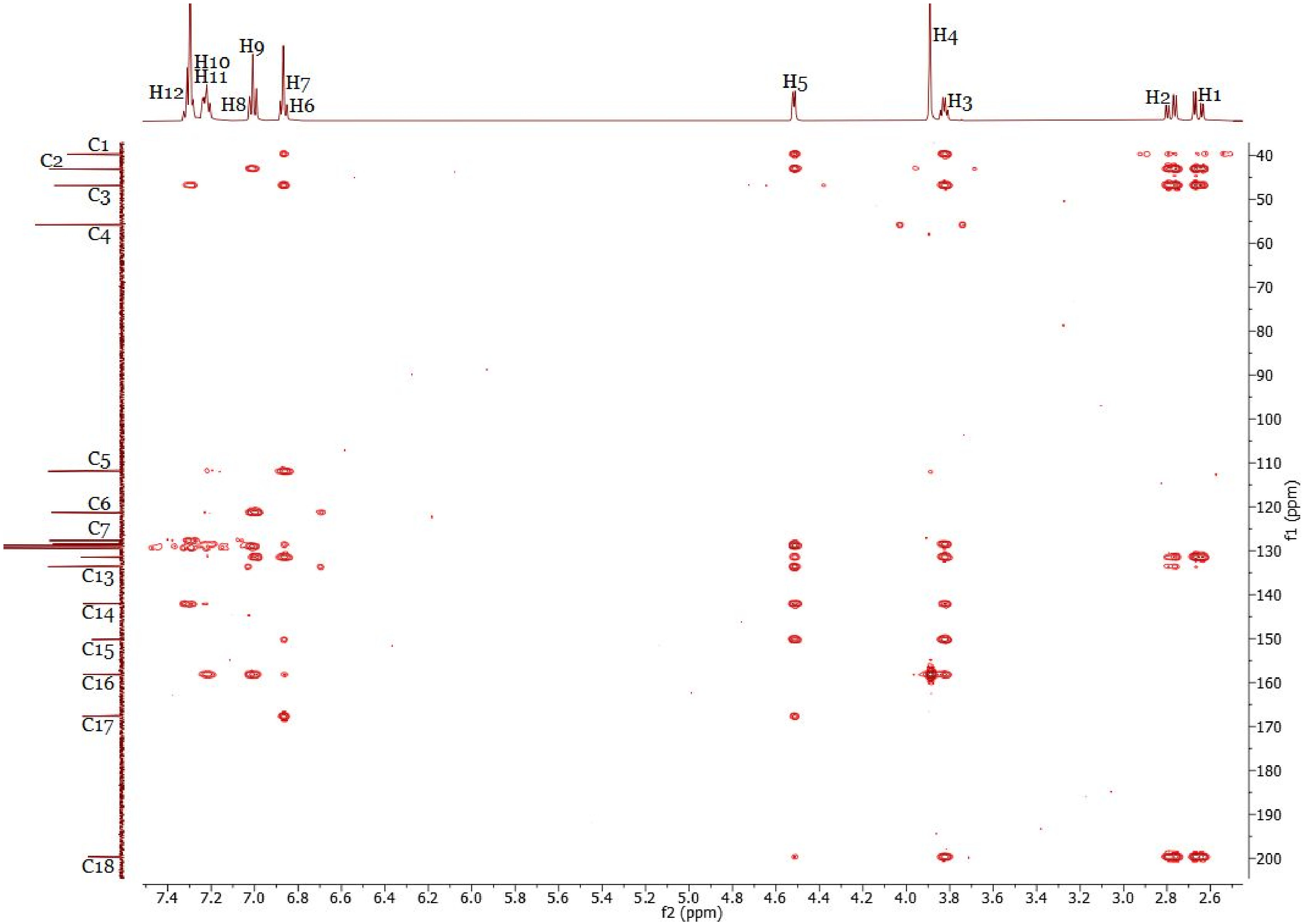

*Figure S89: HMBC spectrum of **8**.*

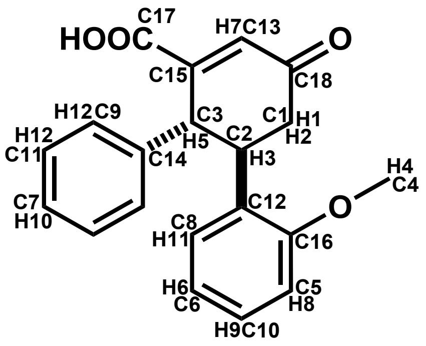

*Figure S90: 2D NMR observations of **8**.*

2D NMR observations of **8**:

Protons H1 and H2 are attached to carbon C1 forming CH_2 group. The group has connectivity to carbons C2, C3, C12, C13 and C18.

Proton H3 is attached to carbon C2 forming CH group. The group has connectivity to carbons C1, C3, C8, C12, C14, C15, C16 and C18.

Protons H4 are attached to carbon C4 forming CH_3 group. The group has connectivity to carbons C5 (weak) and C16.

Proton H5 is attached to carbon C3 forming CH group. The group has connectivity to carbons C1, C2, C9, C12, C13, C14, C15, C17 and C18.

Proton H6 is attached to carbon C6 forming CH group. The group has connectivity to carbons C5, C8 (weak), C12 and C16 (weak).

Proton H7 is attached to carbon C13 forming CH group. The group has connectivity to carbons C1, C5, C15 and C17.

Proton H8 is attached to carbon C5 forming CH group. The group has connectivity to carbons C6, C12 and C16.

Proton H9 is attached to carbon C10 forming CH group. The group has connectivity to carbons C6, C8 and C16.

Proton H10 is attached to carbon C7 forming CH group. The group has connectivity to carbons C9 and C11 and C14 (weak).

Proton H11 is attached to carbon C8 forming CH group. The group has connectivity to carbons C2, C6 and C16.

Protons H12 are attached to carbons C9 and C11 forming four CH groups. The group has connectivity to carbons C3, C7, 9, C11 and C13.

IR spectroscopy of **8**

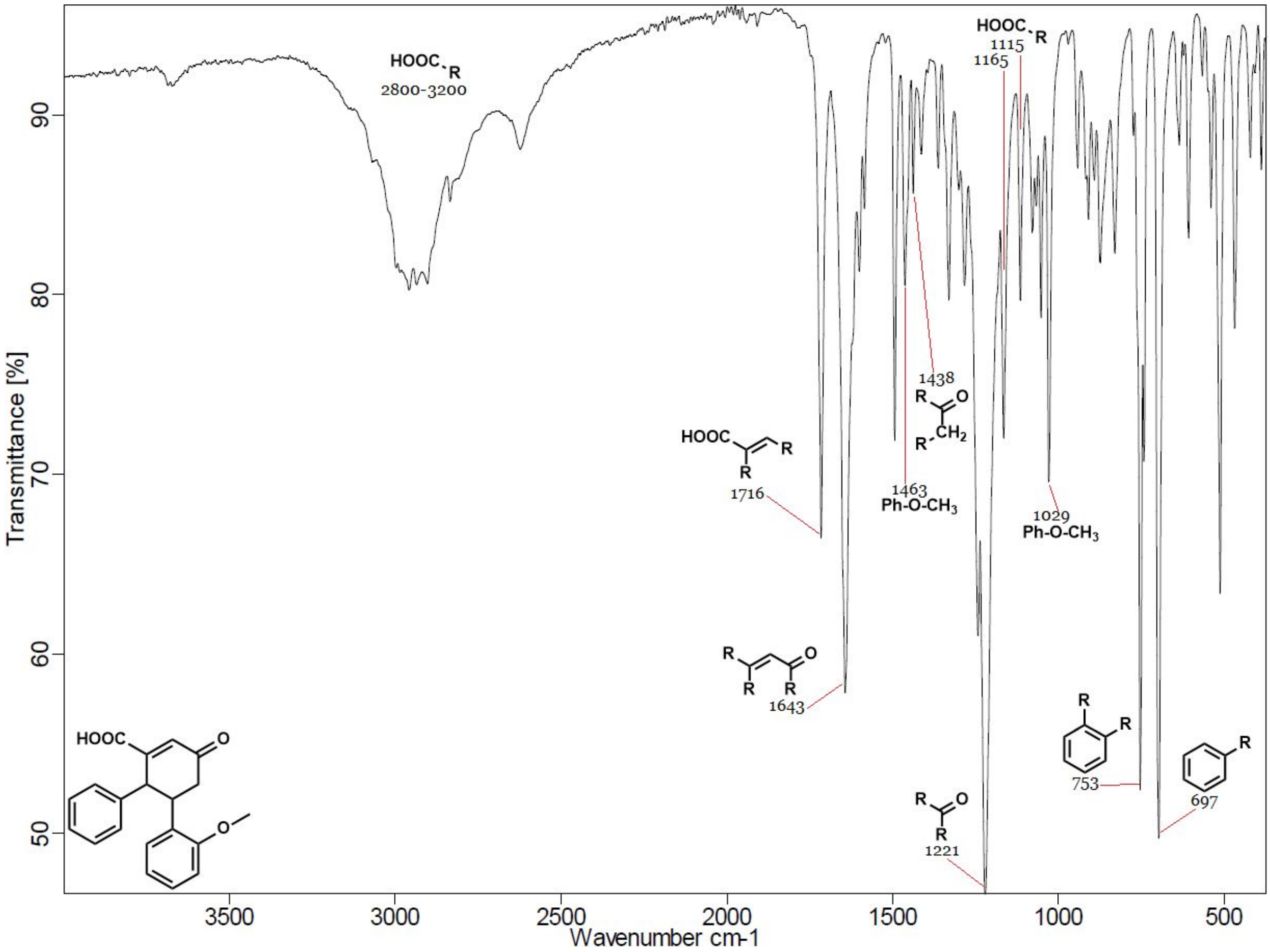

*Figure S91: IR spectrum of **8** (2800-3200 (broad) (R-COOH), 1716 (s) (C=C-ROOH), 1643 (s) (C=C-**CO**-R), 1463 (m), 1029 (m) (Ph-O-CH$_3$), 1438 (w) (R-CO-**CH$_2$**-R), 1221 (s) (R-**CO**-R), 1165 (m), 1115 (m) (R-COOH), 753 (s) (4 adjacent H (Ph)), 697 (s) (5 adjacent H (Ph)) cm^{-1}).*

HRMS of **8**

HRMS (ESI-TOF) m/z: [**8**-H]$^{-}$ calculated for $C_{20}H_{17}O_4$ 321.1121; Found 321.1119; Error 0.862 ppm.

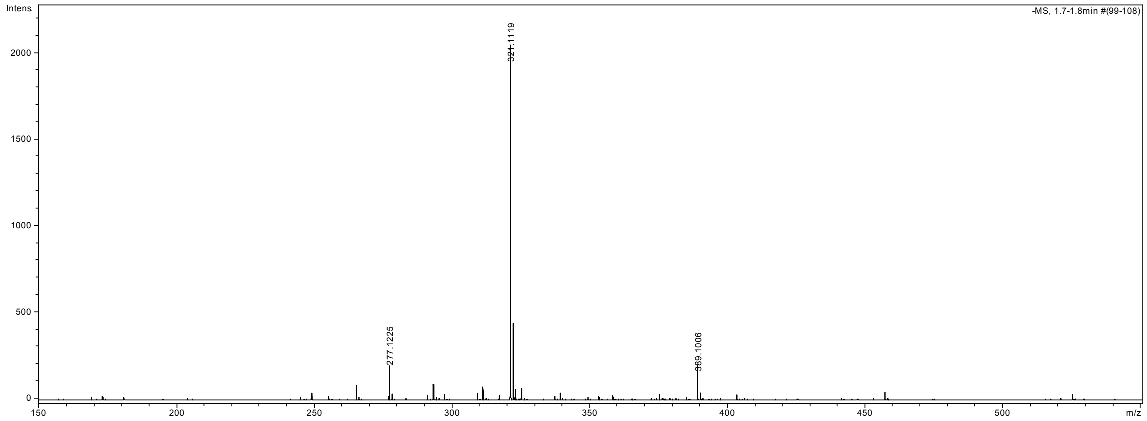

*Figure S92: ESI-TOF-MS of [**8**-H]$^{-}$ (peak: 321.1119 m/z, negative-ion mode).*

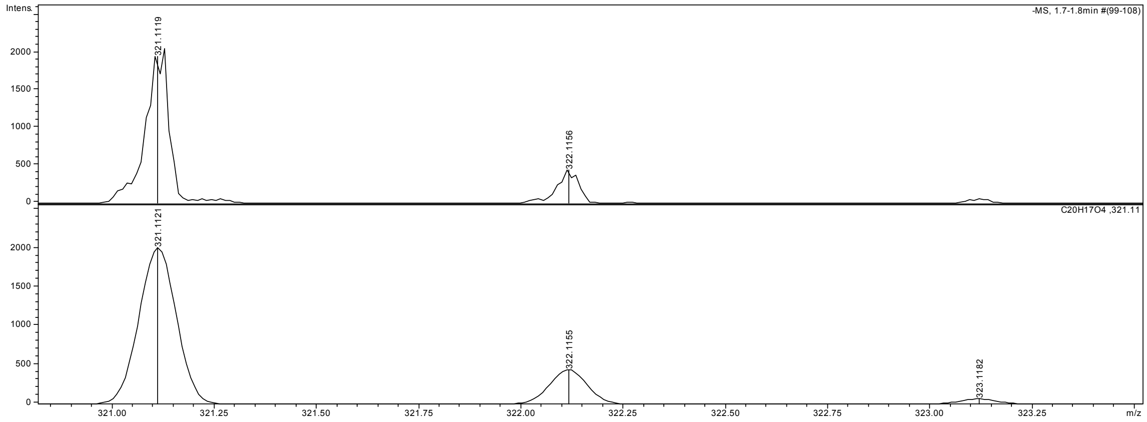

*Figure S93: Measured compound peak of [**8**-H]$^{-}$ (321.1119 m/z) at top, simulated peak ($C_{20}H_{17}O_4$) below.*

3.11 Spectroscopic data of 9

1D NMR of **9**

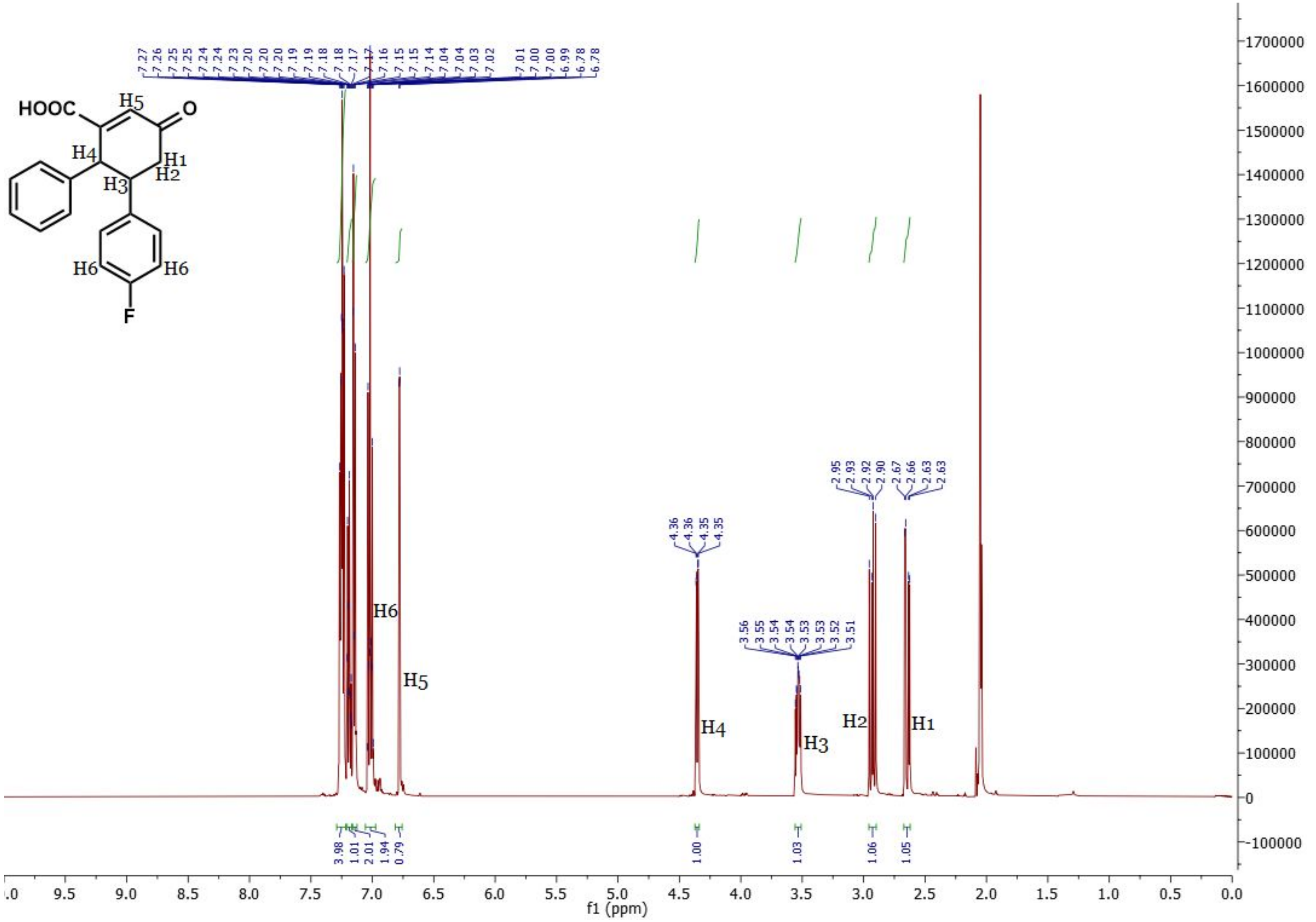

*Figure S94: ^{1}H NMR spectrum of **9** (500 MHz, acetone-d6): δ 7.28-7.22 (m, 4H), 7.21-7.17 (m, 1H), 7.16-7.12 (m, 2H), 7.04-6.99 (m, 2H), 6.78 (d, J = 2.0 Hz, 1H), 4.36 (dd, J = 7.4, 1.9 Hz, 1H), 3.53 (ddd, J = 10.1, 7.4, 4.4 Hz, 1H), 2.93 (dd, J = 16.3, 10.1 Hz, 1H), 2.65 (dd, J =16.3, 4.4 Hz, 1H).*

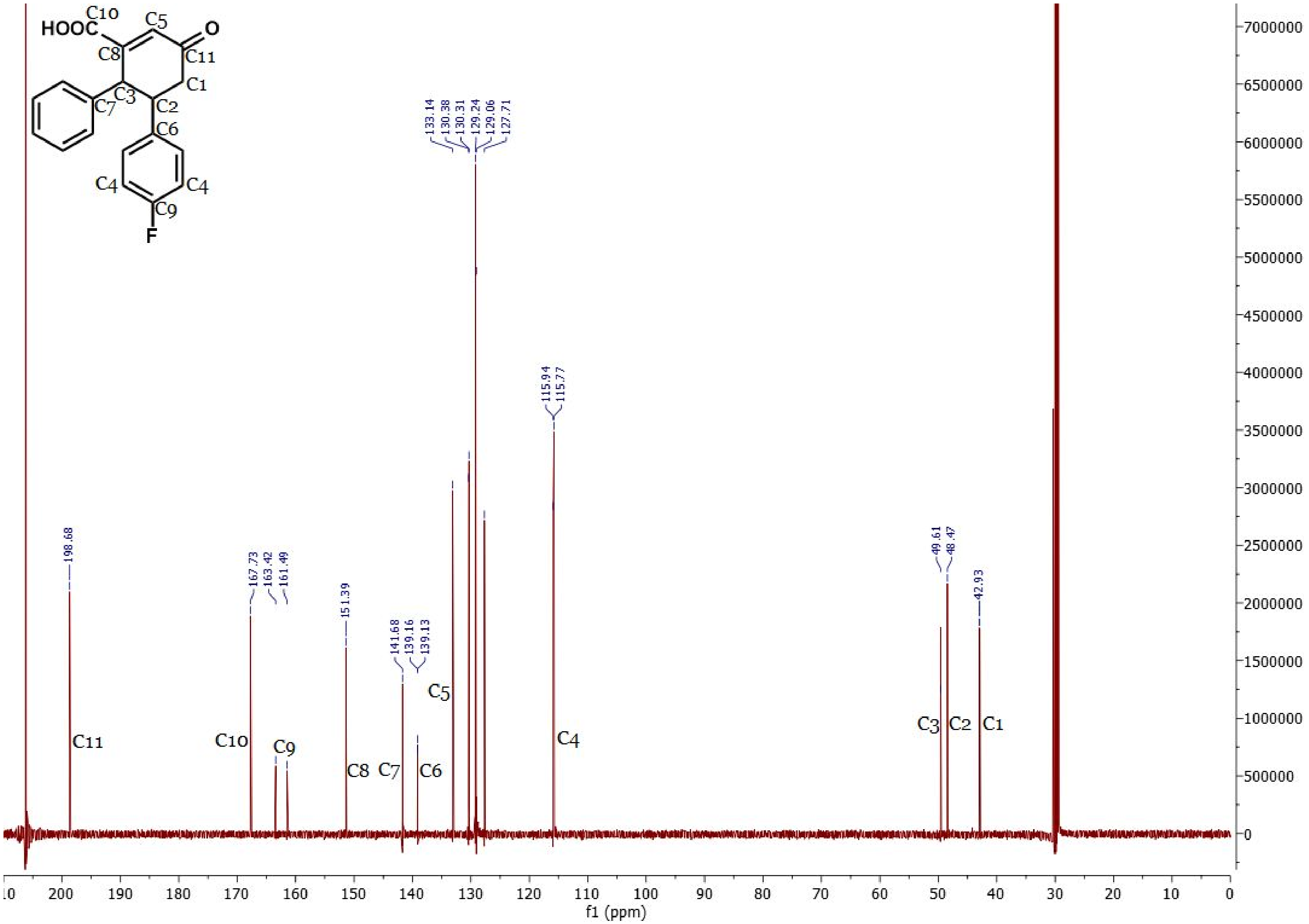

*Figure S95: $^{13}C\{^1H\}$ NMR spectrum of **9** (125 MHz, acetone-d6): δ 198.68, 167.73, 162.45 (d, $J_{C\text{-}F}$ = 243.6 Hz), 151.39, 141.68, 139.14 (d, $J_{C\text{-}F}$ = 3.2 Hz), 133,14, 130.34 (d, $J_{C\text{-}F}$ = 7.8Hz), 129.24, 129.06, 127.71, 115.85 (d, $J_{C\text{-}F}$ = 21.1Hz), 49.61, 48.47, 42.93.*

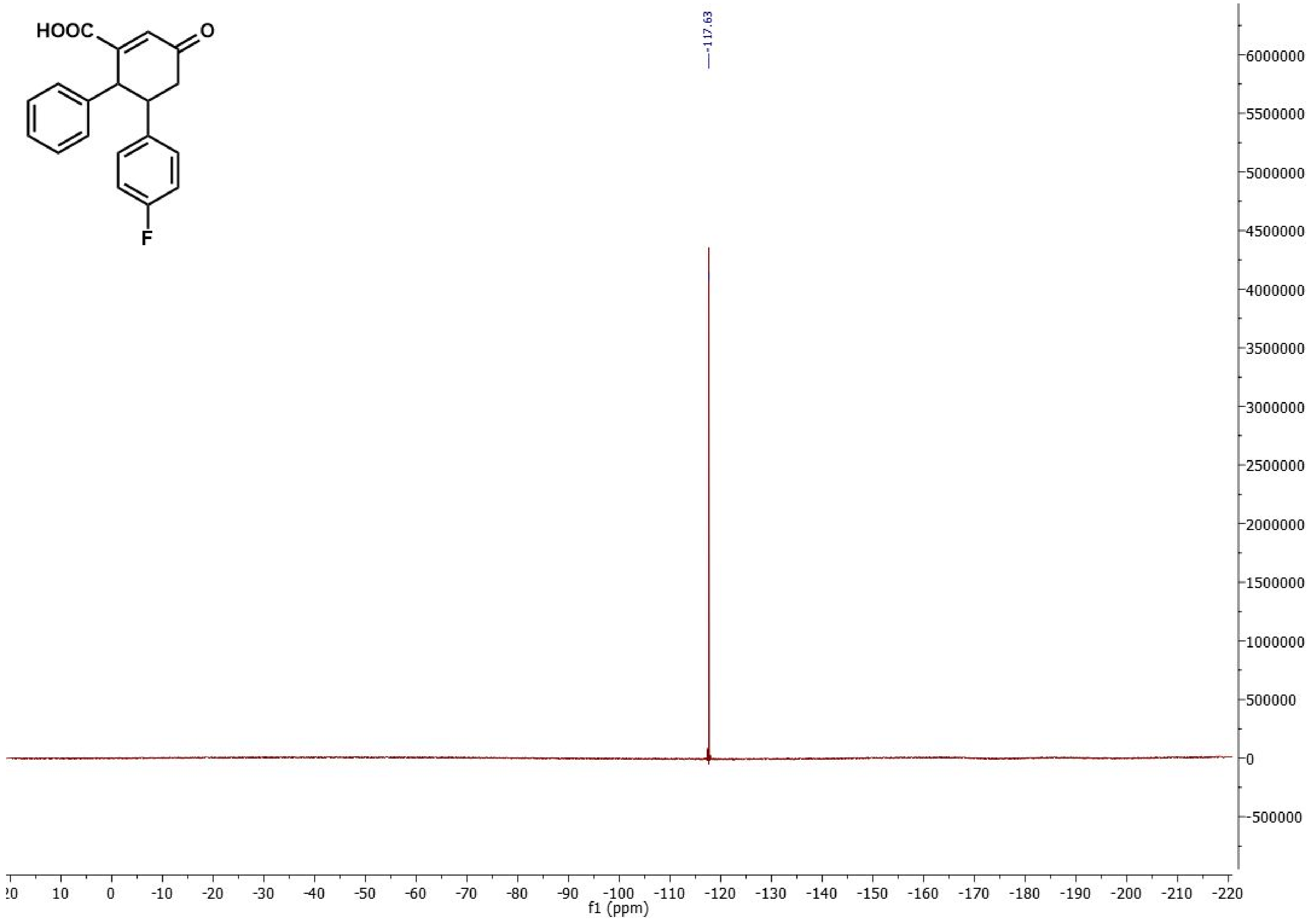

*Figure S96: ^{19}F NMR spectrum of **9** (470 MHz, acetone-d6): δ -117.63.*

2D NMR of **9**

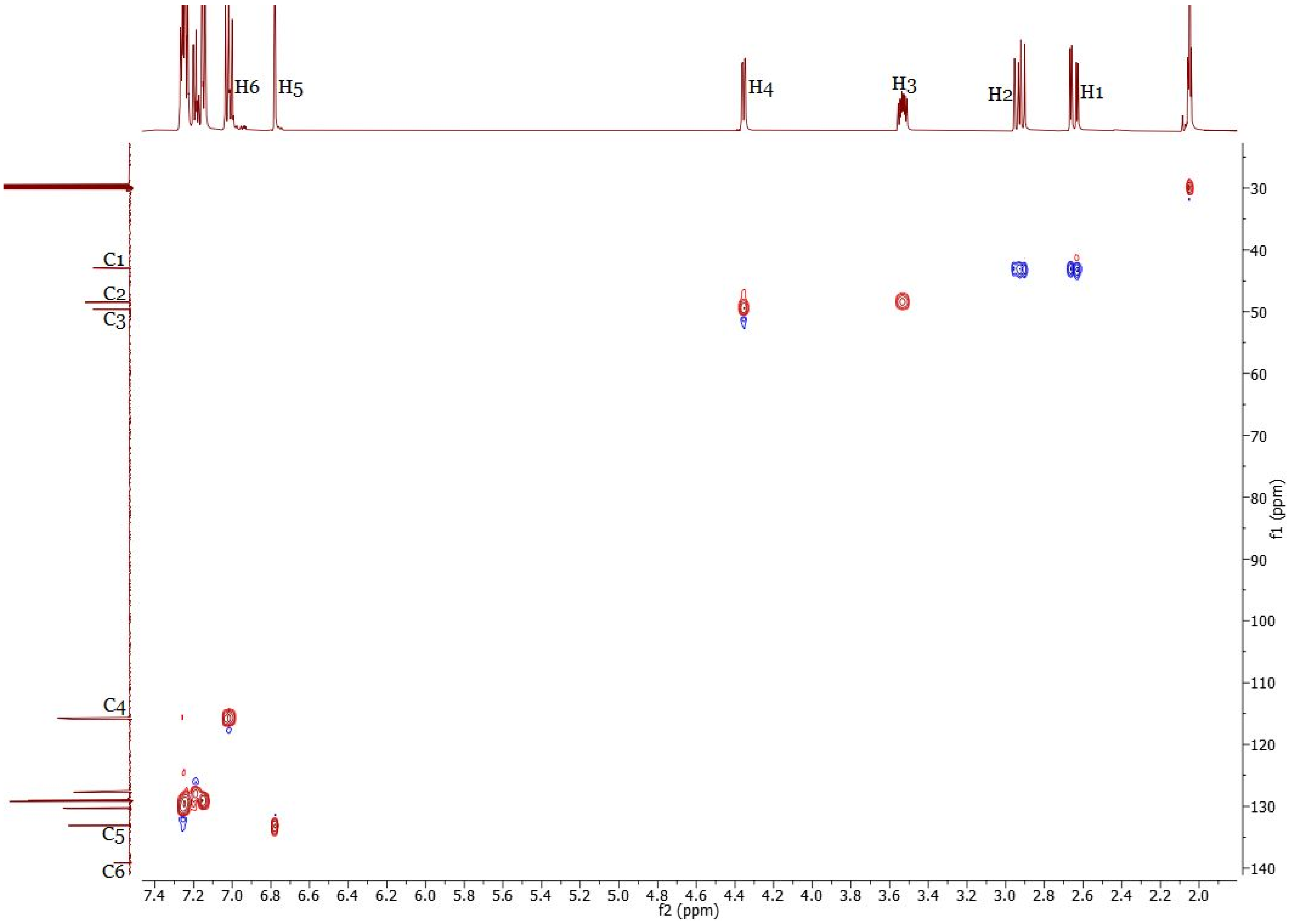

*Figure S97: HSQC spectrum of **9**.*

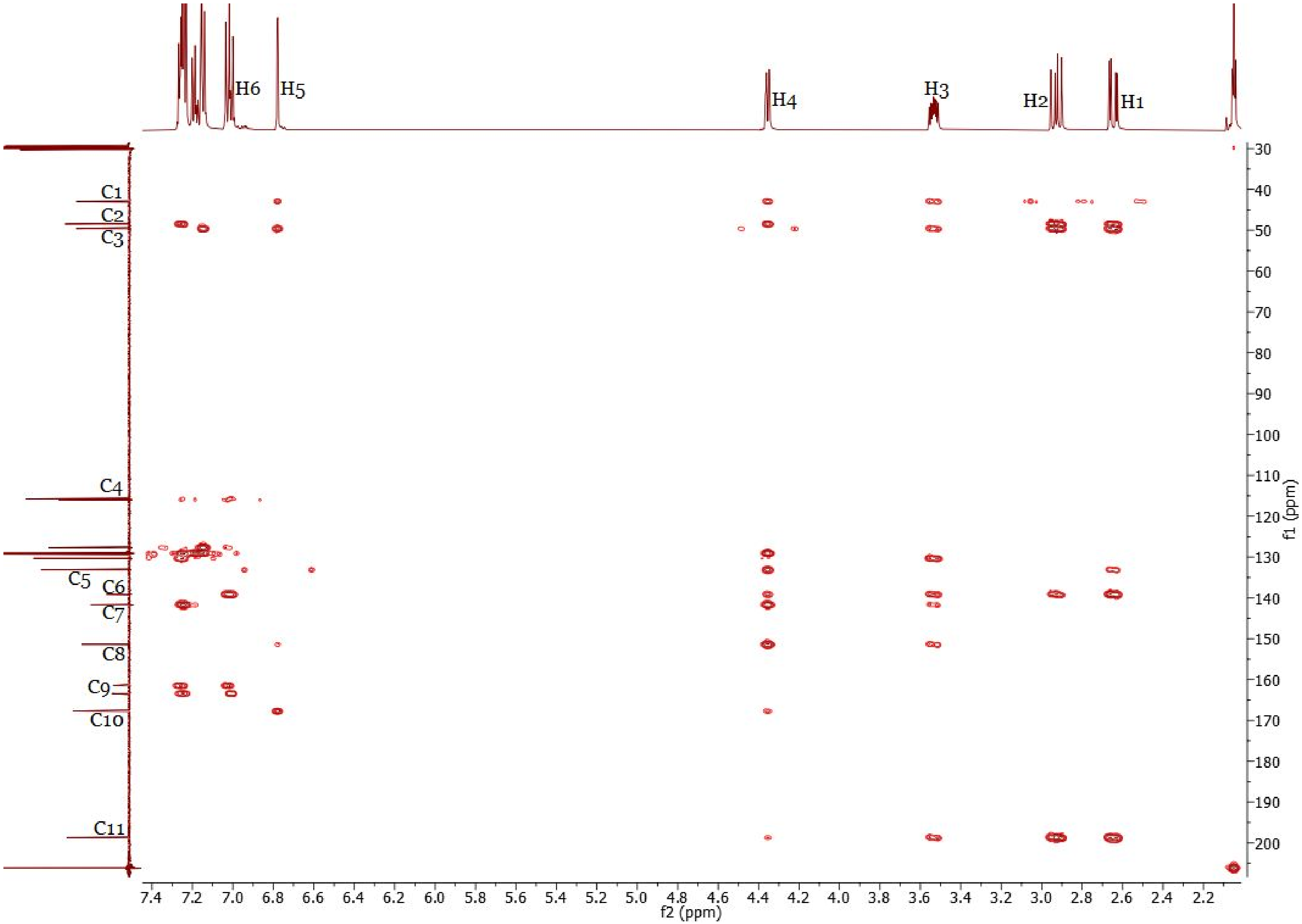

*Figure S98: HMBC spectrum of **9**.*

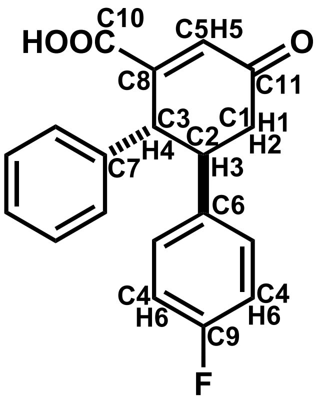

*Figure S99: 2D NMR observations of **9**.*

2D NMR observations of **9**:

Protons H1 and H2 are attached to carbon C1 forming CH_2 group. The group has connectivity to carbons C2, C3, C5 (weak), C6 and C11.

Proton H3 is attached to carbon C2 forming CH group. The group has connectivity to carbons C1, C3, C6, C7, C8 and C11. The group has connectivity inside substituted aromatic ring, suggesting nearby location.

Proton H4 is attached to carbon C3 forming CH group. The group has connectivity to carbons C1, C2, C5, C6, C7, C10 (weak) and C11 (weak). The group has connectivity inside aromatic ring, suggesting nearby location.

Proton H5 is attached to carbon C5 forming CH group. The group has connectivity to carbons C1, C3, C8 and C9.

Protons H6 are attached to carbon C4 forming two CH group. The groups have connectivity to carbons C6 and C9.

IR spectroscopy of **9**

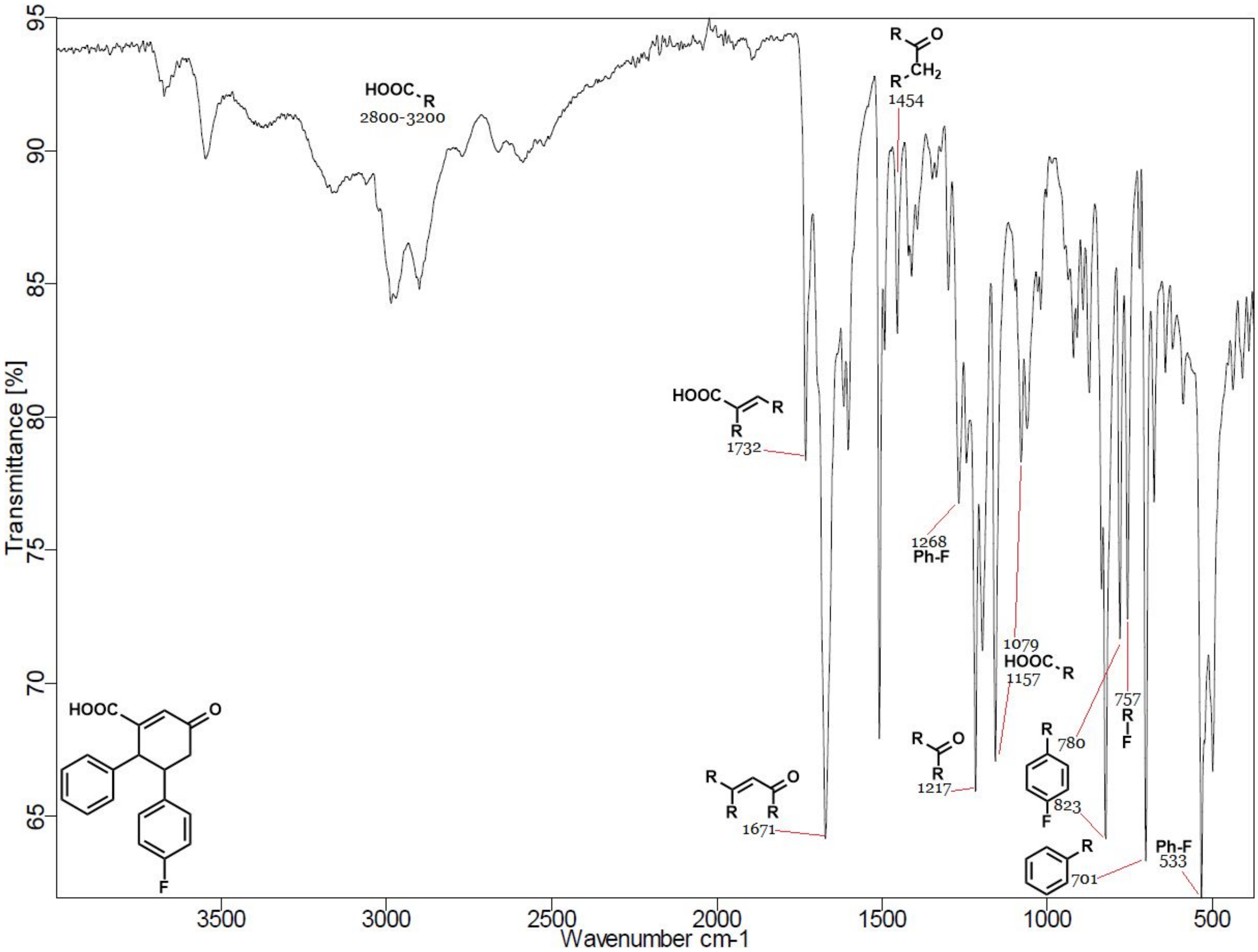

*Figure S100: IR spectrum of **9** (2800-3200 (broad) (R-COOH), 1732 (m) (C=C-ROOH), 1671 (s) (C=C-**CO**-R), 1454 (m) (R-CO-**CH$_2$**-R), 1268 (m), 533 (s) (Ph-F), 1217(s) (R-**CO**-R), 1154 (s), 1079 (m) (R-COOH), 823 (s), 780 (s) (2 adjacent H (R-Ph-p-F)), 757 (s) (C-F), 701 (s) (5 adjacent H (Ph)) cm^{-1}).*

HRMS of **9**

HRMS (ESI-TOF) m/z: [**9**-H]- calculated for $C_{19}H_{14}O_3F$ 309.0921; Found 309.0919; Error 0.917 ppm.

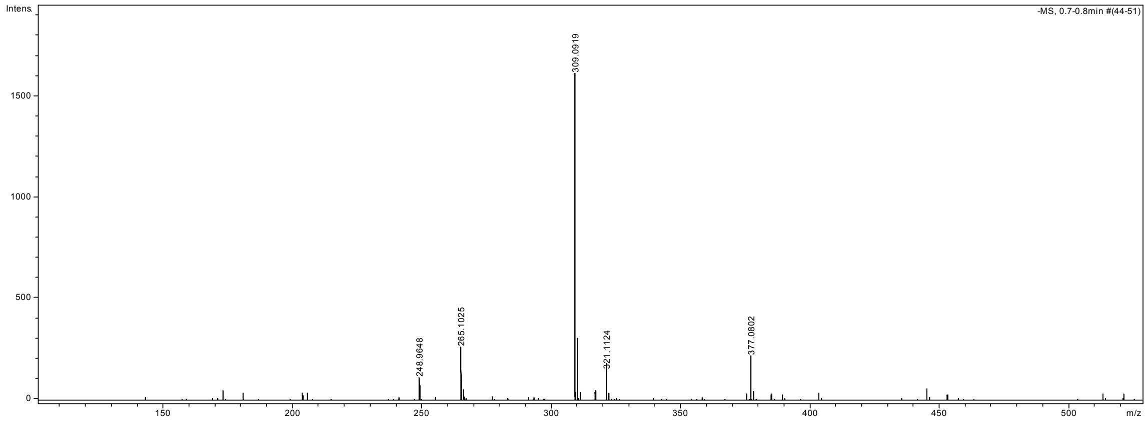

*Figure S101: ESI-TOF-MS of [**9**-H]- (peak: 309.0919 m/z, negative-ion mode).*

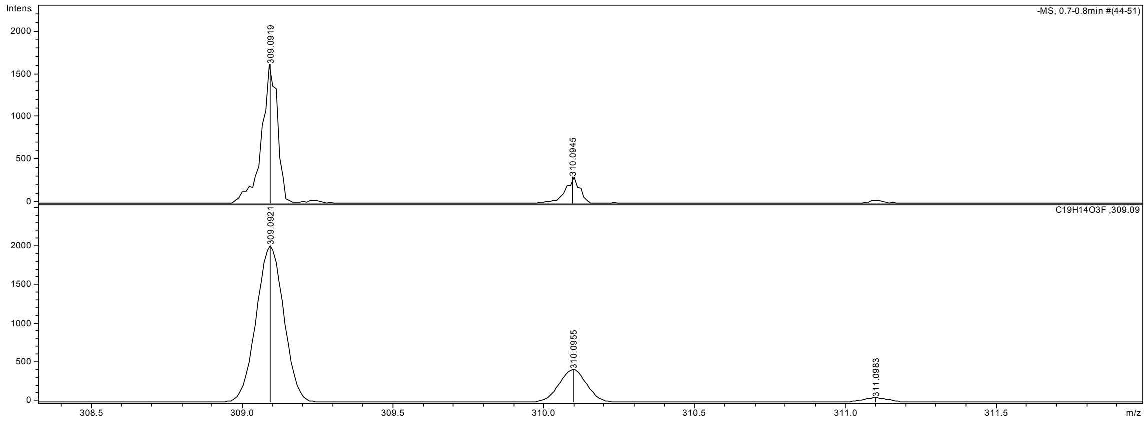

*Figure S102: Measured compound peak of [**9**-H]- (309.0919 m/z) at top, simulated peak ($C_{19}H_{14}O_3F$) below.*

3.12 Spectroscopic data of 10

1D NMR of **10**

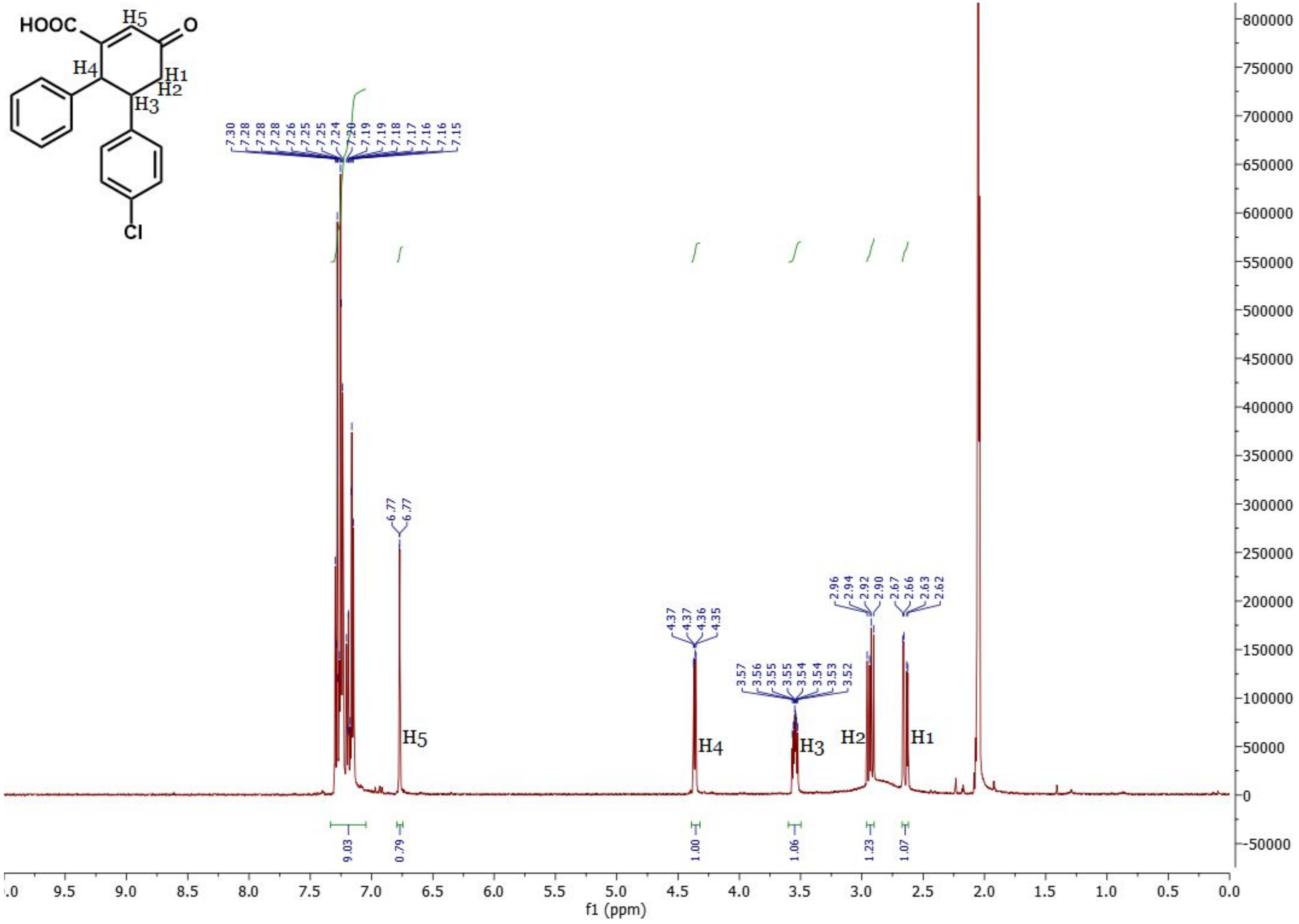

*Figure S103: ^{1}H NMR spectrum of **10** (500 MHz, acetone-d6): δ 7.30-7.14 (m, 9H), 6.77 (d, J = 2.0 Hz, 1H), 4.36 (dd, J = 7.4, 2.0 Hz, 1H), 3.55 (ddd, J = 10.1, 7.5, 4.4 Hz, 1H), 2.93 (dd, J = 16.3, 10.1 Hz, 1H) 2.65 (dd, J = 16.3, 4.4 Hz, 1H).*

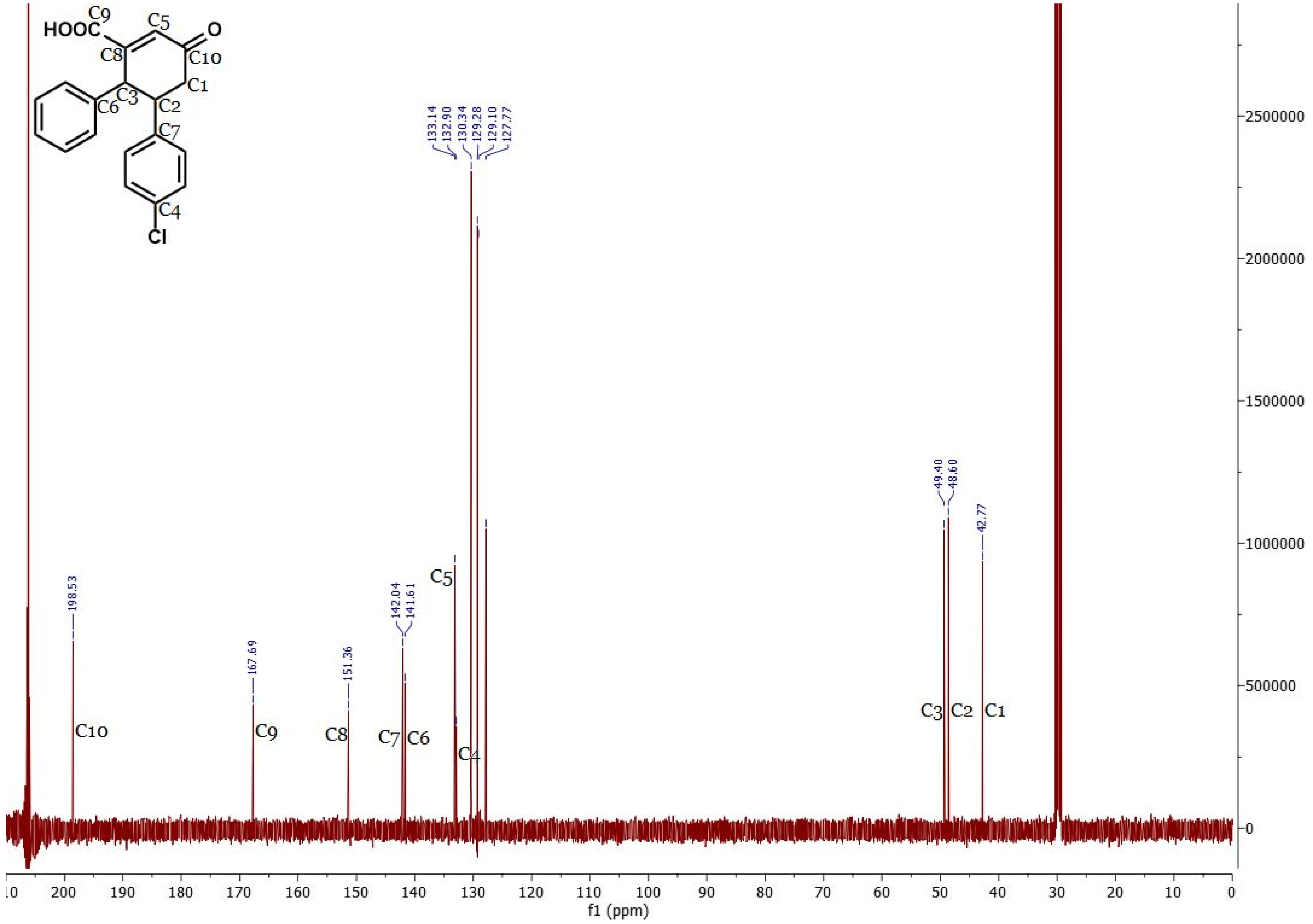

*Figure S104: $^{13}C\{^1H\}$ NMR spectrum of **10** (125 MHz, acetone-d6): δ 198.53, 167.69, 151.36, 142.04, 141.61, 133.14, 132.90, 130.34, 129.28, 129.10, 127.77, 49.40, 48.60, 42.77.*

2D NMR of **10**

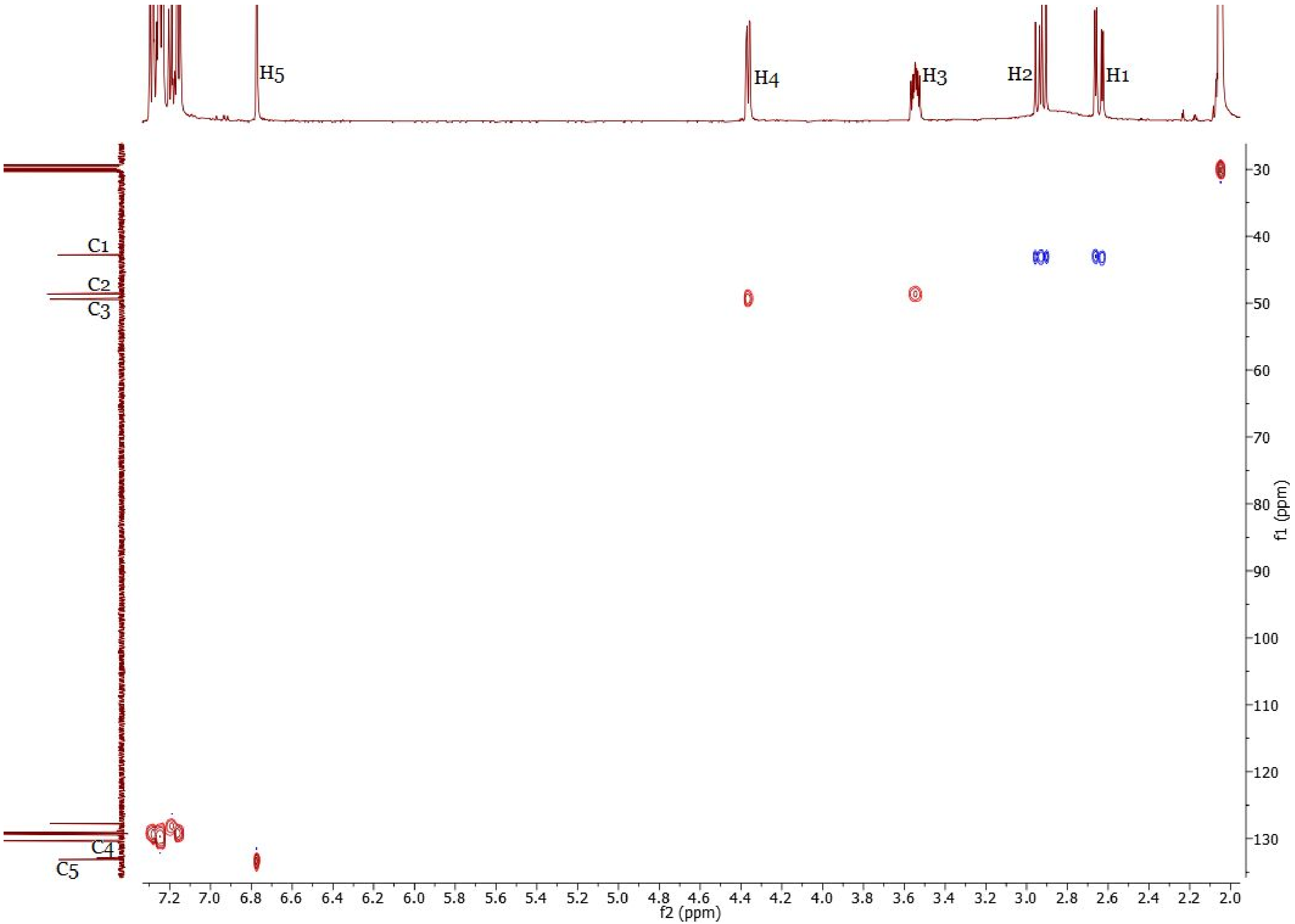

*Figure S105: HSQC spectrum of **10**.*

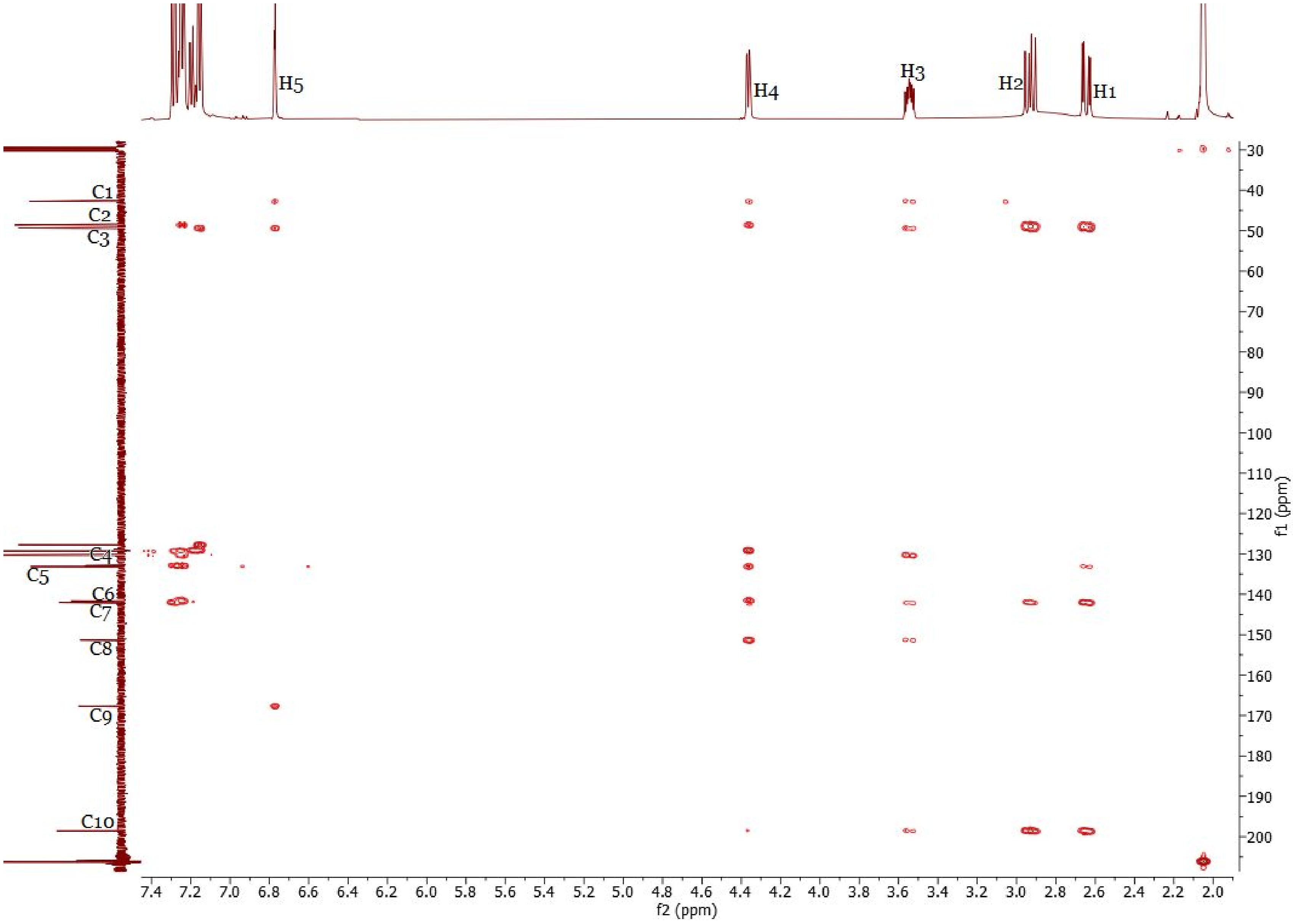

*Figure S106: HMBC spectrum of **10**.*

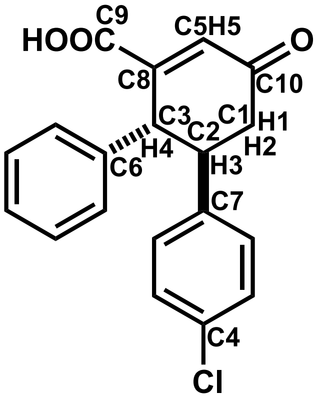

Figure S107: 2D NMR observations of ***10****.*

2D NMR observations of **10**:

Protons H1 and H2 are attached to carbon C1 forming CH_2 group. The groups has connectivity to carbons C2, C3, C5 (weak), C7 and C10.

Proton H3 is attached to carbon C2 forming CH group. The group has connectivity to carbons C1, C3, C7, C8 and C10. The group has connectivity inside the substituted aromatic group, suggesting nearby location.

Proton H4 is attached to carbon C3 forming CH group. The group has connectivity to carbons C1, C2, C5, C6, C7 (weak), C8 and C10 (very weak). The group has connectivity inside aromatic group, suggesting nearby location.

Proton H5 is attached to carbon C5 forming CH group. The group has connectivity to carbons C1, C3 and C9.

IR spectroscopy of **10**

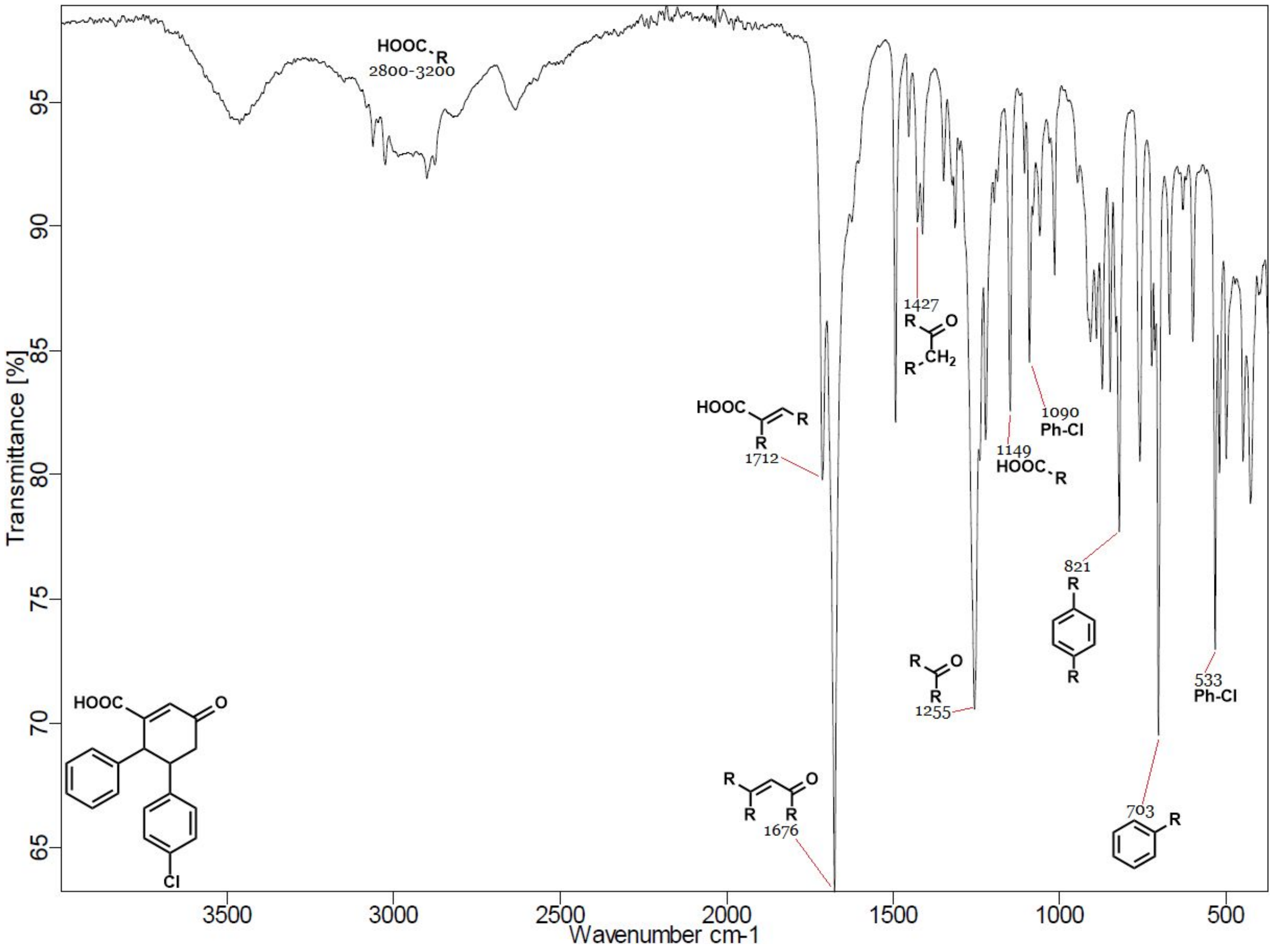

Figure S108: IR spectrum of ***10*** *(2800-3200 (broad), 1149 (m) (R-COOH), 1732 (s) (C=C-ROOH), 1676 (s) (C=C-**CO**-R), 1427 (m) (R-CO-**CH₂**-R), 1255(s) (R-**CO**-R), 1090 (m), 533 (s) (Ph-Cl), 821 (s), 780 (s) (2 adjacent H (R-Ph-p-Cl)), 703 (s) (5 adjacent H (Ph)) cm^{-1}).*

HRMS of **10**

HRMS (ESI-TOF) m/z: [**10**-H]- calculated for $C_{19}H_{14}O_3Cl$ 325.0626; Found 325.0625; Error 0.280 ppm.

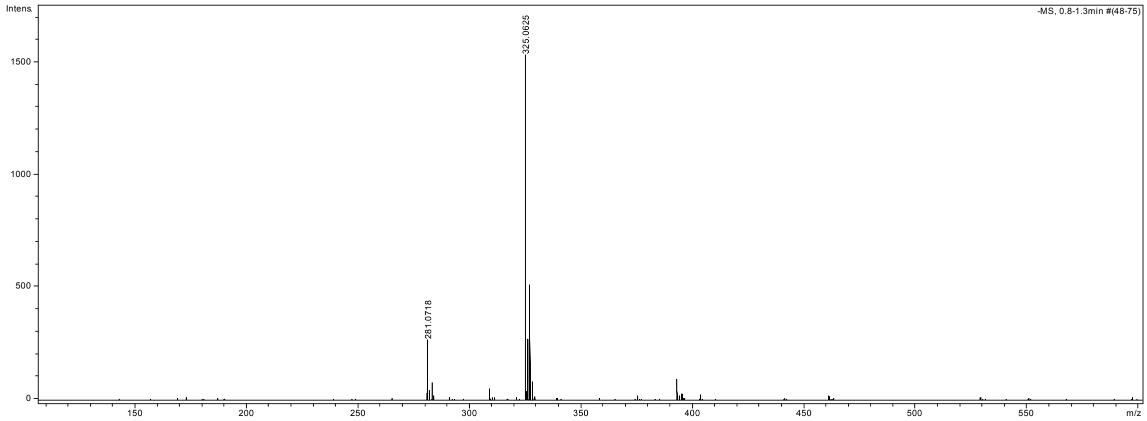

*Figure S109: ESI-TOF-MS of [**10**-H]- (peak: 325.0625 m/z, negative-ion mode).*

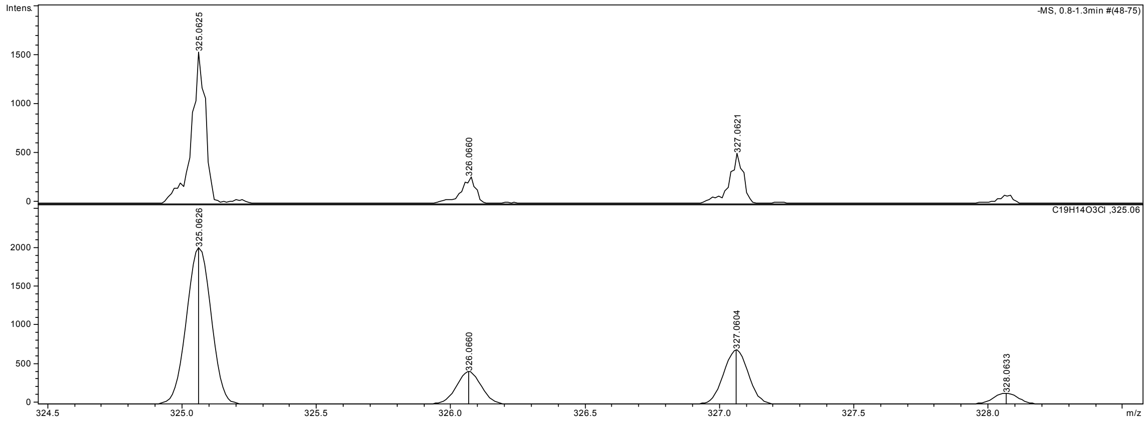

*Figure S110: Measured compound peak of [**10**-H]- (325.0625 m/z) at top, simulated peak ($C_{19}H_{14}O_3Cl$) below.*

3.13 Spectroscopic data of 11

1D NMR of **11**

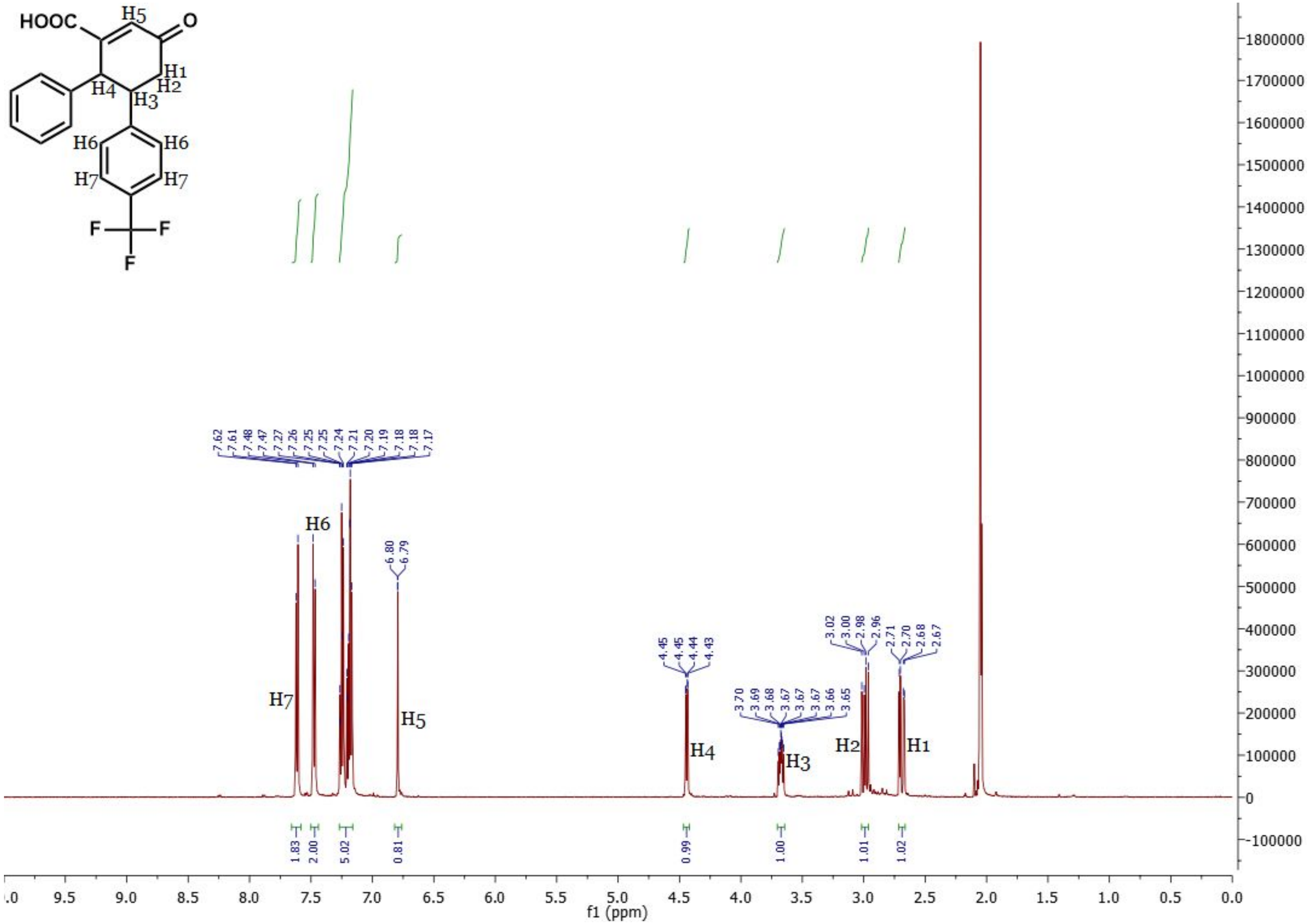

*Figure S111: ^{1}H NMR spectrum of **11** (500 MHz, acetone-d6): δ 7.61 (d, J = 8.1 Hz, 2H), 7.47 (d, J = 8.1 Hz, 2H), 7.27-7.16 (m, 5H), 6.79 (d, J = 2.0 Hz, 1H), 4.44 (dd, J = 7.5, 1.9 Hz, 1H), 3.67 (ddd, J = 10.3, 7.5, 4.4 Hz, 1H), 2.99 (dd, 16.4, 10.1 Hz, 1H), 2.69 (dd, J = 16.3, 4.4 Hz, 1H).*

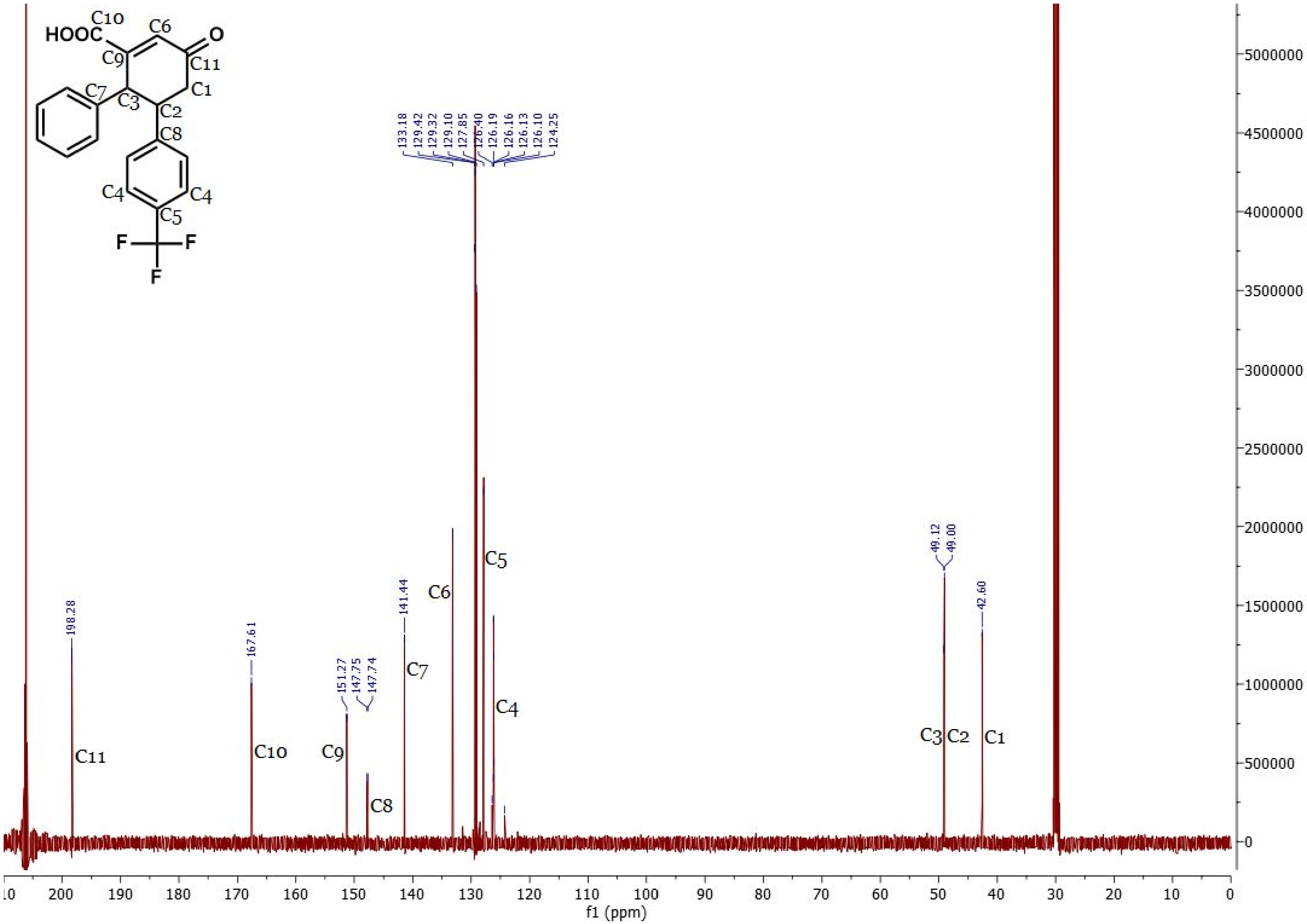

*Figure S112: $^{13}C\{^{1}H\}$ NMR spectrum of **11** (125 MHz, acetone-d6): δ 198.28, 167.61, 151.27, 147.74 (d, $J_{C\text{-}F}$ = 1.4 Hz), 141.44, 133.18, 129.42, 129.32, 129.10, 127.85, 126.40, 126.14 (q, $J_{C\text{-}F}$ = 3.8 Hz), 124.25, 49.12, 49.00, 42.60.*

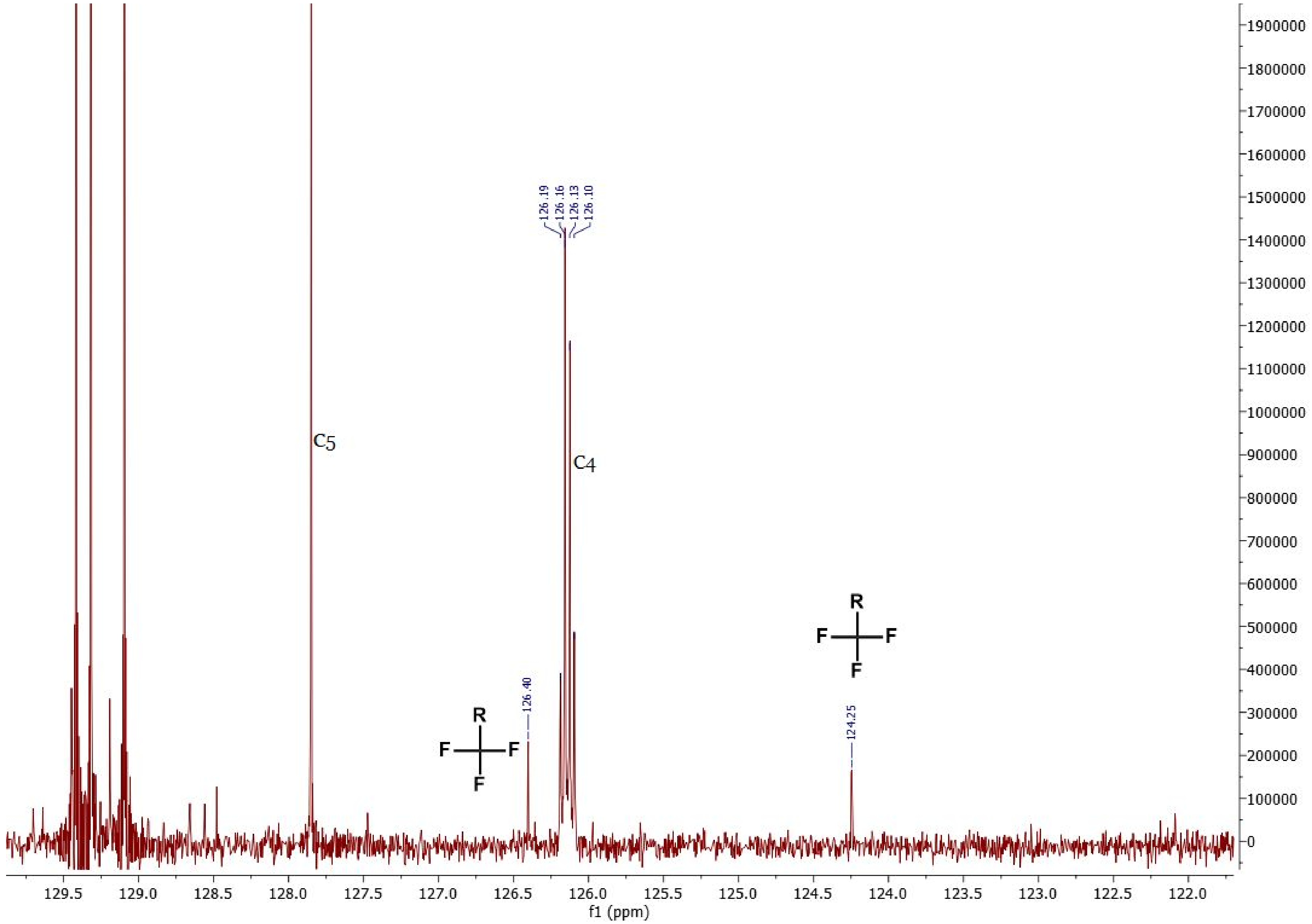

*Figure S113: $^{13}C\{^{1}H\}$ NMR spectrum of **11** from the CF_3 region.*

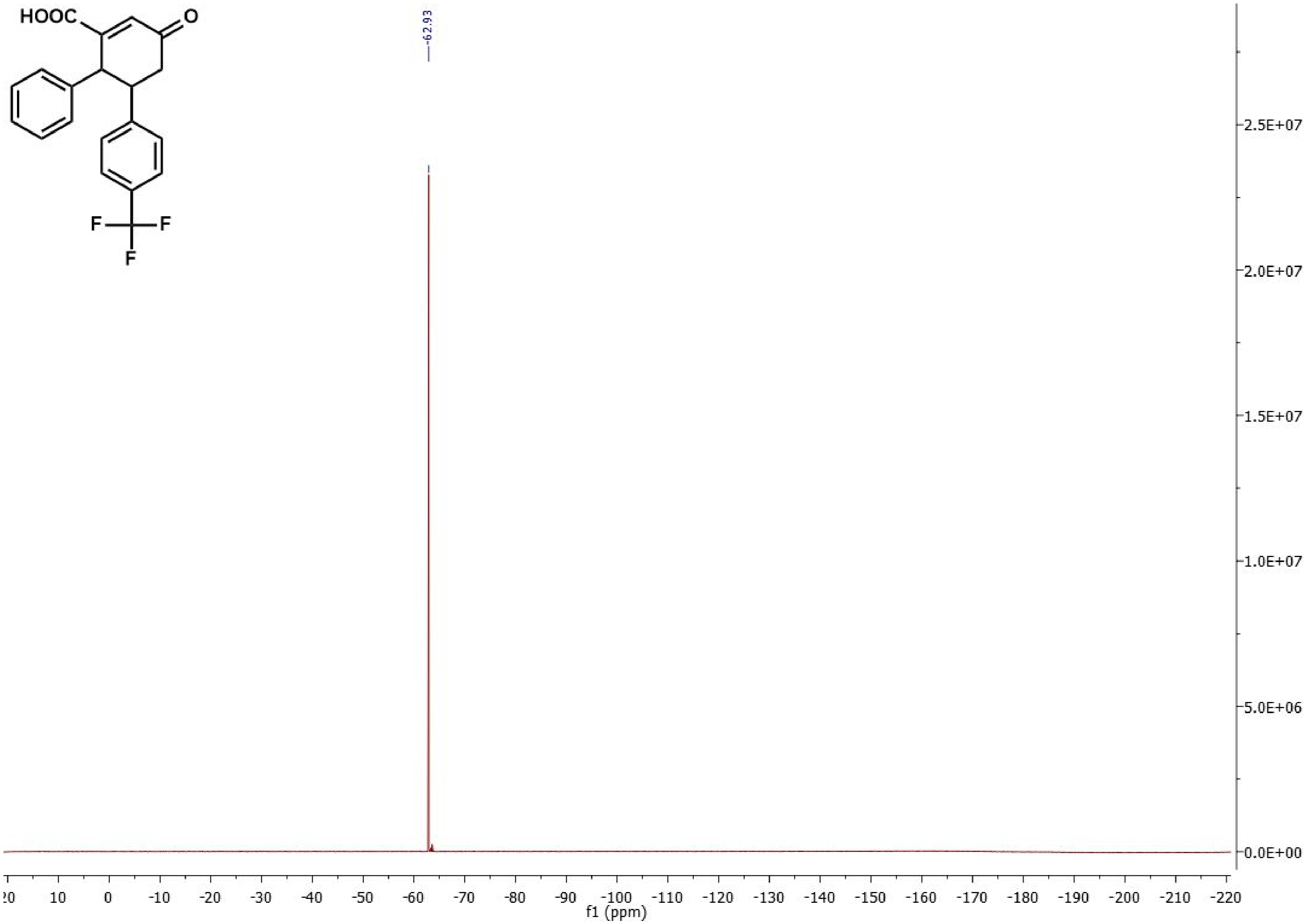

*Figure S114: ^{19}F NMR spectrum of **11** (470 MHz, acetone-d6): δ -62.93.*

2D NMR of **11**

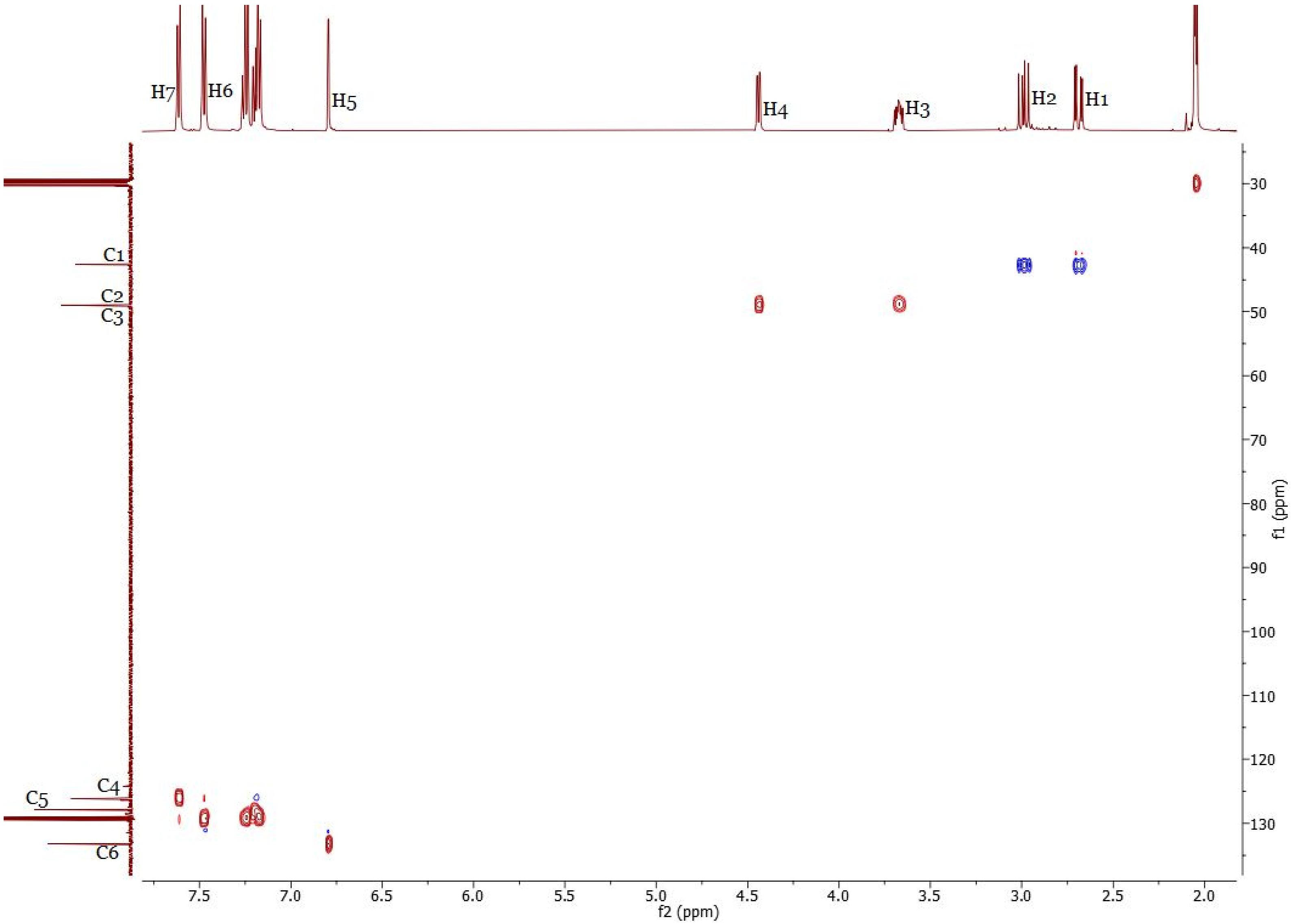

*Figure S115: HSQC spectrum of **11**.*

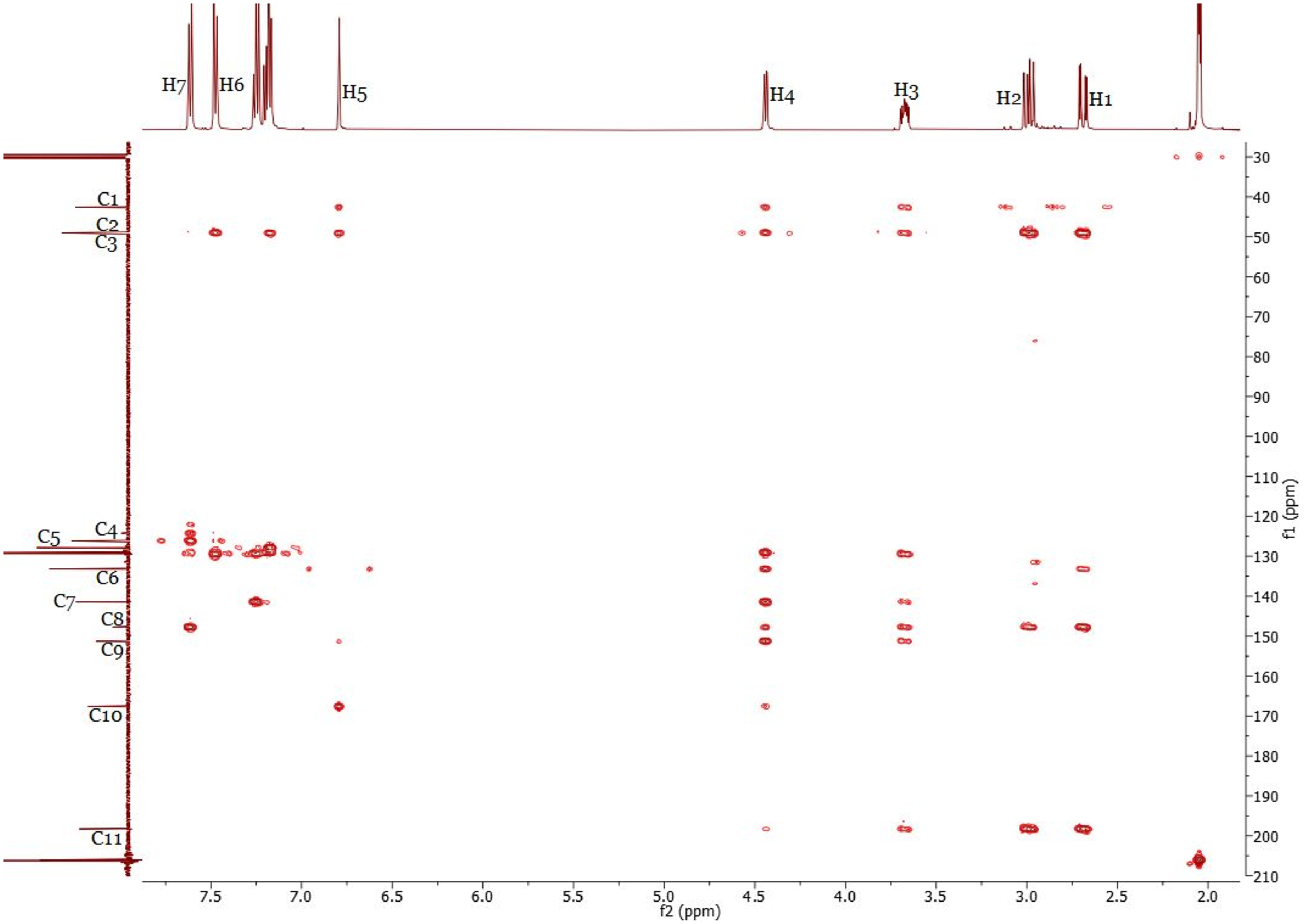

*Figure S116: HMBC spectrum of **11**.*

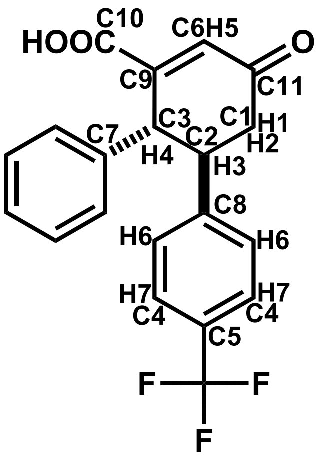

*Figure S117: 2D observations of **11**.*

2D NMR observations of **11**:

Protons H1 and H2 are attached to carbon C1 forming CH_2 group. The group has connectivity to carbons C2, C3, C6, C8 and C11.

Proton H3 is attached to carbon C2 forming CH group. The group has connectivity to carbons C1, C3, C7 (weak), C8, C9 and C11.

Proton H4 is attached to carbon C3 forming CH group. The group has connectivity to carbons C1, C2, C6, C7, C8, C9, C10 and C11 (weak).

Proton H5 is attached to carbon C6 forming CH group. The group has connectivity to carbons C1, C3, C9 and C10.

Protons H7 are attached to carbons C4 forming two CH groups. The groups have connectivity to carbons C8 and CF_3 group.

IR spectroscopy of **11**

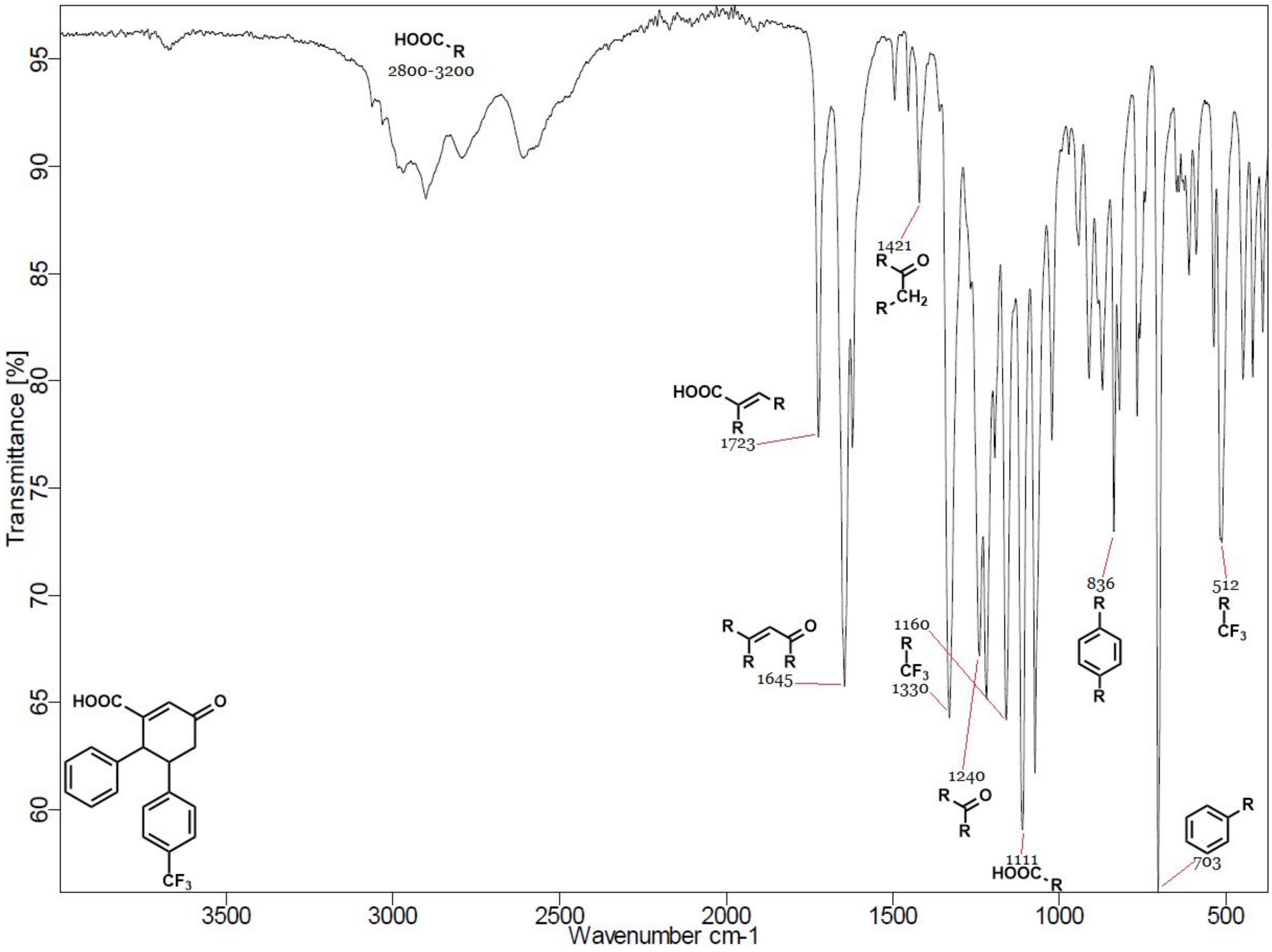

*Figure S118: IR spectrum of **11** (2800-3200 (broad), 1111 (s) (R-COOH), 1723 (s) (C=C-ROOH), 1645 (s) (C=C-**CO**-R), 1421 (m) (R-CO-**CH$_2$**-R), 1330 (s), 1160 (s), 512 (s) (R-CF$_3$), 1240 (s) (R-**CO**-R), 836 (s) (2 adjacent H (R-Ph-p-CF$_3$)), 703 (s) (5 adjacent H (Ph)) cm^{-1}).*

HRMS of **11**

HRMS (ESI-TOF) m/z: [**11**-H]- calculated for $C_{20}H_{14}O_3F_3$ 359.0890; Found 359.0893; Error 0.923 ppm.

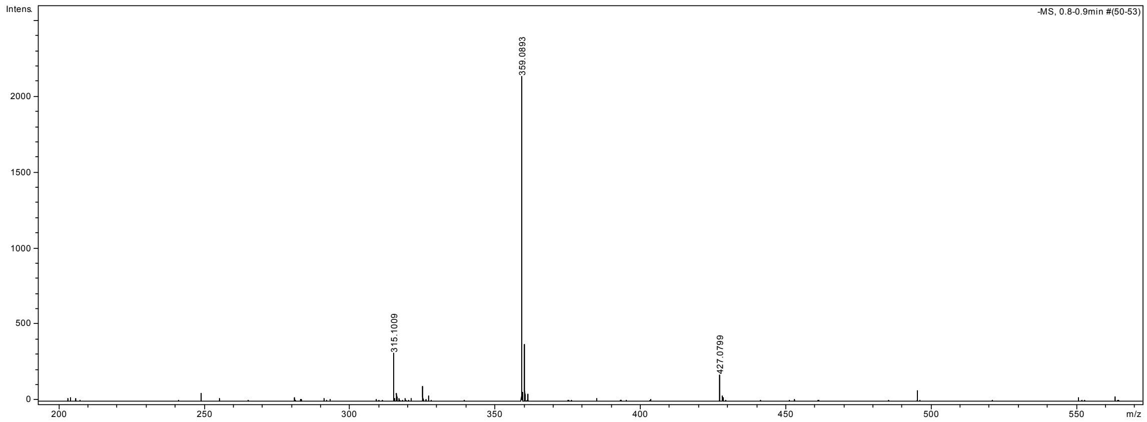

*Figure S119: ESI-TOF-MS of [**11**-H]- (peak: 359.0893 m/z, negative-ion mode).*

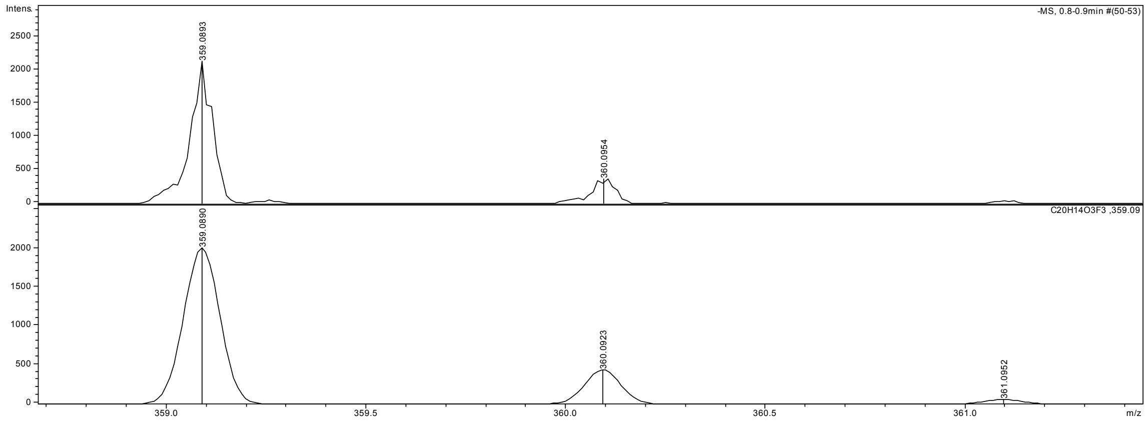

*Figure S120: Measured compound peak of [**11**-H]- (359.0893 m/z) at top, simulated peak ($C_{20}H_{14}O_3F_3$) below.*

3.14 Spectroscopic data of 12a

1D NMR of **12a**

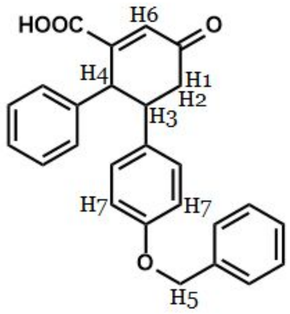

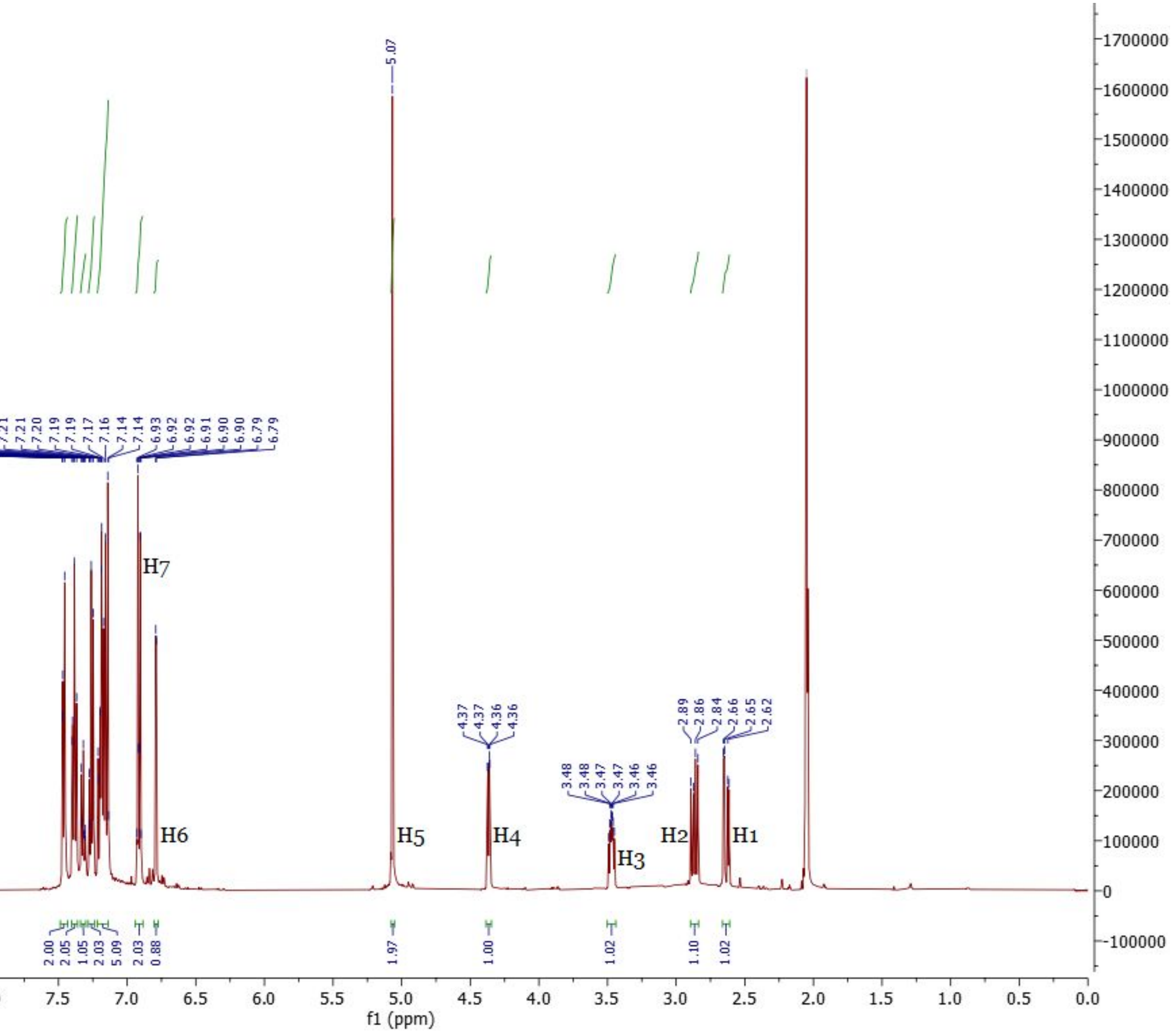

*Figure S121: ^{1}H NMR spectrum of **12a** (500 MHz, acetone-d6): δ 7.47 (m, 2H), 7.39 (m, 2H), 7.32 (m, 1H), 7.26 (m, 2H), 7.22-7.11 (m, 5H), 6.91 (m, 2H), 6.79 (d, J = 1.9 Hz, 1H), 5.07 (s, 2H), 4.37 (dd, J = 6.7, 2.0 Hz, 1H), 3.47 (ddd, J = 9.3, 6.7, 4.5 Hz, 1H), 2.87 (dd, J = 16.4, 9.2 Hz, 1H), 2.64 (dd, J = 16.4, 4.5 Hz, 1H).*

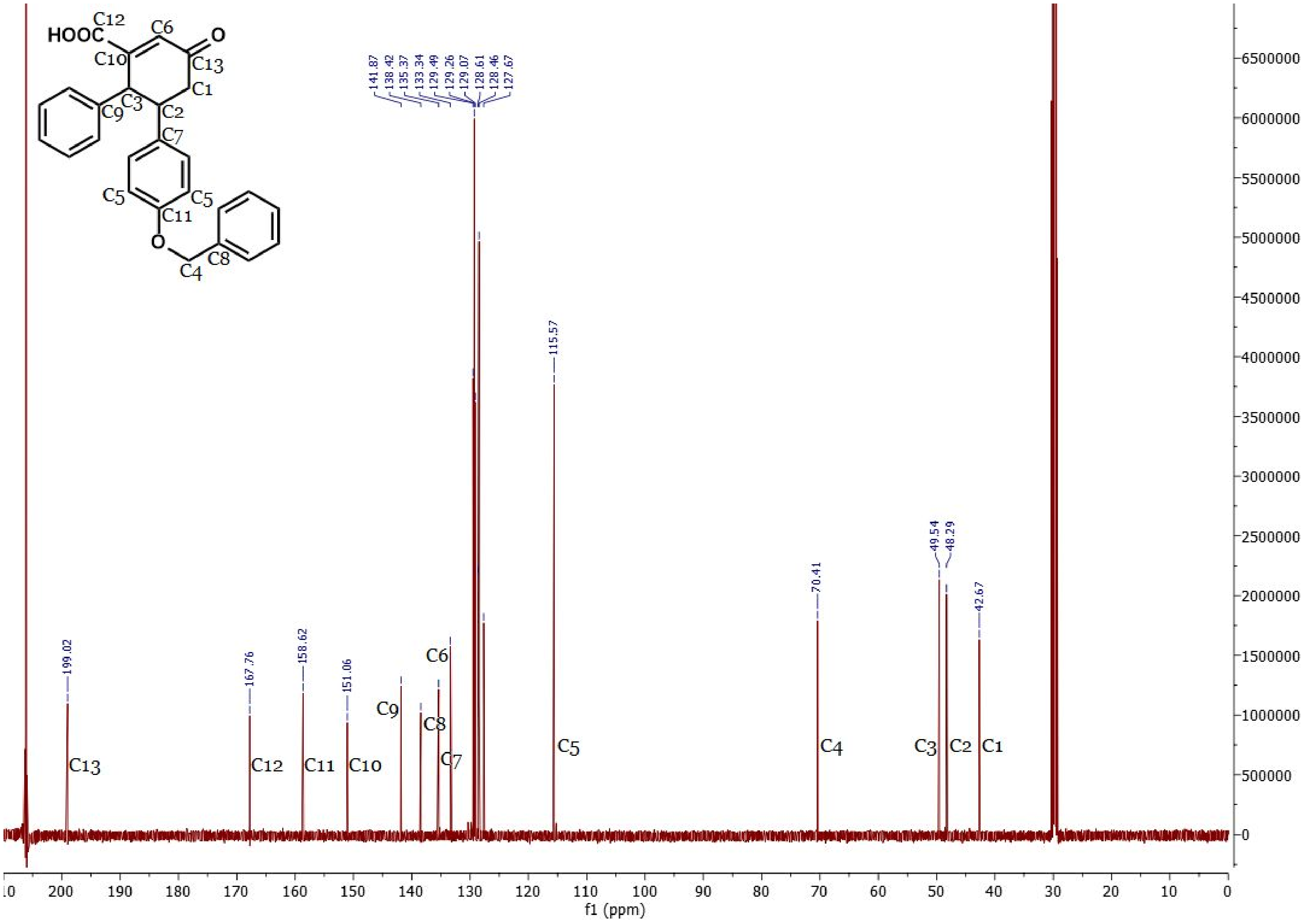

*Figure S122: $^{13}C\{^1H\}$ NMR spectrum of **12a** (125 MHz, acetone-d6): δ 199.02, 167.76, 158.62, 151.06, 141.87, 138.42, 135.37, 133.34, 129.49, 129.26, 129.07, 128.61, 128.46, 127.67, 115.57, 70.41, 49.54, 48.29, 42.67. Note: one carbon peak is missing, probably due overlapping at aromatic region.*

2D NMR of **12a**

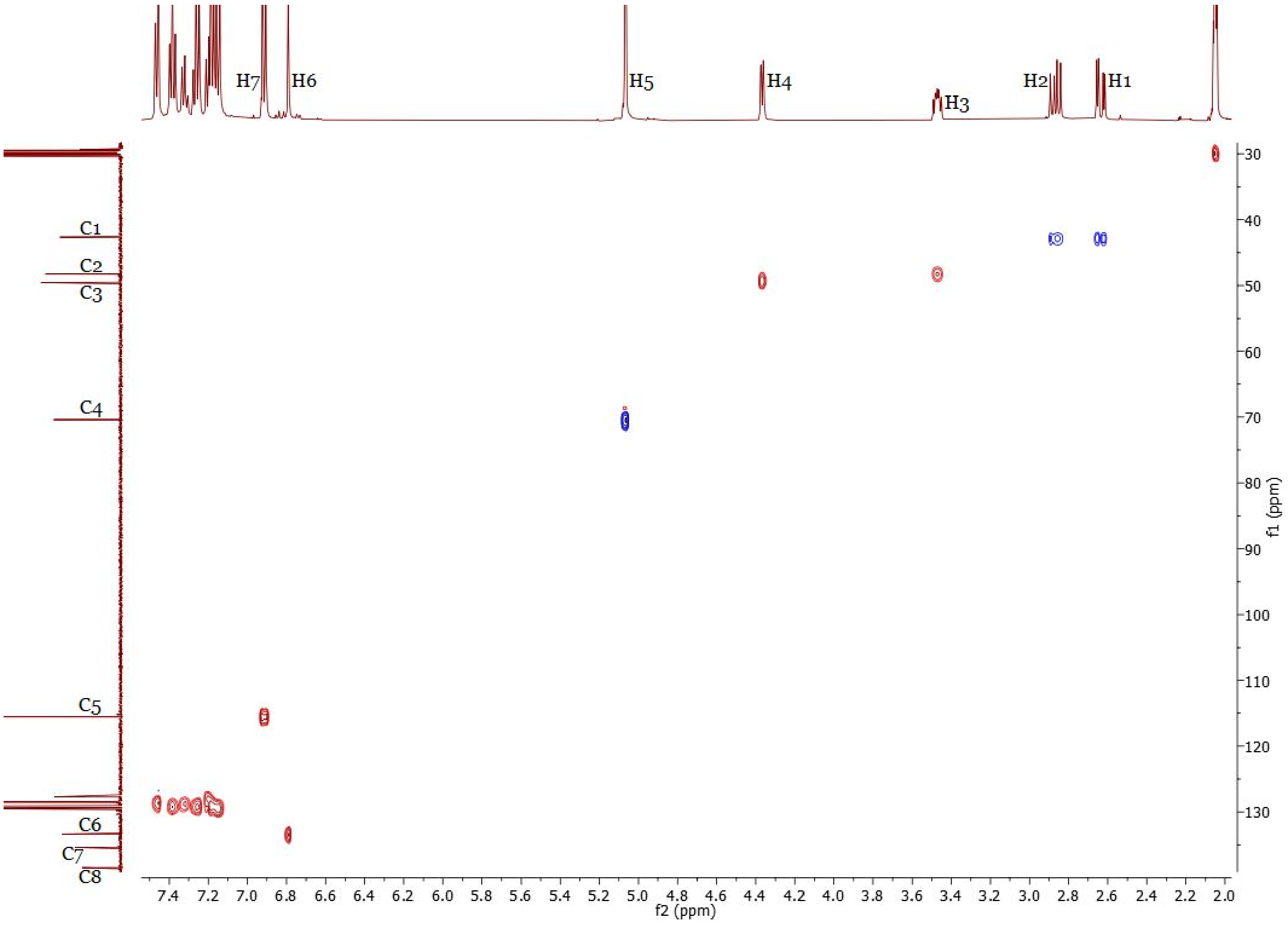

*Figure S123: HSQC spectrum of **12a**.*

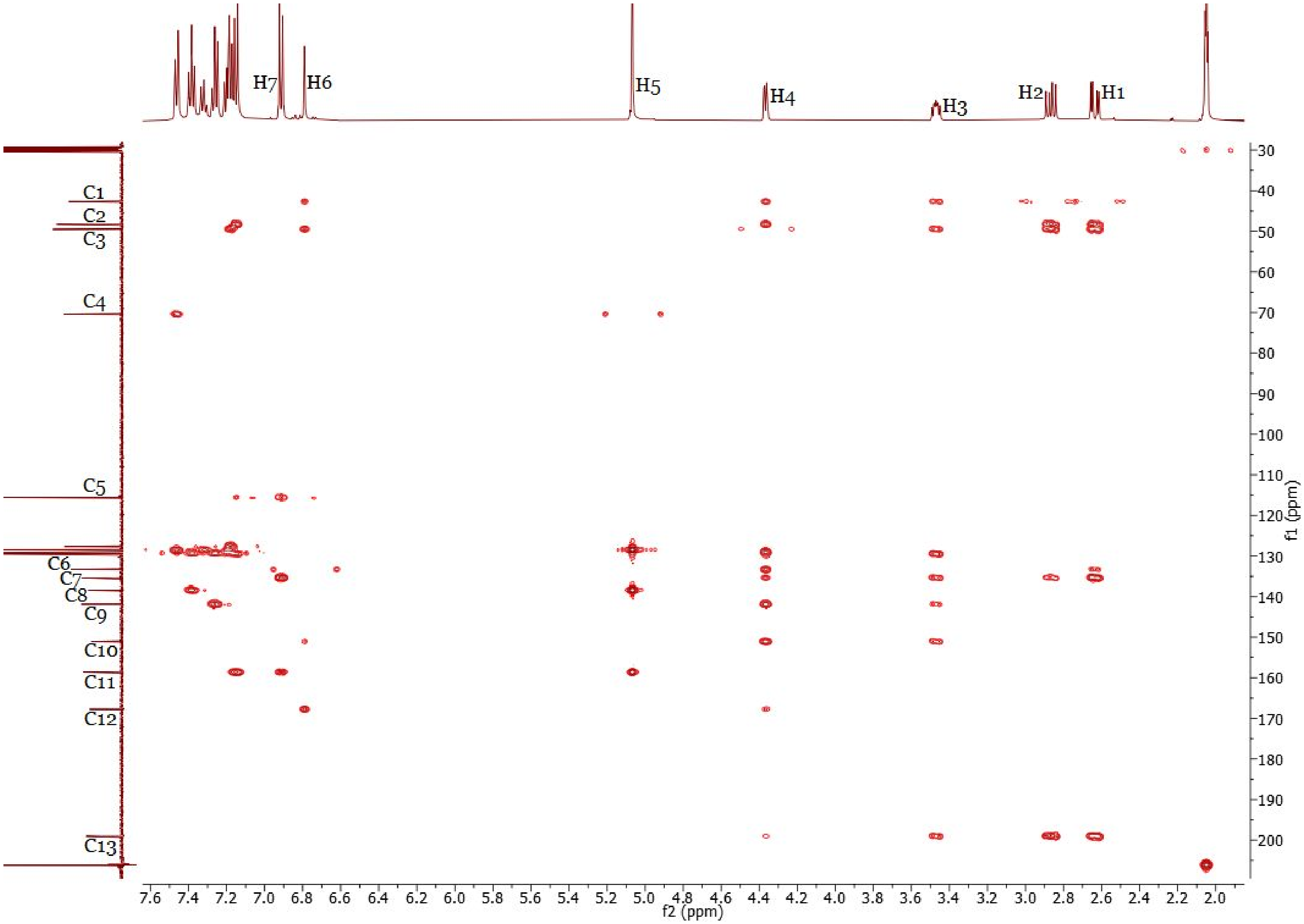

*Figure S124: HMBC spectrum of **12a**.*

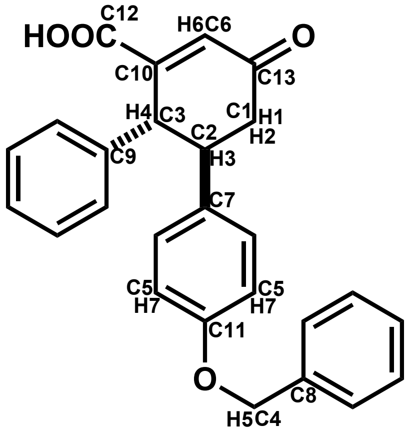

*Figure S125: 2D observations of **12a**.*

2D NMR observations of **12a**:

Protons H1 and H2 are attached to carbon C1 forming CH_2 group. The group has connectivity to carbons C2, C3, C6, C7 and C13.

Proton H3 is attached to carbon C2 forming CH group. The group has connectivity to carbons C1, C3, C7, C9, C10 and C13.

Proton H4 is attached to carbon C3 forming CH group. The group has connectivity to carbons C1, C2, C6, C7, C9, C10, C12 (weak) and C13 (weak).

Protons H5 are attached to carbon C4 forming CH_2 group. The group has connectivity to carbons C8 and C11.

Proton H6 is attached to carbon C6 forming CH group. The group has connectivity to carbons C1, C3, C10 and C12.

Protons H7 are attached to carbons C5 forming two CH groups. The groups have connectivity to carbons C5 (itself), C7 and C11.

IR spectroscopy of **12a**

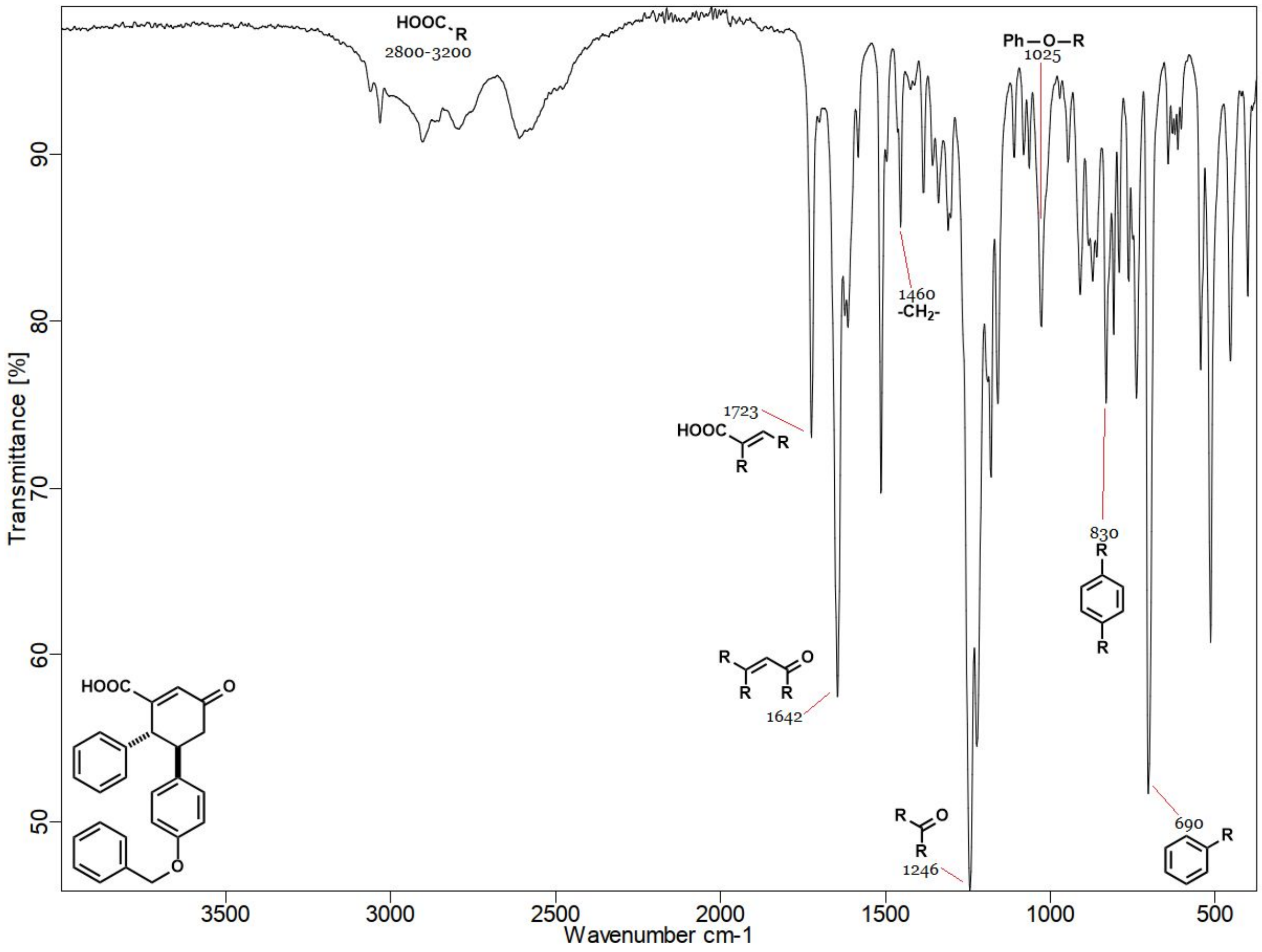

*Figure S126: IR spectrum of **12a** (2800-3200 (broad), (R-COOH), 1723 (s) (C=C-ROOH), 1460 (m) (R-CH$_2$-R), 1246 (s) (R-**CO**-R), 1025 (m) (Ph-O-R), 830 (m) (2 adjacent H (R-Ph-R)), 690 (s) (5 adjacent H (Ph)) cm^{-1}).*

HRMS of **12a**

HRMS (ESI-TOF) m/z: [**12a**-H]$^-$ calculated for $C_{26}H_{21}O_4$ 397.1434; Found 397.1430; Error 0.992 ppm.

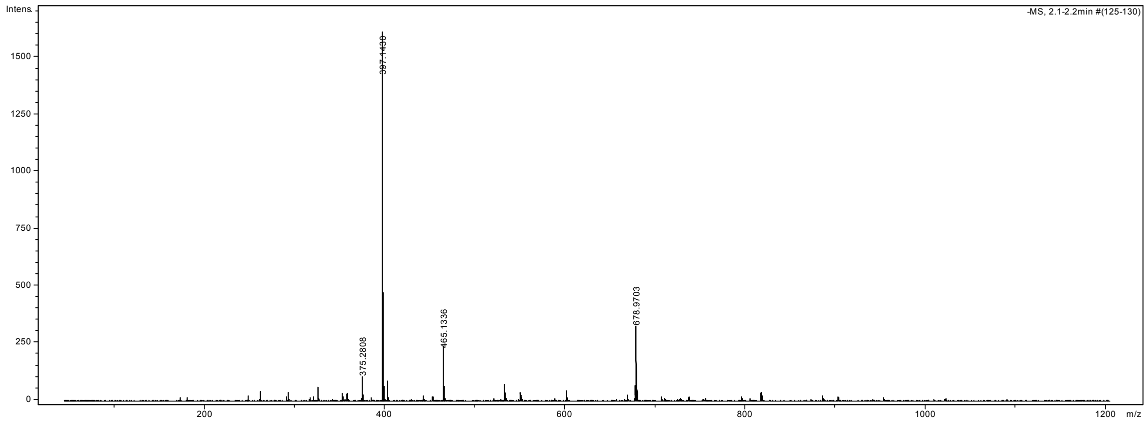

*Figure S127: ESI-TOF-MS of [**12a**-H]$^-$ (peak: 397.1430 m/z, negative-ion mode).*

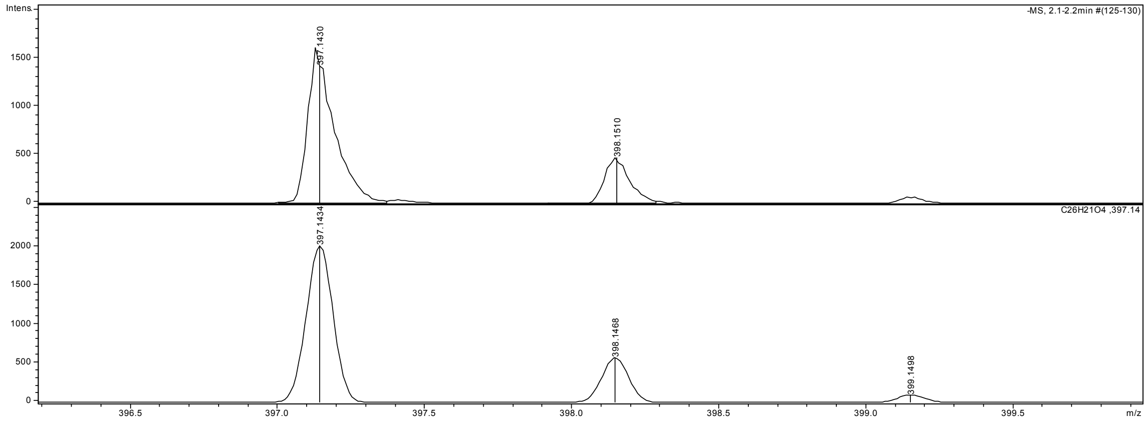

*Figure S128: Measured compound peak of [**12a**-H]$^-$ (397.1430 m/z) at top, simulated peak ($C_{26}H_{21}O_4$) below.*

3.15 Spectroscopic data of 12b

1D NMR of **12b**

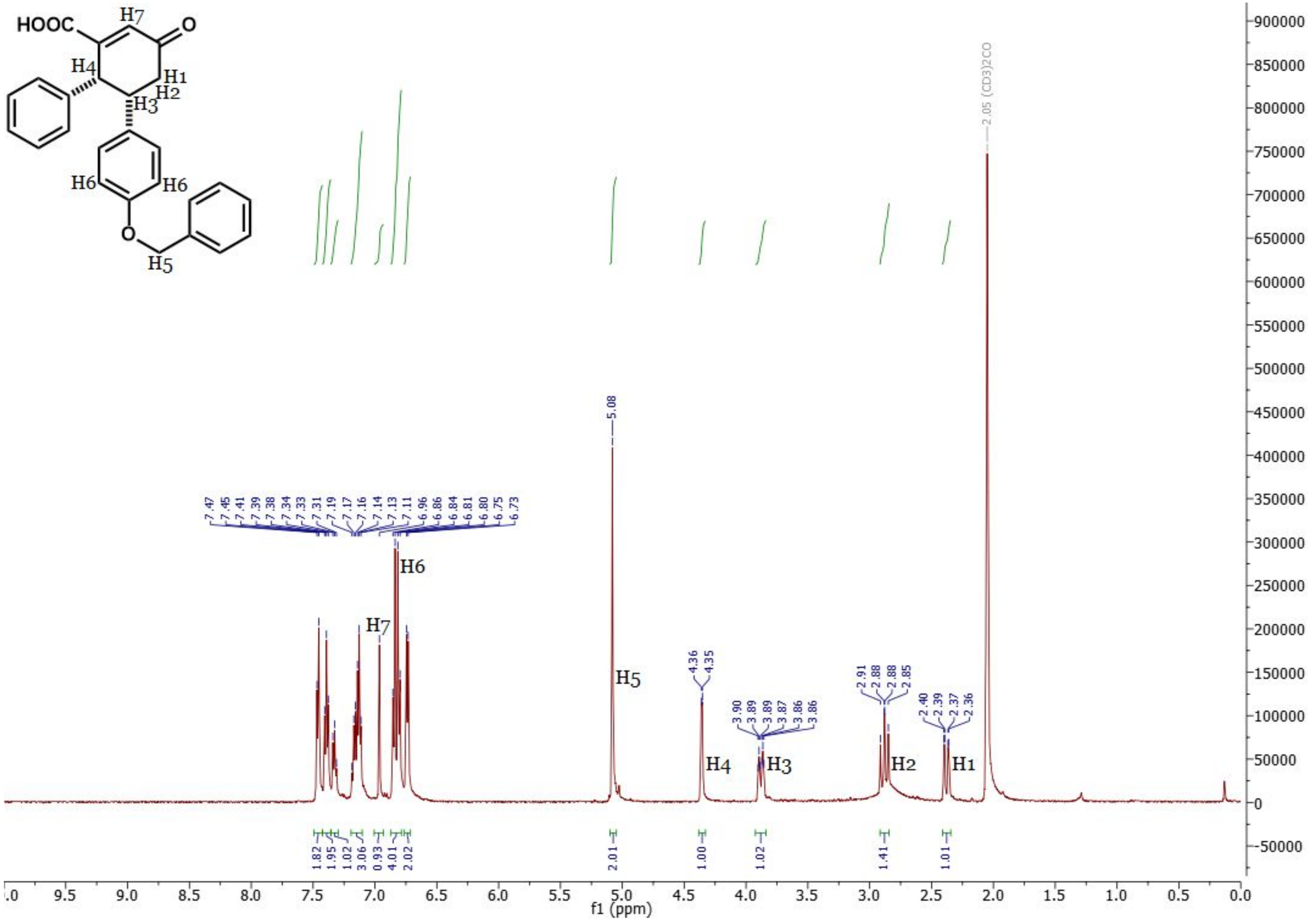

*Figure S129: ¹H NMR spectrum of **12b** (500 MHz, acetone-d6): δ 7.49-7.43 (m, 2H), 7.42-7.36 (m, 2H), 7.35-7.29 (m, 1H), 7.19-7.09 (m, 3H), 6.96 (s, 1H), 6.87-6.78 (m, 4H), 6.82 (d, J = 6.8 Hz, 2H), 5.08 (s, 2H), 4.36 (d, J = 4.9 Hz, 1H), 3.88 (dt, J = 15.2, 4.1 Hz, 1H), 2.88 (dd, J = 16.9, 15.1 Hz, 1H), 2.38 (dd, J = 16.8, 3.6 Hz, 1H).*

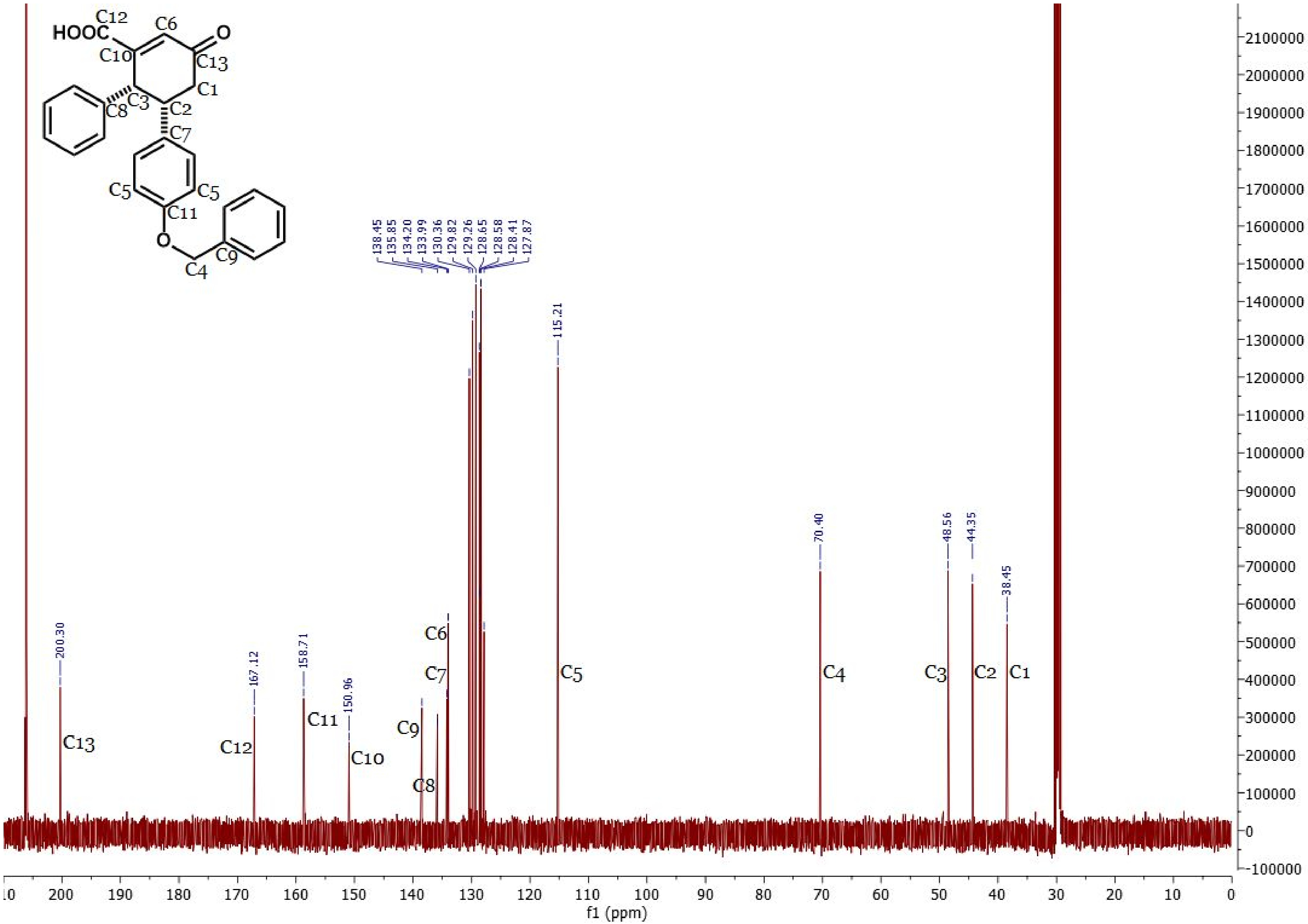

*Figure S130: $^{13}C\{^{1}H\}$ NMR spectrum of **12b** (125 MHz, acetone-d6): δ 200.30, 167.12, 158.71, 150.96, 138.45, 135.85, 134.20, 133.99, 130.36, 129.82, 129.26, 128.65, 128.58, 128.41, 127.87, 115.21, 70.40, 48.56, 44.35, 38.45.*

2D NMR of **12b**

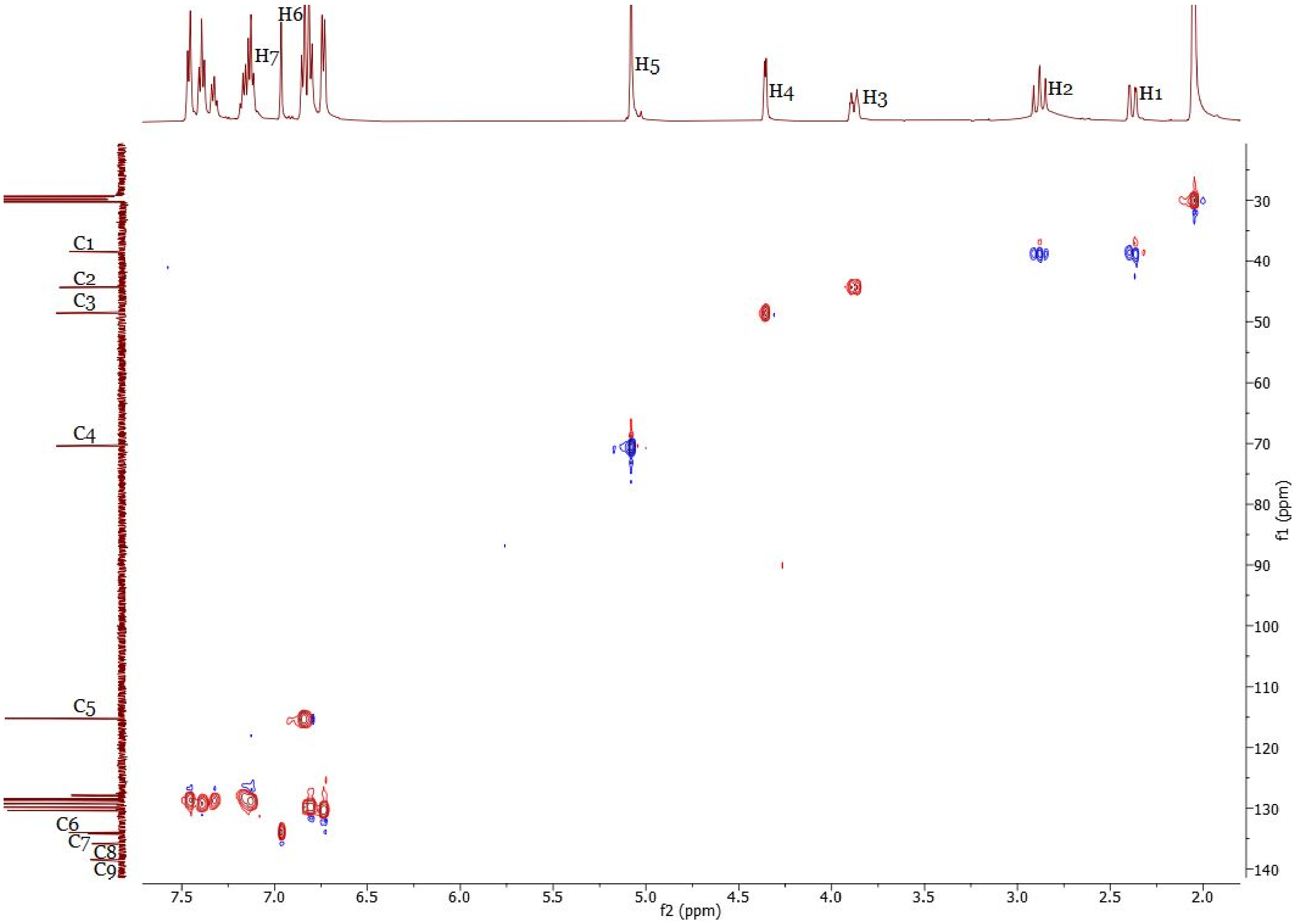

*Figure S131: HSQC spectrum of **12b**.*

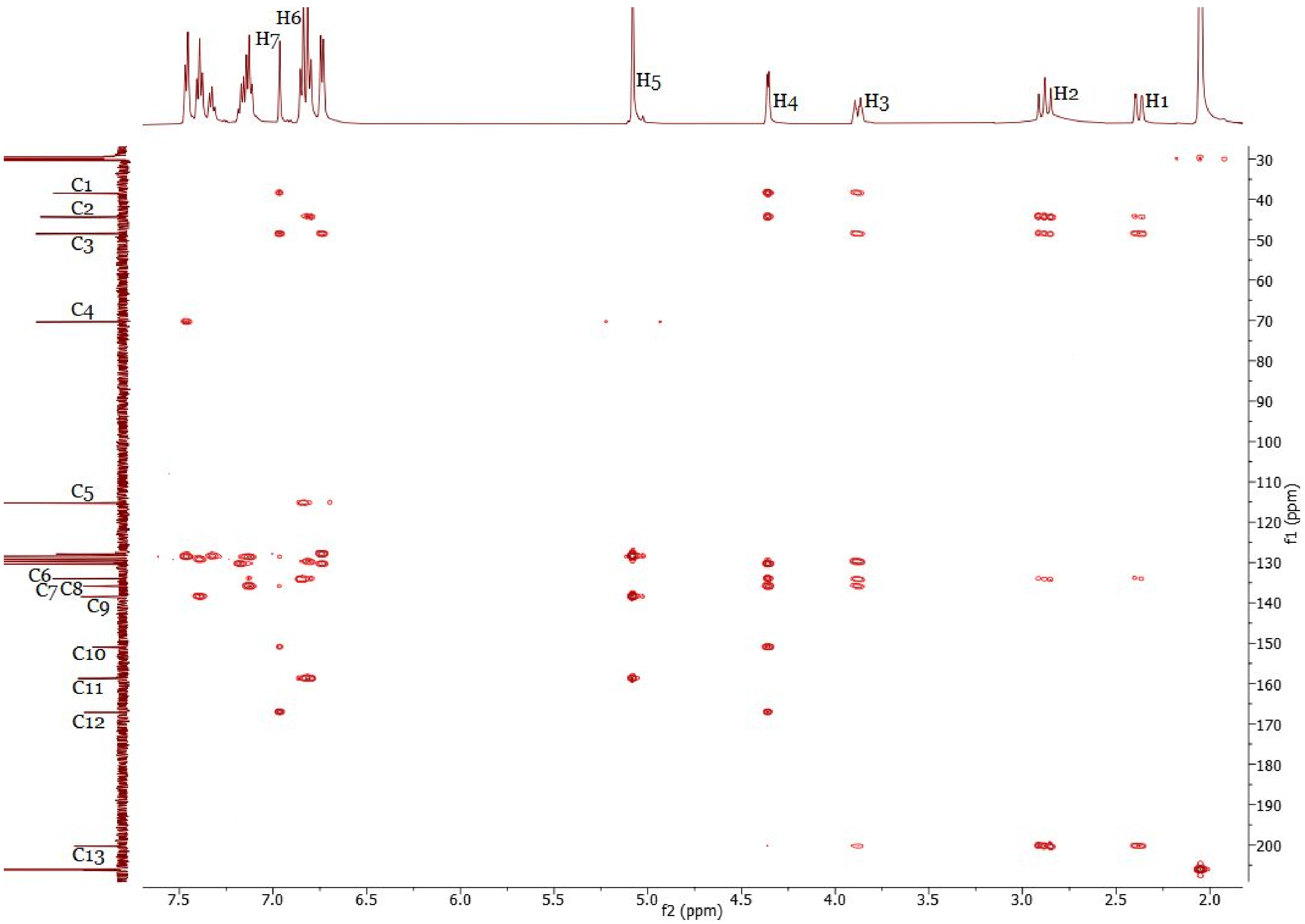

*Figure S132: HMBC spectrum of **12b**.*

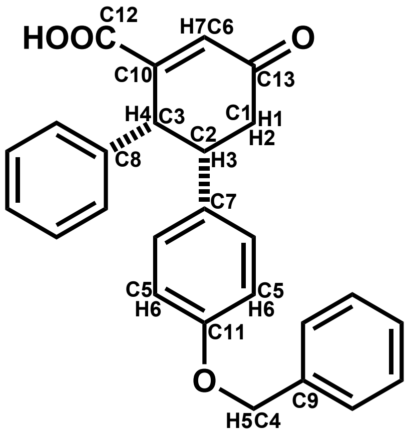

*Figure S133: 2D observations of **12b**.*

2D NMR observations of **12b**:

Protons H1 and H2 are attached to carbon C1 forming CH_2 group. The group has connectivity to carbons C2, C3, C6 (weak), C7 (weak) and C13.

Proton H3 is attached to carbon C2 forming CH group. The group has connectivity to carbons C1, C3, C7, C8, C10 (very weak) and C13 (weak). The group has connection inside a phenyl group, suggesting nearby location.

Proton H4 is attached to carbon C3 forming CH group. The group has connectivity to carbons C1, C2, C6, C8, C10 and C12. The group has connection inside a phenyl group, suggesting nearby location.

Protons H5 are connected to carbon C4 forming CH_2 group. The group has connectivity to carbons C9 and C11. The group has connection inside a phenyl group, suggesting nearby location.

Protons H6 are attached to carbons C5 forming two aromatic CH groups. The groups have connection to carbons C5 (itself), C7 and C11.

Proton H7 is attached to carbon C6 forming CH group. The group has connectivity to carbons C1, C3, C8 (very weak), C10 and C12.

IR spectroscopy of **12b**

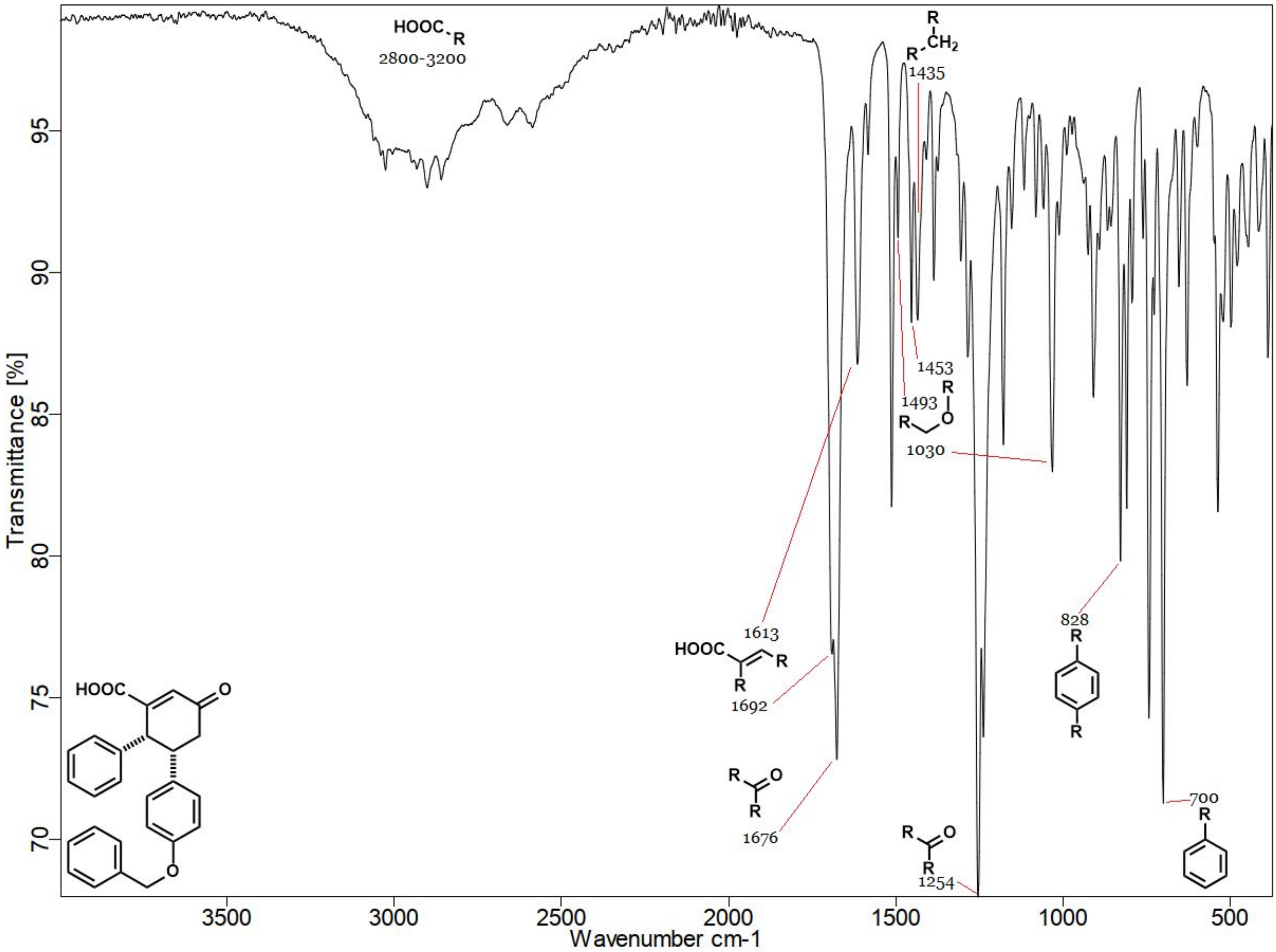

*Figure S134: IR spectrum of **12b** (2800-3200 (broad) (R-COOH), 1692 (s), 1613 (m) (C=C-ROOH), 1676 (s), 1254 (s) (R-**CO**-R), 1493 (w), 1453 (m), 1030 (m) (R-O-$\mathbf{CH_2}$-R), 1435 (m) (R-$\mathbf{CH_2}$-R), 828 (m) (2 adjacent H (R-Ph-R)), 700 (s) (5 adjacent H (Ph)) cm^{-1}).*

HRMS of **12b**

HRMS (ESI-TOF): m/z: [**12b**-H]$^-$ calculated for $C_{26}H_{21}O_4$ 397.1434; Found 397.1435; Error 0.186 ppm.

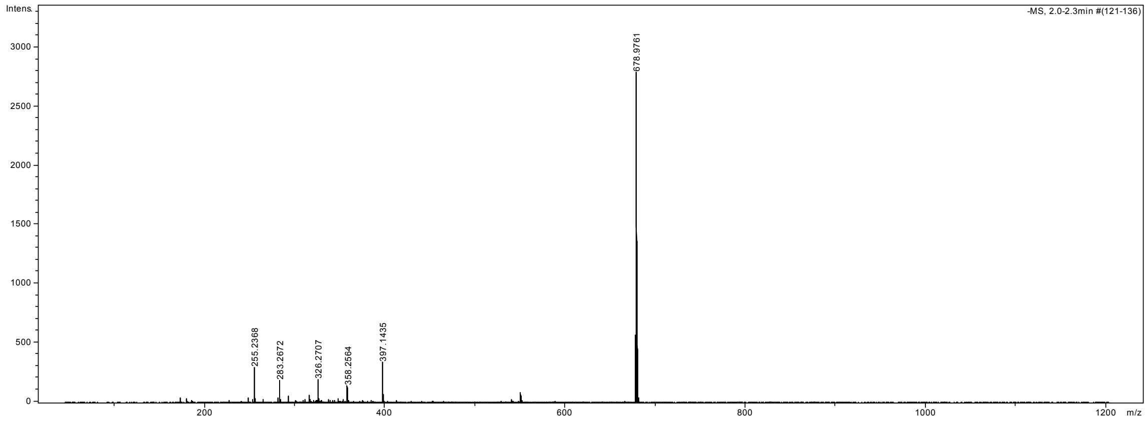

*Figure S135: ESI-TOF-MS of [**12b**-H]$^-$ (peak: 397.1435 m/z, negative-ion mode).*

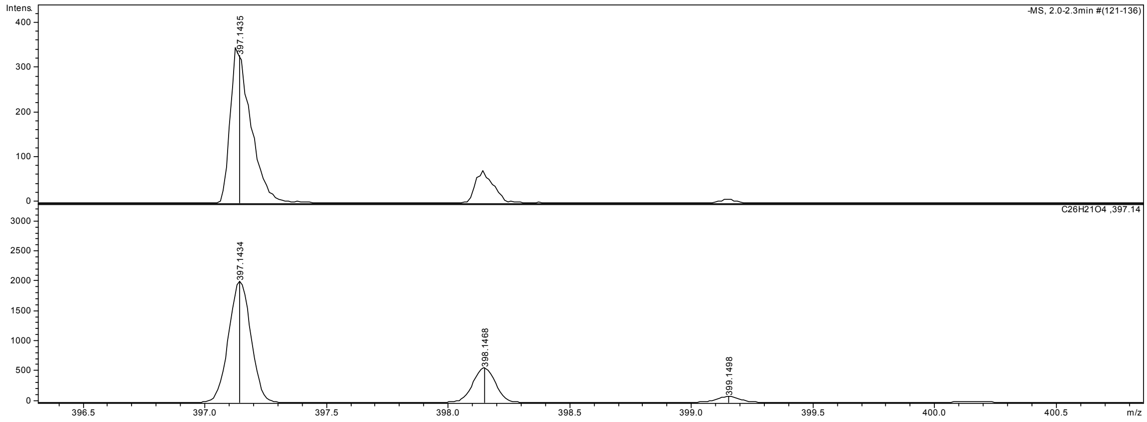

*Figure S136: Measured compound peak of [**12b**-H]$^-$ (397.1435 m/z) at top, simulated peak ($C_{26}H_{21}O_4$) below.*

3.16 Spectroscopic data of 13a

1D NMR of **13a**

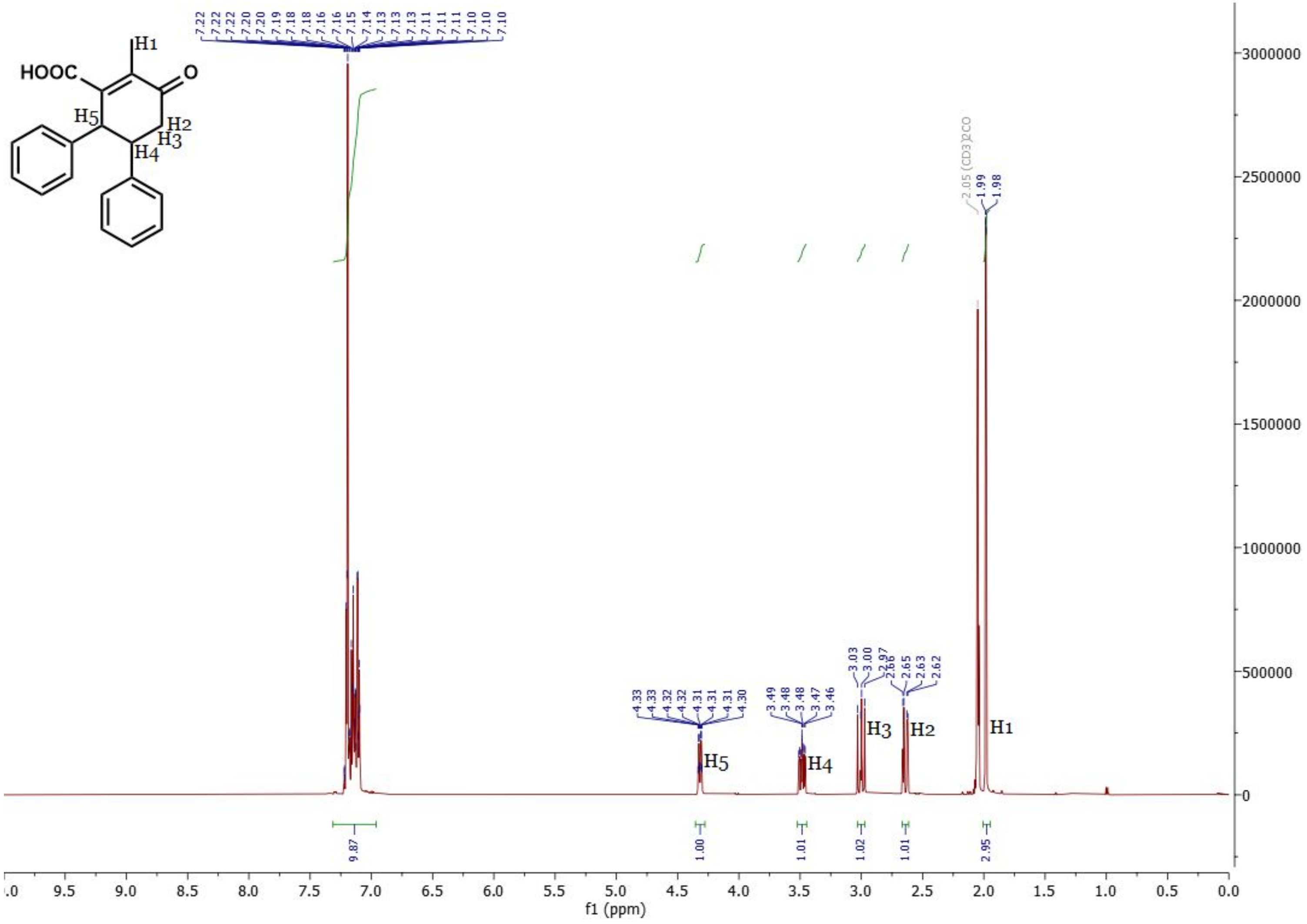

*Figure S137: ^{1}H NMR spectrum of **13a** (500 MHz, acetone-d6): δ 7.22-7.09 (m, 10H), 4.32 (dq, J = 9.3, 2.3 Hz, 1H), 3.48 (ddd, J = 12.4, 9.3, 4.0 Hz, 1H), 3.00 (dd, J = 15.9, 12.5 Hz, 1H), 2.64 (dd, J = 15.9, 4.0 Hz, 1H), 1.98 (d, J = 2.3 Hz, 3H).*

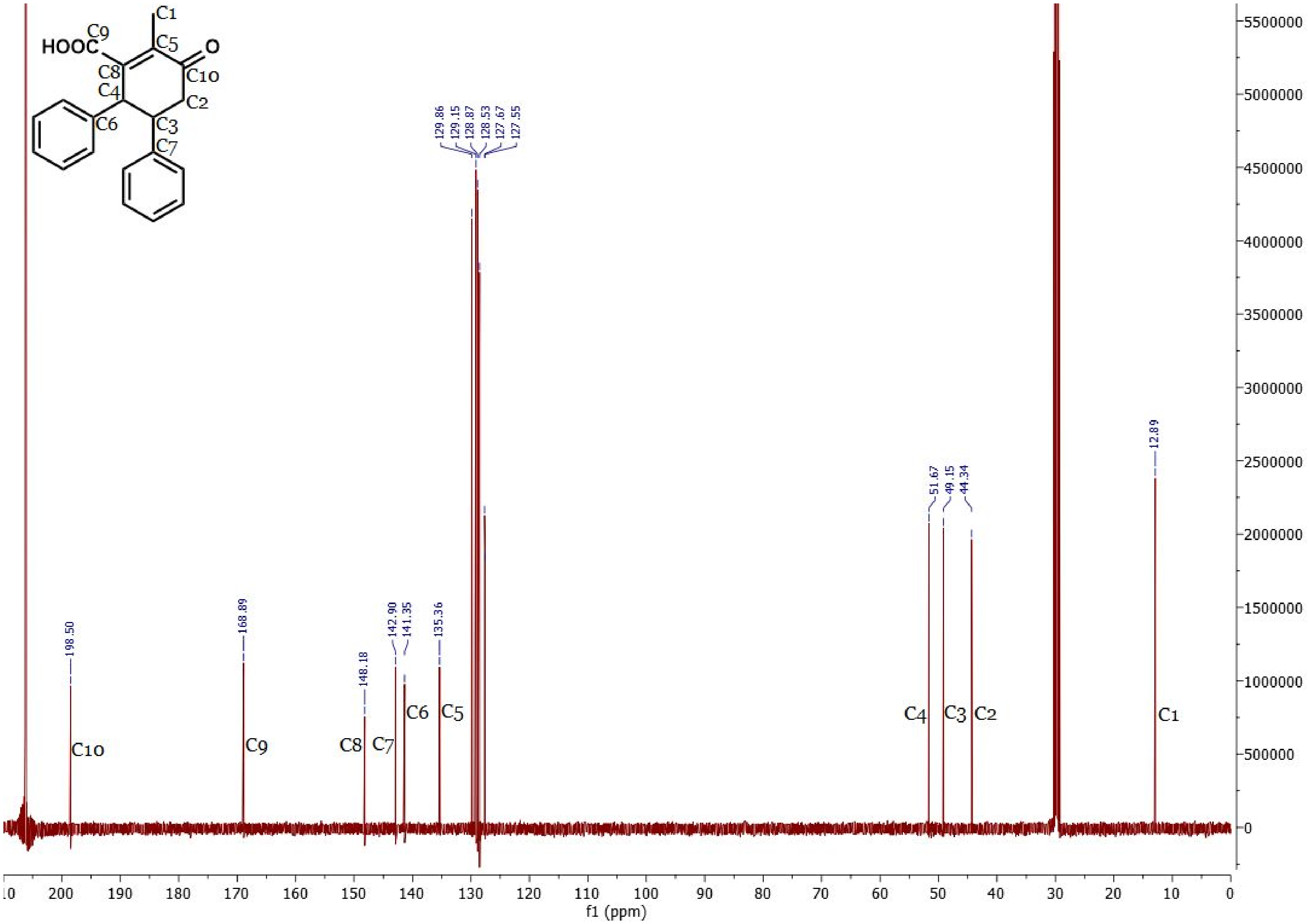

*Figure S138: $^{13}C\{^1H\}$ NMR spectrum of **13a** (125 MHz, acetone-d6): δ 198.50, 168.89, 148.18, 142.90, 141.35, 135.36, 129.86, 129.15, 128.87, 128.53, 127.67, 127.55, 51.67, 49.15, 44.34, 12.89.*

2D NMR of **13a**

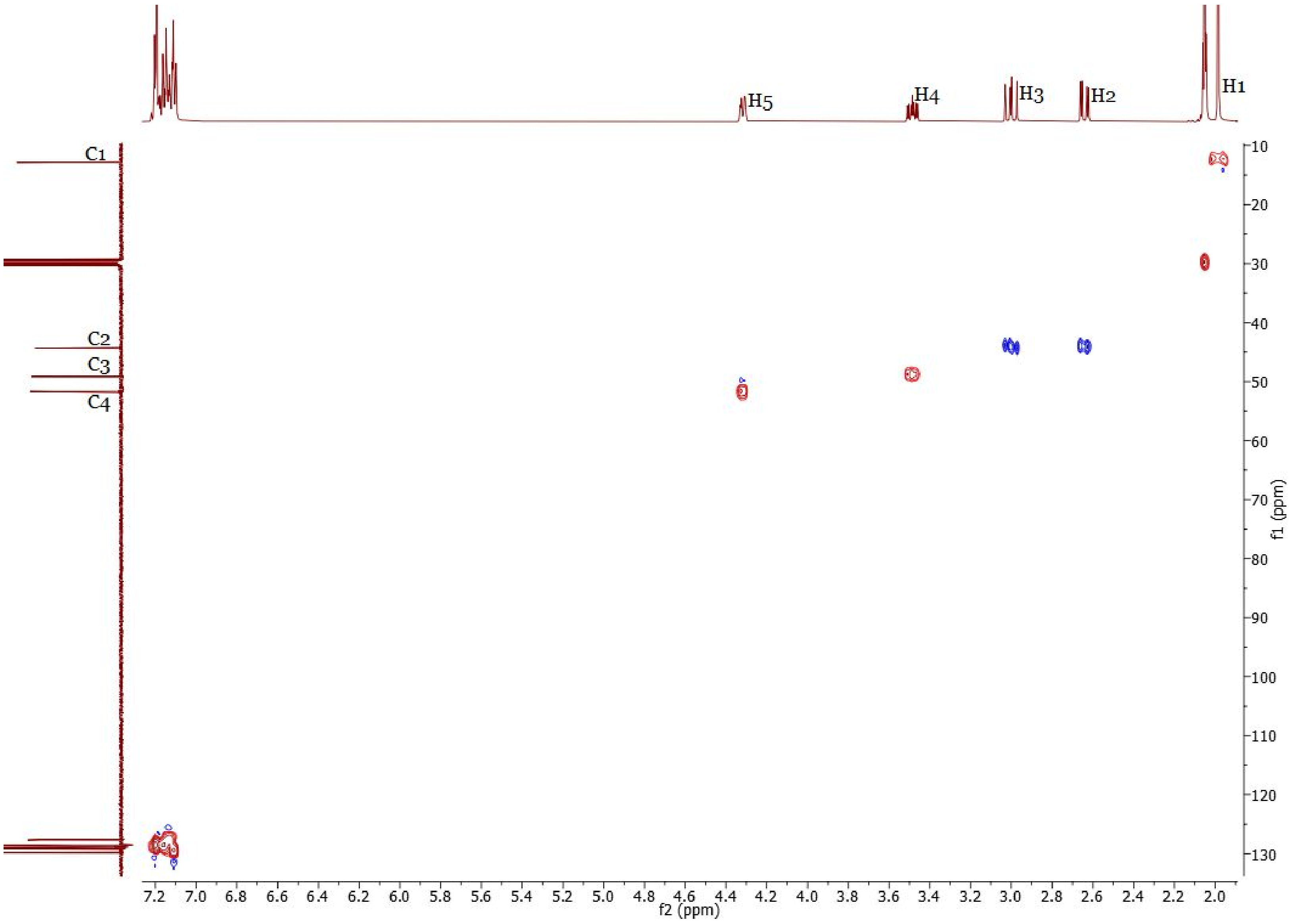

*Figure S139: HSQC spectrum of **13a**.*

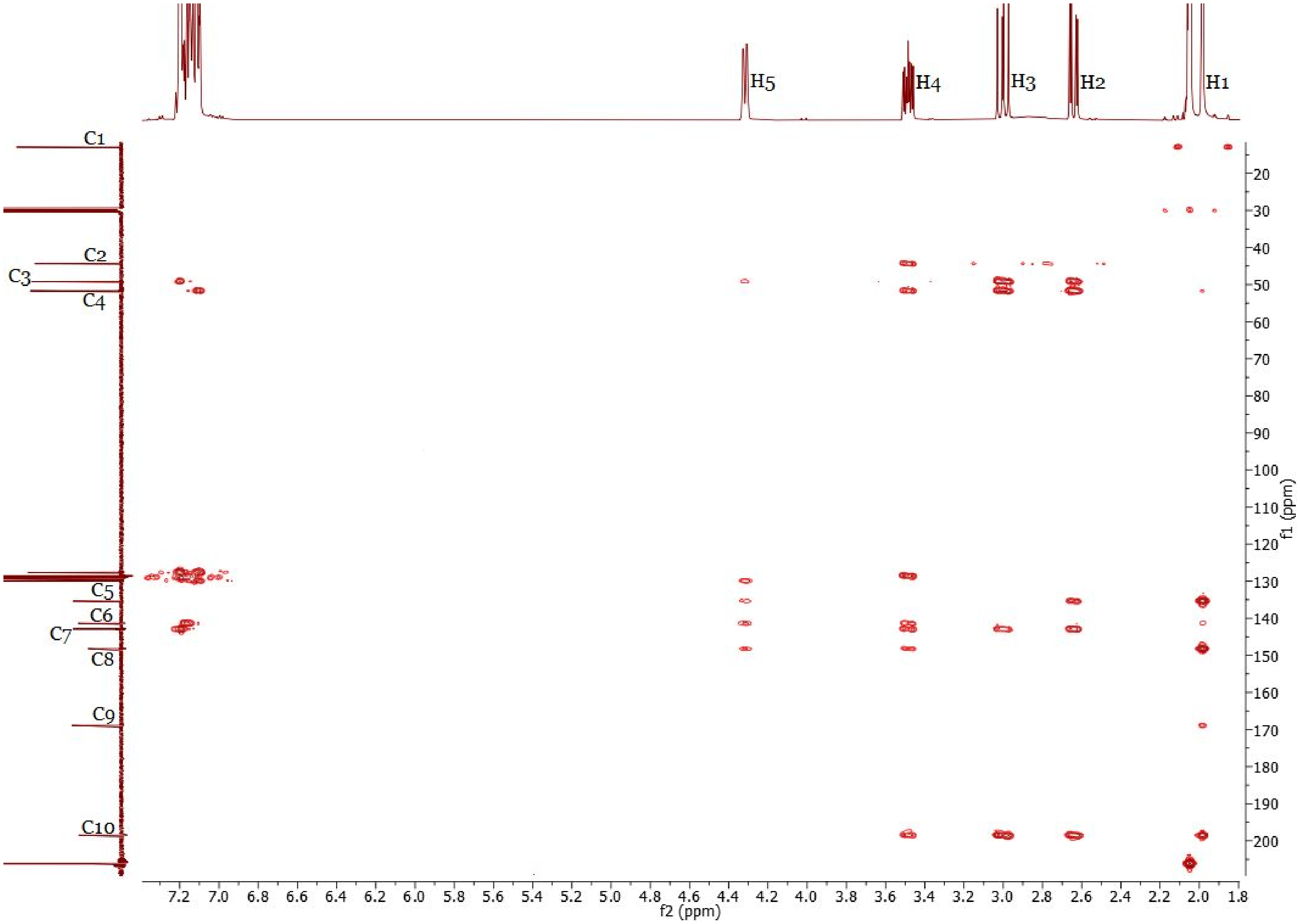

*Figure S140: HMBC spectrum of **13a**.*

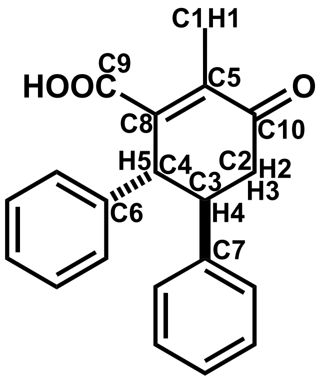

*Figure S141: 2D observations of **13a**.*

2D NMR observations of **13a**:

Protons H1 are attached to carbon C1 forming CH_3 group. The group has connectivity to carbons C5, C8, C9 (weak) and C10.

Protons H2 and H3 are attached to carbon C2 forming CH_2 group. The group has connectivity to carbons C3, C4, C5 (weak), C7 and C10.

Proton H4 is attached to carbon C3 forming CH group. The group has connectivity to carbons C2, C4, C6, C7, C8 and C10. The group has connectivity to inside aromatic group, suggesting nearby location.

Proton H5 is attached to carbon C4 forming CH group. The group has connectivity to carbons C3, C5 (weak), C6 and C8. The group has connectivity to inside aromatic group, suggesting nearby location.

IR spectroscopy of **13a**

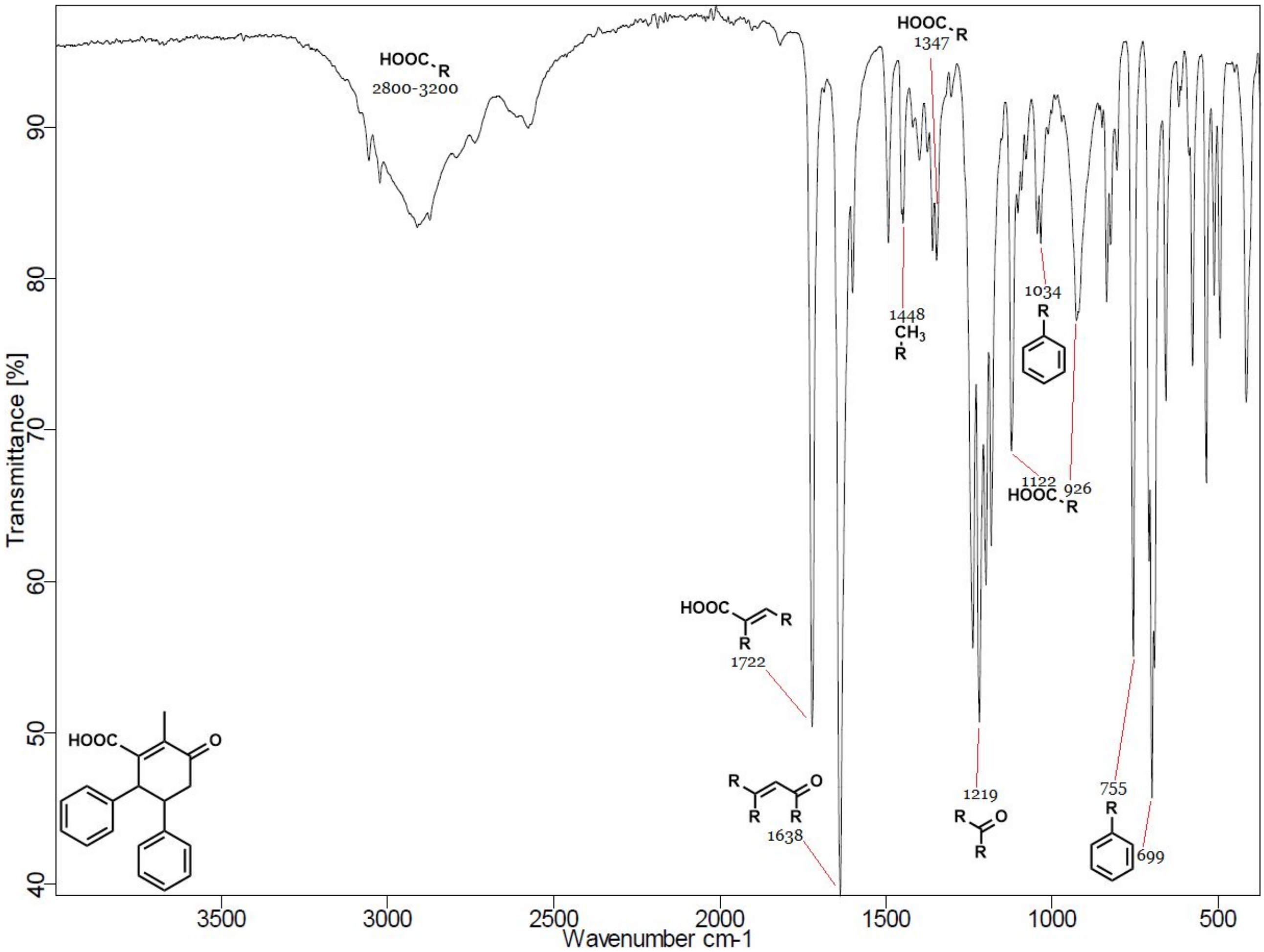

*Figure S142: IR spectrum of **13a** (2800-3200 (broad), 1347 (m), 1122 (m), 926 (m) (R-COOH), 1722 (s) (C=C-ROOH), 1638 (s) (C=C-**CO**-R), 1448 (m) (R-CH_3), 1219 (s) (R-**CO**-R), 1034(w), 755 (s), 699 (s) (5 adjacent H (Ph)) cm^{-1}).*

HRMS of **13a**

HRMS (ESI-TOF) m/z: [**13a**-H]$^-$ calculated for $C_{20}H_{17}O_3$ 305.1172; Found 305.1170; Error 0.789 ppm.

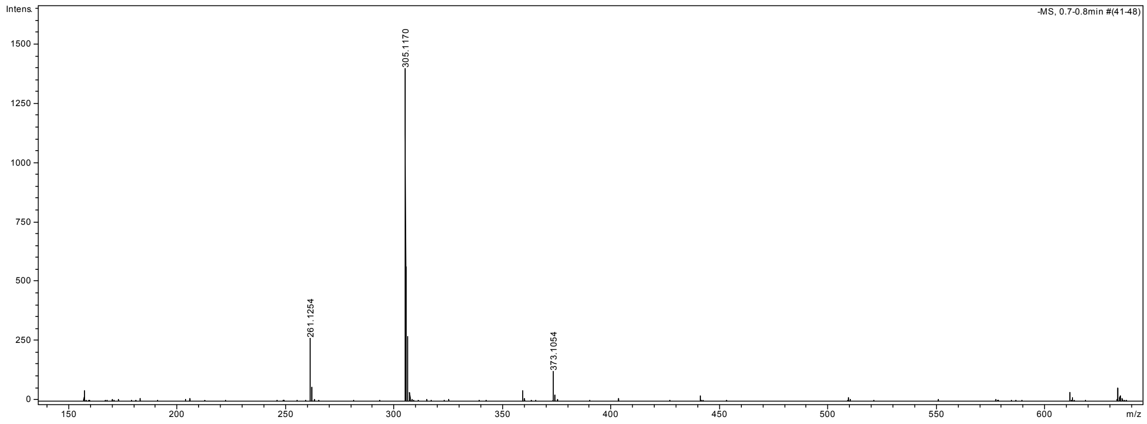

*Figure S143: ESI-TOF-MS of [**13a**-H]$^-$ (peak: 305.1170 m/z, negative-ion mode).*

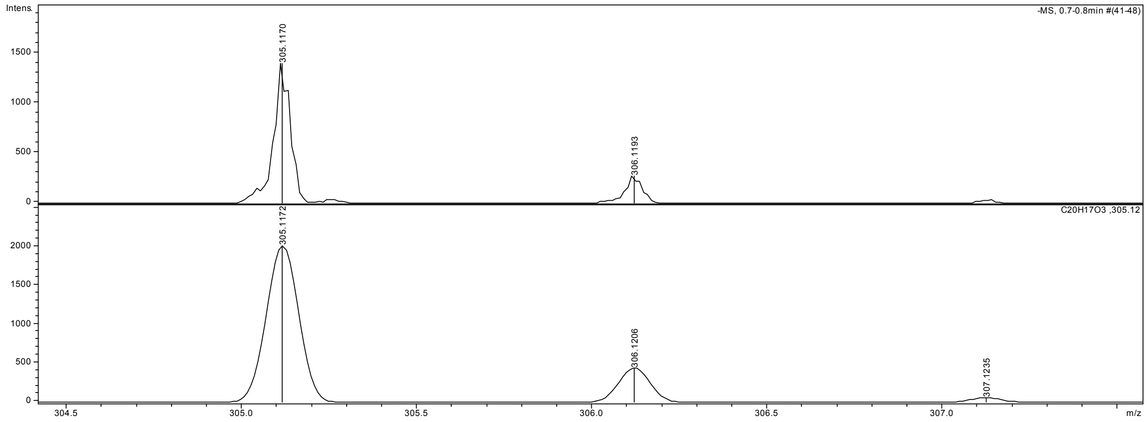

*Figure S144: Measured compound peak of [**13a**-H]$^-$ (305.1170 m/z) at top, simulated peak ($C_{20}H_{17}O_3$) below.*

3.17 Spectroscopic data of 13b

1D NMR of **13b**

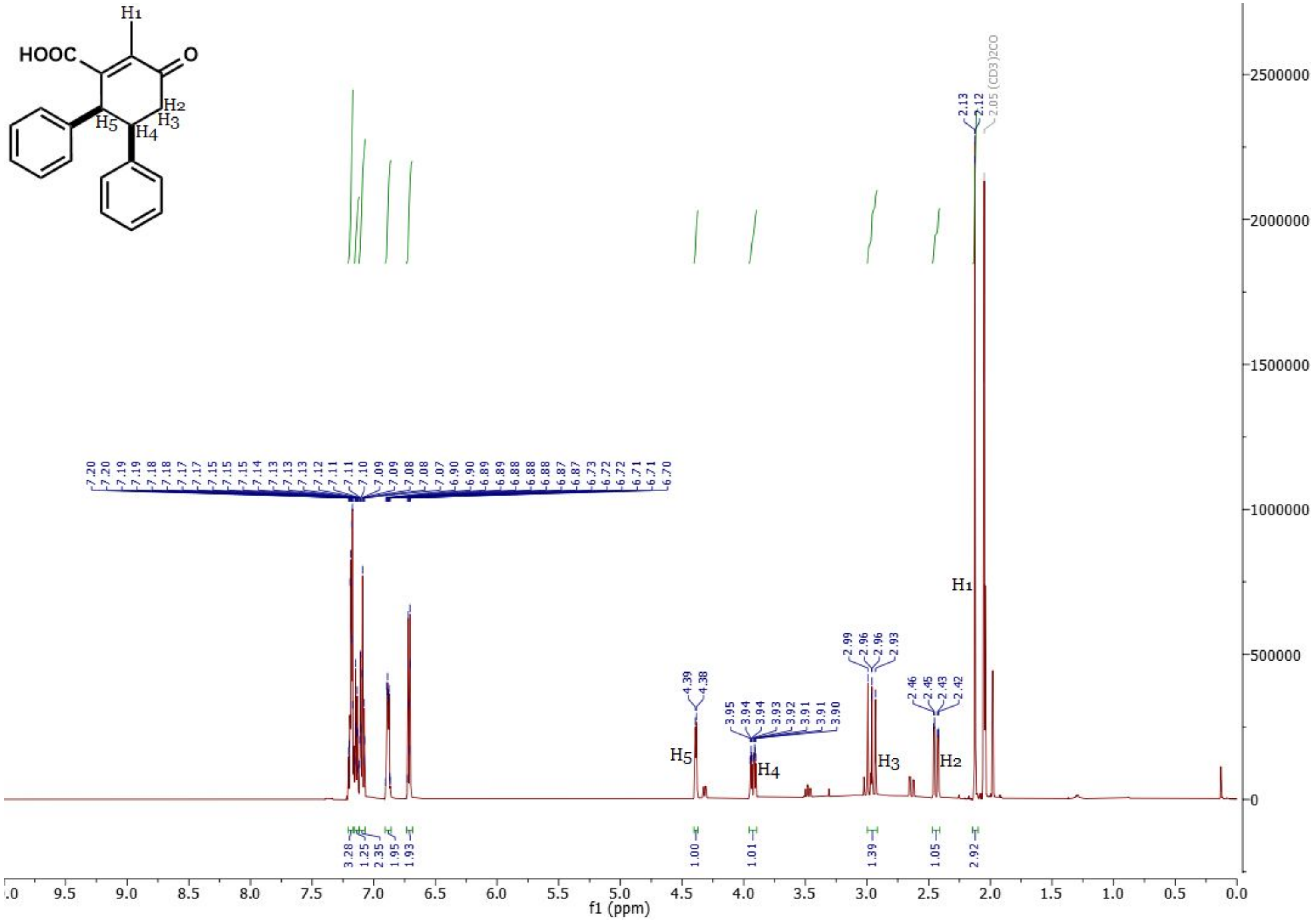

Figure S145: ^{1}H NMR spectrum of enriched ***13b*** *(the spectrum is mixture of* ***13b*** *and* ***13a****) (500 MHz, acetone-d6): δ 7.21-7.17 (m, 3H), 7.15-7.12 (m, 1H), 7.11-7.07 (m, 2H), 6.90-6.86 (m, 2H), 6.74-6.69 (m, 2H), 4.39 (d, J = 5.1 Hz, 1H), 3.93 (ddd, J = 15.1, 5.0, 3.6 Hz, 1H), 2.96 (dd, J = 16.7, 15.1 Hz, 1H), 2.44 (dd, J = 16.7, 3.6 Hz, 1H), 2.12 (d, J = 1.3 Hz, 3H).*

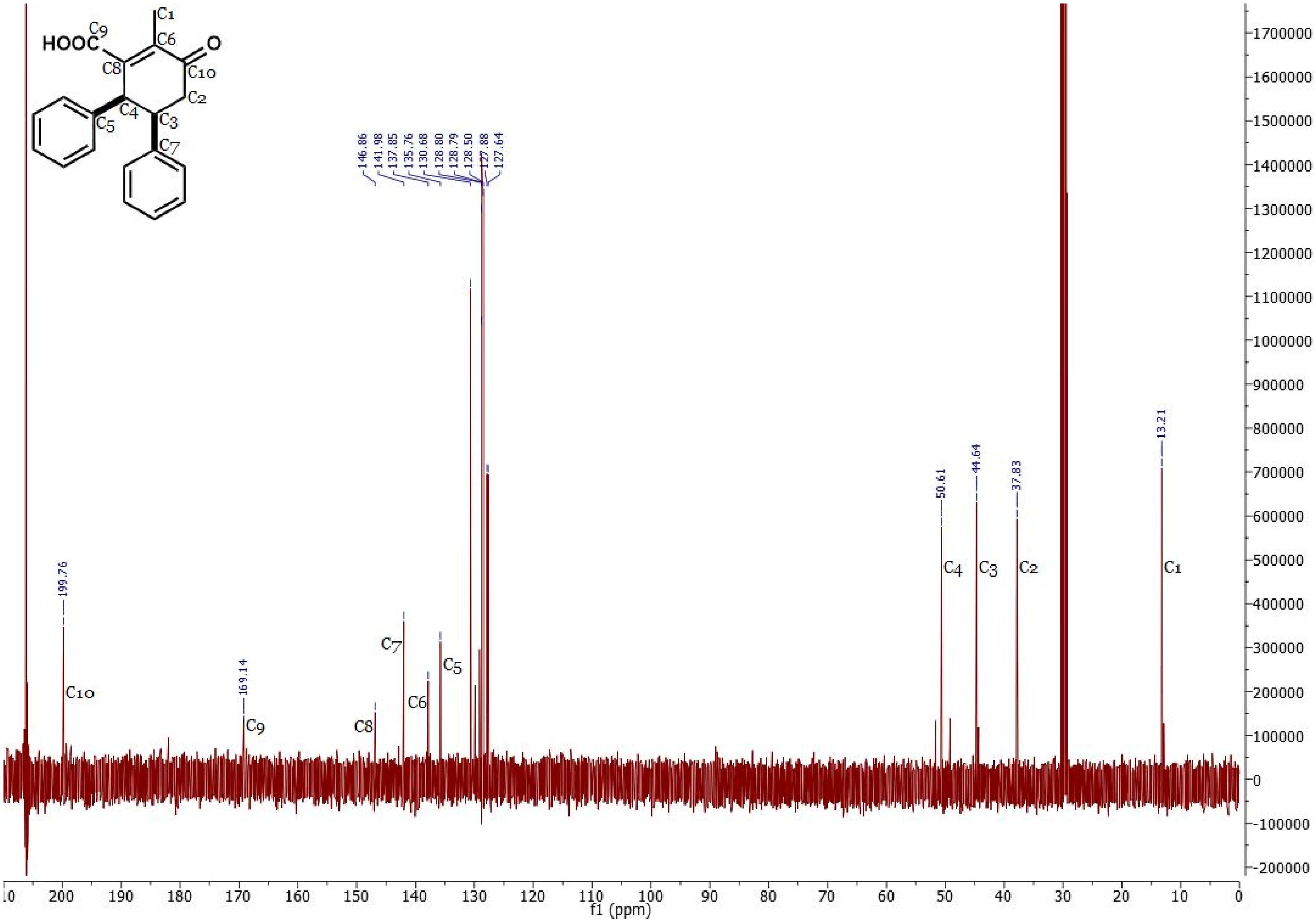

*Figure S146: $^{13}C\{^{1}H\}$ NMR spectrum of enriched **13b** (the spectrum is mixture of **13b** and **13a**) (125 MHz, acetone-d6): δ 199.76, 169.14, 146.86, 141.98, 137.85, 135.76, 130.68, 128.80, 128.79, 128.50, 127.88, 127.64, 50.61, 44.64, 37.83, 13.21.*

2D NMR of **13b**

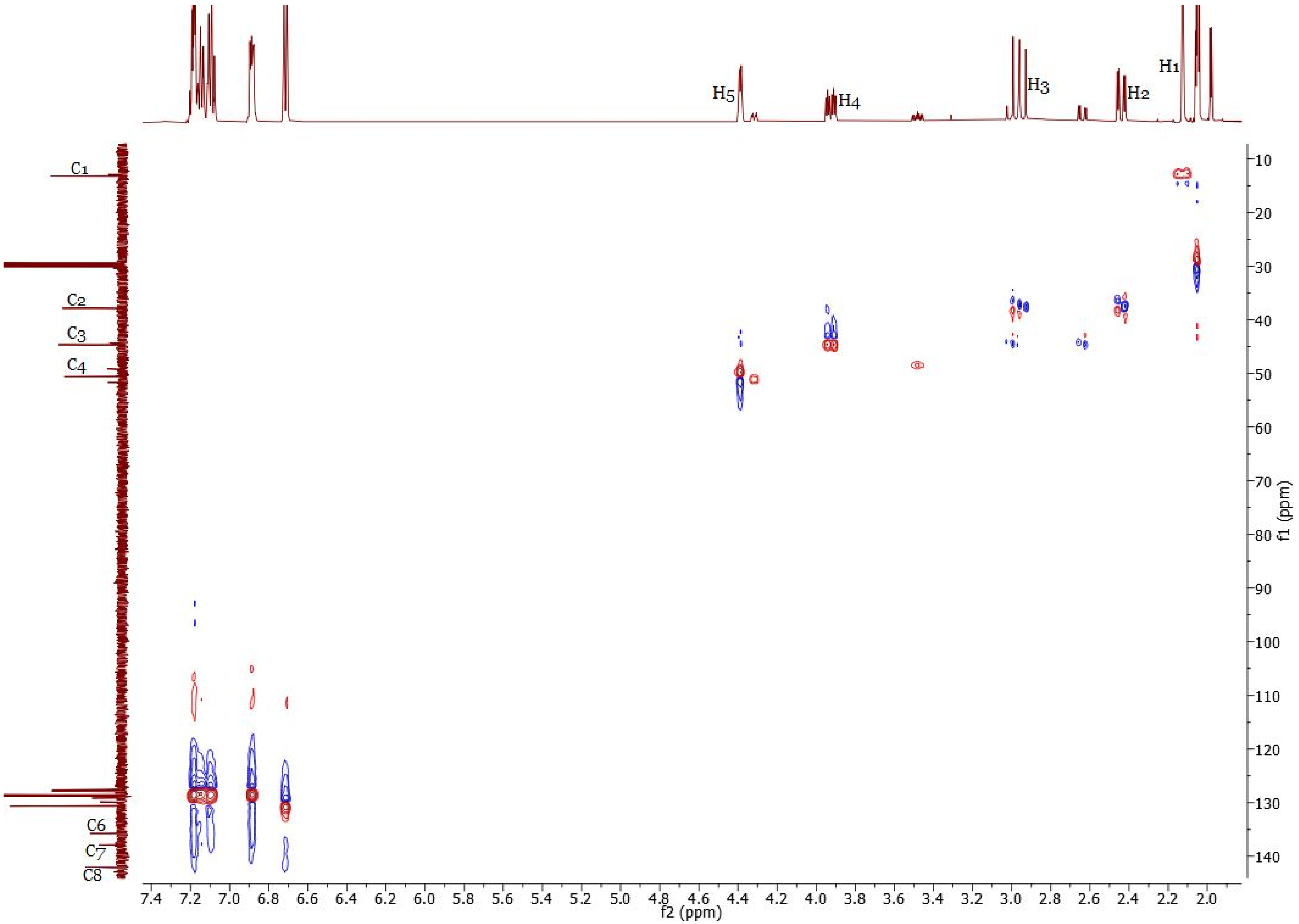

Figure S147: HSQC spectrum of enriched ***13b*** *(the spectrum is mixture of* ***13b*** *and* ***13a****).*

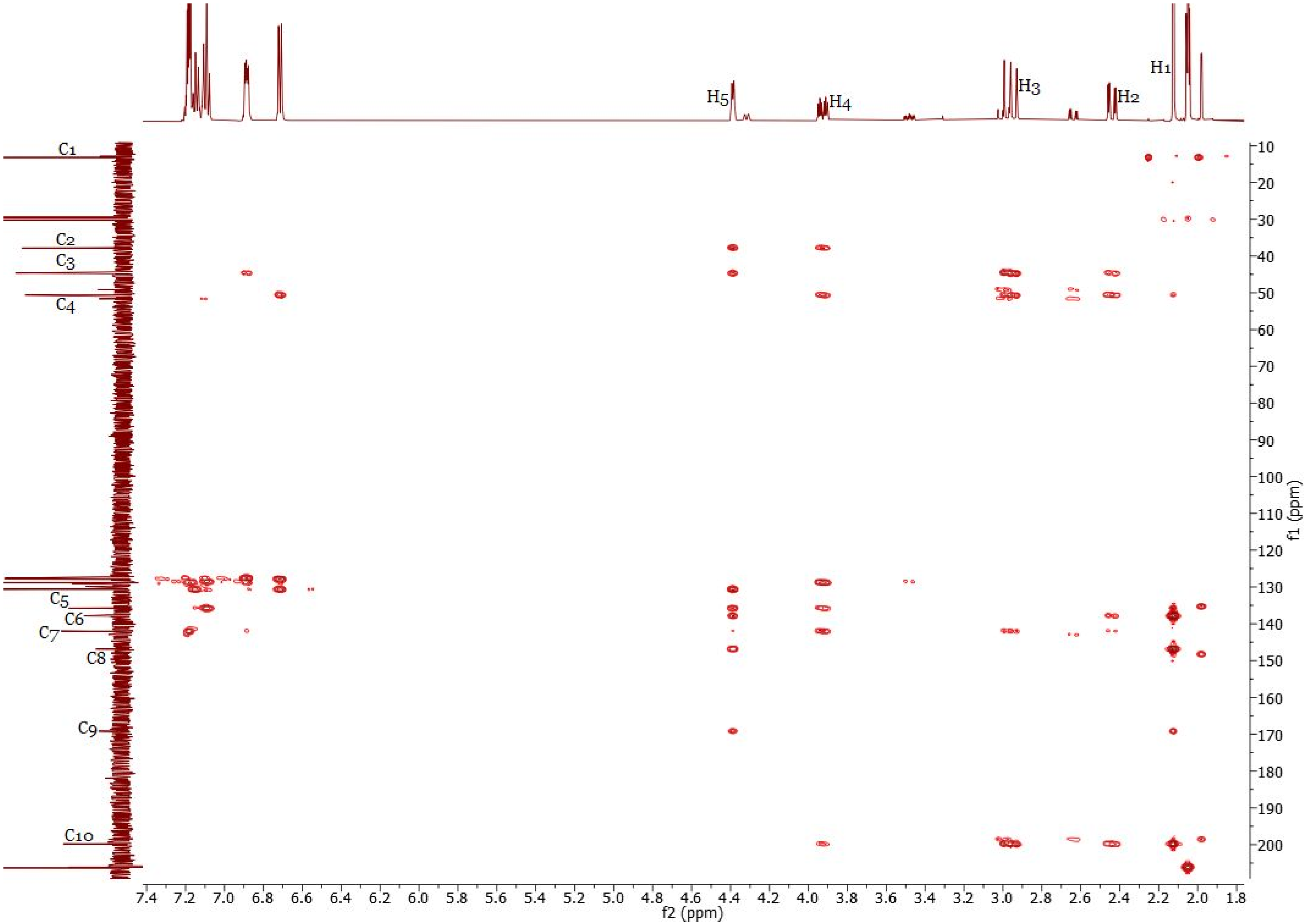

*Figure S148: HMBC spectrum of enriched **13b** (the spectrum is mixture of **13b** and **13a**).*

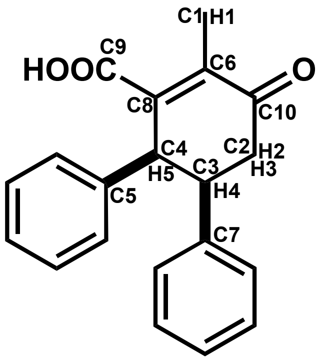

*Figure S149: 2D NMR observations of **13b**.*

2D NMR observations of **13b**

Protons H1 are attached to carbon C1 forming CH_3 group. The group has connectivity to carbons C4 (weak), C6, C8, C9 (weak) and C10.

Protons H2 and H3 are attached to carbon C2 forming CH_2 group. The group has connectivity to carbons C3, C4, C6, C7 (weak) and C10.

Proton H4 is attached to carbon C3 forming CH group. The group has connectivity to carbons C2, C4, C5 (weak), C7, C8 (weak) and C10. The group has connectivity to inside of the phenyl group, suggesting nearby location.

Proton H5 is attached to carbon C4 forming CH group. The group has connectivity to carbons C2, C3, C5, C6, C7 (weak), C8 and C9.

IR spectroscopy of **13b**

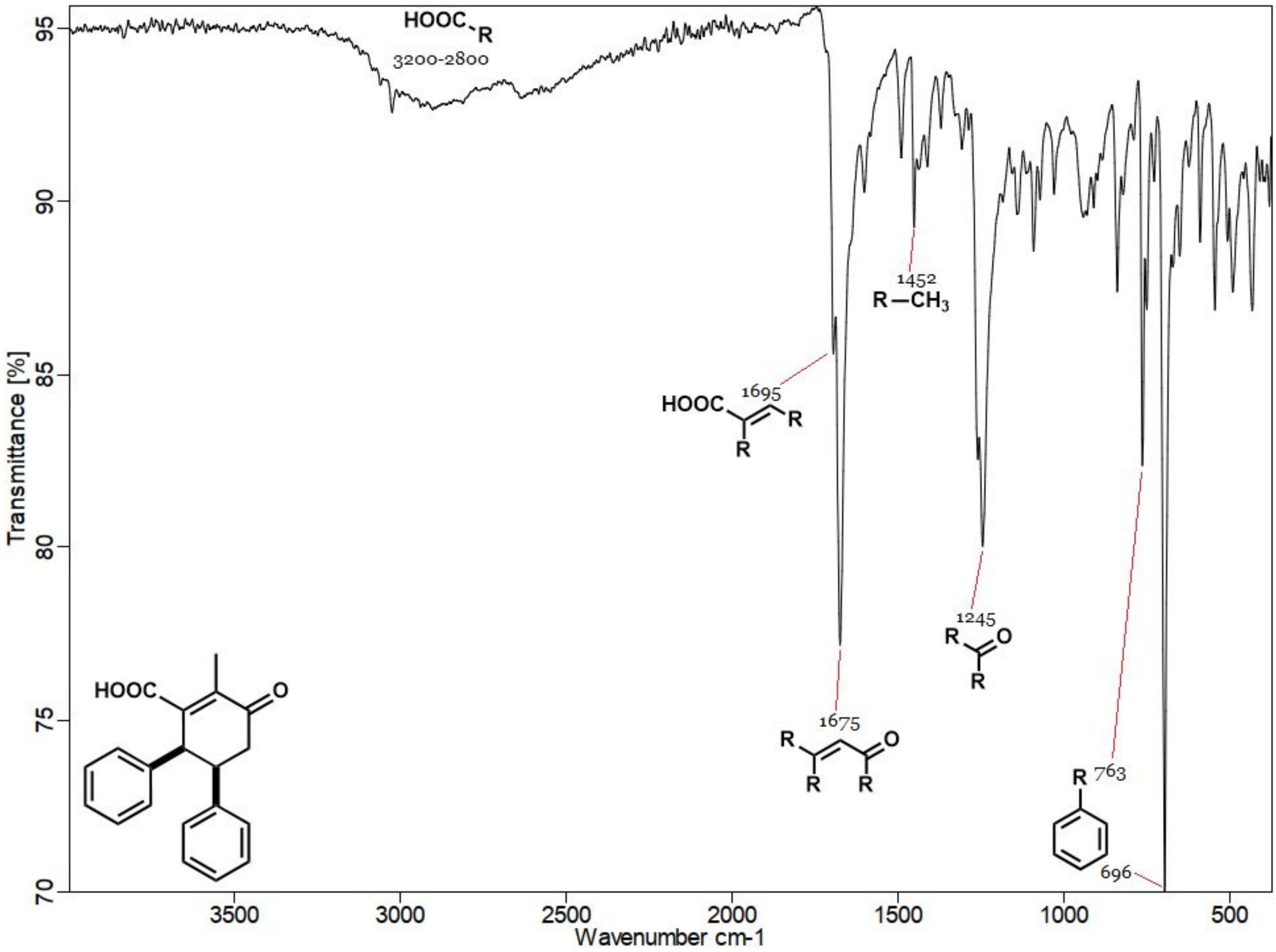

*Figure S150: IR spectrum of **13b** (2800-3200 (broad) (R-COOH), 1695 (s) (C=C-ROOH), 1675 (s) (C=C-**CO**-R), 1452 (w) (R-CH_3), 1245 (s) (R-**CO**-R), 763 (s), 696 (s) (5 adjacent H (Ph)) cm^{-1}).*

HRMS of **13b**

HRMS (ESI-TOF) m/z: [**13b**-H]$^-$ calculated for $C_{20}H_{17}O_3$ 305.1172; Found 305.1173; Error 0.108 ppm.

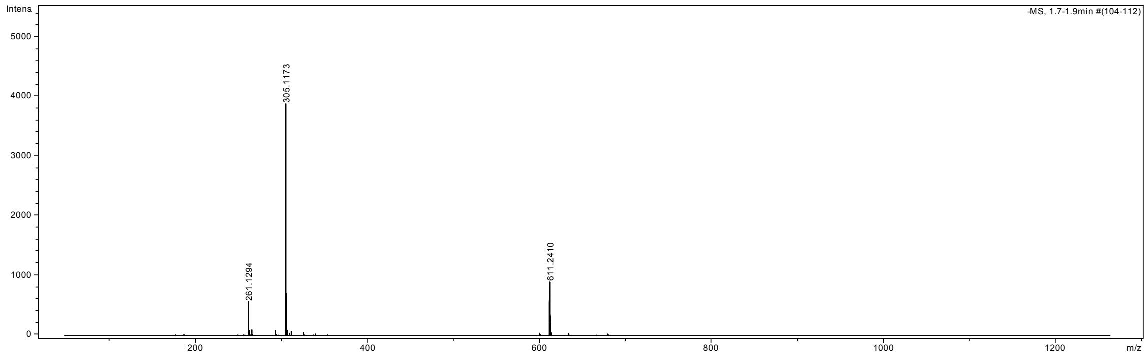

*Figure S151: ESI-TOF-MS of [**13b**-H]$^-$ (peak: 305.1173 m/z, negative-ion mode).*

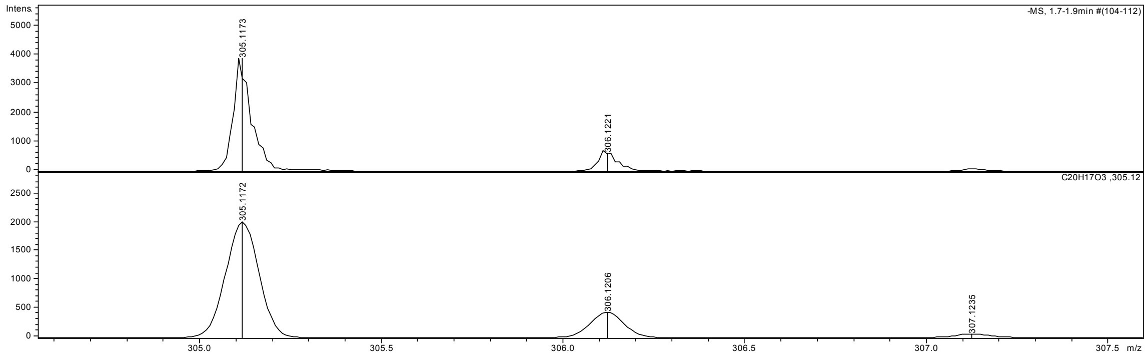

*Figure S152: Measured compound peak of [**13b**-H]$^-$ (305.1173 m/z) at top, simulated peak ($C_{20}H_{17}O_3$) below*

3.18 Spectroscopic data of 14a

1D NMR of **14a**

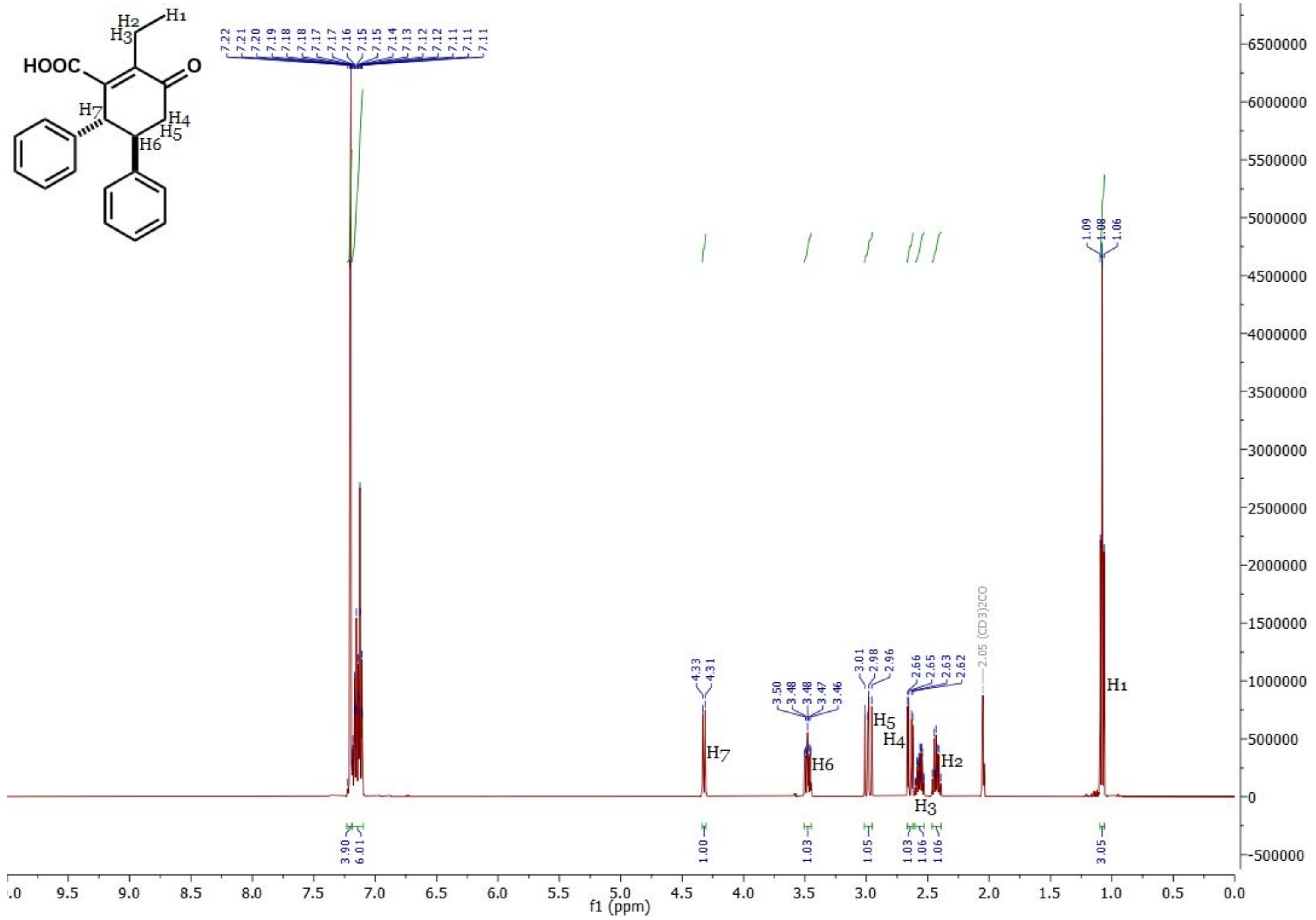

*Figure S153: ¹H NMR spectrum of **14a** (500 MHz, acetone-d6): δ 7.23-7.19 (m, 4H), 7.19-7.09 (m, 6H), 4.32 (d, J = 9.2 Hz, 1H), 3.48 (ddd, J = 12.2, 9.3, 4.0 Hz,1H), 2.98 (dd, J = 15.9, 12.3 Hz), 2.64 (dd, J = 15.9, 4.0 Hz, 1H), 2.56 (m, 1H), 2.43 (dq, J = 12.5, 7.5 Hz, 1H), 1.08 (t, J = 7.4 Hz, 3H).*

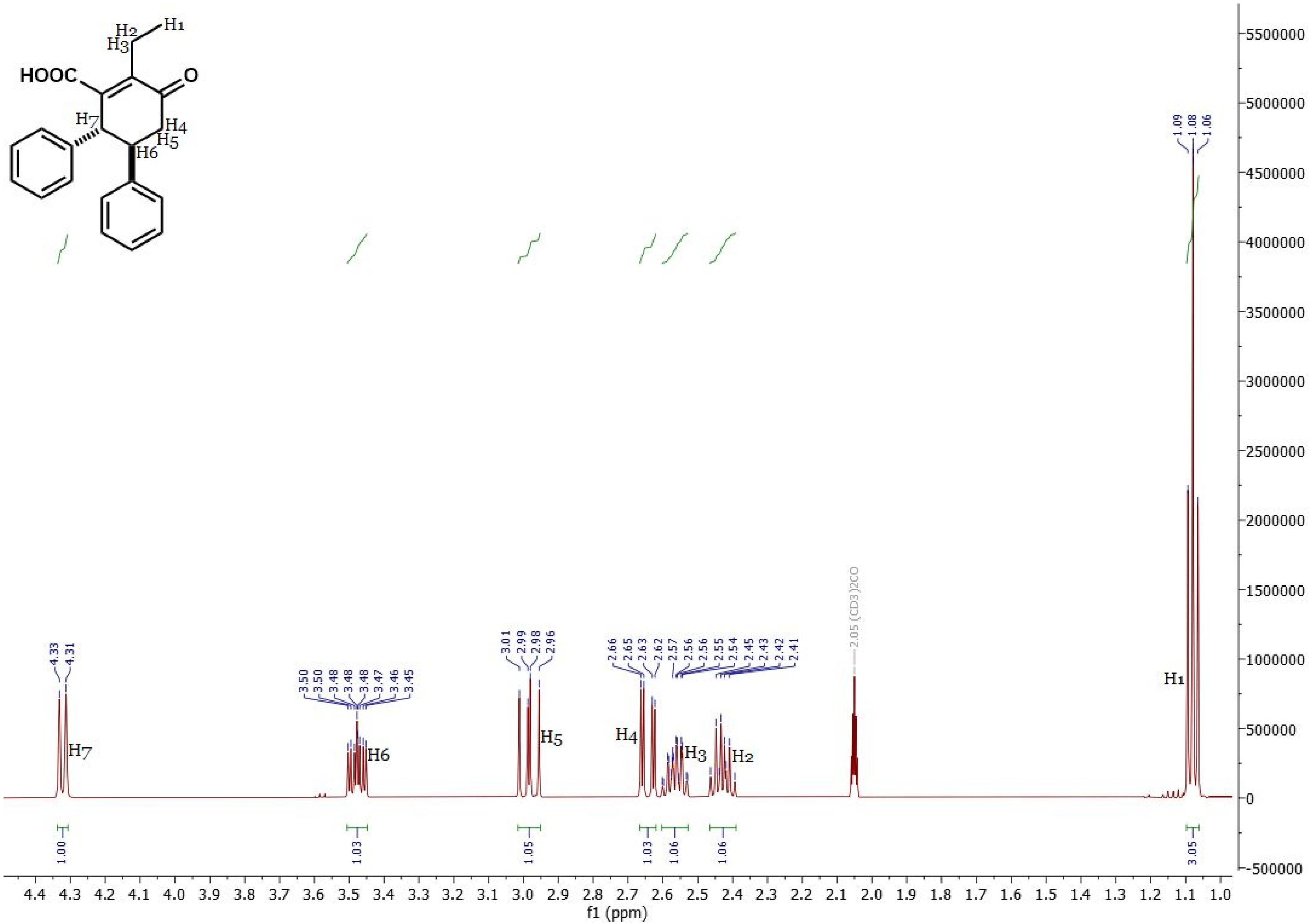

*Figure S154: ^{1}H NMR spectrum of **14a** from the aliphatic region (500 MHz, acetone-d6): δ 4.32 (d, J = 9.2 Hz, 1H), 3.48 (ddd, J = 12.2, 9.3, 4.0 Hz,1H), 2.98 (dd, J = 15.9, 12.3 Hz), 2.64 (dd, J = 15.9, 4.0 Hz, 1H), 2.56 (m, 1H), 2.43 (dq, J = 12.5, 7.5 Hz, 1H), 1.08 (t, J = 7.4 Hz, 3H).*

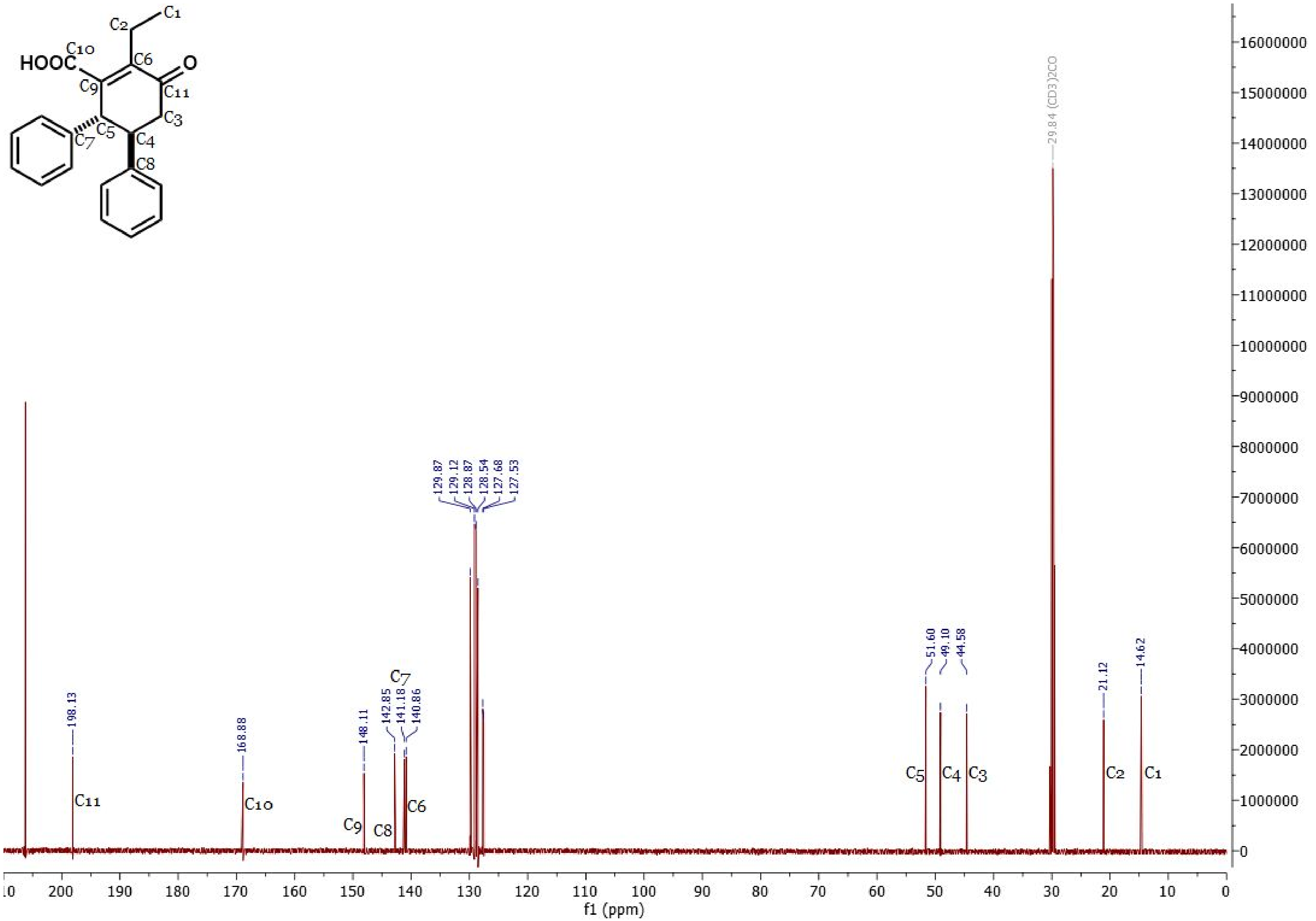

*Figure S155: $^{13}C\{^{1}H\}$ NMR spectrum of **14a** (125 MHz, acetone-d6): δ 198.13, 168.88, 148.11, 142.85, 141.18, 140.86, 129.87, 129.12, 128.87, 128.54, 127.68, 127.53, 51.60, 49.10, 44.58, 21.12, 14.62.*

2D NMR of **14a**

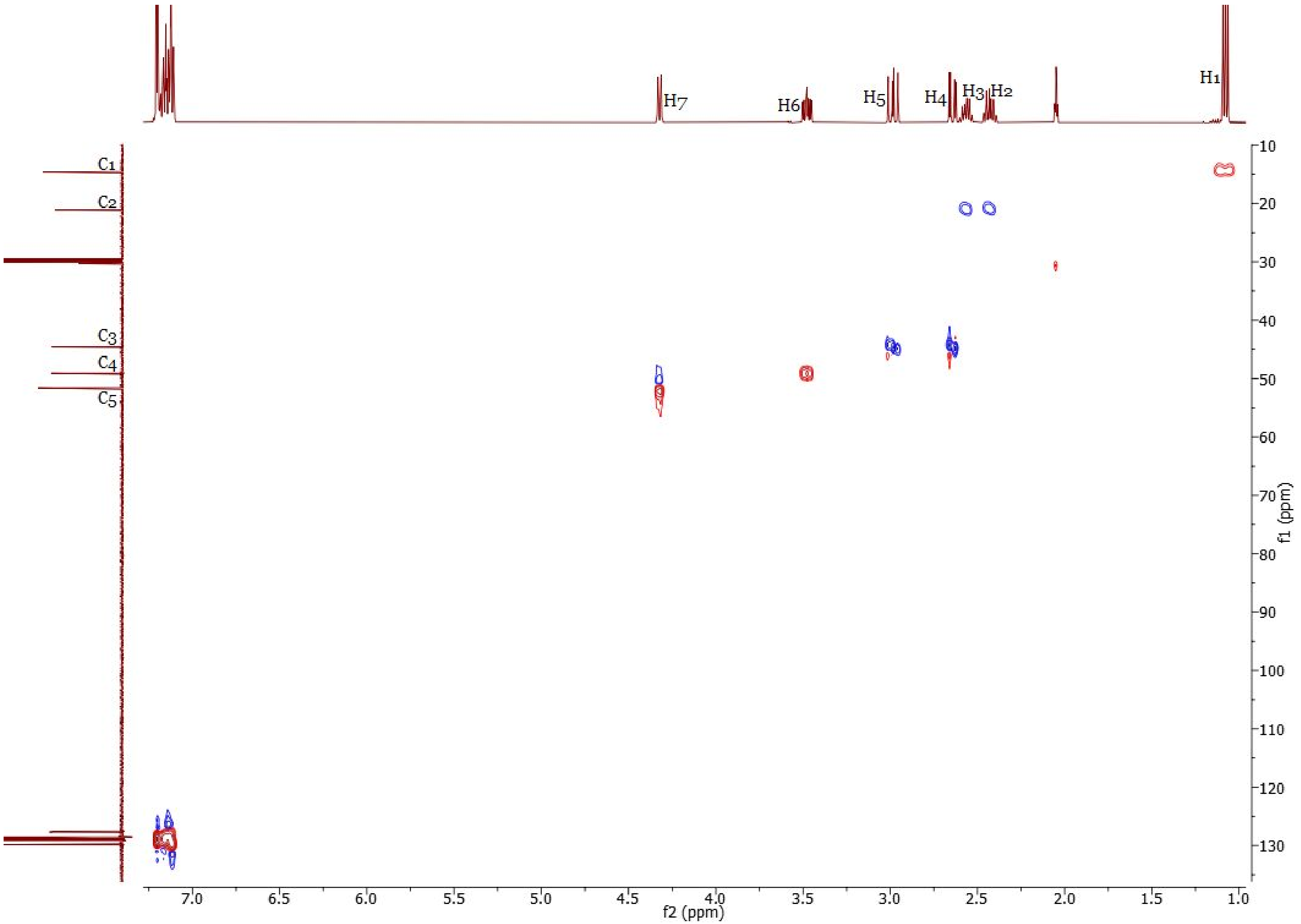

Figure S156: HSQC spectrum of ***14a****.*

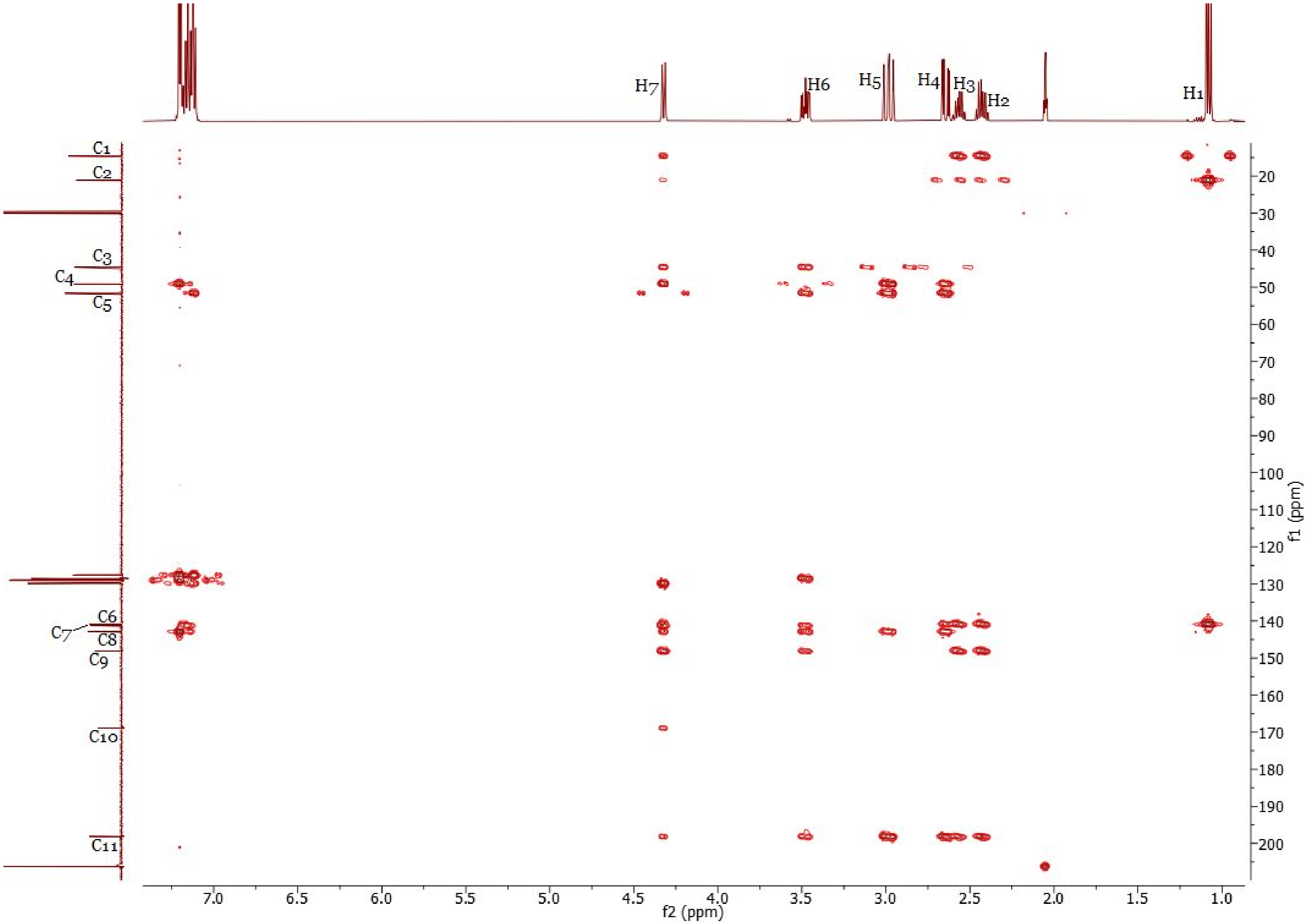

*Figure S157: HMBC spectrum of **14a**.*

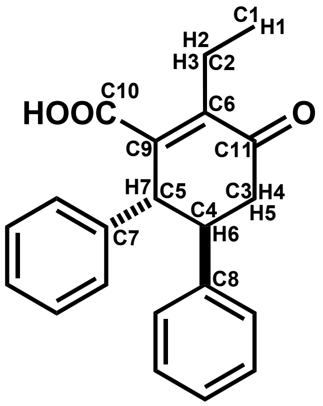

*Figure S158: 2D NMR observations for **14a**.*

2D NMR observations of **14a**:

Protons H1 are attached to carbon C1 forming CH_3 group. The group has connectivity to carbons C2 and C6.

Protons H2 and H3 are attached to carbon C2 forming CH_2 group. The group has connectivity to carbons C1, C9 and C11.

Protons H4 and H5 are attached to carbon C3 forming CH_2 group. The group has connectivity to carbons C4, C5, C6, C8 and C11.

Proton H6 is attached to carbon C4 forming CH group. The group has connectivity to carbons C3, C5, C7, C8, C9 and C11. The group has connection inside a phenyl group, suggesting nearby location.

Proton H7 is attached to carbon C5 forming CH group. The group has connectivity to carbons C1, C2 (weak), C3, C4, C6, C7, C8, C9, C10 and C11. The group has connection inside a phenyl group, suggesting nearby location.

IR spectroscopy of **14a**

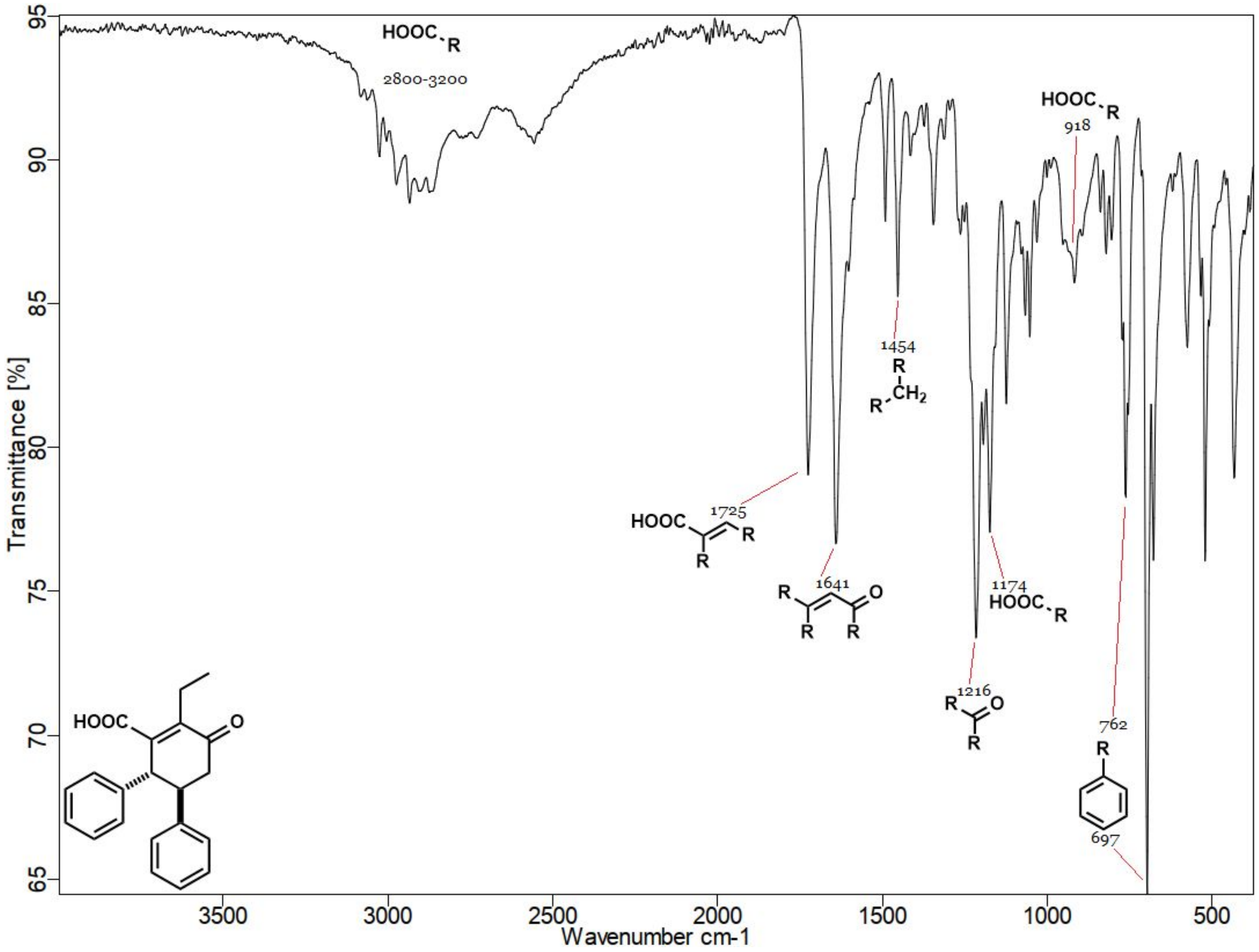

*Figure S159: IR spectrum of **14a** (2800-3200 (broad), 1174 (s), 918 (w) (R-COOH), 1725 (C=C-ROOH), 1641 (s) (C=C-**CO**-R), 1454 (m) (R-CO-**CH_2**-R), 1216 (s) (R-**CO**-R), 762 (s), 697 (s) (5 adjacent H (Ph)) cm^{-1}).*

HRMS of **14a**

HRMS (ESI-TOF) m/z: [**14a**-H]$^-$ calculated for $C_{21}H_{19}O_3$ 319.1329; Found 319.1331; Error 0.699 ppm.

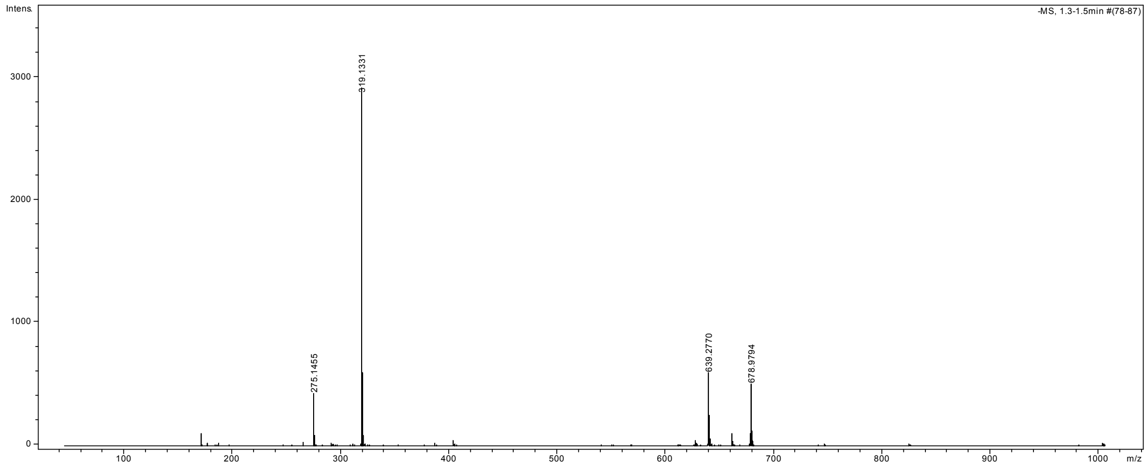

*Figure S160: ESI-TOF-MS of [**14a**-H]$^-$ (peak: 319.1331 m/z, negative-ion mode).*

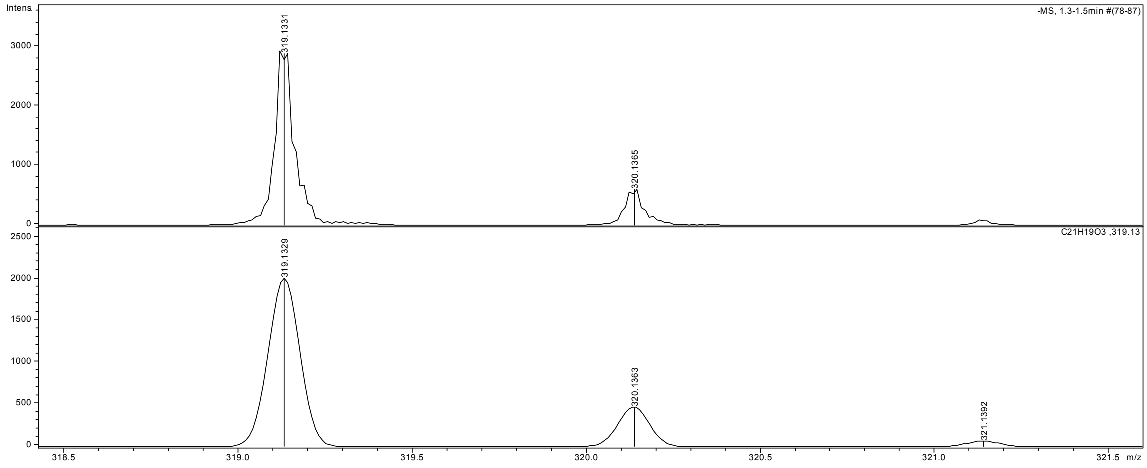

*Figure S161: Measured compound peak of [**14a**-H]$^-$ (319.1331 m/z) at top, simulated peak ($C_{21}H_{19}O_3$) below.*

3.19 Spectroscopic data of 14b

1D NMR of **14b**

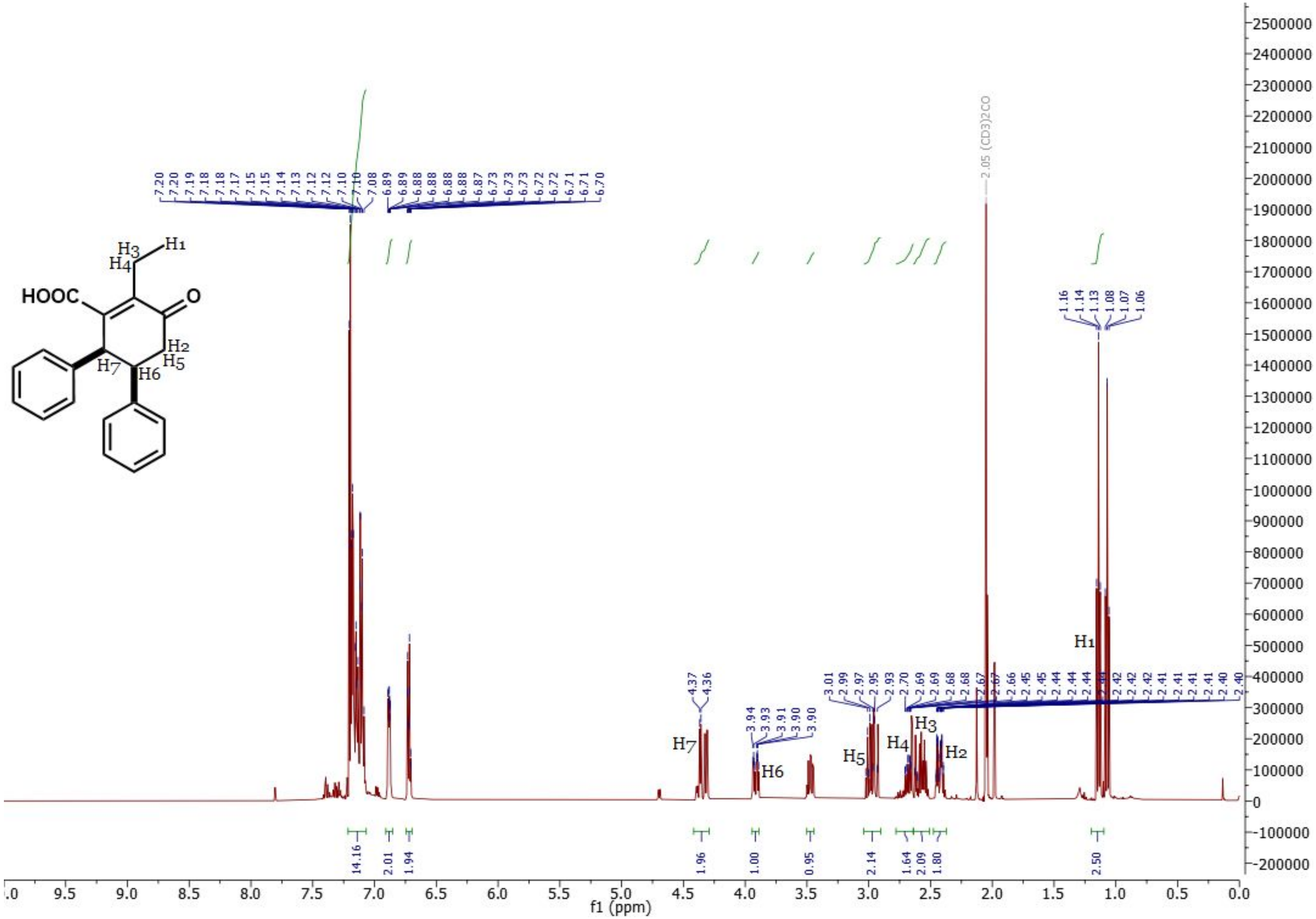

*Figure S162: ^{1}H NMR spectrum of enriched **14b** (the spectrum is mixture of **14b** and **14a**) (500 MHz, acetone-d6): δ 7.22-7.07 (m, 6H, note: heavy overlapping at aromatic region), 6.91-6.85 (m, 2H), 6.74-6.69 (m, 2H), 4.36 (d, J = 5.0 Hz, 1H), 3.92 (ddd, J = 15.1, 5.0, 3.6 Hz, 1H), 2.96 (m, 1H), 2,69 (m, 1H), 2.56 (m, 1H), 2.43 (m, 1H), 1.14 (t, J = 7.4 Hz, 3H).*

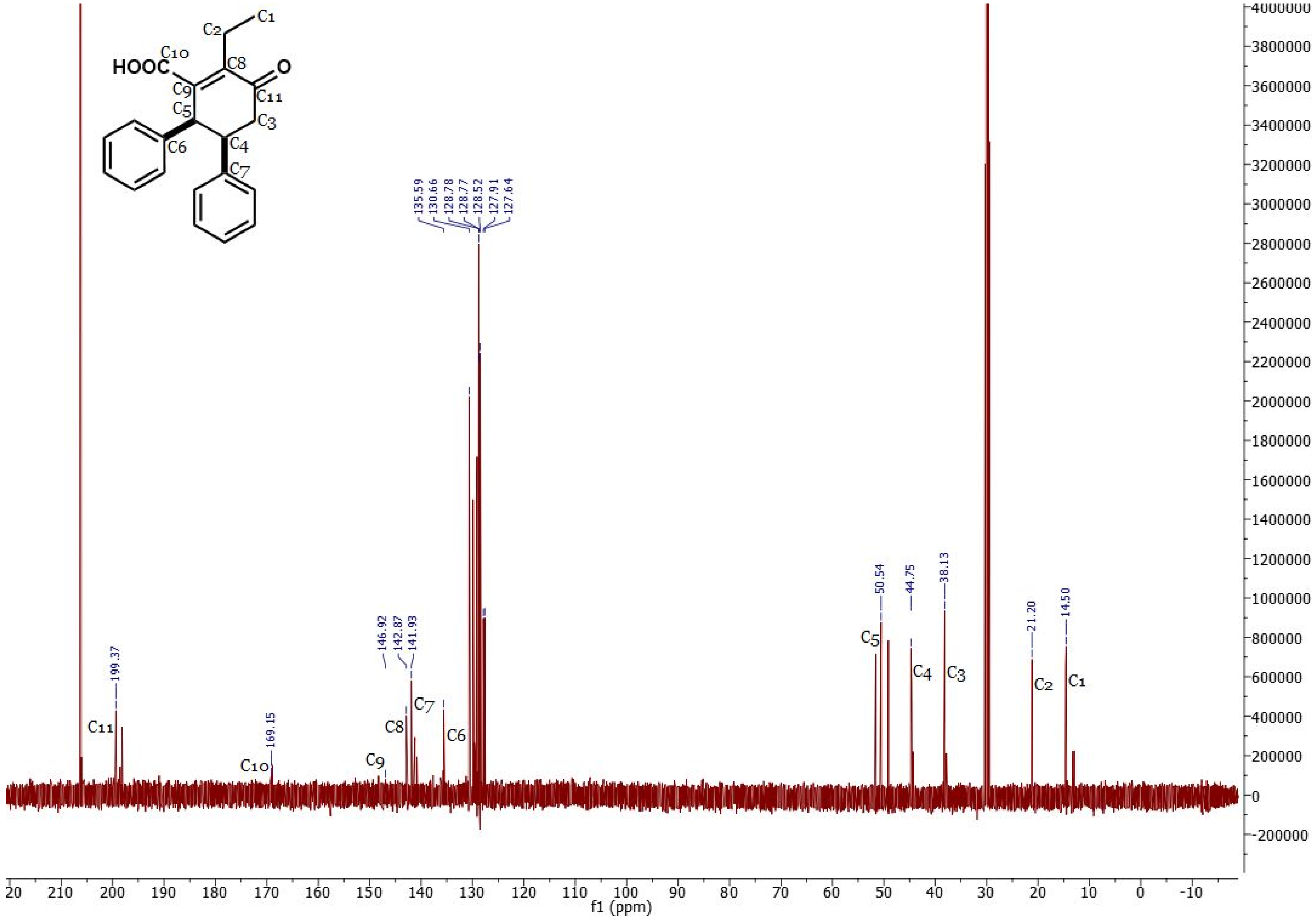

*Figure S163: $^{13}C\{^1H\}$ NMR spectrum of enriched **14b** (the spectrum is mixture of **14b** and **14a**) (125 MHz, acetone-d6): δ 199.37, 169.15, 146.92, 142.87, 141.93, 135.59, 130.66, 128.78, 128.77, 128.52, 127.91, 127.64, 50.54, 44.75, 38.13, 21.20, 14.50.*

2D NMR of **14b**

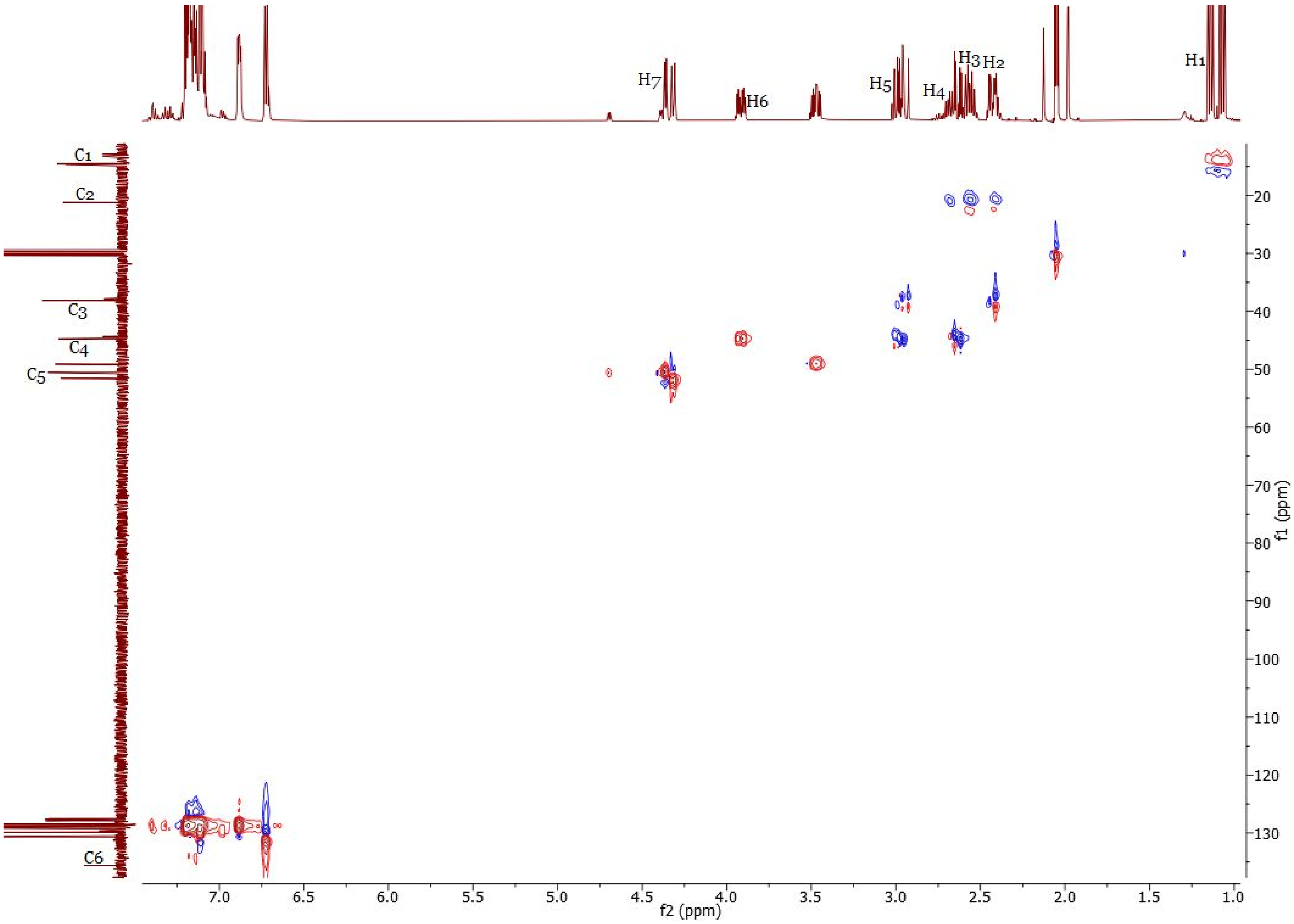

*Figure S164: HSQC spectrum of enriched **14b** (the spectrum is mixture of **14b** and **14a**).*

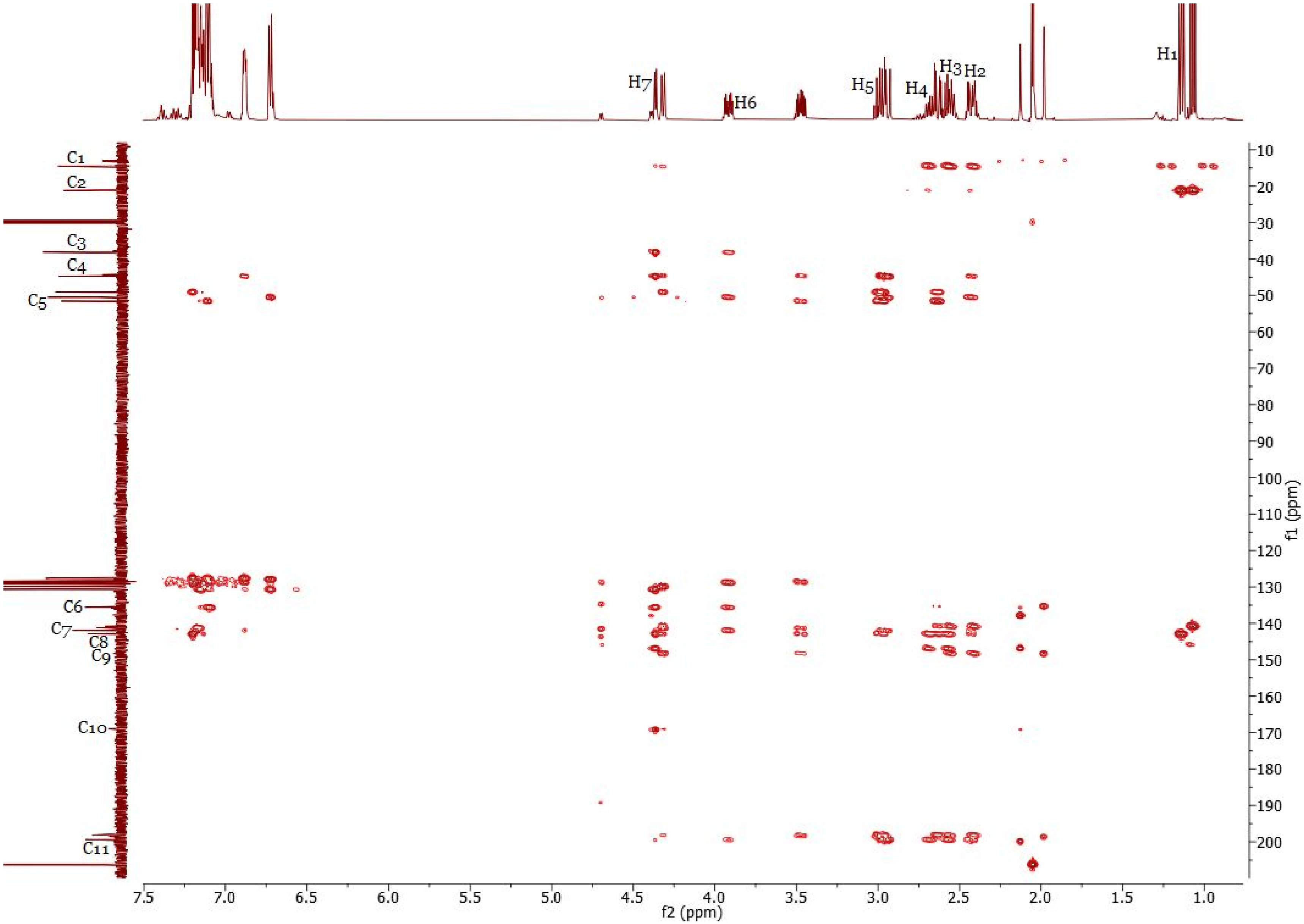

*Figure S165: HMBC spectrum of enriched **14b** (the spectrum is mixture of **14b** and **14a**).*

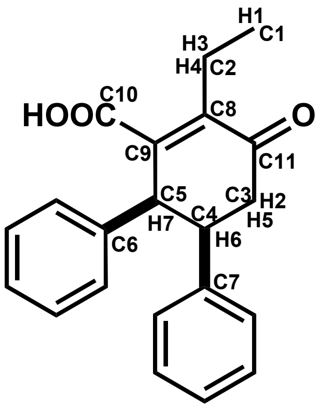

*Figure S166: 2D NMR observations of **14b**.*

2D NMR observations for **14b**:

Protons H1 are attached to carbon C1 forming CH_3 group. The group has connectivity to carbons C2 and C8.

Protons H2 and H5 are attached to carbon C3 forming CH_2 group. The group has connectivity to carbons C4, C5, C7, C8 and C11.

Protons H3 and H4 are attached to carbon C2 forming CH_2 group. The group has connectivity to carbons C1, C8, C9 and C11

Proton H6 is attached to carbon C4 forming CH group. The group has connectivity to carbons C3, C5, C6, C7 and C11. The group has connectivity inside a phenyl group, suggesting nearby location.

Proton H7 is connected to carbon C5 forming CH group. The group has connectivity to carbons C3, C4, C6, C8, C9, C10 and C11 (weak).

IR spectroscopy of **14b**

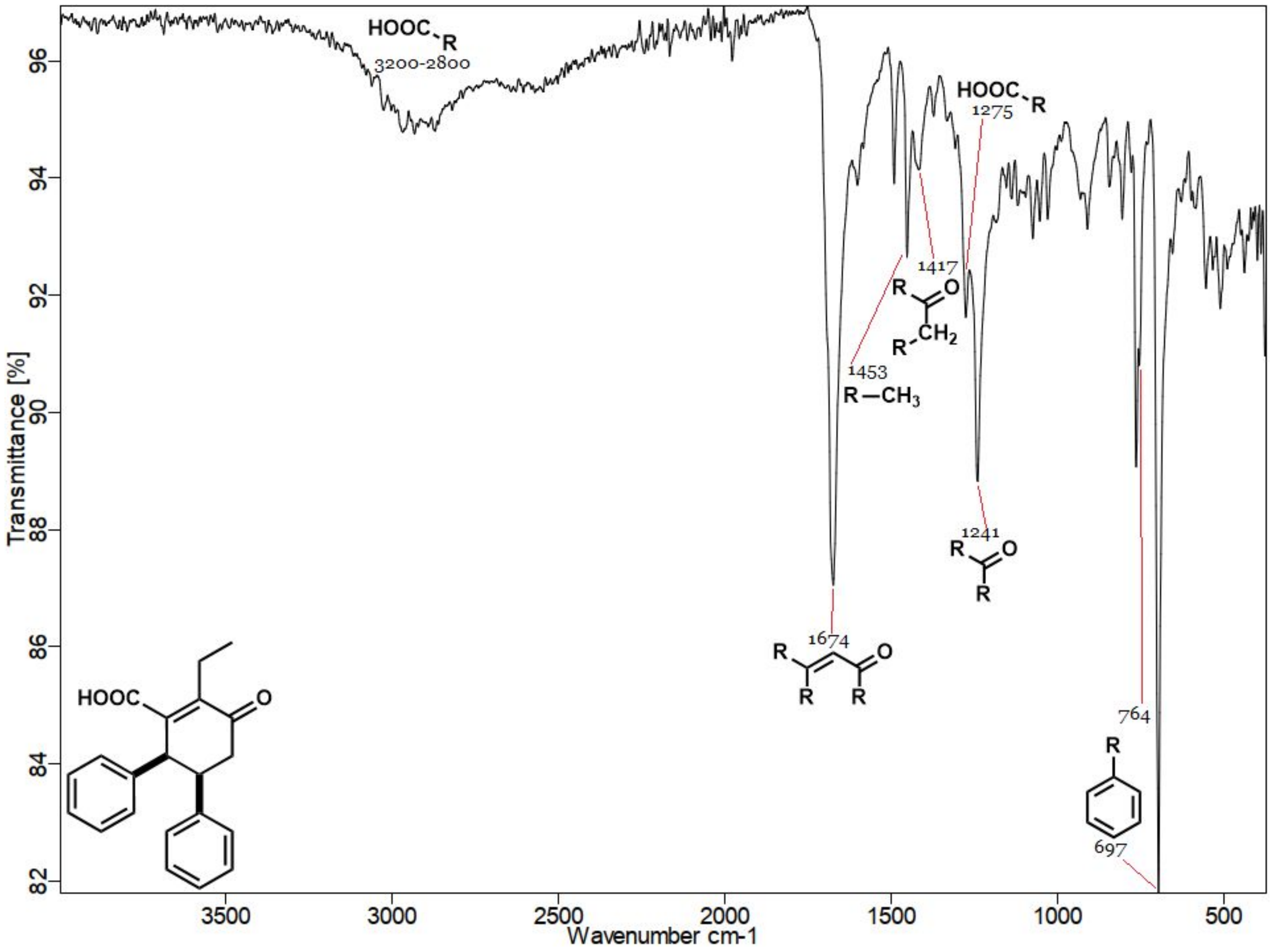

Figure S167: IR spectrum of ***14b*** *(2800-3200 (broad), 1275 (m) (R-COOH), 1674 (s) (C=C-**CO**-R), 1453 (m) (R-CH_3), 1417 (w) (R-CO-**CH_2**-R), 1241 (s) (R-**CO**-R), 764 (s), 697 (s) (5 adjacent H (Ph)) cm^{-1}).*

HRMS of **14b**

HRMS (ESI-TOF) m/z: [**14b**-H]⁻ calculated for $C_{21}H_{19}O_3$ 319.1329; Found 319.1329; Error 0.029 ppm.

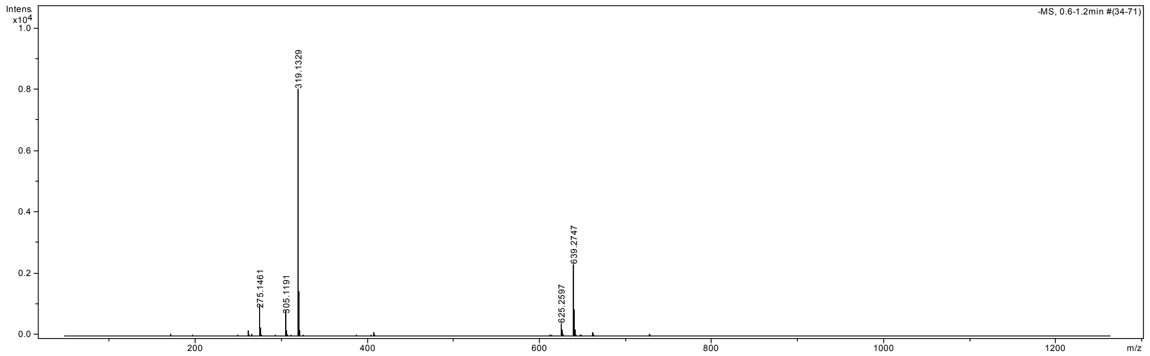

*Figure S168: ESI-TOF-MS of [**14b**-H]⁻ (peak: 319.1329 m/z, negative-ion mode).*

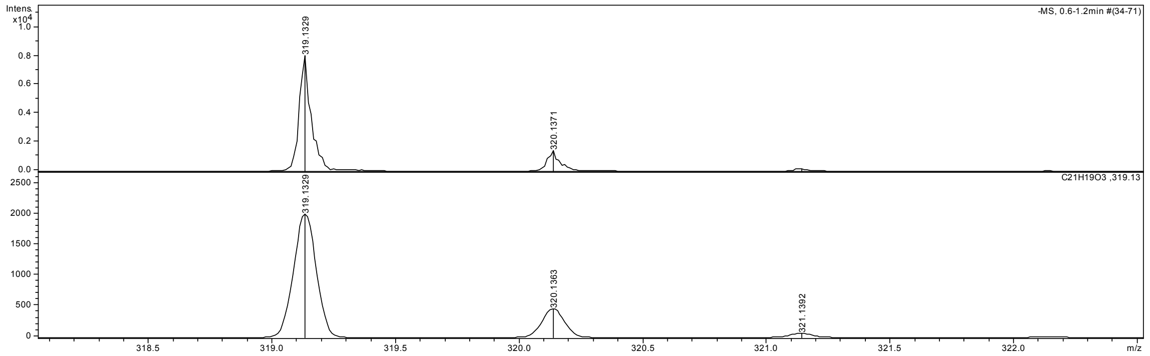

*Figure S169: Measured compound peak of [**14b**-H]⁻ (319.1329 m/z) at top, simulated peak ($C_{21}H_{19}O_3$) below.*

3.20 Spectroscopic data of 14A

1D NMR of **14A**

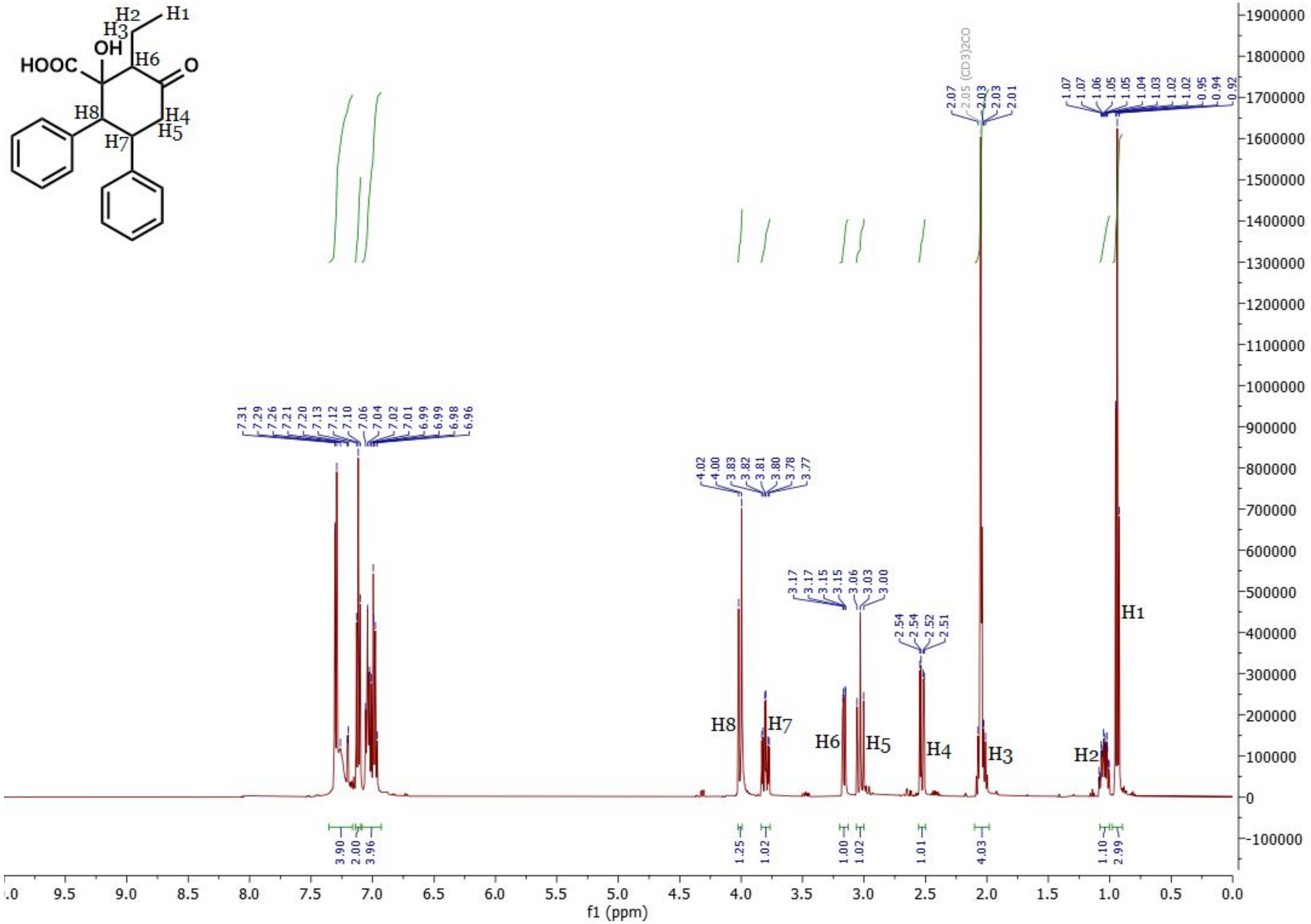

*Figure S170: ^{1}H NMR spectrum of **14A** (500 MHz, acetone-d6): δ 7.32-7.16 (m, 4H), 7.12 (t, J = 7.7 Hz, 2H), 7.06-6.94 (m, 4H), 4.01 (d, J = 12.2 Hz, 1H), 3.80 (td, J = 12.6, 4.4 Hz, 1H), 3.16 (dd, J = 9.3, 1.5 Hz, 1H), 3.03 (t, J = 13.2 Hz, 1H), 2.53 (dd, J = 13.5, 4.4 Hz, 1H), 2.04 (m, 1H), 1.04 (m, 1H), 0.94 (t, J = 7.3 Hz, 3H). Note: H3 is overlapped by acetone-d6 signal.*

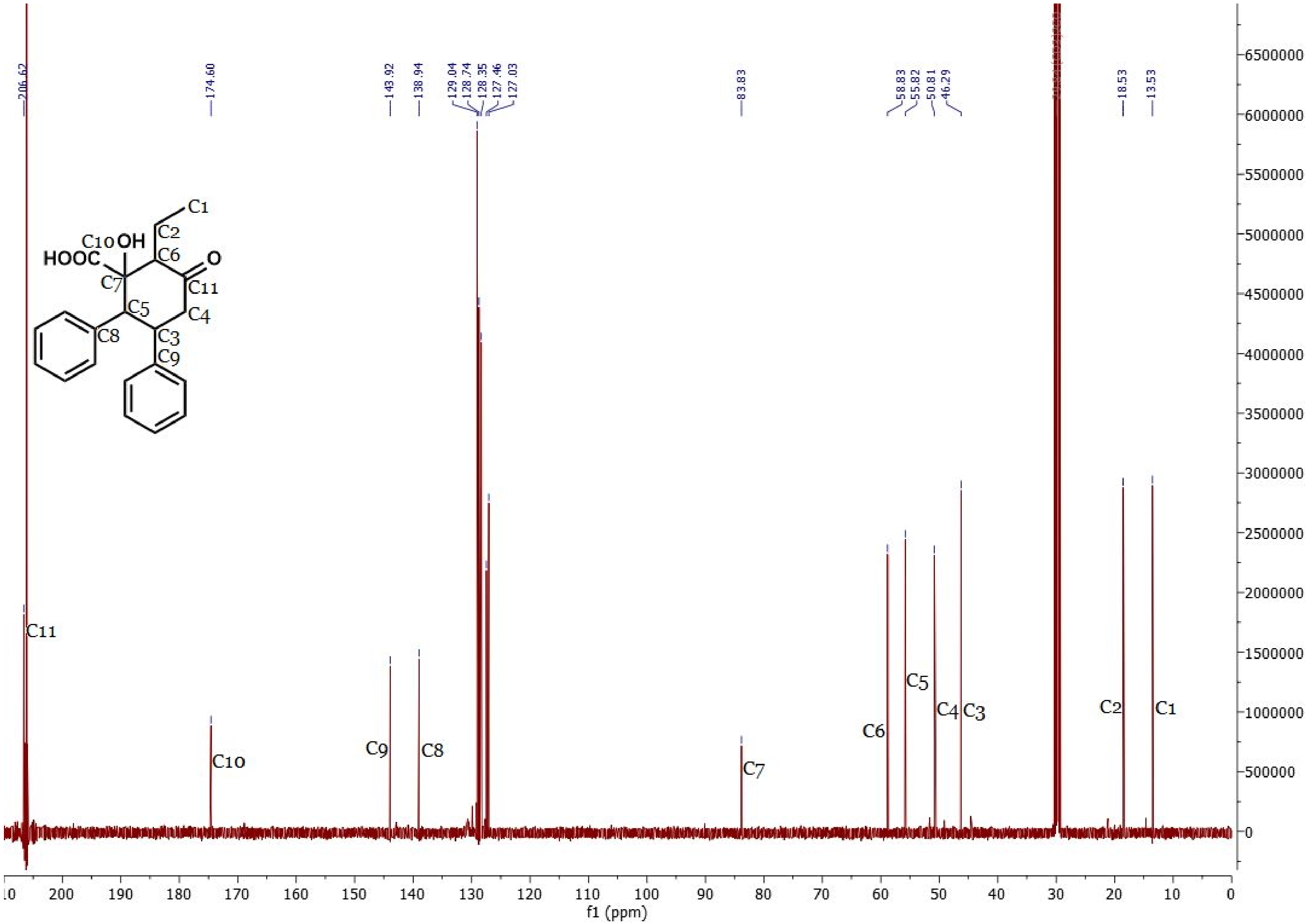

*Figure S171: ${}^{13}C\{{}^{1}H\}$ NMR spectrum of **14A** (125 MHz, acetone-d6): δ 206.62, 174.60, 143.92, 138.94, 129.04, 128.74, 128.35, 127.46, 127.03, 83.83, 58.83, 55.82, 50.81, 46.29, 18.53, 13.53. Note: one aromatic peak is missing. 128.74 is wider than usual, it could be double peak.*

2D NMR of **14A**

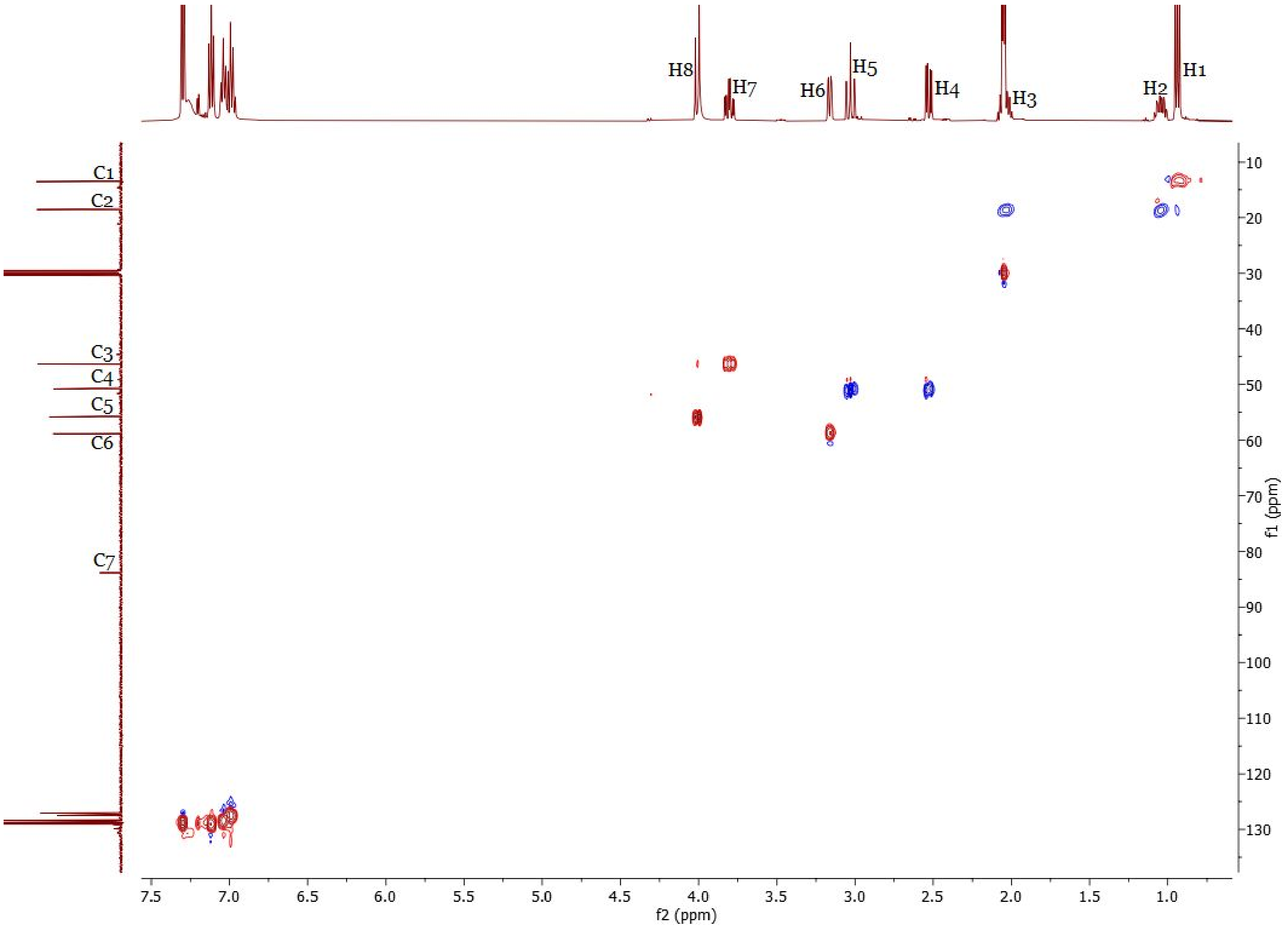

*Figure S172: HSQC spectrum of **14A**.*

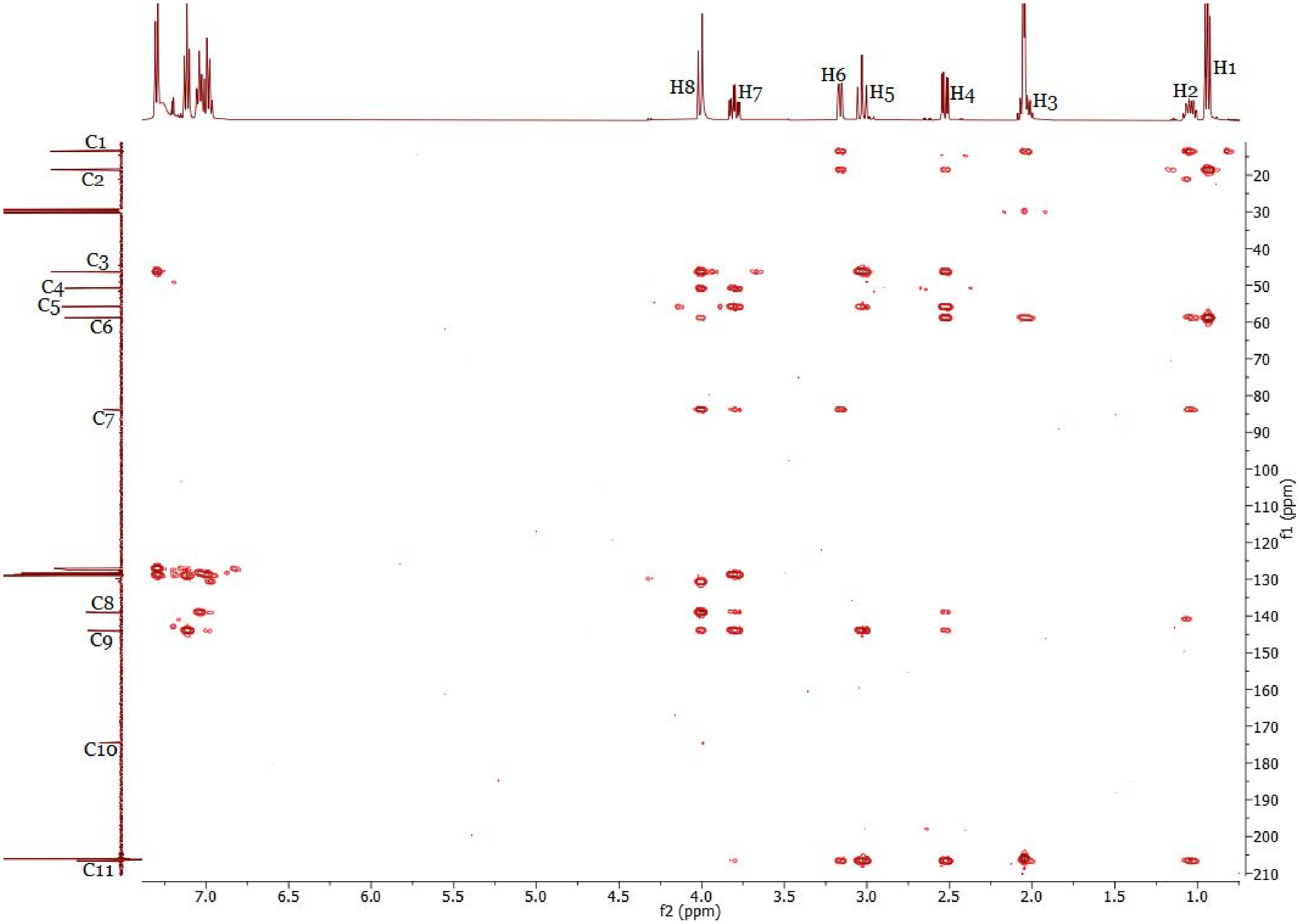

*Figure S173: HMBC spectrum of **14A**.*

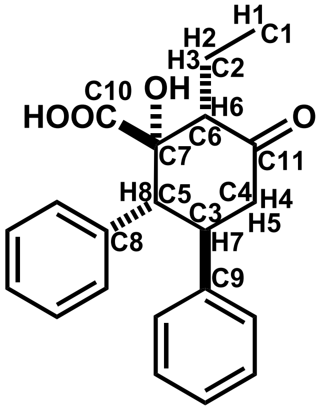

*Figure S174: 2D observations of **14A**.*

2D NMR observations of **14A**:

Protons H1 are attached to carbon C1 forming CH_3 group. The group has connectivity to carbons C2 and C6.

Protons H2 and H3 are attached to carbon C2 forming CH_2 group. The group has connectivity to carbons C1, C6, C7 and C11.

Protons H4 and H5 are attached to carbon C4 forming CH_2 group. The group has connectivity to carbons C2 (weak), C3, C5, C6, C8 (weak), C9 and C11.

Proton H6 is attached to carbon C6 forming CH group. The group has connectivity to carbons C1, C2, C7 and C11.

Proton H7 is attached to carbon C3 forming CH group. The group has connectivity to carbons C4, C5, C7, C8, C9 and C11 (very weak). The group has connectivity inside one of the aromatic ring, suggesting nearby location.

Proton H8 is attached to carbon C5 forming CH group. The group has connectivity to carbons C3, C4, C6, C7, C8 and C9. The group has connectivity inside one of the aromatic ring, suggesting nearby location.

IR spectroscopy of **14A**

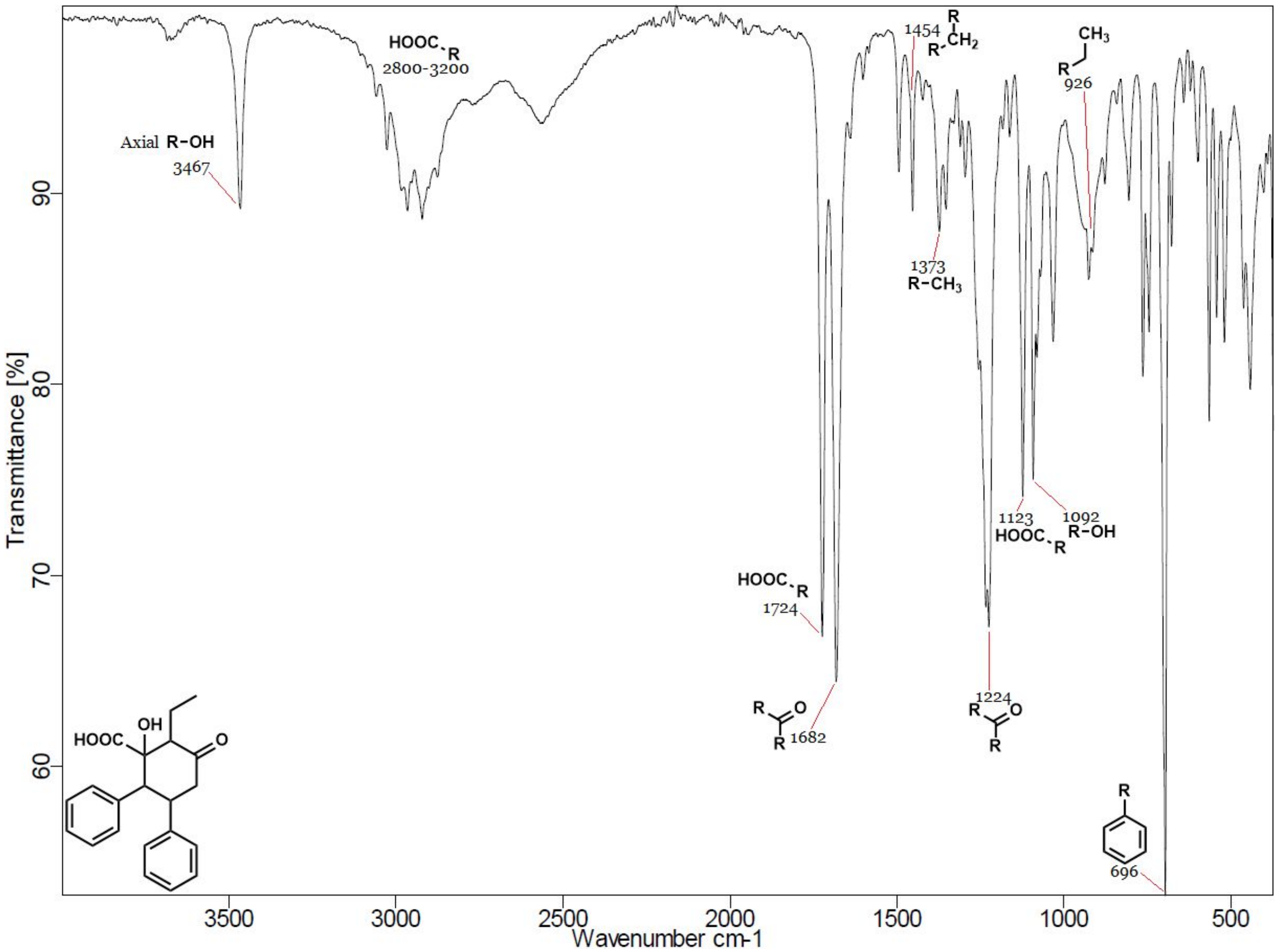

*Figure S175: IR spectrum of **14A** (3467 (m, sharp) (Axial -OH), 2800-3200 (broad), 1724 (s), 1123 (R-COOH), 1682 (s), 1224 (s) (R-**CO**-R), 1454 (m) (R-CH_2-R), 1373(m), 926 (m), (R-CH_3), 1092 (s) (R-OH), 696 (s) (5 adjacent H (Ph)) cm^{-1}).*

HRMS of **14A**

HRMS (ESI-TOF) m/z: [**14A**-H]$^-$ calculated for $C_{21}H_{21}O_4$ 337.1434; Found 337.1438; Error 1.057 ppm.

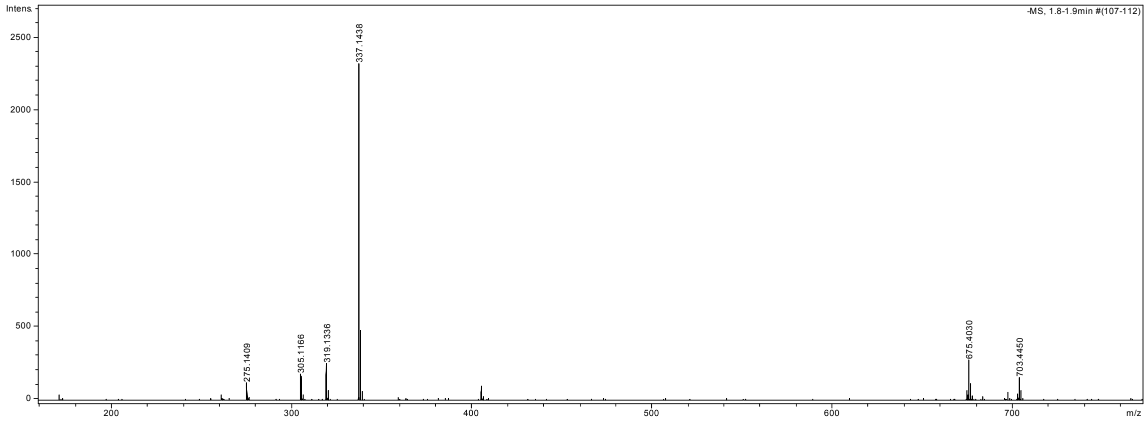

*Figure S176: ESI-TOF-MS of [**14A**-H]$^-$ (peak: 337.1438 m/z, negative-ion mode).*

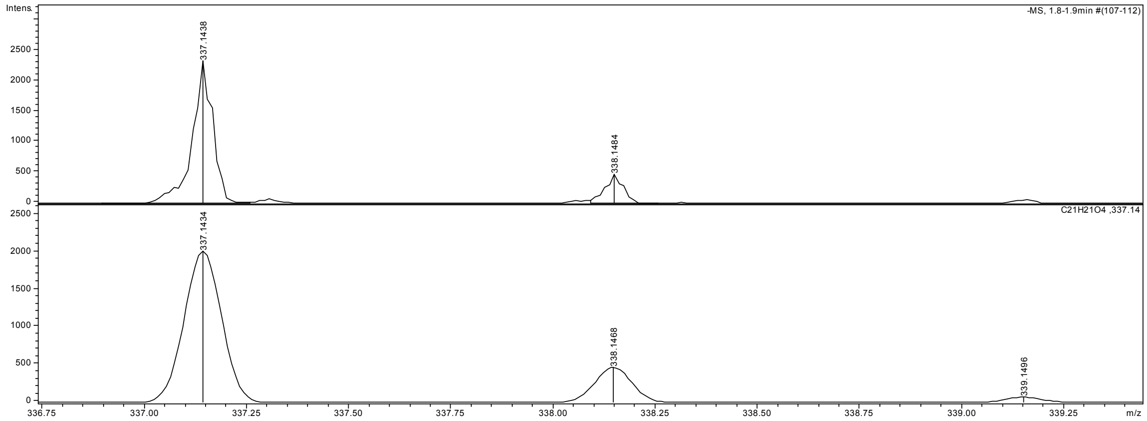

*Figure S177: Measured compound peak of [**14A**-H]$^-$ (337.1438 m/z) at top, simulated peak ($C_{21}H_{21}O_4$) below.*

3.21 Spectroscopic data of 15a

1D NMR of **15a**

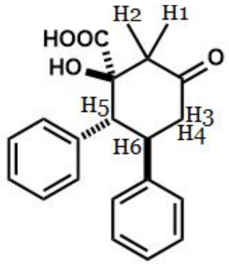

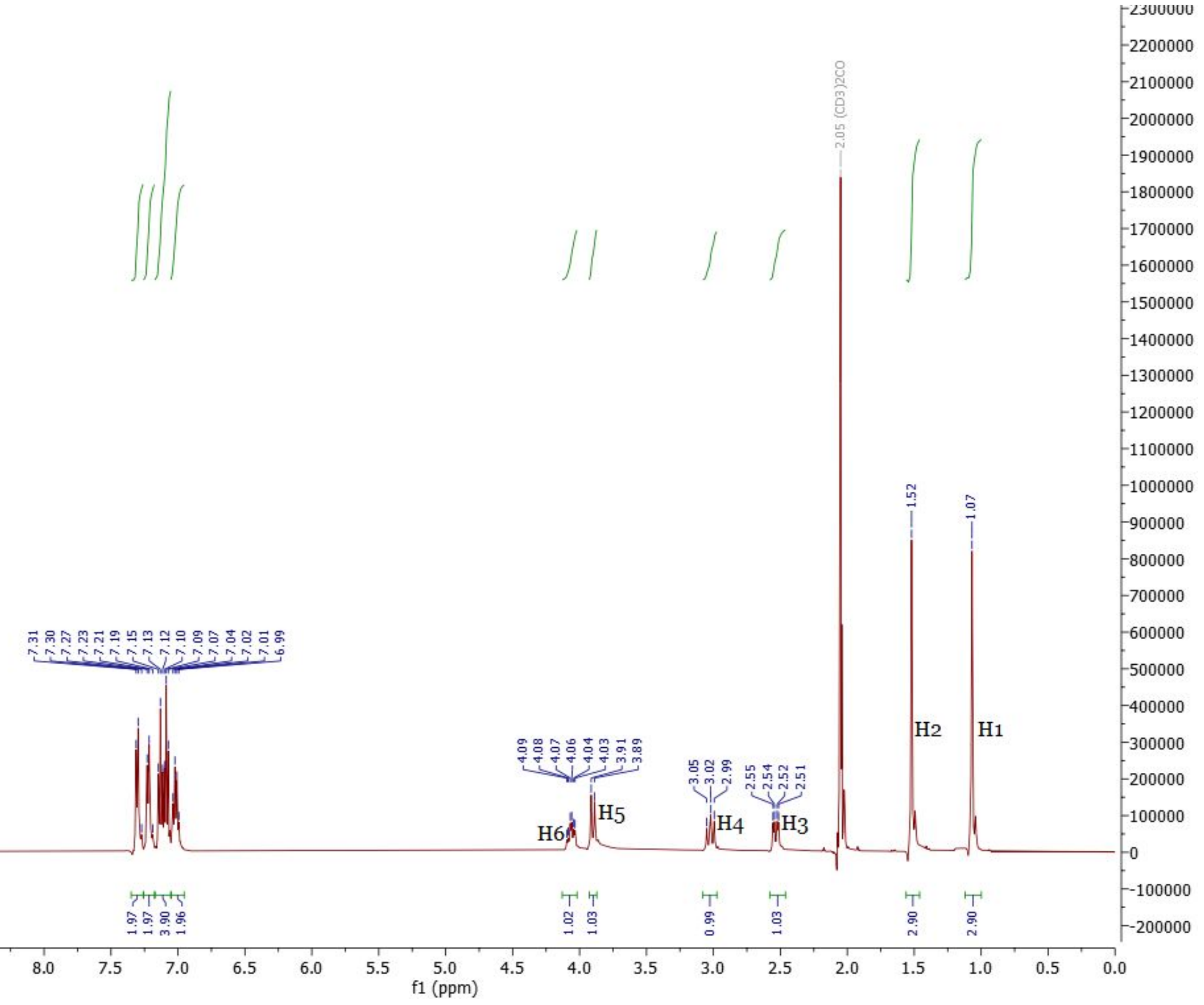

*Figure S178: ¹H NMR spectrum of **15a** (500 MHz, acetone-d6): δ 7.31 (m, 2H), 7.22 (m, 2H), 7.16 – 7.05 (m, 4H), 7.02 (m, 2H), 4.06 (td, J = 12.6, 5.2 Hz, 1H), 3.90 (d, J = 12.5 Hz, 1H), 3.02 (m, 1H), 2.53 (dd, J = 15.4, 5.4 Hz, 1H), 1.52 (s, 3H), 1.07 (s, 3H).*

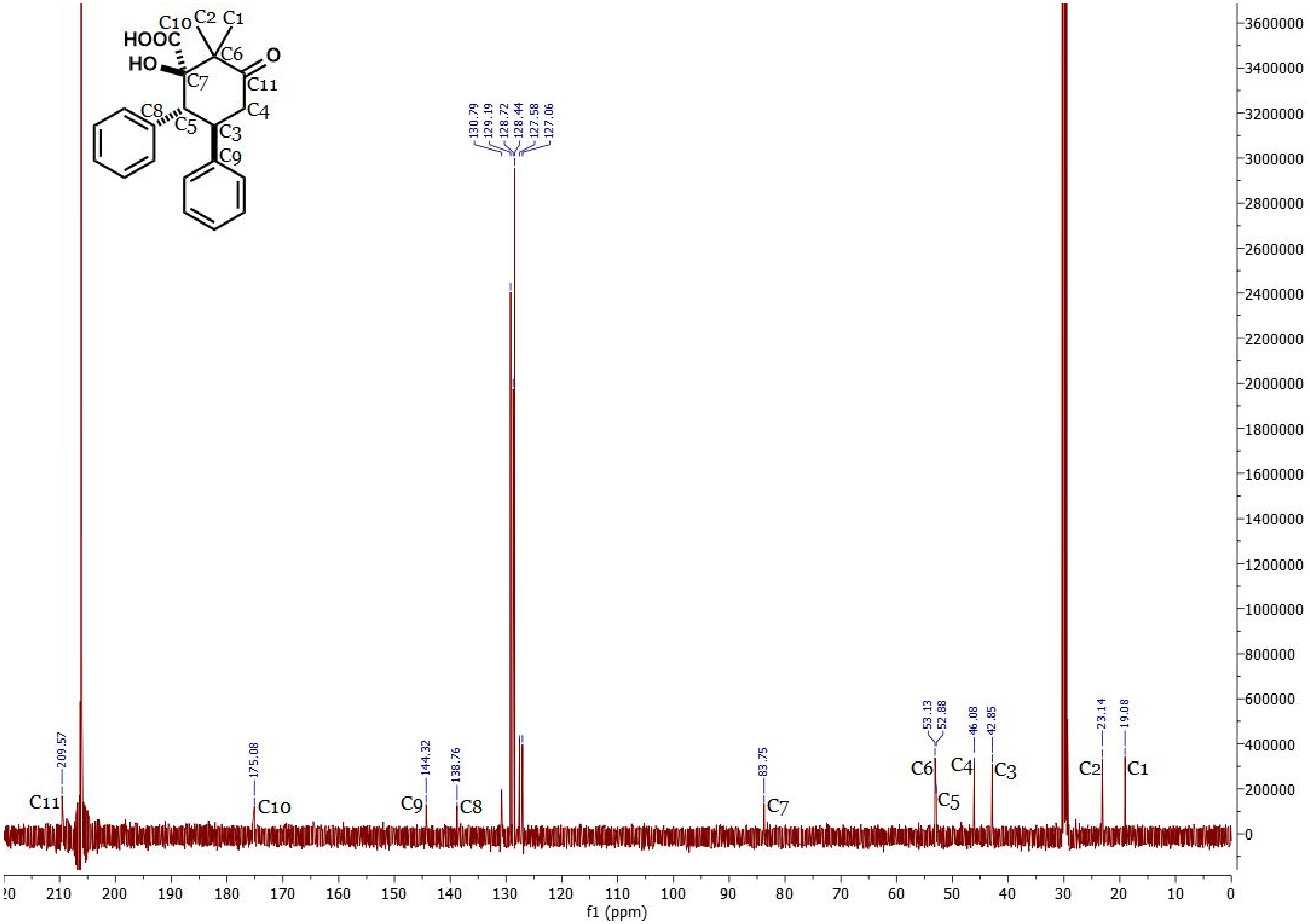

Figure S179: $^{13}C\{^{1}H\}$ NMR spectrum of ***15a*** *(125 MHz, acetone-d6): δ 209.57, 175.08, 144.32, 138.76, 130.79, 129.19, 128.72, 128.44, 127.58, 127.06, 83.75, 53.13, 52.88, 46.08, 42.85, 23.14, 19.08.*

2D NMR of **15a**

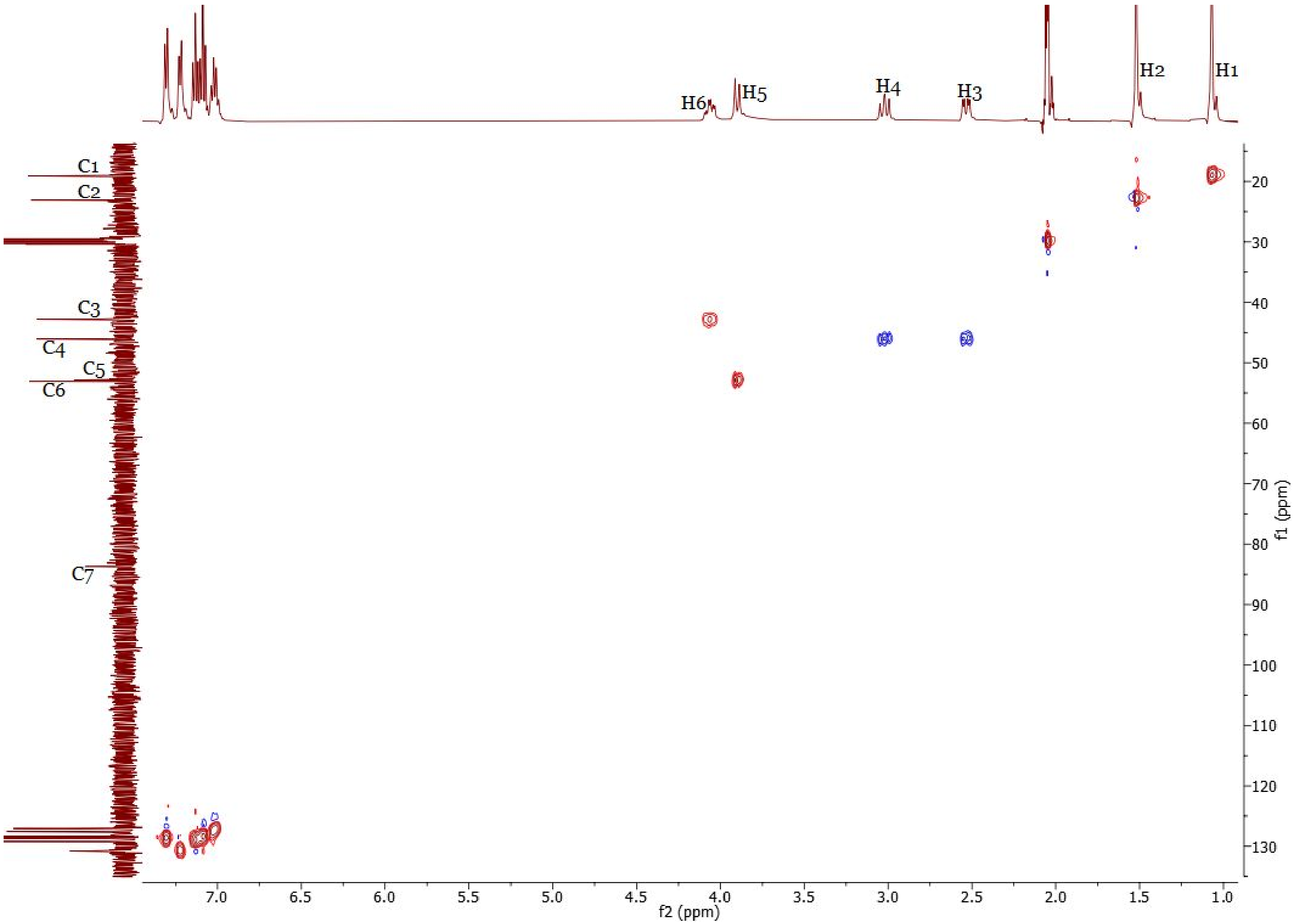

*Figure S180: HSQC spectrum of **15a**.*

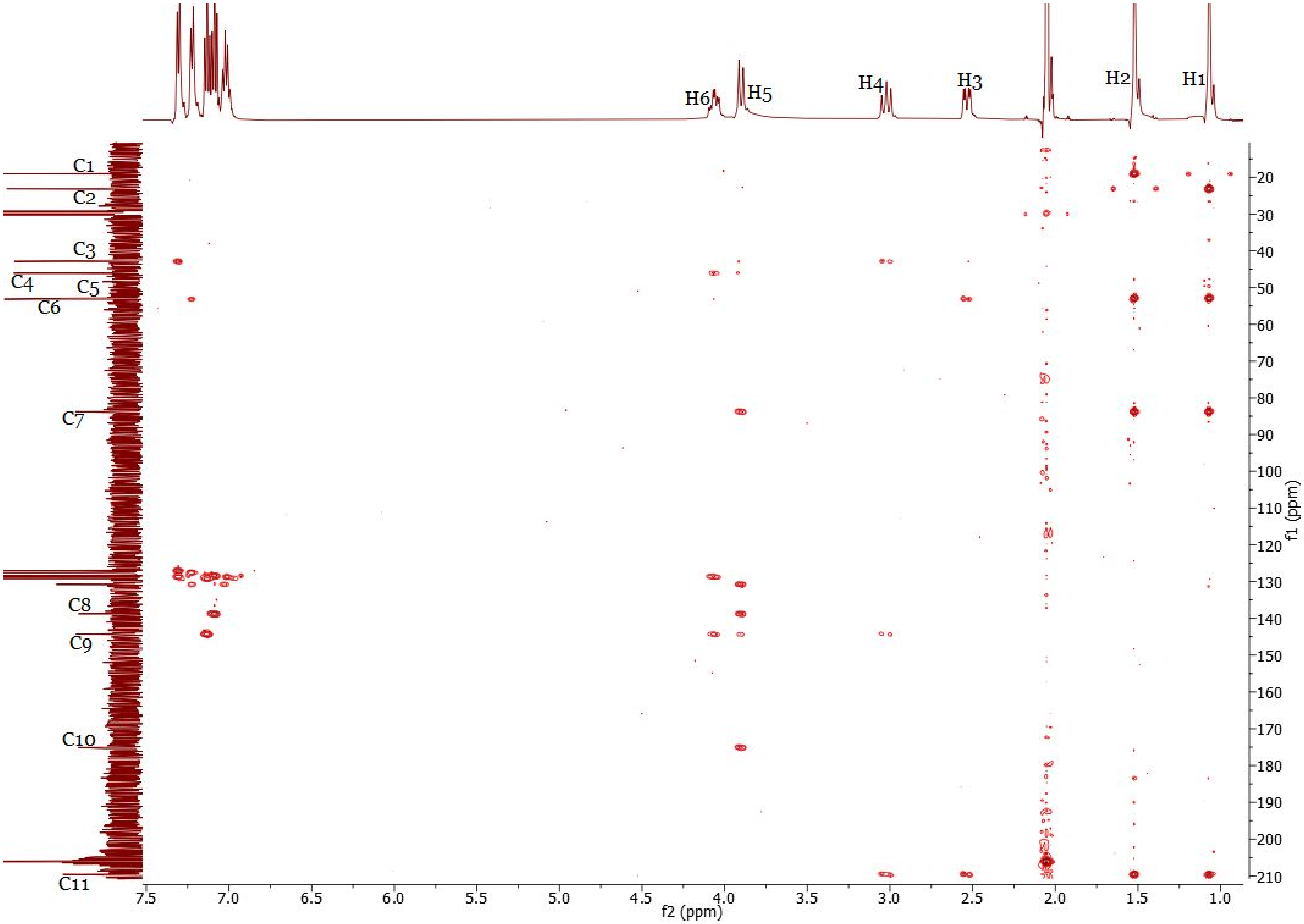

*Figure S181: HMBC spectrum of **15a**.*

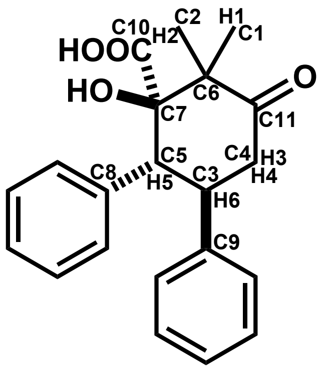

*Figure S182: 2D NMR observations of **15a**.*

2D NMR observations of **15a**:

Protons H1 are attached to carbon C1 forming CH_3 group. The group has connectivity to carbons C2, C6, C7 and C11.

Protons H2 are attached to carbon C2 forming CH_3 group. The group has connectivity to carbons C1, C6, C7 and C11.

Protons H3 and H4 are attached to carbon C4 forming CH_2 group. The group has connectivity to carbons C3, C5, C9 (weak) and C11.

Proton H5 is attached to carbon C5 forming CH group. The group has connectivity to carbons C4, C7, C8, C9 (weak) and C10. The group also sees inside the phenyl group, suggesting nearby position.

Proton H6 is attached to carbon C3 forming CH group. The group has connectivity to carbons C4, C9 and sees inside the phenyl group, suggesting nearby position.

IR spectroscopy of **15a**

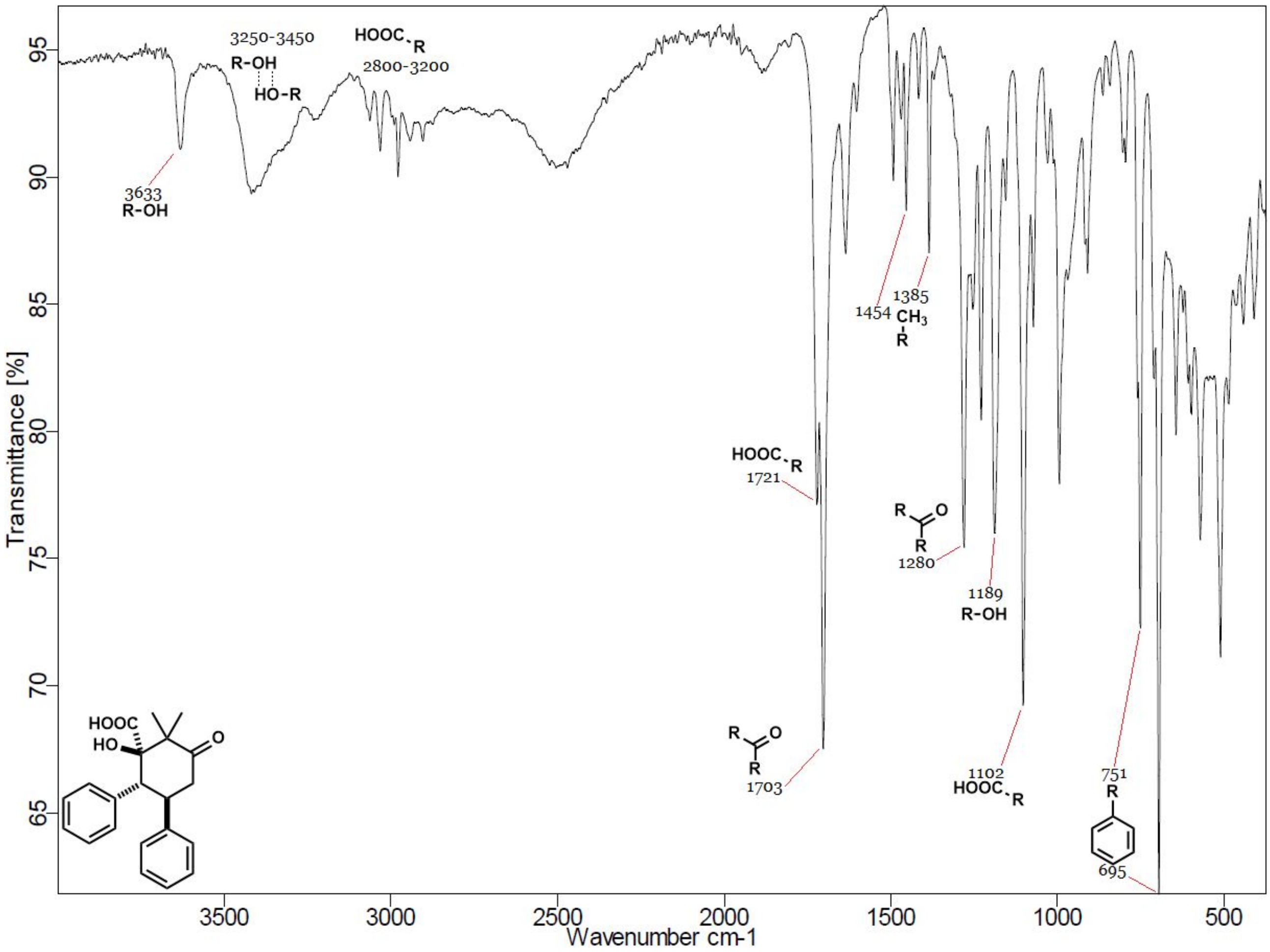

*Figure S183: IR spectrum of **15a** (3633 (m, sharp), 1189 (s) (R-OH) 3250-3450 (m, broad) (intermolecular hydrogen bonds), 2800-3200 (broad), 1721 (s), 1102 (s) (R-COOH), 1703 (s), 1280 (s) (R-**CO**-R), 1454 (m), 1385 (m) (R-CH_3), 751 (s), 695 (s) (5 adjacent H (Ph)) cm^{-1}).*

HRMS of **15a**

HRMS (ESI-TOF) m/z: [**15a**-H]$^-$ calculated for $C_{21}H_{21}O_4$ 337.1434; Found 337.1430; Error 1.327 ppm.

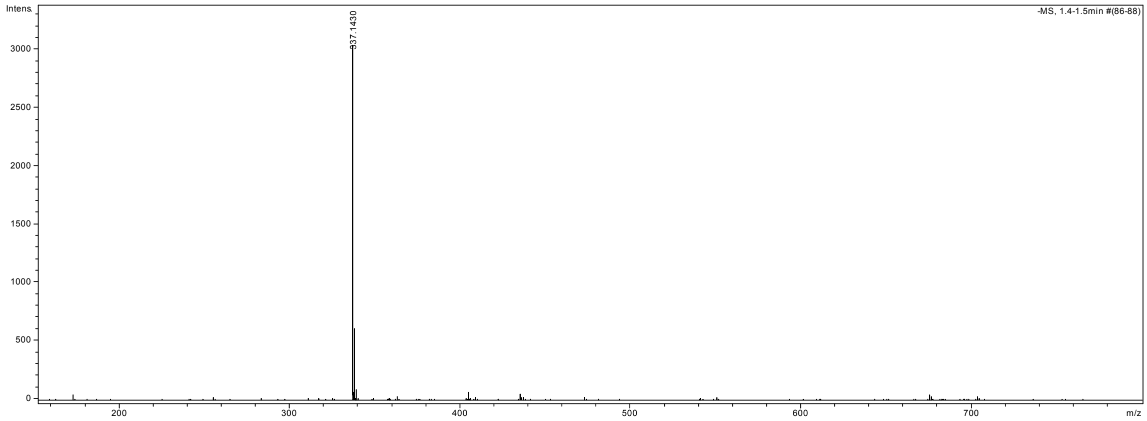

*Figure S184: ESI-TOF-MS of [**15a**-H]$^-$ (peak: 337.1430 m/z, negative-ion mode).*

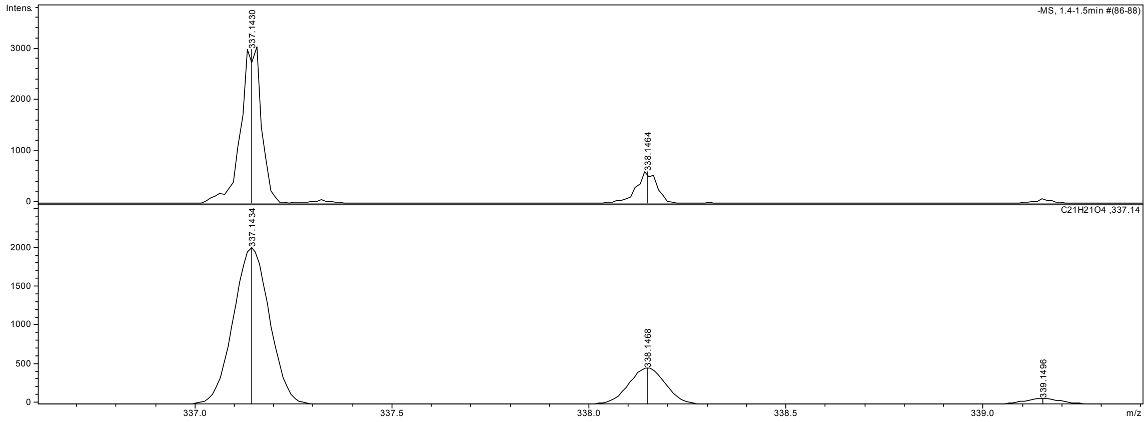

*Figure S185: Measured compound peak of [**15a**-H]$^-$ (337.1430 m/z) at top, simulated peak ($C_{21}H_{21}O_4$) below.*

3.22 Spectroscopic data of 15b

1D NMR of **15b**

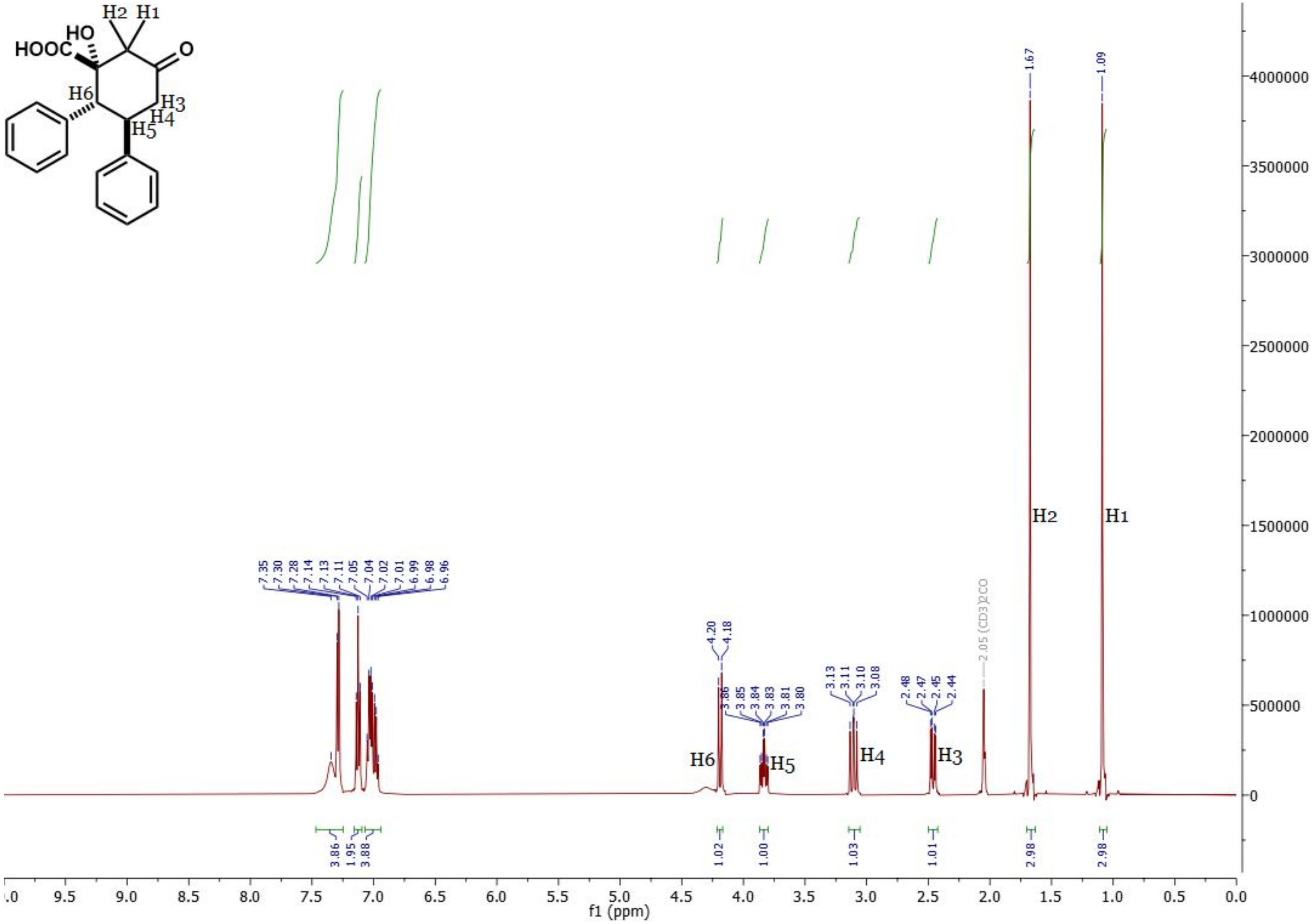

*Figure S186: ^{1}H NMR spectrum of **15b** (500 MHz, acetone-d6): δ 7.48-7.25 (m, 4H), 7.13 (t, J = 7.8 Hz, 2H), 7.07-6.94 (m, 4H), 4.19 (d, J = 12.3 Hz, 1H), 3.83 (td, J = 12.6, 5.3 Hz, 1H), 3.11 (dd, J = 14.9, 12.8 Hz, 1H), 2.46 (dd, J = 14.9, 5.3 Hz, 1H), 1.67 (s, 3H), 1.09 (s, 3H).*

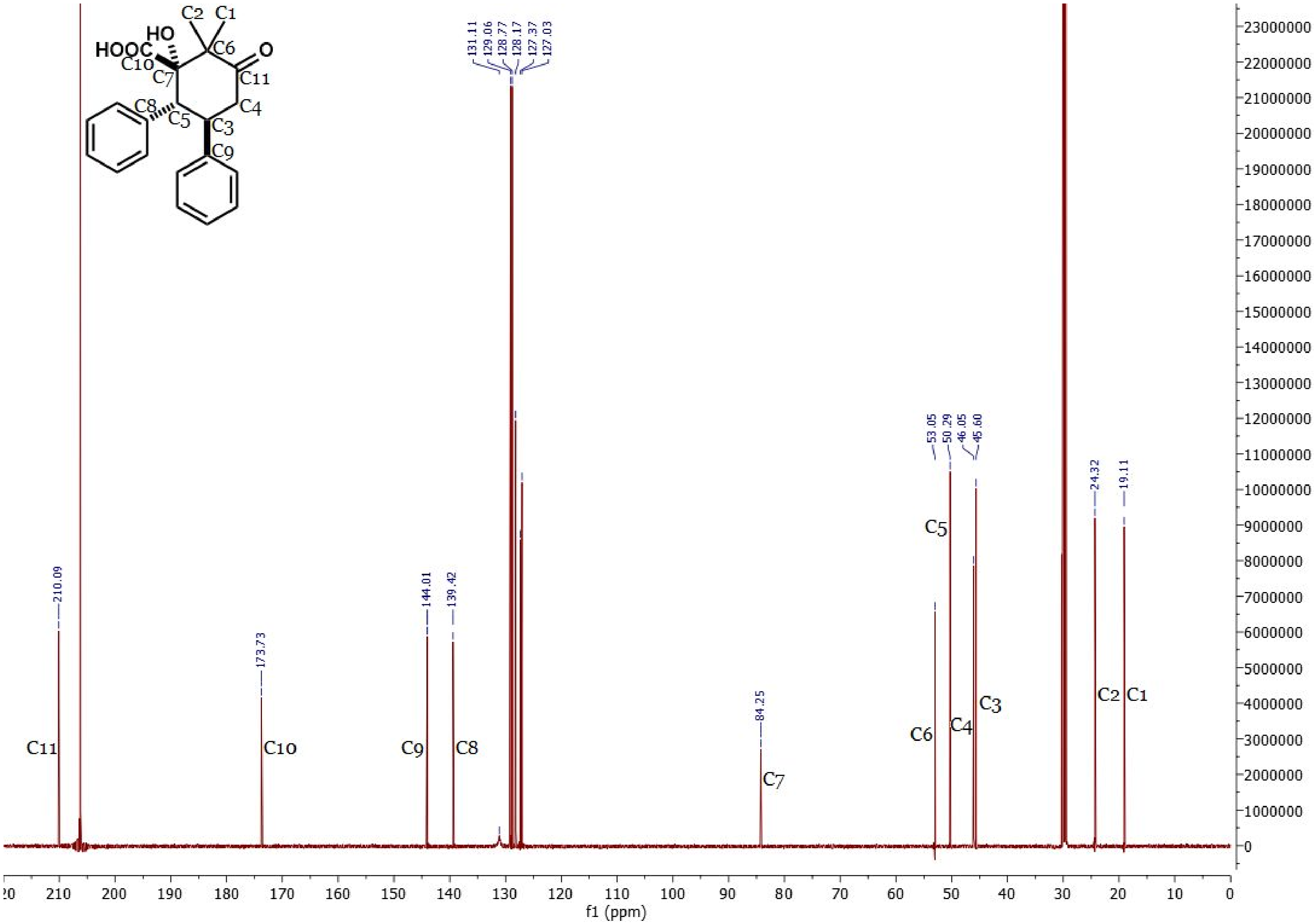

*Figure S187: $^{13}C\{^1H\}$ NMR spectrum of **15b** (125 MHz, acetone-d6): δ 210.09, 173.73, 144.01, 139.42, 131.11, 129.06, 128.77, 128.17, 127.37, 127.03, 84.25, 53.05, 50.29, 46.05, 45.60, 24.32, 19.11.*

2D NMR of **15b**

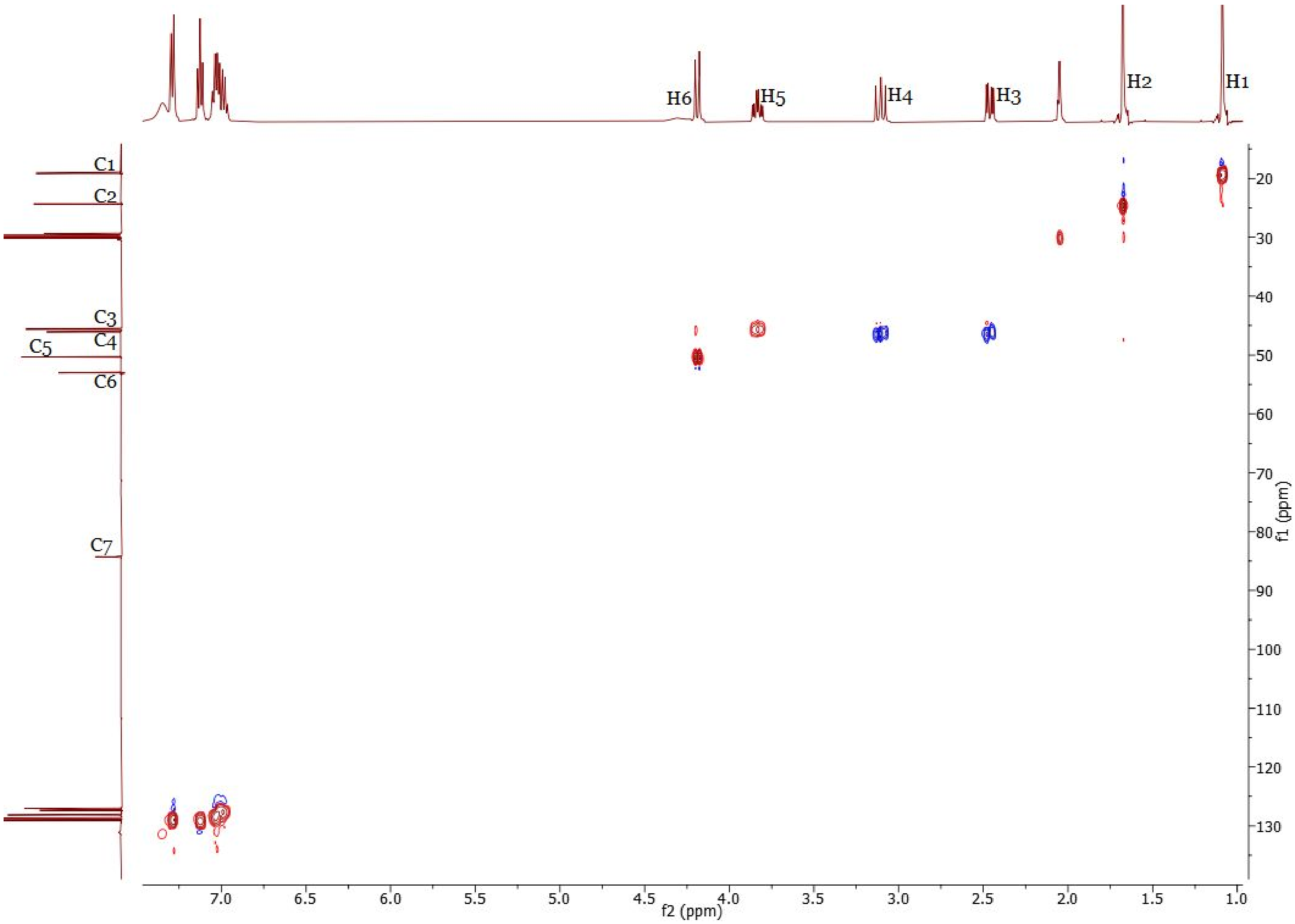

*Figure S188: HSQC spectrum of **15b**.*

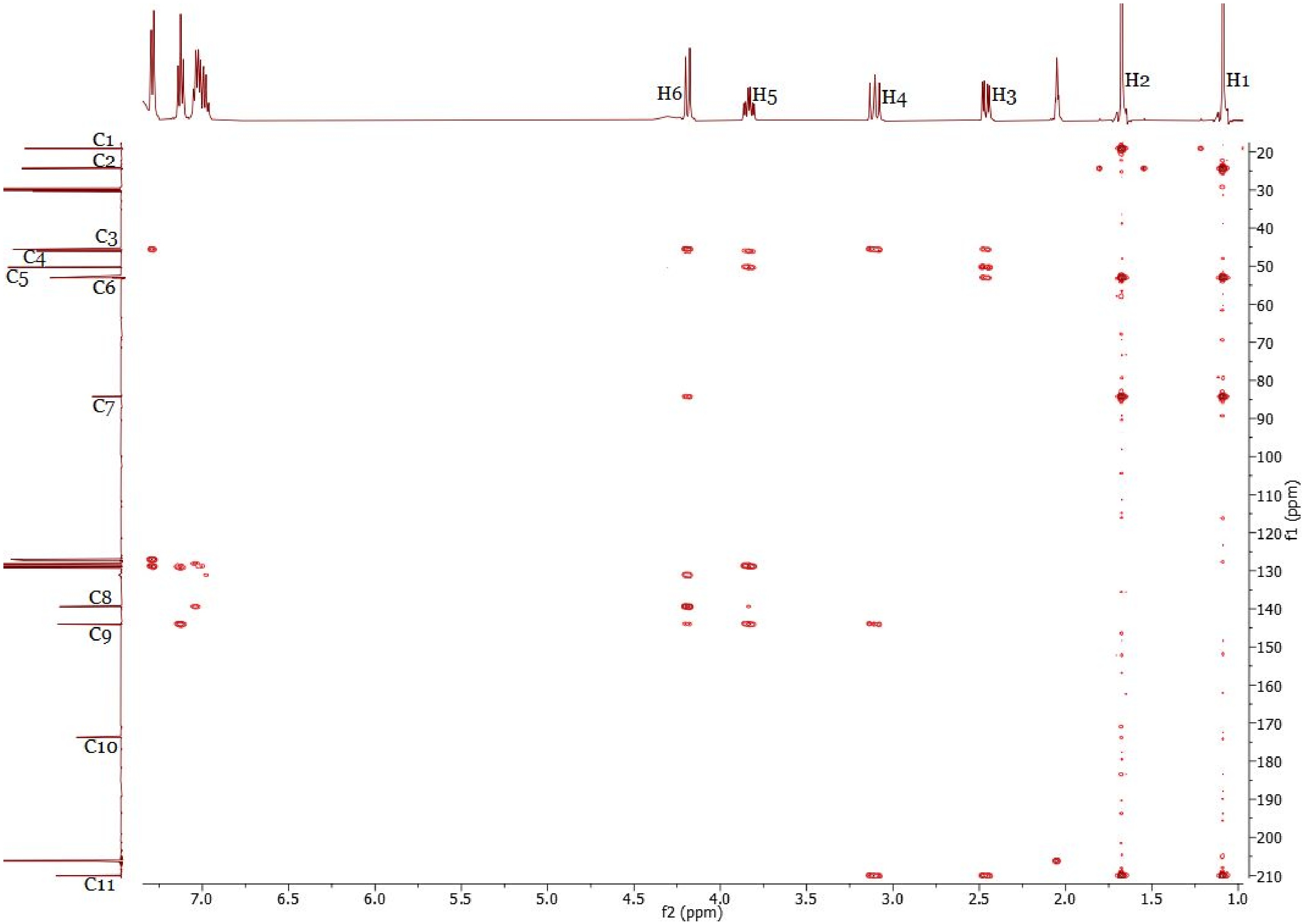

*Figure S189: HSQC spectrum of **15b**.*

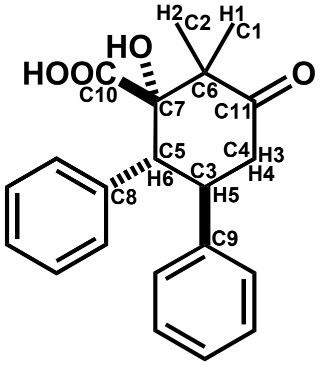

*Figure S190: 2D NMR observations of **15b**.*

2D NMR observations of **15b**:

Protons H1 are attached to carbon C1 forming CH_3 group. The group has connectivity to carbons C2, C6, C7 and C11.

Protons H2 are attached to carbon C2 forming CH_3 group. The group has connectivity to carbons C1, C6, C7 and C11.

Protons H3 and H4 are attached to carbon C4 forming CH_2 group. The group has connectivity to carbons C3, C5, C6, C9 (weak) and C11.

Proton H5 is attached to carbon C3 forming CH group. The group has connectivity to carbons C4, C5, C9 and sees inside the phenyl group, suggesting nearby position.

Proton H6 is attached to carbon C5 forming CH group. The group has connectivity to carbons C3, C7, C8, C9 (weak) and sees inside the phenyl group, suggesting nearby position.

IR spectroscopy of **15b**

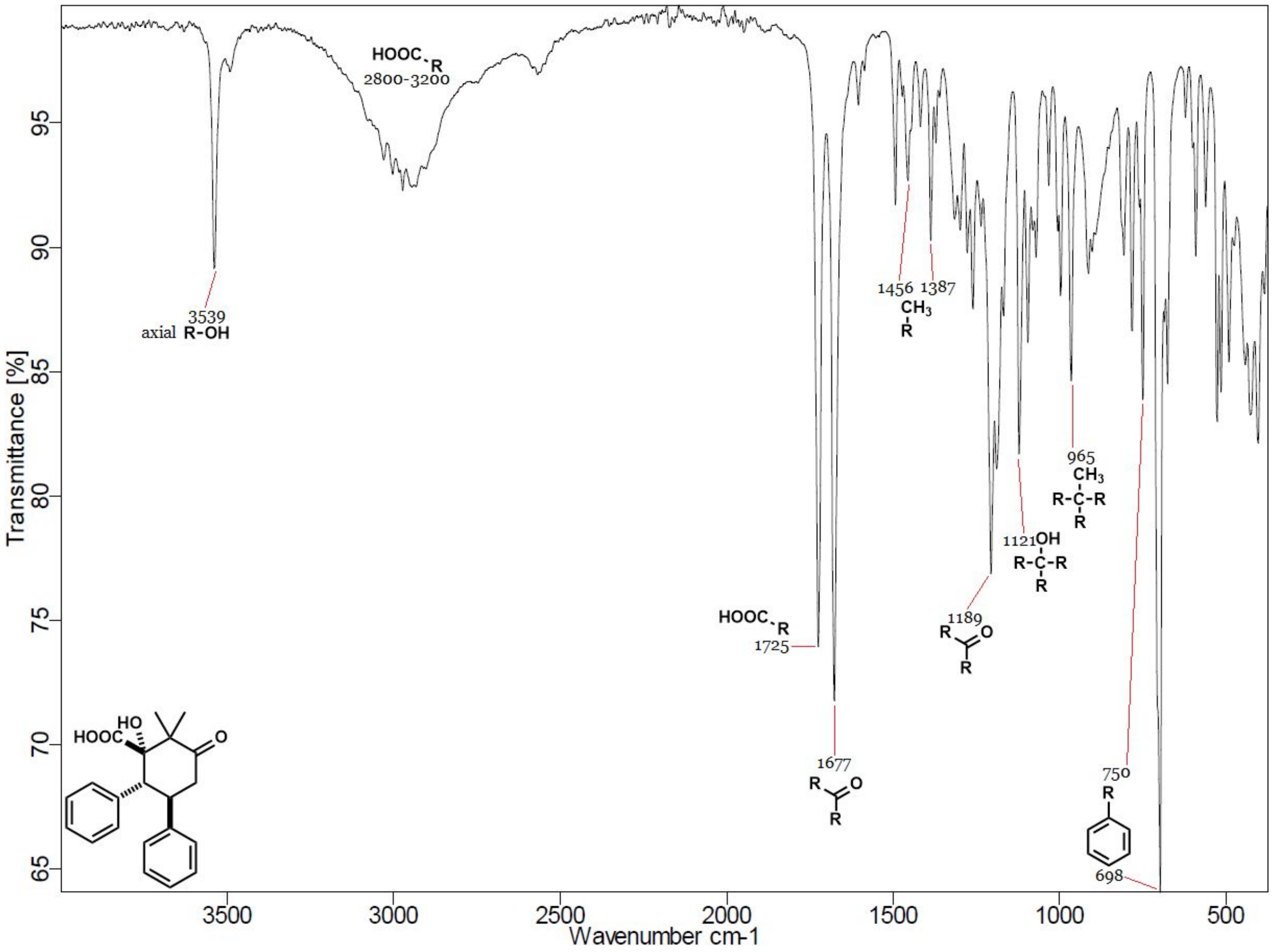

*Figure S191: IR spectrum of **15b** (3539 (m, sharp) (Axial -OH), 2800-3200 (broad), 1725 (s) (R-COOH), 1677 (s), 1189 (s) (R-**CO**-R), 1456 (w), 1387 (w), 965 (m) (R-CH_3), 1121 (m) (tert-OH), 750 (m), 698 (s) (5 adjacent H (Ph)) cm^{-1}).*

HRMS of **15b**

HRMS (ESI-TOF) m/z: [**15b**-H]- calculated for $C_{21}H_{21}O_4$ 337.1434; Found 337.1431; Error 0.982 ppm.

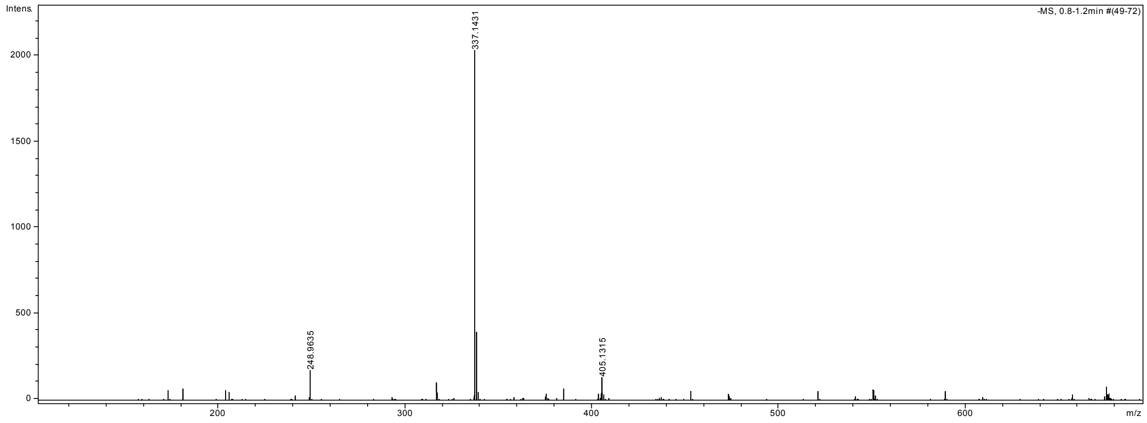

*Figure S192: ESI-TOF-MS of [**15b**-H]- (peak: 337.1431 m/z, negative-ion mode).*

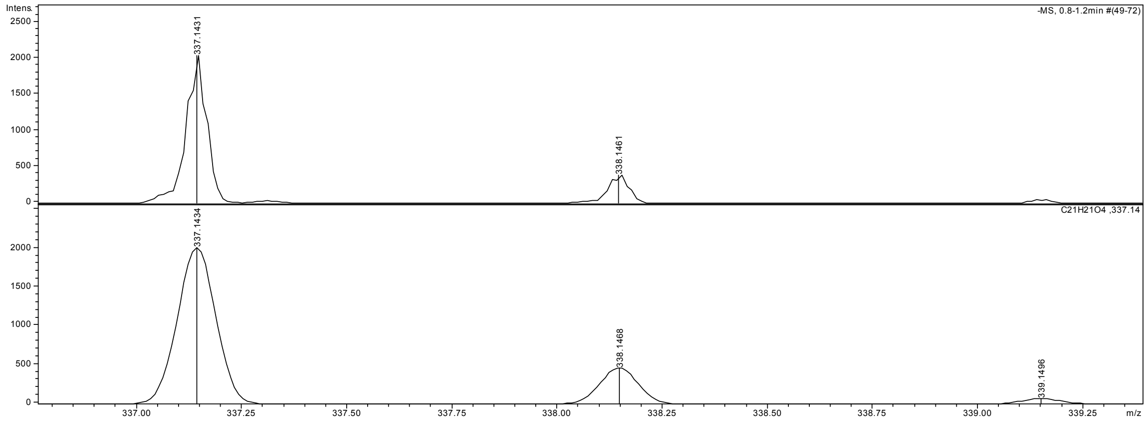

*Figure S193: Measured compound peak of [**15b**-H]- (337.1431 m/z) at top, simulated peak ($C_{21}H_{21}O_4$) below.*

3.23 Spectroscopic data of 16a

1D NMR of **16a**

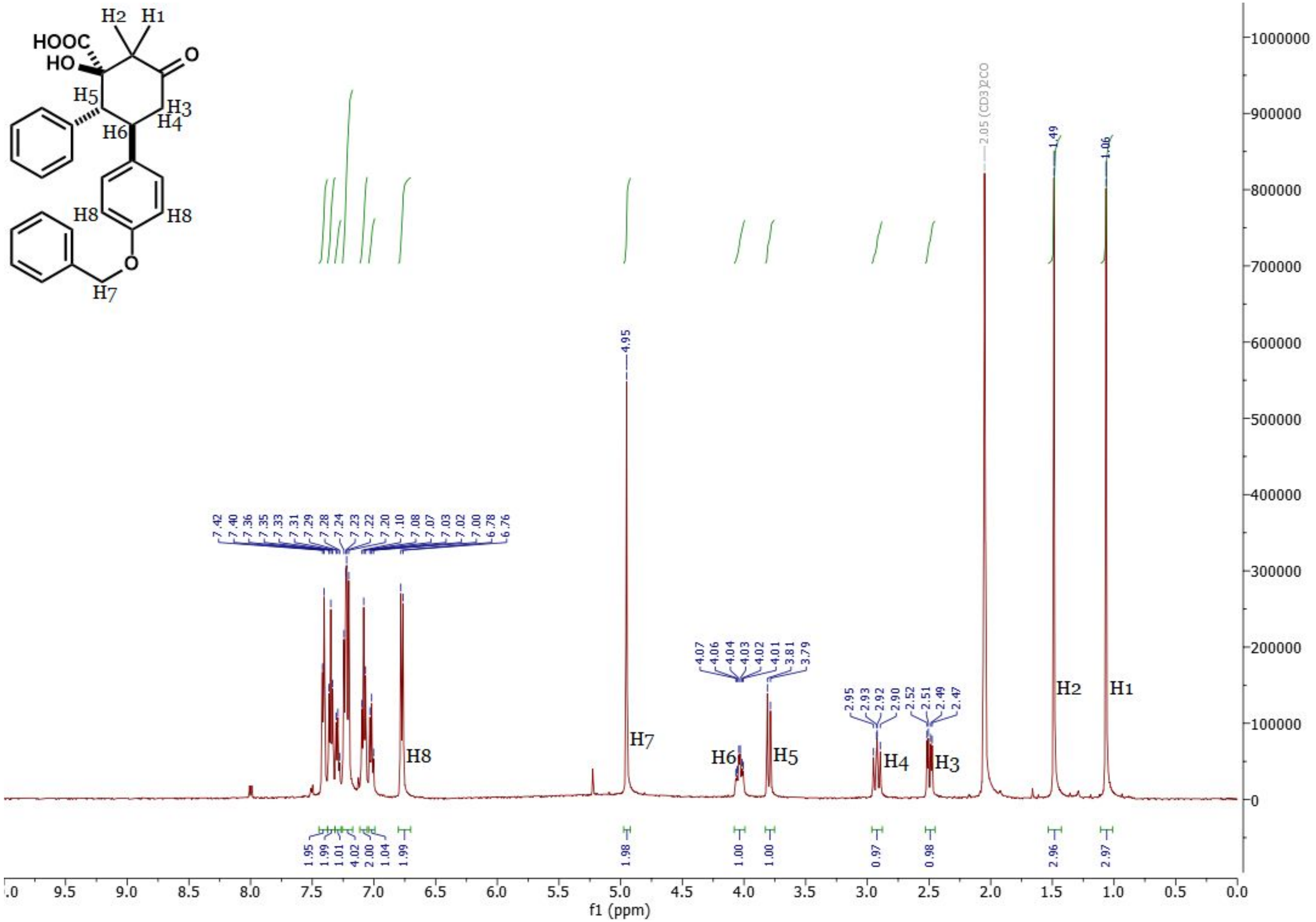

*Figure S194: ¹H NMR spectrum of **16a** (500 MHz, acetone-d6): δ 7.45-7.38 (m, 2H), 7.38-7.32 (m, 2H), 7.32-7.27 (m, 1H), 7.27-7.16 (m, 4H), 7.12-7.05 (m, 2H), 7.05-7.00 (m, 1H), 6.77 (d, J = 8.7 Hz, 2H), 4.95 (s, 2H), 4.04 (td, J = 12.3, 5.4 Hz, 1H), 3.80 (d, J =12.8 Hz, 1H), 2.92 (dd, J = 15.1, 12.4 Hz, 1H), 2.50 (dd, J = 15.2, 5.5 Hz, 1H), 1.49 (s, 3H), 1.06 (s, 3H).*

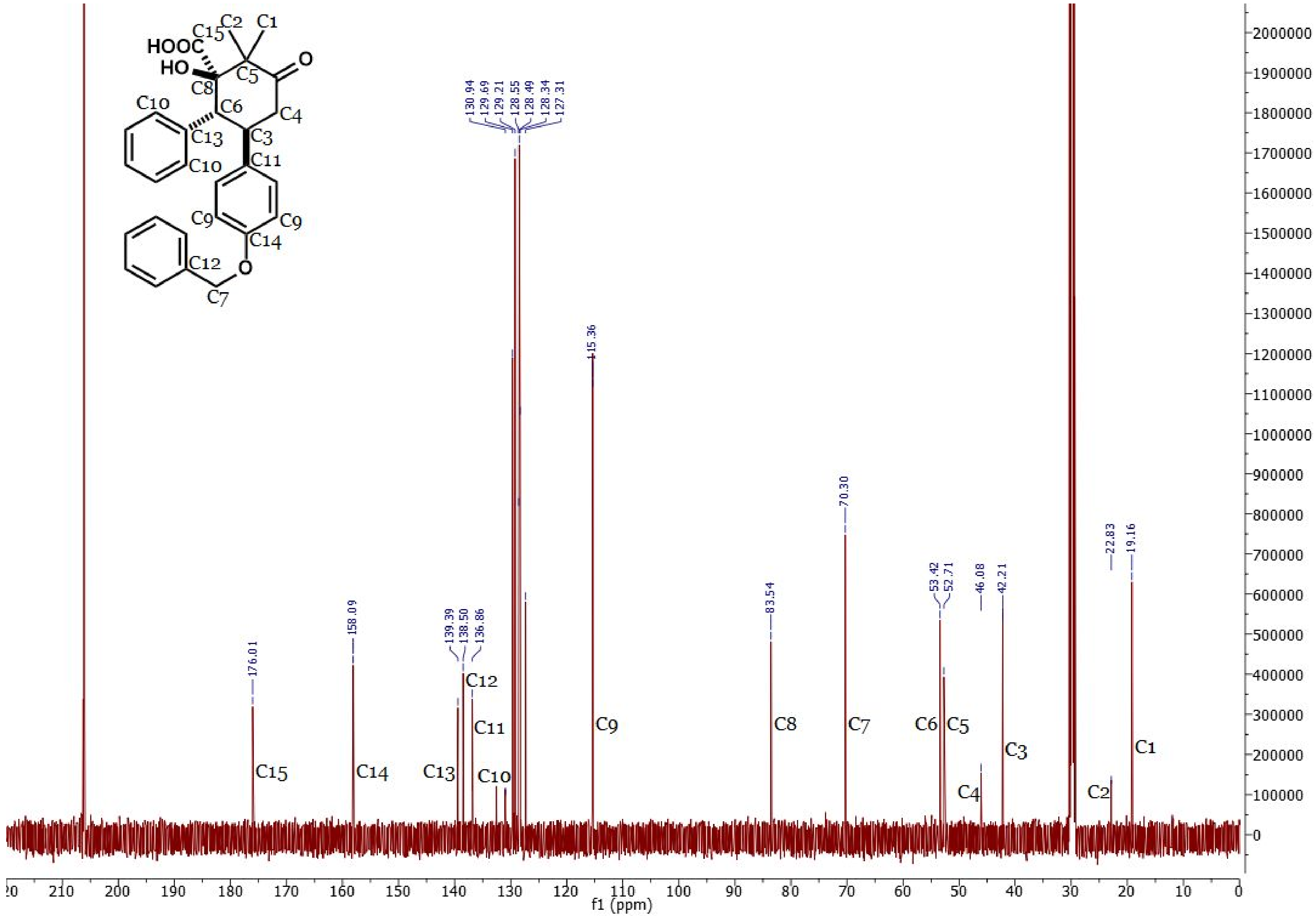

*Figure S195: $^{13}C\{^1H\}$ NMR spectrum of **16a** (125 MHz, acetone-d6): δ 176.01, 158.09, 139.39, 138.50, 136.86, 130.94, 129.69, 129.21, 128.55, 128.49, 128.34, 127.31, 115.36, 83.54, 70.30, 53.42, 52.71, 46.08, 42.21, 22.83, 19.16. Note: Ketone signal is not observed. Even with extended spectral window or increased relaxation time.*

2D NMR of **16a**

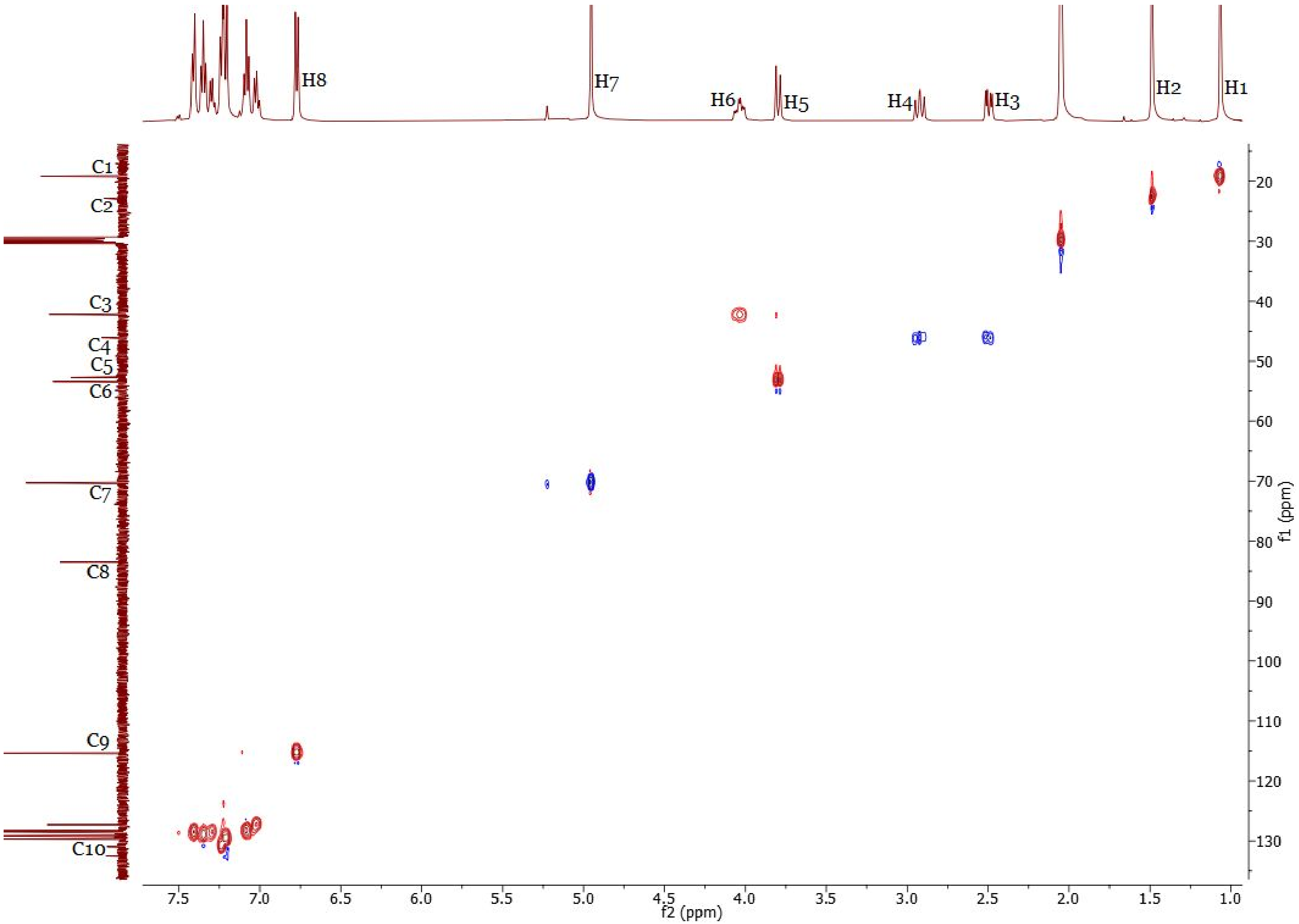

*Figure S196: HSQC spectrum of **16a**.*

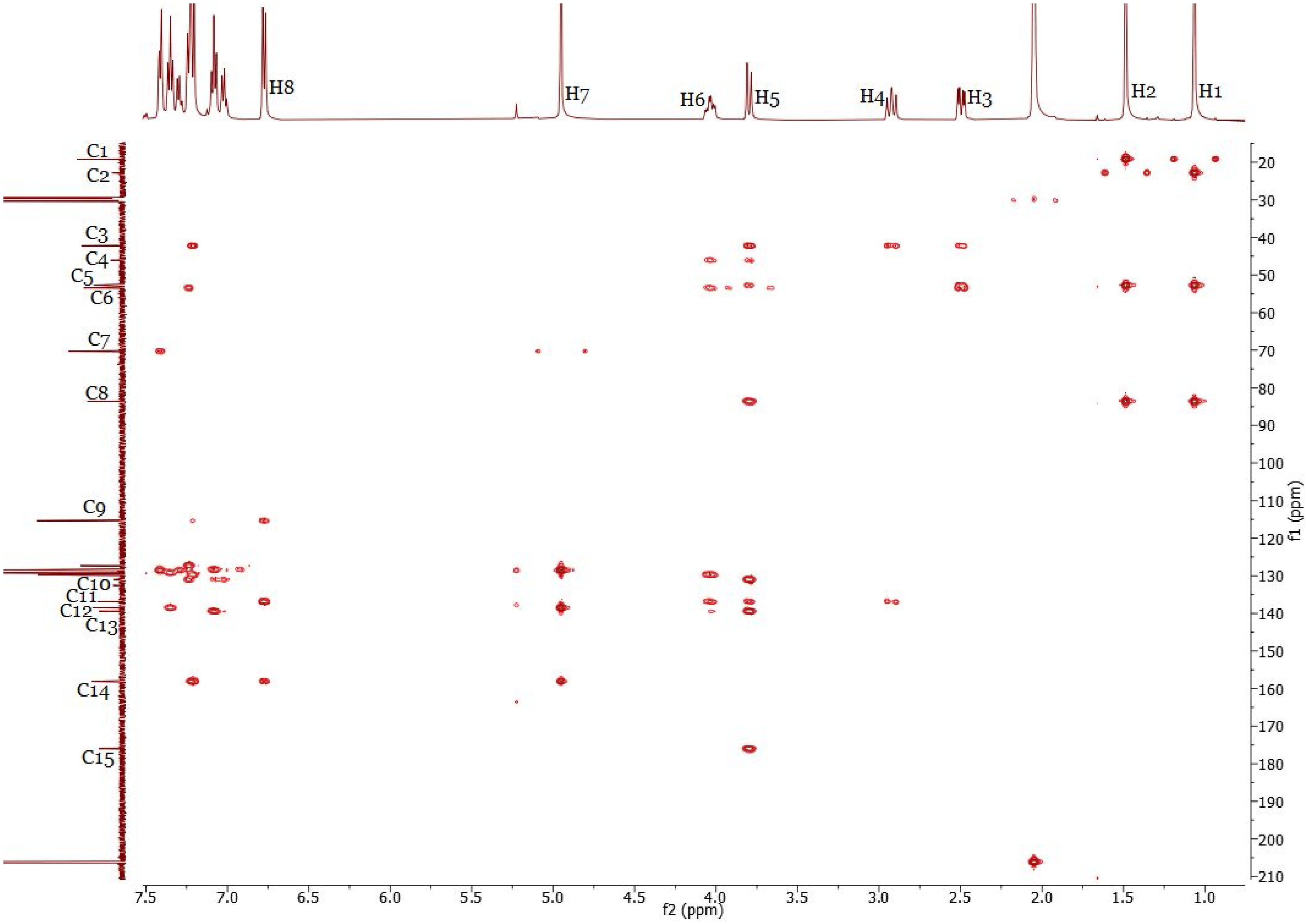

*Figure S197: HMBC spectrum of **16a**.*

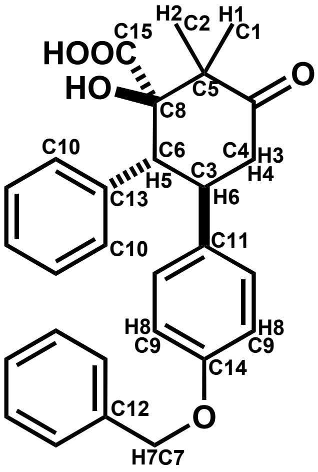

*Figure S198: 2D NMR Observations of **16b***

2D NMR observations of **16b**:

Protons H1 are attached to carbon C1 forming CH_3 group. The group has connectivity to carbons C2, C5 and C8.

Protons H2 are attached to carbon C2 forming CH_3 group. The group has connectivity to carbons C1, C5 and C8.

Protons H3 and H4 are attached to carbon C4 forming CH_2 group. The group has connectivity carbons C3, C6 and C11 (weak).

Proton H5 is attached to carbon C6 forming CH group. The group has connectivity to carbons C4, C3, C5, C8, C10, C11, C13 and C15.

Proton H6 is attached to carbon C3 forming CH group. The group has connectivity to carbons C4, C6, C11 and C13 (weak). The group has connectivity inside a phenyl group, suggesting nearby location.

Protons H7 are attached to carbon C7 forming CH_2 group. The group has connectivity to carbons C12 and C14. The group has connectivity inside a phenyl group, suggesting nearby location.

Protons H8 are attached to carbons C9 forming two CH groups. The groups have connectivity to carbons C9 (itself), C11 and C14.

note: Ketone group is not observed, even with extended spectral window or increased relaxation time.

IR spectroscopy of **16a**

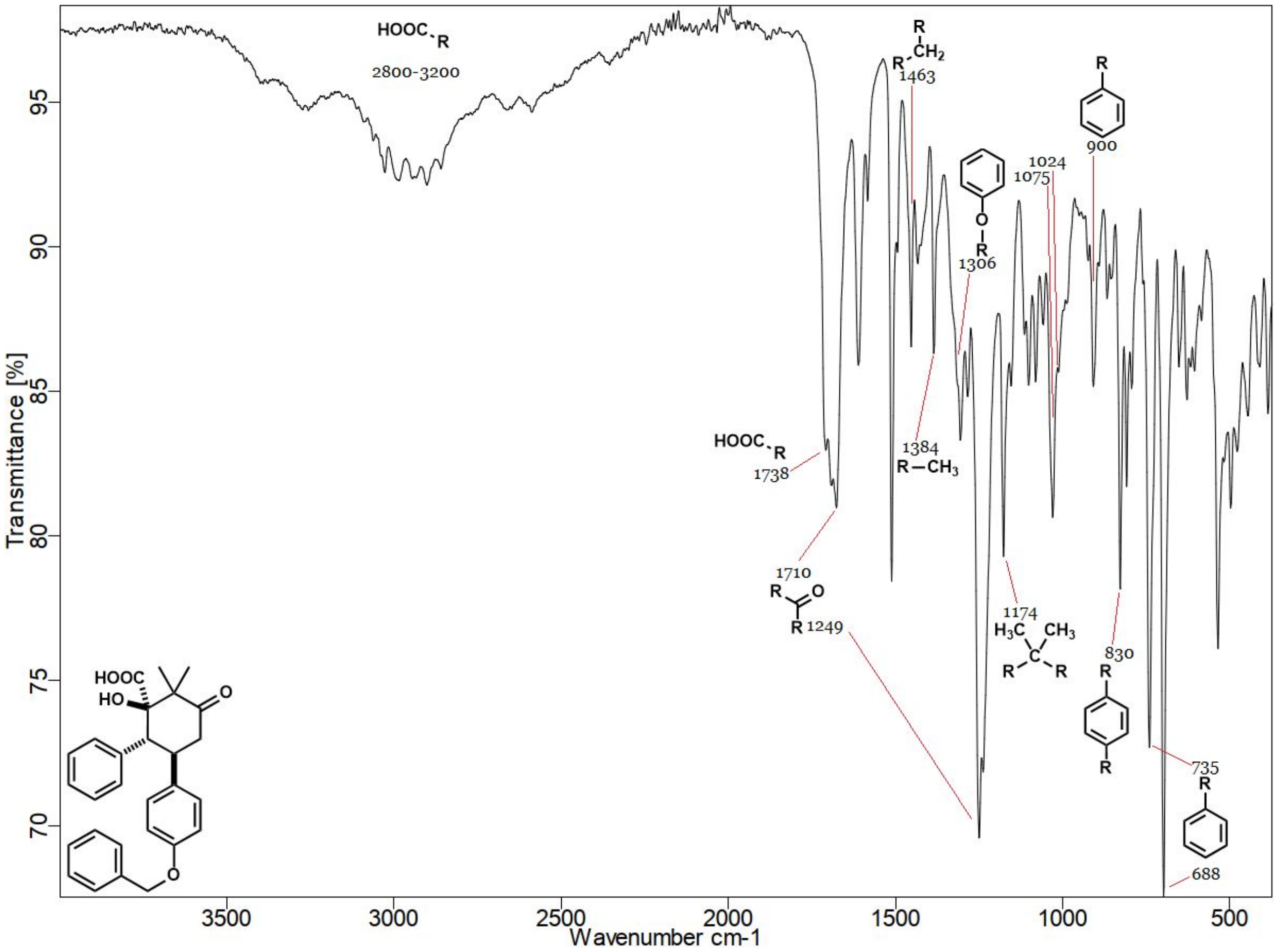

*Figure S199: IR spectrum of **16a** (2800-3200 (broad), 1738 (s) (R-COOH), 1710 (s), 1249 (s) (R-**CO**-R), 1463 (m) (R-CH_2-R), 1384 (m) (R-CH_3), 1306 (m), 1075 (s), 1024 (w) (Ph-O-R), 1174 (m) ((CH_3)$_2$-C-R_2), 900 (m), 735 (s), 688 (s) (5 adjacent H (Ph)), 830 (m) (2 adjacent H (R-Ph-R)) cm^{-1}).*

HRMS of **16a**

HRMS (ESI-TOF) m/z: [**16a**-H]$^-$ calculated for $C_{28}H_{27}O_5$ 443.1853; Found 443.1849; Error 0.992 ppm.

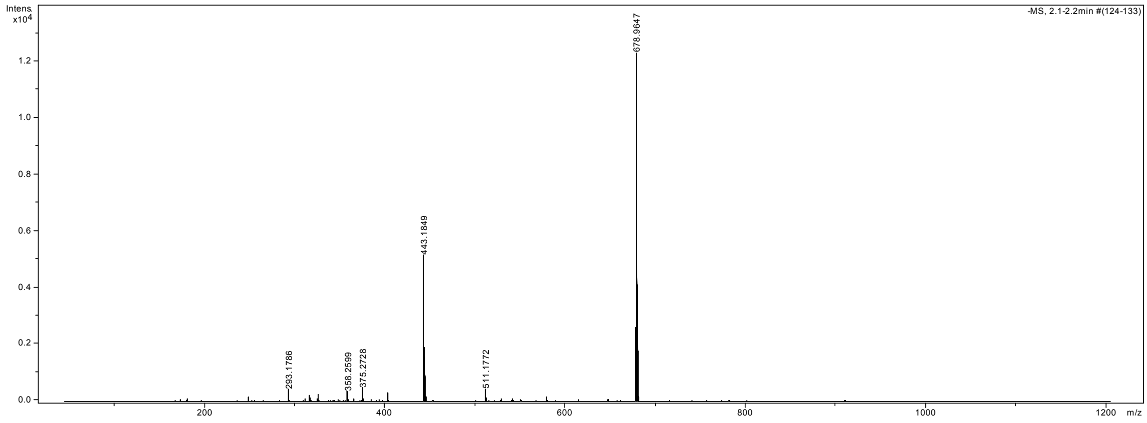

*Figure S200: ESI-TOF-MS of [**16a**-H]$^-$ (peak: 443.1849 m/z, negative-ion mode).*

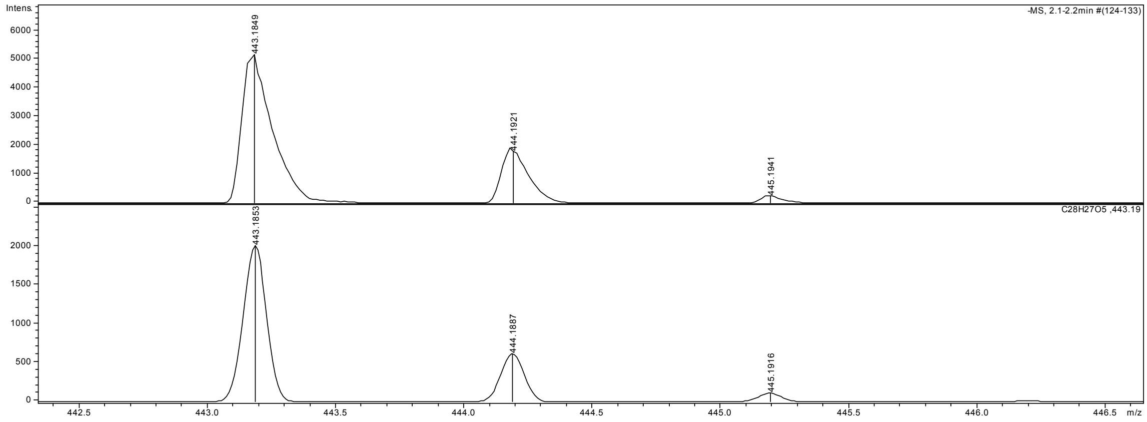

*Figure S201: Measured compound peak of [**16a**-H]$^-$ (443.1849 m/z) at top, simulated peak ($C_{28}H_{27}O_5$) below.*

3.24 Spectroscopic data of 16b

1D NMR of **16b**

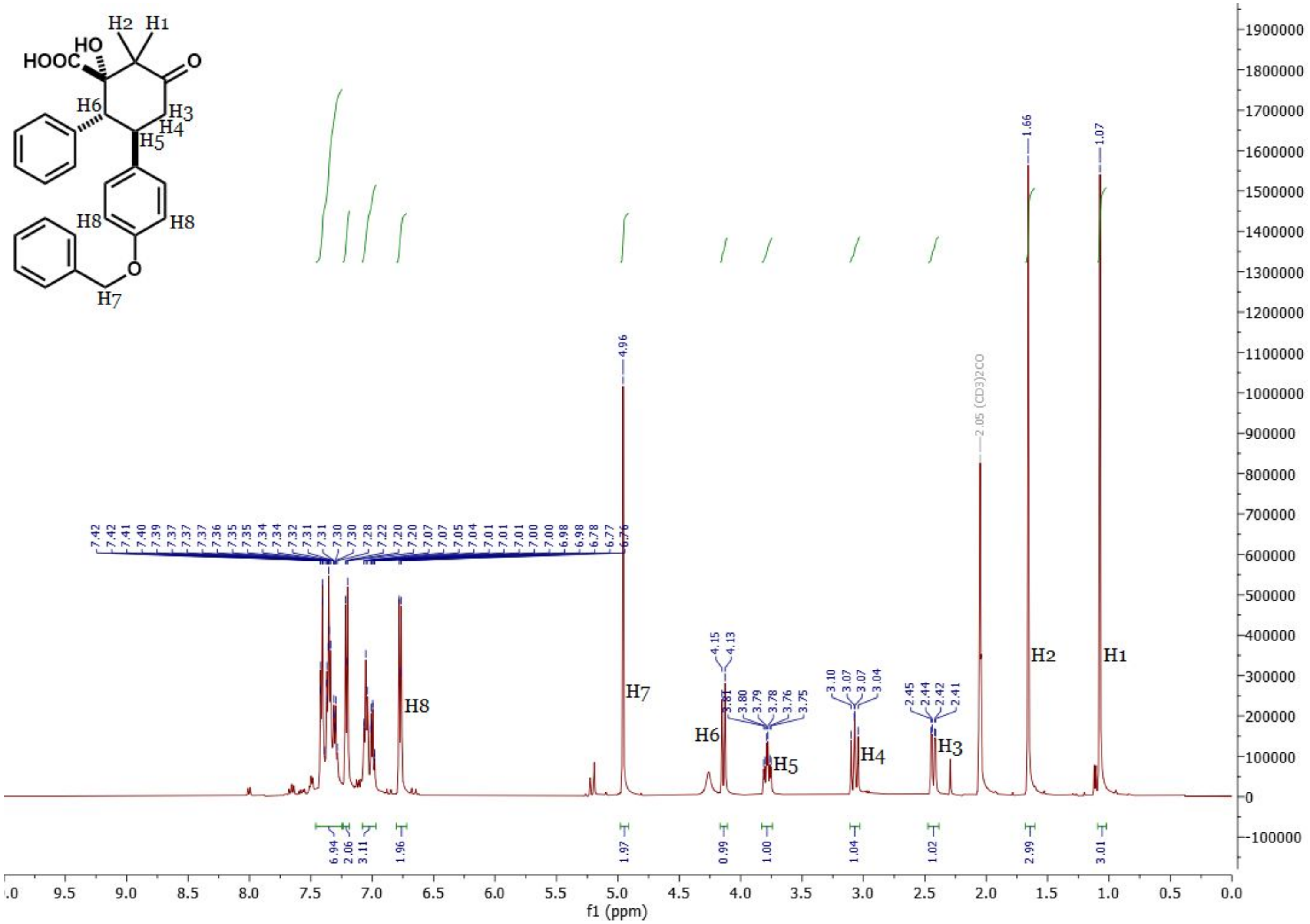

Figure S202: ¹H NMR spectrum of ***16b*** *(500 MHz, acetone-d6): δ 7.43-7.26 (m, 7H), 7.21 (m, 2H), 7.08-6.95 (m, 3H), 6.77 (m, 2H), 4.96 (s, 2H), 4.14 (d, J = 12.2 Hz, 1H), 3.78 (td, J = 12.5, 5.2 Hz), 3.07 (dd, J = 14.9, 12.8 Hz, 1H), 2.43 (dd, J = 14.9, 5.2 Hz, 1H), 1.66 (s, 3H), 1.07 (s, 3H).*

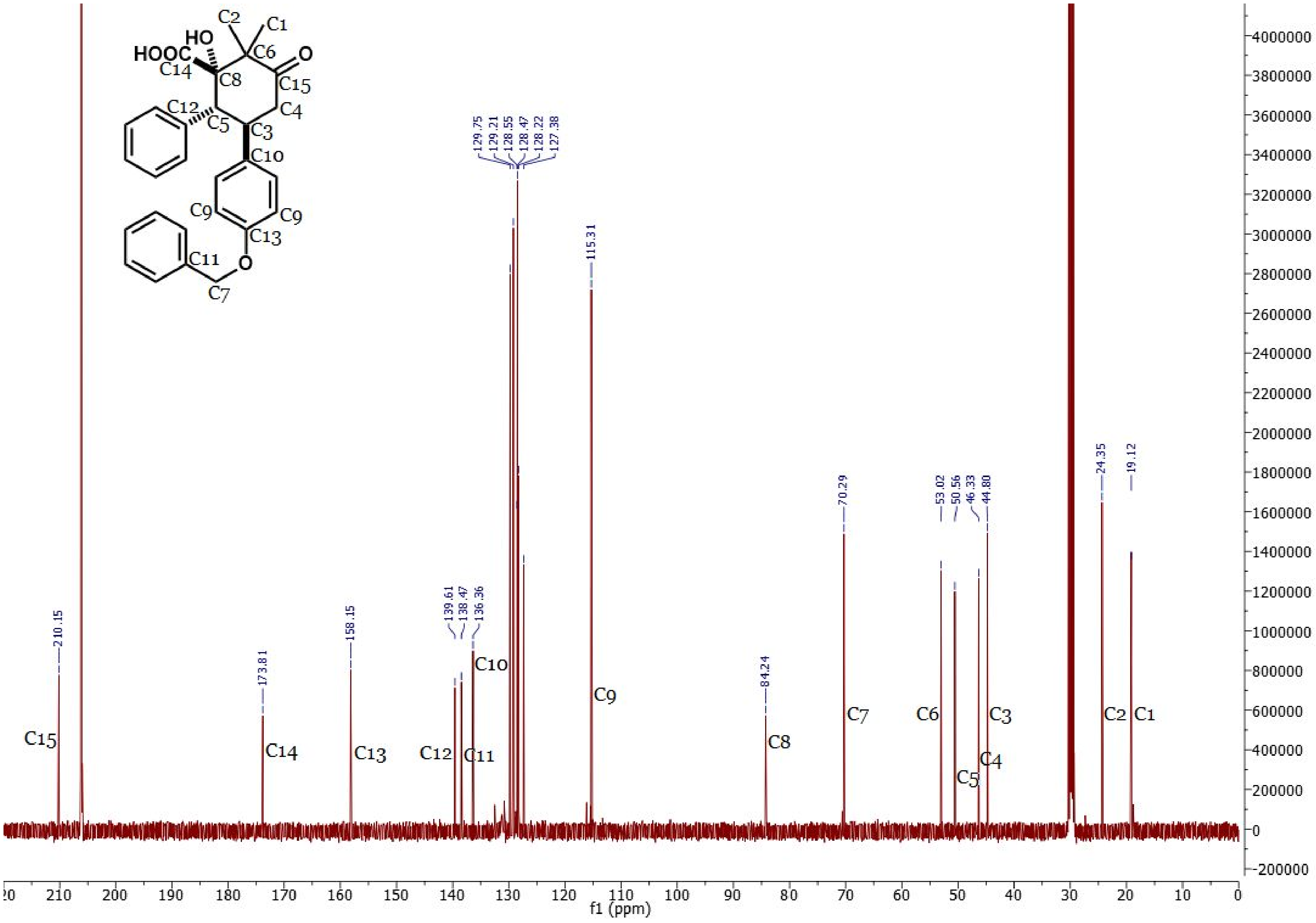

*Figure S203: $^{13}C\{^1H\}$ NMR spectrum of **16b** (125 MHz, acetone-d6): δ 210.15, 173.81, 158.15, 139.61, 138.47, 136.36, 129.75, 129.21, 128.55, 128.47, 128.22, 127.38, 115.31, 84.24, 70.29, 53.02, 50.56, 46.33, 44.80, 24.35, 19.12. Note: one signal is missing, probably due overlapping at aromatic region.*

2D NMR of **16b**

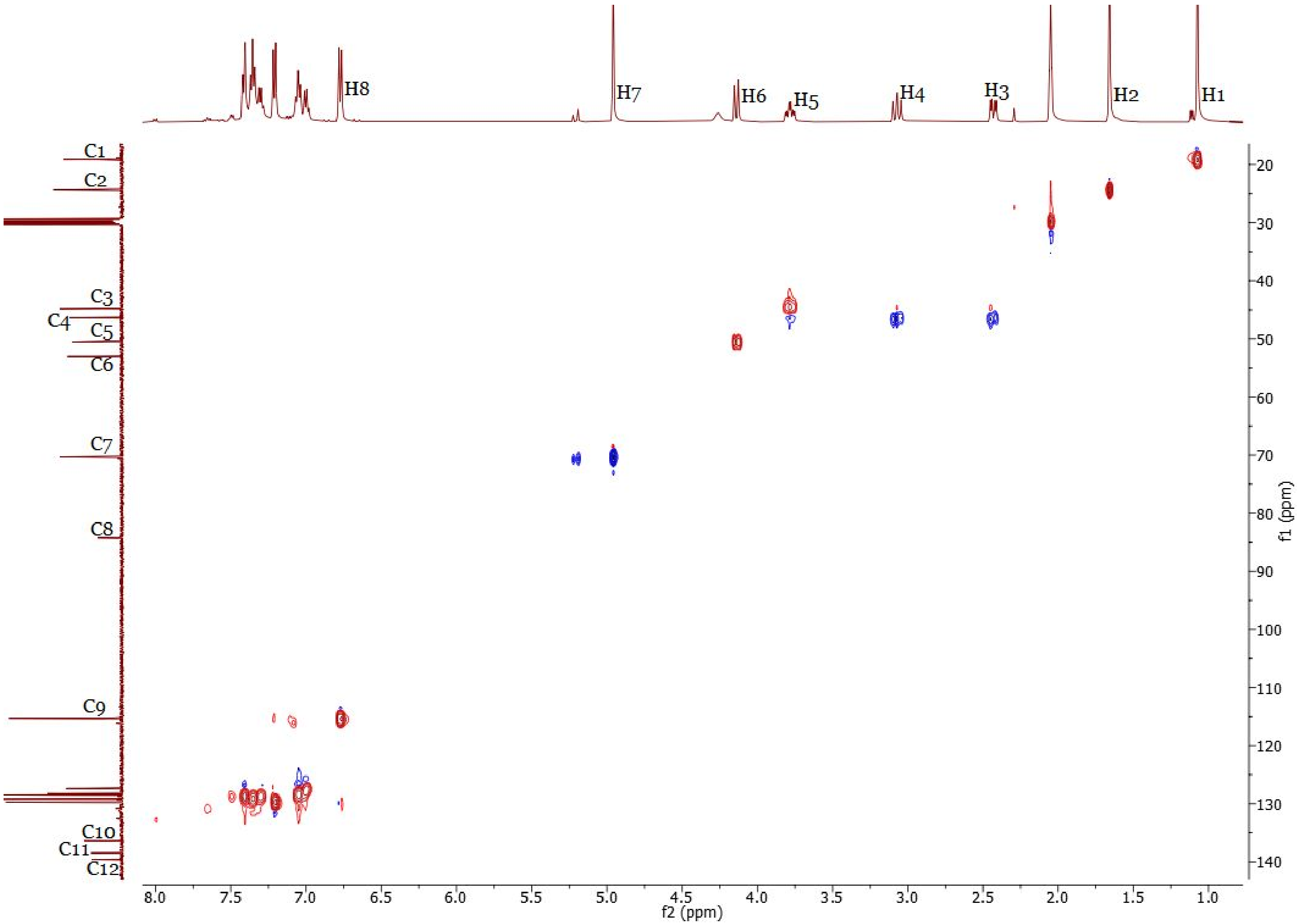

*Figure S204: HSQC spectrum of **16b**.*

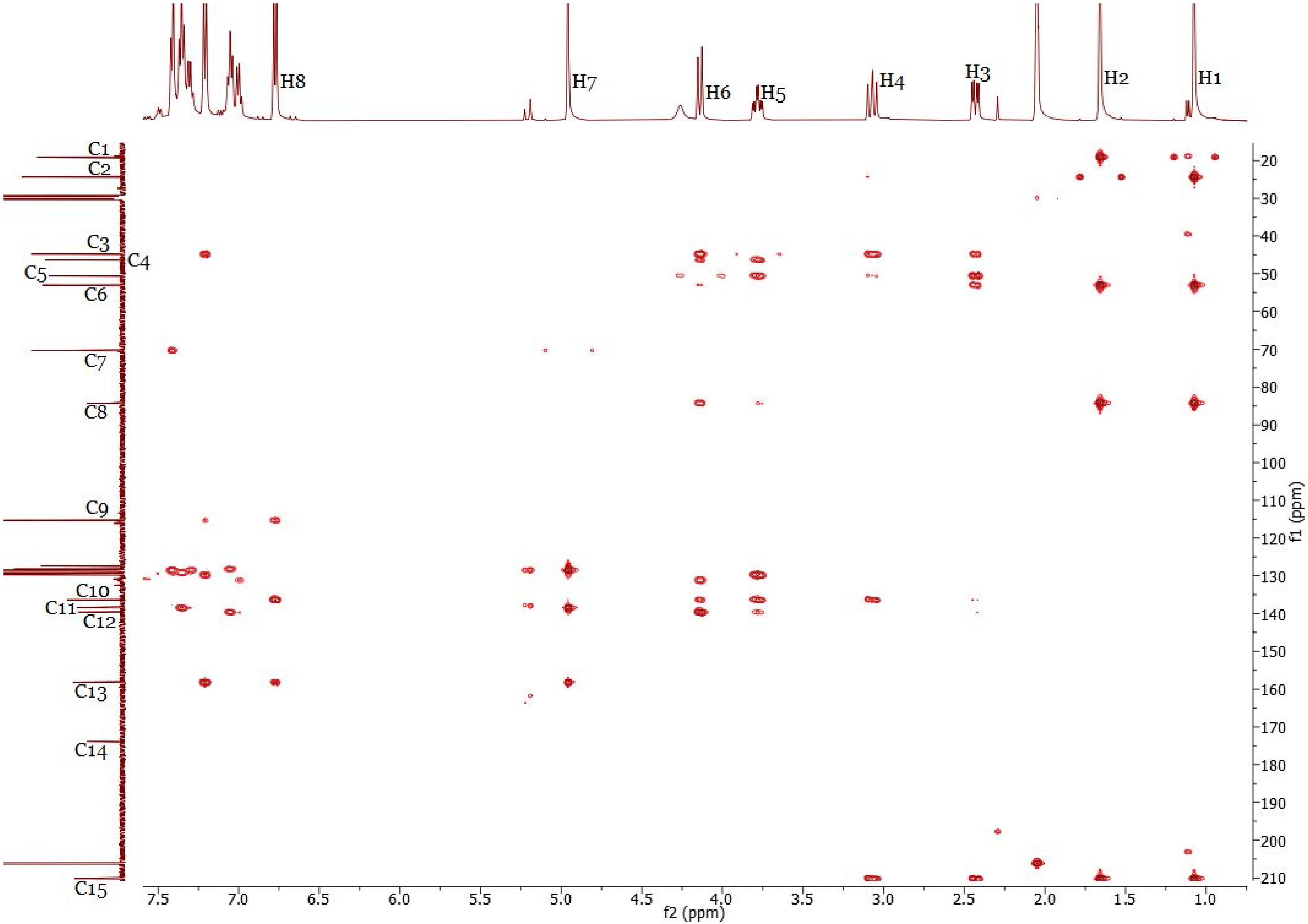

*Figure S205: HMBC spectrum of **16b**.*

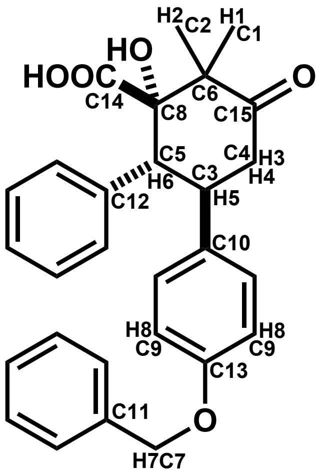

*Figure S206: 2D NMR Observations of **16b**.*

2D NMR observations of **16b**:

Protons H1 are attached to carbon C1 forming CH_3 group. The group has connectivity to carbons C2, C6, C8 and C15.

Protons H2 are attached to carbon C2 forming CH_3 group. The group has connectivity to carbons C1, C6, C8 and C15.

Protons H3 and H4 are attached to carbon C4 forming CH_2 group. The group has connectivity to carbons C3, C5, C6, C10 and C15.

Proton H5 is attached to carbon C3 forming CH group. The group has connectivity to carbons C4, C5, C8 (weak), C10 and C12 (weak). The group has connectivity inside a phenyl ring, suggesting nearby location.

Proton H6 is attached to carbon C5 forming CH group. The group has connectivity to carbons C3, C4, C6 (weak), C8, C10 (weak) and C12. The group has connectivity inside a phenyl ring, suggesting nearby location.

Protons H7 are attached to carbon C7 forming CH_2 group. The group has connectivity to carbons C11 and C13. The group has connectivity inside a phenyl ring, suggesting nearby location.

Protons H8 are attached to carbons C9 forming two CH groups. The group has connectivity to carbons C9 (itself), C10 and C13.

IR spectroscopy of **16b**

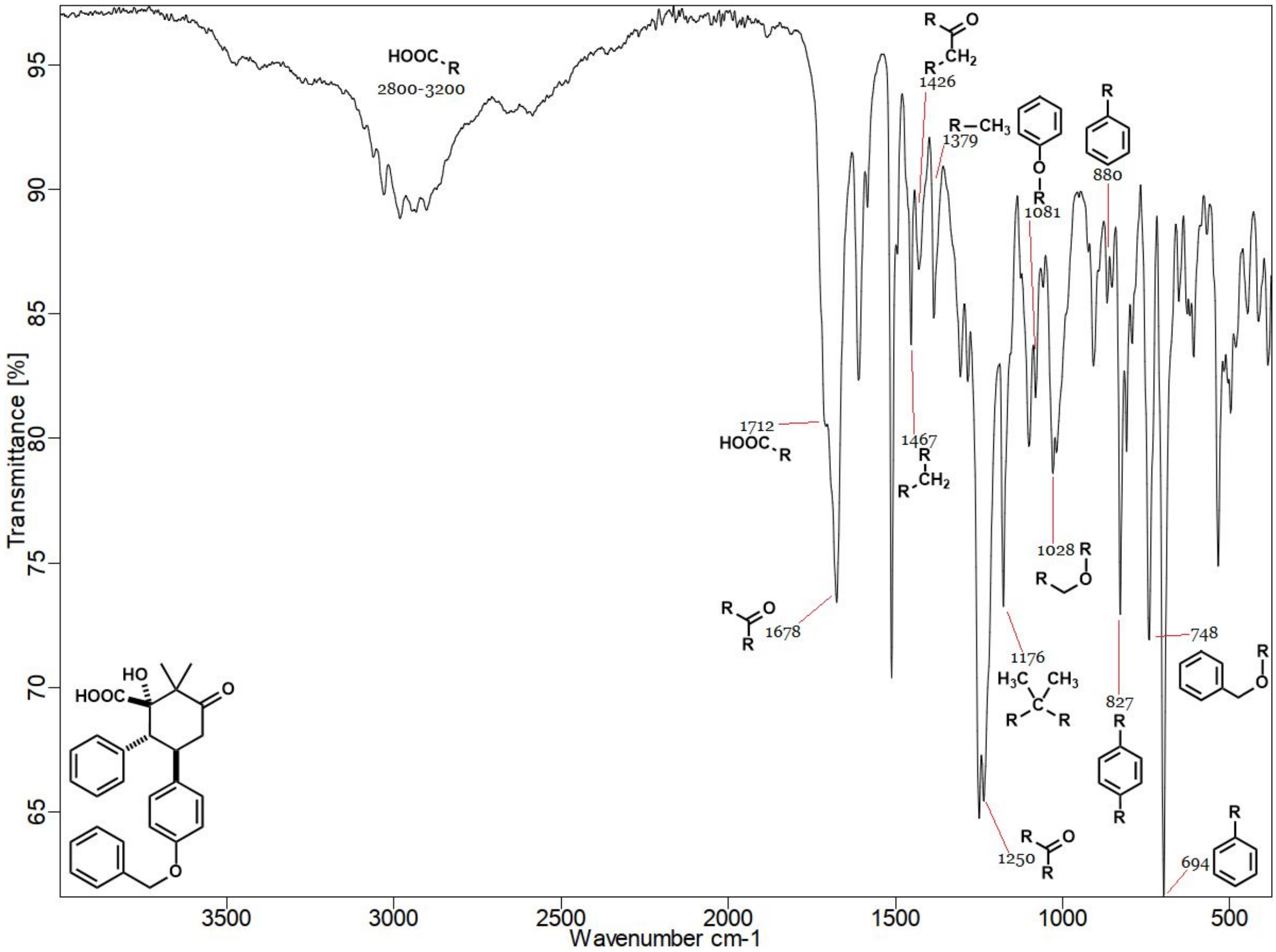

*Figure S207: IR spectrum of **16b** (2800-3200 (broad), 1712 (s) (R-COOH), 1678 (s), 1250 (s) (R-**CO**-R), 1467 (m) (R-CH_2-R), 1426 (m) (R-**CH_2**-CO-R), 1379 (m) (R-CH_3), 1176 (s) ($(CH_3)_2$-C-R_2), 1081 (m) (Ph-O-R), 1028 (m), 748 (s) (Ph-CH_2-O-R), 880 (m), 694 (s) (5 adjacent H (Ph)), 827 (m) (2 adjacent H (R-Ph-R)) cm^{-1}).*

HRMS of **16b**

HRMS (ESI-TOF) m/z: [**16b**-H]$^-$ calculated for $C_{28}H_{27}O_5$ 443.1853; Found 443.1853; Error 0.063 ppm.

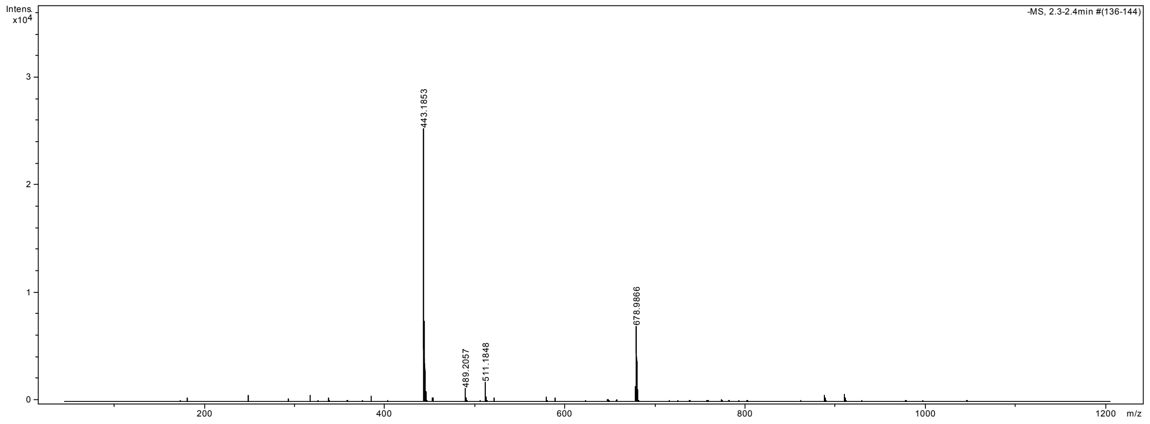

*Figure S208: ESI-TOF-MS of [**16b**-H]$^-$ (peak: 443.1853 m/z, negative-ion mode).*

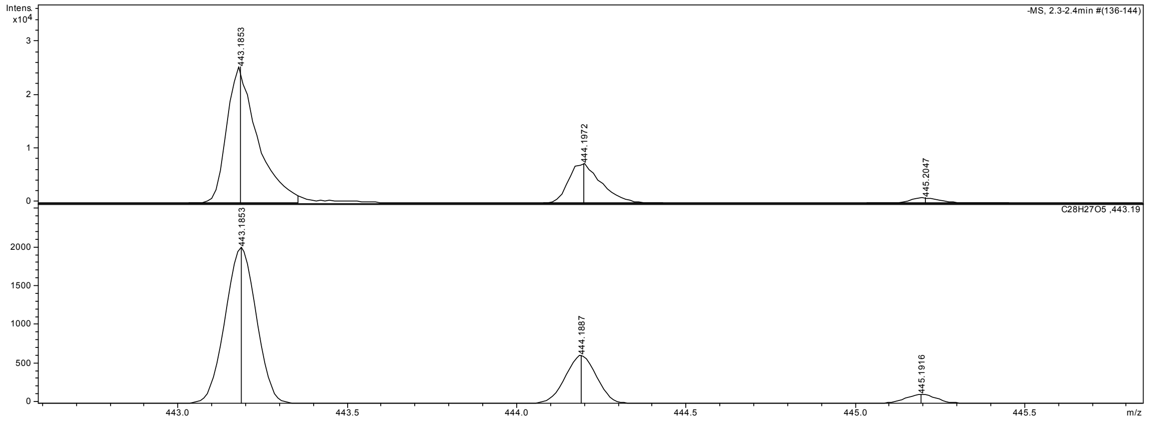

*Figure S209: Measured compound peak of [**16b**-H]$^-$ (443.1853 m/z) at top, simulated peak ($C_{28}H_{27}O_5$) below.*

4 The single-crystal X-ray diffraction studies 1a, 14A and 15a

The single-crystal X-ray diffraction study were carried out on a Bruker D8 Venture diffractometer with PhotonII CPAD detector at 173(2) K (**1a**, **14A**) or Rigaku XtaLAB Synergy-S with HyPIX-6000 detector at 123(2) K (**15a**) using Cu-Kα radiation (λ = 1.54178 Å). Dual space methods (SHELXT) [G. M. Sheldrick, *Acta Crystallogr.* 2015, **A71**, 3-8] were used for structure solution and refinement was carried out using SHELXL-2014 (full-matrix least-squares on F^2) [G. M. Sheldrick, *Acta Crystallogr.* 2015, **C71**, 3-8]. Hydrogen atoms were localized by difference electron density determination and refined using a riding model (H(O, O) free). An extinction correction were applied for **14A**. Semi-empirical absorption corrections were applied.

4.1 Single crystal growing and data

Product **1a**: Sodium salt of **1a** was dissolved into a minimal amount of methanol. A layer of isopropanol (anti solvent) was carefully placed on top of the methanol layer. The vial containing the mixture was capped and kept at room temperature. The methanol and isopropanol layers slowly diffused together facilitating a slow crystal growth. After a month, the single crystals were collected and the crystal structure was determined by X-ray diffraction.

Product **14A**: **14A** was dissolved into a minimal amount of acetone. A layer of water (anti solvent) was added on top of the acetone layer. The vial containing the growing mixture was capped and kept at room temperature. After a month, the single crystals formed and the crystal structure was determined by X-ray diffraction.

Product **15a**: **15a** was dissolved into a minimal amount of acetone. A layer of water (anti solvent) was added on top of the acetone layer. The vial containing the growing mixture was capped and kept at room temperature. After six month, the single crystals formed and the crystal structure was determined by X-ray diffraction.

1a: colourless crystals, $C_{19}H_{15}Na_2O_3 \cdot H_2O$, M_r = 314.30, crystal size 0.38 × 0.08 × 0.02 mm, triclinic, space group *P*-1 (No. 2), a = 6.2426(3) Å, b = 9.0513(4) Å, c = 14.3361(6) Å, α = 90.331(2)°, β = 93.360(2)°, γ = 100.711(2)°, V = 794.45(6) Å^3, Z = 2, ρ = 1.314 Mg/m^{-3}, μ(Cu-K$_\alpha$) = 0.95 mm^{-1}, F(000) = 328, $2\theta_{max}$ = 144.4°, 10974 reflections, of which 3116 were independent (R_{int} = 0.024), 208 parameters, R_1 = 0.034 (for 2950 I > 2σ(I)), wR_2 = 0.090 (all data), S = 1.02, largest diff. peak / hole = 0.29 / -0.19 e Å^{-3}.

14A: colourless crystals, $C_{21}H_{22}O_2$, M_r = 338.38, crystal size 0.16 × 0.08 × 0.04 mm, monoclinic, space group $P2_1$/n (No. 14), a = 14.1307(3) Å, b = 9.9607(2) Å, c = 26.9951(6) Å, β = 104.875(1)°, V = 3672.27(14) Å^3, Z = 8, ρ = 1.224 Mg/m^{-3}, μ(Cu-K$_\alpha$) = 0.68 mm^{-1}, F(000) = 1440, $2\theta_{max}$ = 144.6°, 40686 reflections, of which 7472 were independent (R_{int} = 0.062), 465 parameters, 4 restraints, R_1 = 0.043 (for 6581 I > 2σ(I)), wR_2 = 0.115 (all data), S = 1.04, largest diff. peak / hole = 0.41 / -0.24 e Å^{-3}.

15a: colourless crystals, $C_{21}H_2O_4 \cdot H_2O$, M_r = 356.40, crystal size 0.50 × 0.30 × 0.20 mm, monoclinic, space group $P2_1$/c (No. 14), a = 10.7557(1) Å, b = 14.0138(1) Å, c = 11.8249(1) Å, β = 99.386(1)°, V = 1758.48(3) Å^3, Z = 4, ρ = 1.346 Mg/m^{-3}, μ(Cu-K$_\alpha$) = 0.78 mm^{-1}, F(000) = 760, $2\theta_{max}$ = 159.6°, 34279 reflections, of which 3759 were independent (R_{int} = 0.038), 249 parameters, 3 restraints, R_1 = 0.038 (for 3622 I > 2σ(I)), wR_2 = 0.100 (all data), S = 1.03, largest diff. peak / hole = 0.30 / -0.43 e Å^{-3}.

CCDC 2193907 (**1a**), 2193908 (**14A**), and 2193909 (**15a**) contain the supplementary crystallographic data for this paper. These data can be obtained free of charge from The Cambridge Crystallographic Data Centre via www.ccdc.cam.ac.uk/data_request/cif.

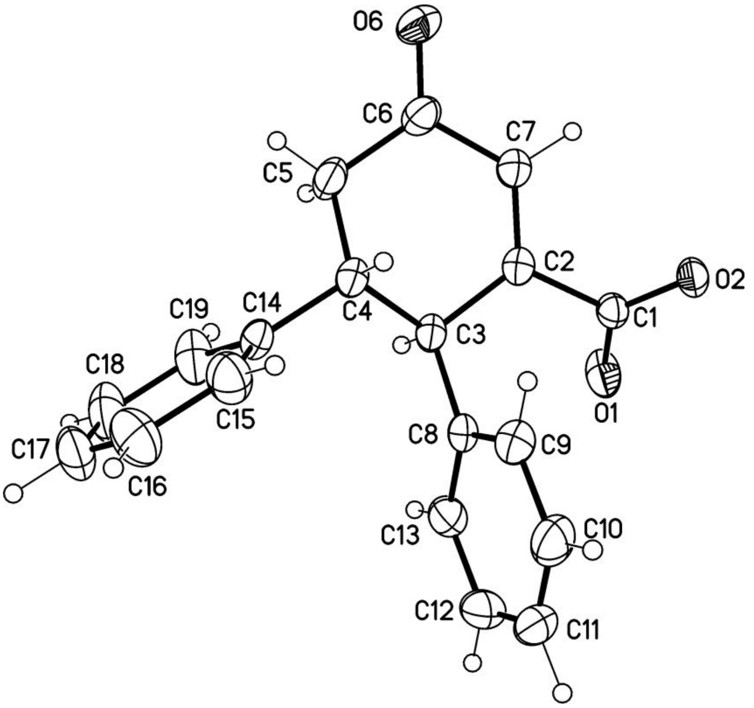

*Figure S210: Molecular structure of the anion of **1a** (displacement parameters are drawn at 50 % probability level).*

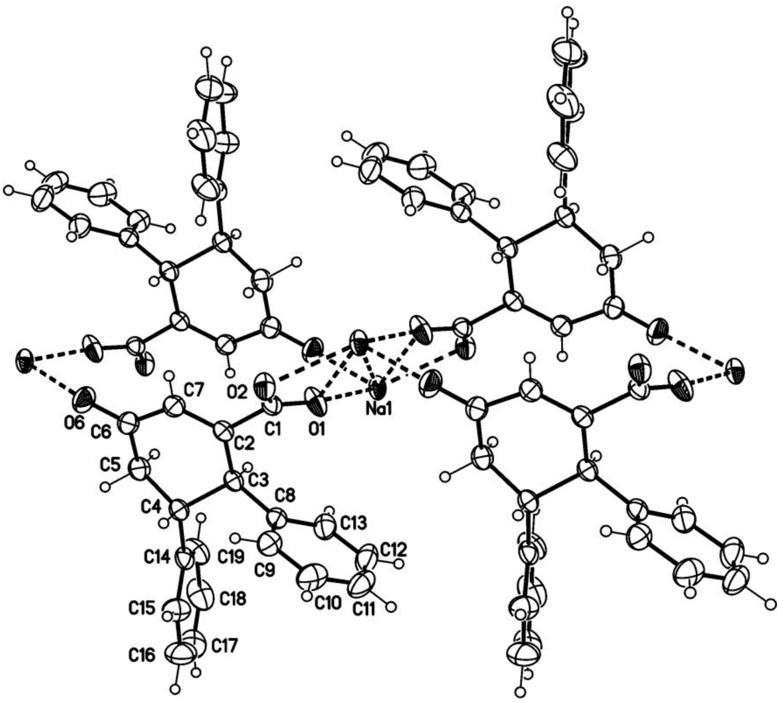

*Figure S211: Structure of **1a** (part of the polymeric 'close ion pair' shown, displacement parameters are drawn at 50 % probability level).*

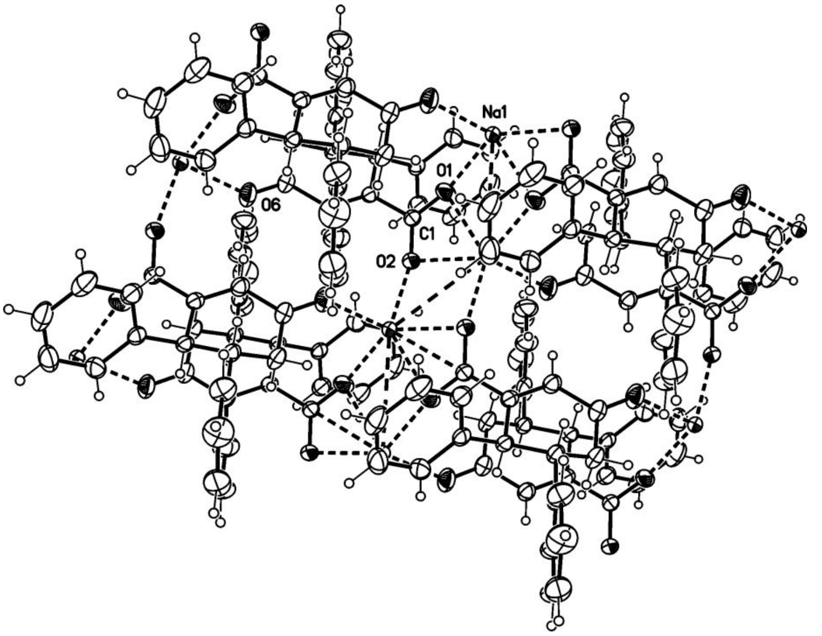

*Figure S212: Structure of **1a** (part of the polymeric 'close ion pair' shown, displacement parameters are drawn at 50 % probability level).*

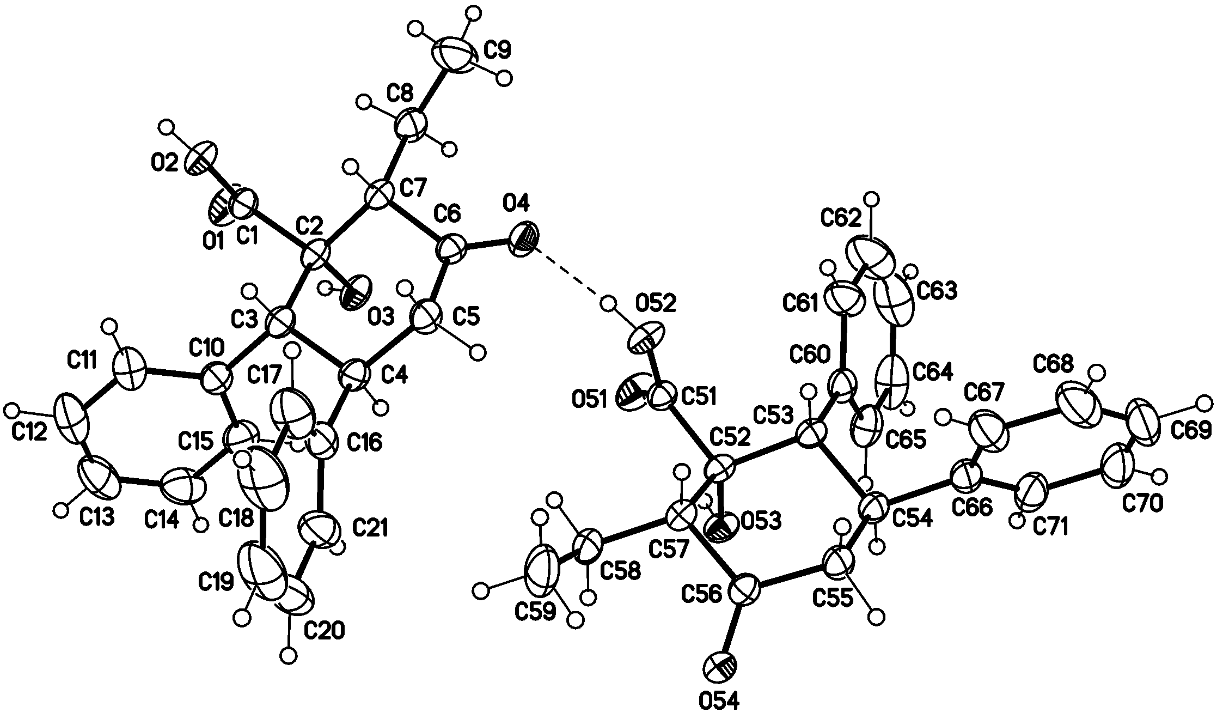

*Figure S213: Structure of **14A** (asymmetric unit shown, displacement parameters are drawn at 50 % probability level).*

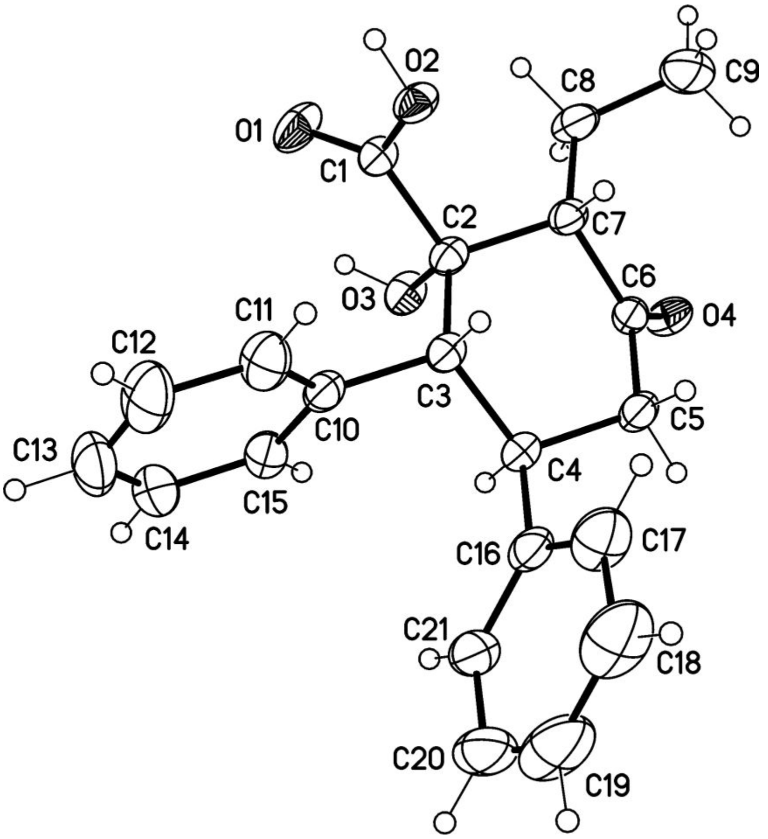

*Figure S214: Structure of the 1st crystallographic independent molecule of **14A** (displacement parameters are drawn at 50 % probability level).*

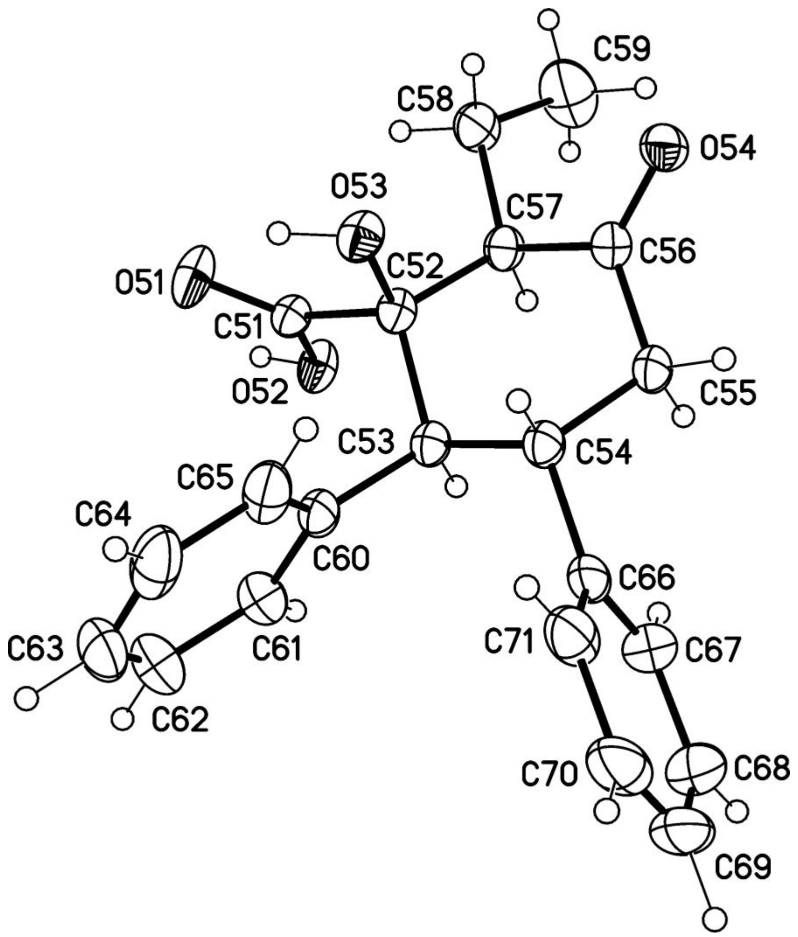

*Figure S215: Structure of the 2nd crystallographic independent molecule of **14A** (displacement parameters are drawn at 50 % probability level).*

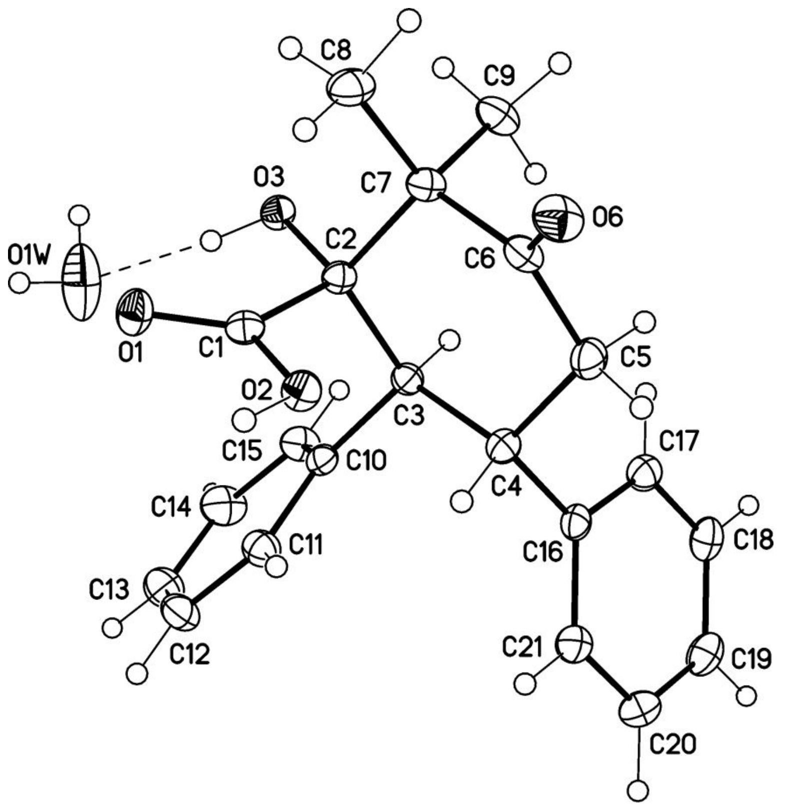

*Figure S216: Molecular structure of **15a** (displacement parameters are drawn at 50 % probability level).*
