# Supplementary figures and images for: Stereospecific Synthesis of Cyclohexenone Acids by [3,3]-Sigmatropic Rearrangement Route
Source: J Org Chem. 2023 Sep 1;88(18):12914–23. doi: 10.1021/acs.joc.3c00757 (PMC10507681; doi:10.1021/acs.joc.3c00757)

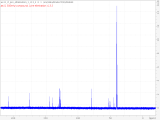

Supplement: Supplementary file 2 — jo3c00757_si_002.zip [file jo3c00757_si_002.zip › FID for Publication/1 big A/C13/6/pdata/1/thumb.png]

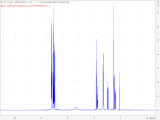

Supplement: Supplementary file 2 — jo3c00757_si_002.zip [file jo3c00757_si_002.zip › FID for Publication/1 big A/H1/1/pdata/1/thumb.png]

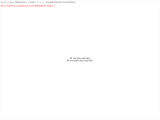

Supplement: Supplementary file 2 — jo3c00757_si_002.zip [file jo3c00757_si_002.zip › FID for Publication/1 big A/HMBC/5/pdata/1/thumb.png]

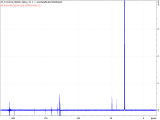

Supplement: Supplementary file 2 — jo3c00757_si_002.zip [file jo3c00757_si_002.zip › FID for Publication/10/13C/4/pdata/1/thumb.png]

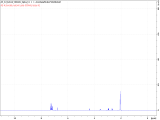

Supplement: Supplementary file 2 — jo3c00757_si_002.zip [file jo3c00757_si_002.zip › FID for Publication/10/1H/1/pdata/1/thumb.png]
